# Sleep disturbances and the risk of lung cancer: a meta-epidemiological study

**DOI:** 10.1186/s12885-023-11392-2

**Published:** 2023-09-19

**Authors:** Tong Zhou, Zichen Wang, Chenxi Qiao, Shuo Wang, Shuaihang Hu, Xinyan Wang, Xiumei Ma, Dandan Wang, Jinglei Li, Zheng Li, Wei Hou

**Affiliations:** 1 Department of Oncology, China Academy of Chinese Medical Sciences Guang’anmen Hospital, Beixian’ge Street No. 5 Xicheng District, Beijing, China; 2 Graduate school, Beijing University of Chinese Medicine, Beijing, China

**Keywords:** Sleep disturbances, Lung neoplasm, Epidemiological study, Meta

## Abstract

**Background:**

The relationship between sleep disturbances and lung cancer is complex and bidirectional. This meta-epidemiological study aimed to explore the potential association between sleep disruption and the risk of pulmonary cancer.

**Methods:**

We conducted a comprehensive literature search of the PubMed, Embase, Cochrane Library, and Web of Science databases to retrieve relevant studies. We employed the Newcastle–Ottawa Scale to assess the quality of the observational studies. Stata 17.0 was used to synthesize and conduct a meta-analysis of odds ratios (ORs) and corresponding 95% confidence intervals (CIs). We used funnel plot analysis and Egger’s regression test to evaluate potential publication bias.

**Results:**

A total of 11 studies were included with 469,691 participants. The methodological quality of the included studies ranged from moderate to high. Compared with 7–8 h of sleep time, short sleep duration was associated with a 13% higher lung cancer risk [OR, 1.13; 95%CI: 1.02–1.25; I^2^ = 67.6%; *P* = 0.018] and long sleep duration with a 22% higher risk [OR, 1.22; 95%CI: 1.12–1.33; I^2^ = 6.9%; *P* < 0.001]. Insomnia symptoms [OR, 1.11; 95%CI: 1.07–1.16; I^2^ = 0%; *P* < 0.001] and evening chronotype [OR, 1.15; 95%CI: 1.05–1.26;* P* = 0.002] were all related to a higher risk of lung cancer. Egger’s test revealed no publication bias for sleep duration (*P* = 0.13).

**Discussion:**

This systematic review is the first one which observes positive correction between sleep disturbances and the incidence of lung cancer. While the plausible mechanism is not clear, it is hypothesized that the association of short sleep duration and lung cancer mainly mediated by melatonin secretion and the immune-inflammatory balance. Further studies are needed to examine whether other risk factors, such as age, occupation, cumulative effect of sleep disturbances might mediate the relationship between sleep disturbances and lung cancer risk.

**Conclusion:**

The present study revealed that insufficient and excessive sleep duration, insomnia symptoms, and evening chronotype were significantly predictive of an increased risk of lung cancer. This finding underscores the need to account for sleep disturbances as an independent risk factor for evaluating susceptibility to lung cancer.

**Trial registration:**

CRD42023405351.

**Supplementary Information:**

The online version contains supplementary material available at 10.1186/s12885-023-11392-2.

## Background

Lung cancer, the second most prevalent form of cancer, remains a significant health challenge and threat to life worldwide [1]. Studies indicate that the 5-year survival rate of patients with lung cancer ranges from 10 to 20% in most countries [2]. Moreover, lung cancer became the primary cause of cancer-related mortality in 2020, with an estimated 1.8 million deaths accounting for 18% of all fatalities globally [3]. Therefore, the etiology and pathogenesis of lung cancer have garnered considerable attention.

Sleep disturbance is a disorder that encompasses difficulties in sleep initiation and maintenance, or excessive sleepiness [4]. A study in Sweden revealed that approximately 20–30% of adults frequently experience sleep disturbances [5]. Recent research contends that sleep disturbance may serve as a risk factor for breast, colorectal, and gastric cancer [6–8]. Multiple studies have suggested that sleep disturbances could be associated with cancer, specifically through the disruption of circadian rhythms, reduction of melatonin secretion, and inflammatory responses, leading to unregulated cell proliferation [9–11].

Animal studies have shown that circadian disruption can promote lung cancer tumor growth and metastasis in male C57BL/6 mice by affecting the expression of tumor-related genes [12]. According to a previous cross-sectional study, the concentration of melatonin in patients with non-small cell lung cancer was significantly lower than that in healthy volunteers [13]. Chen et al. found that melatonin has a good anticancer effect by inhibiting lung cancer growth in a Lewis mouse model [14]. Accordingly, we propose an underlying association between lung cancer risk and sleep disturbances, which may be related to circadian rhythms and melatonin secretion.

However, few pooled analyses have explored the effects of multiple sleep parameters on the occurrence of lung cancer, resulting in inconsistent results. Detecting sleep features associated with the occurrence of lung cancer may enable the identification of individuals at risk for this disease and promote primary prevention efforts. Therefore, this meta-analysis aimed to quantitatively assess the relationship between sleep disturbances and lung cancer to provide valuable insights into future lung cancer prevention efforts.

## Methods

This study was conducted according to the Preferred Reporting Items for Systematic Reviews and Meta-Analyses (PRISMA) statements [15]. The protocol for this meta-analysis was registered with the International Prospective Register of Systematic Reviews under the registration number CRD42023405351.

### Data sources

To identify studies exploring the potential links between sleep disturbances and lung cancer risk, we conducted a comprehensive search of four electronic databases (PubMed, Embase, Cochrane Library, and Web of Science) covering studies published up to March 5, 2023. Additionally, we manually screened review article reference lists for relevant reports.

The search strategy encompassed two themes that delineated the diverse sleep characteristics during both physiological and pathological conditions as exposure variables, while considering lung cancer risk as the outcome variable. No restrictions were established concerning study types or geographic boundaries to enhance the comprehensiveness of the literature retrieval. The search string used for sleep disturbances contained: "Sleep [Mesh]","Sleep Initiation and Maintenance Disorders [Mesh] ","Sleep Wake Disorders [Mesh] ","Sleep* Habit*","Nap*","Daytime sleep", "Siesta", "Daytime sleepiness", "Daytime somnolence", "Insomnia*","Disorders of Initiating and Maintaining Sleep", "Early Awakening". The following terms are used for lung cancer: "Lung Neoplasm [Mesh]","Pulmonary Neoplasm*","Lung* Neoplasm*","Lung Cancer*", " Pulmonary Cancer*."

### Eligibility criteria

The present study included original literature that met the following inclusion criteria:1) observational studies utilizing a cross-sectional, case–control, retrospective or prospective cohort design; 2) investigations that explored the implications of sleep disturbances, such as insufficient or excessive sleep duration, insomnia symptoms, and subjective breathing disorders, on the development of lung cancer; 3) sleep disruption was determined through self-reported questionnaires, face-to-face questioning, or clinical diagnosis in patients experiencing symptoms of sleep disorders; and 4) studies that provided risk estimates, including odds ratios (ORs), risk ratios (RRs), hazard ratios (HRs) with 95% confidence intervals (95% CIs), or adequate data for computing.

The exclusion criteria were as follows: 1)case studies, reports, reviews, conference abstracts, or genetic, animal and cell studies;2) assessing sleep disorders after lung cancer; 3) the cohort was examined for multiple cancer types, but no subgroup analyses were conducted specifically for lung cancer; 4) only survival or mortality was used as the outcome measure;5) outcome measures was compound unit about the length of population survival time; and 6) studies that reported repeated outcome measures within the overlapping sample, which had a small sample size.

### Study selection

Two researchers (WZC and QCX) performed primary screening by reading the study titles and abstracts, and then downloaded and browsed the full text of potentially eligible studies to determine the final literature that met the eligibility criteria. When the two researchers disagreed on the selection, the decision was made through discussion and consultation with a third researcher (ZT).

### Data extraction

Two researchers (WZC and QCX) independently collected information using a predesigned checklist. In case of disputes, the final decision was made by the researchers (ZT and WS) after a full-text review. The checklist aimed to obtain details such as publication information (first author, publication year, country), study features (type of study, follow-up year, mean age/age range, number of cases, and control group), exposure and outcome diagnostic criteria, sleep disturbance categorization, subgroup stratifications (including specific sleep duration, insomnia symptoms, morning/evening chronotype, and subjective sleep-disorder breathing features), adjustment for confounding factors, and effect sizes with corresponding 95% CIs (effect sizes adjusted for all the available covariates were selected). To ensure a consistent definition of sleep duration, sleep durations of less than 6 h, less than 6.5 h, or 7 h were categorized as short durations, while durations exceeding 8 h were categorized as long durations. Furthermore, to eliminate the possible influence of newly diagnosed lung cancer on physiological state and sleep duration, we prioritized the statistical results after eliminating cases diagnosed within 2 years of baseline.

### Risk of bias

Two independent researchers (HSH and WXY) used the Newcastle–Ottawa scale [16] to assess the quality of the studies, which were classified into three levels: low (7 or more), moderate (4–6), and high risk (3 or less) of bias.

### Statistical analysis

Pooled ORs and corresponding 95% CIs were calculated as effect measures for lung cancer risk irrespective of the study design. Owing to the low incidence of lung cancer within populations, ORs were considered equivalent to RRs and HRs in this meta-analysis. In the presence of significant heterogeneity (*P* < 0.1 or *I*^2^ > 50%), a random-effects model was used; otherwise, a fixed-effects model was used. Sensitivity analyses were performed to assess the robustness of our pooled results and to examine the influence of individual studies on the overall estimates. Publication bias was evaluated using funnel plots and Egger’s test. All statistical analyses were conducted using Stata software (version 17.0).

## Results

### Study selection

**Fig. 1:**
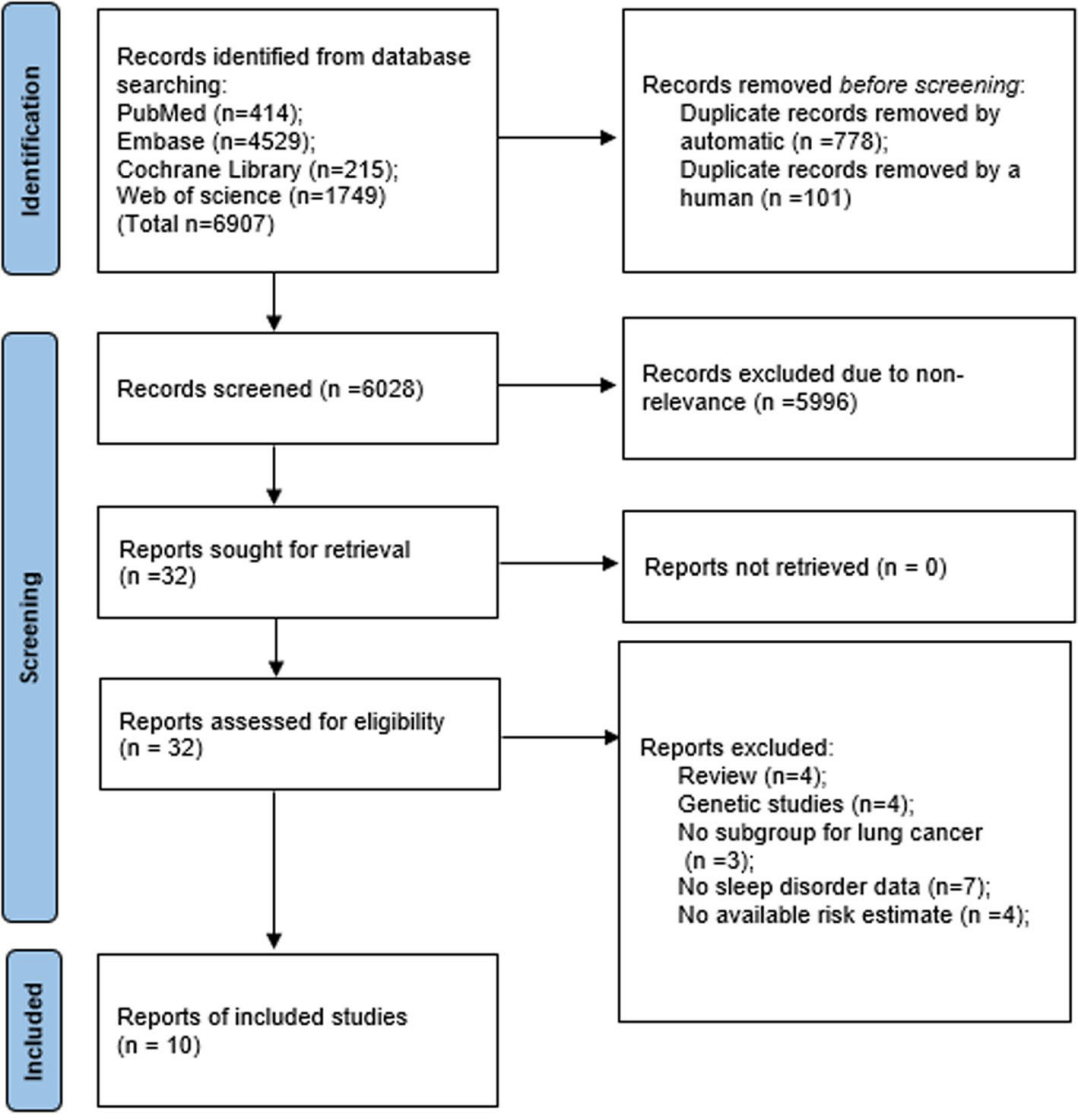
Flow diagram of the search strategy and study selection

As presented in Fig. 1, 6907 records were retrieved from the electronic databases. After removing duplicates using an automatic tool and manual searches, 6028 articles were included in the initial screening. Of these, 5996 articles were excluded because of untargeted participants or outcome data. Finally, 10 studies [17–26] reached eligibility criteria after browsing the full‐text.

### Study characteristics

The ten studies were published between 2014 and 2022, with sample sizes ranging from 950 to 469,691 participants (8,288 with lung cancer events). Eight studies [17, 19–25] were cohort studies, and two [18, 26] were case–control studies. Most studies were conducted in Europe [19, 22, 24, 26] and America [21, 23, 25], two in Canada [18, 20], and one in China [17]. Self-reported questionnaires were the most widely used method for assessing sleep parameters; only one study [17] used face-to-face questioning. The diagnosis of lung cancer was verified through cancer registries or medical records and classified according to the International Classification of Diseases (ICD 9 or 10). All studies showed adjusted HRs or ORs with corresponding 95% CIs; however, the adjusted confounders varied slightly between the studies. The characteristics of the included studies are summarized in Table 1.

**Table 1:** Characteristics of included studies

Authors, year	Study design	Country	Sample size	Mean age/ age range at baseline (y)	Follow-up duration (y)	Sleep variables	Sleep variable assessment scale	Sleep duration	Adjusted confounding factors	NOS scores
Owais et al. 2014 [23]	Cohort	America	T:21,026 LC:150	68.3 ± 8.8	7.5 ± 2.2	Sleep duration	Self-reported questionnaire	≤ 6, 6–8, ≥ 8	Age, race, parental history of cancer, exercise frequency, caloric intake, body mass index, type 2 diabetes, alcohol intake, sleep apnea, snoring, smoking status	7
Maria et al. 2014 [22]	Cohort	Finland	T:2,586 LC:81	42– 60	23	Sleep duration; IS; S-SDB	Self-reported questionnaire	≤ 6.5, 7–7.5, ≥ 8	Age, examination years, cumulative smoking history, family cancer history	9
Susan et al. 2015 [21]	Cohort	California	T:101,609 LC:728	52	NA	Sleep duration	Self-reported questionnaire	≤ 6,7–9, ≥ 10	Race, BMI, physical activity, alcohol intake, comorbidity, smoking status, neighborhood urbanization	6
McNei et al. 2019 [20]	Cohort	Alberta	T:45,984 LC:62	35–69	7.47 ± 3.25	Sleep duration; chronotype	Self-reported questionnaire; Updated Health and Lifestyle Questionnaire	< 7, 7–9, > 9	Sex, BMI, presence of medical conditions/comorbidities, alcohol intake, menopausal status	7
Xie et al. 2021 [19]	Cohort	United Kingdom	T: 469,691 LC: 2,177	Cancer: 61.86 ± 5.78; Noncancer:56.29 ± 8.11	7.13	Sleep duration; IS; S-SDB; chronotype	Standardized questionnaire and standardized interview	< 7, 7–8, > 8	Age, sex, race, smoking status, alcohol intake, education, physical activity, BMI, sleep medication, history of consulting for mental health, respiratory comorbidities, sleep apnea	8
Cao et al. 2022 [17]	Cohort	China	T:60,443 LC:407	47.0 ± 17.3	9.9 ± 4.8	Sleep duration; IS;S-SDB	Face-to-face questioning	≤ 7, 7–10, ≥ 10	Sex, education, occupation, annual household income, smoking, physical activity level, alcohol intake, and intake of various foods and fruits, BMI	7
Noah et al. 2022 [24]	Cohort	United Kingdom	T:382,996 LC:3,664	Media: 58 years	11.43	Sleep duration; IS; chronotype	Self-reported questionnaire	< 7, 7–8, < 8	sex, race, BMI, coffee and tea consumption, Smoking status, alcohol use, age, solid fuel cooking/heating, respiratory comorbidities, education, and genetic kinship	8
Emilie et al. 2022 [26]	Case–control	Ile-de-France region	T:1,474 LC:716	18–75	NA	Sleep duration; chronotype	Self-reported questionnaire	≤ 6, 6–8, ≥ 8	Age, area of residence, marital status, socio-economic status, BMI	6
Arthur et al. 2022 [25]	Cohort	America	T:4,580 LC:113	73	12	S-SDB; IS	Self-reported questionnaire	NA	Age, sex, enrollment wave, smoking, BMI, diabetes, physical activity levels, alcohol consumption	6
Rachel et al. 2022 [18]	Case–control	Canadian	T:950 LC:190	58 ± 8.62	NA	Sleep duration	Self-reported questionnaire	≤ 6, 6–9, ≥ 9	Age, sex, regional cohort, income	7

*T* Total number of participants, *IS* Insomnia symptoms, *S-SDB* Subjective sleep disordered breathing, *ICD* International Classification of Diseases, 10th revision, *NA* Not available, *BMI* Body mass index

### Quality assessment

According to Newcastle–Ottawa Scale, the mean score of included studies was 7.00 ± 0.92, with a media of 7. Seven of the studies were judged as having a low risk of bias and three as having a moderate risk, indicating that the methodological quality of the included studies was medium or high. Eight studies [18, 20–26] used a self-reported questionnaire as the main assessment scale, which was considered to have a selection bias.

### Short sleep duration and lung cancer risk

**Fig. 2:**
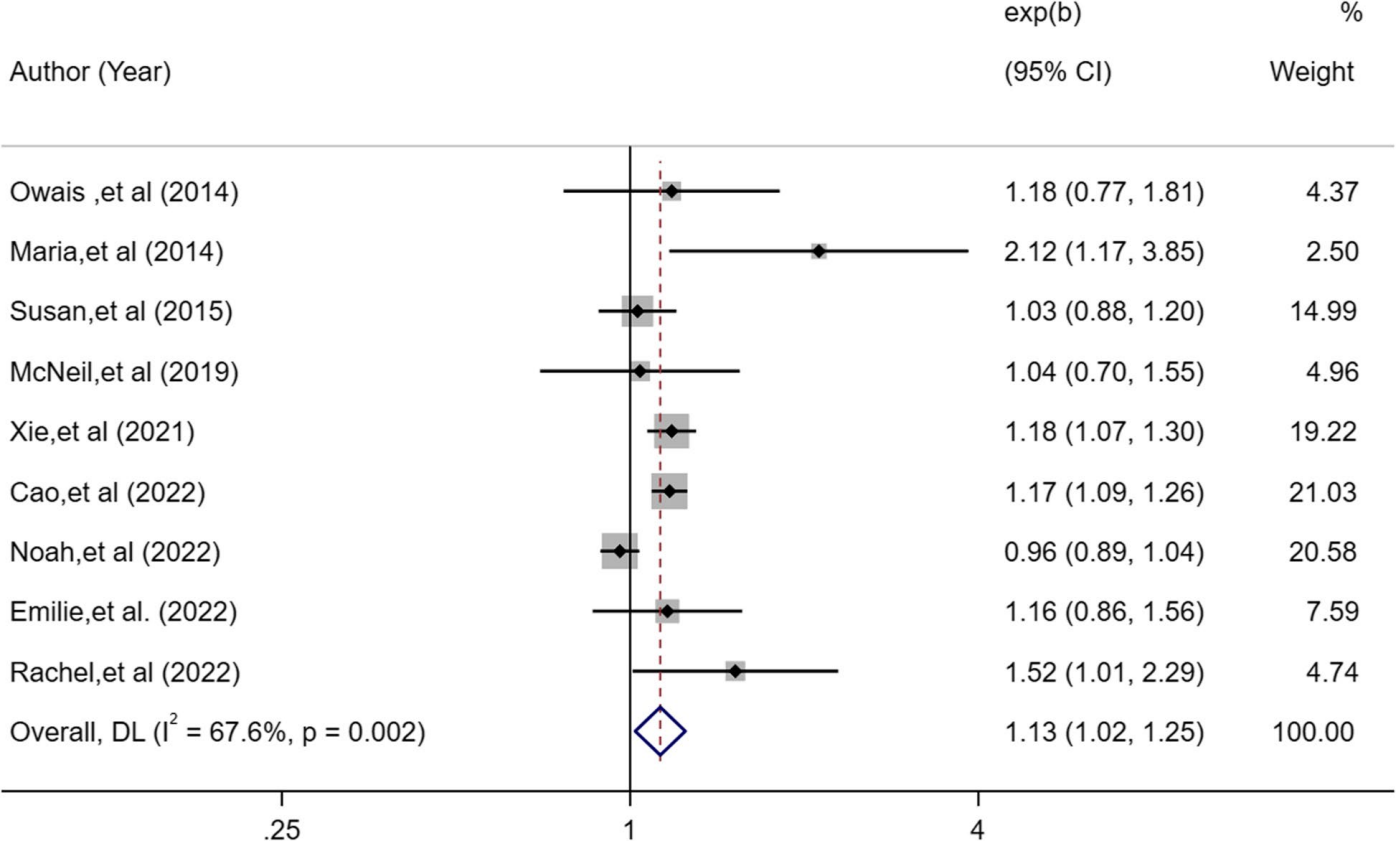
Forest plot of association between short sleep duration and cancer risk

Nine eligible studies [17–24, 26] assessed the relationship between short sleep duration and the risk of lung cancer. The OR for short duration associated with the risk of lung cancer was 1.13 in a random-effects model (95%CI: 1.02–1.25, *I*^2^ = 67.6%, *P* = 0.018; Fig. 2). There was high heterogeneity in the short duration; therefore, we performed a sensitivity analysis. The sensitivity analysis suggested that removing Noah’s study [24] as an outlier significantly reduced heterogeneity (*I*^2^ = 15.9%,* P* = 0.002) but had no effect on the pooled OR (OR, 1.16; 95%CI: 1.11–1.23; Fig. 2). The specificity of this study is that it excluded participants who reported shift work at baseline and did not adjust for occupation. The discrepancy in the inclusion of participants may be the main reason why this is inconsistent with the conclusions regarding short sleep duration in other studies.

### Long sleep duration and lung cancer risk

**Fig. 3:**
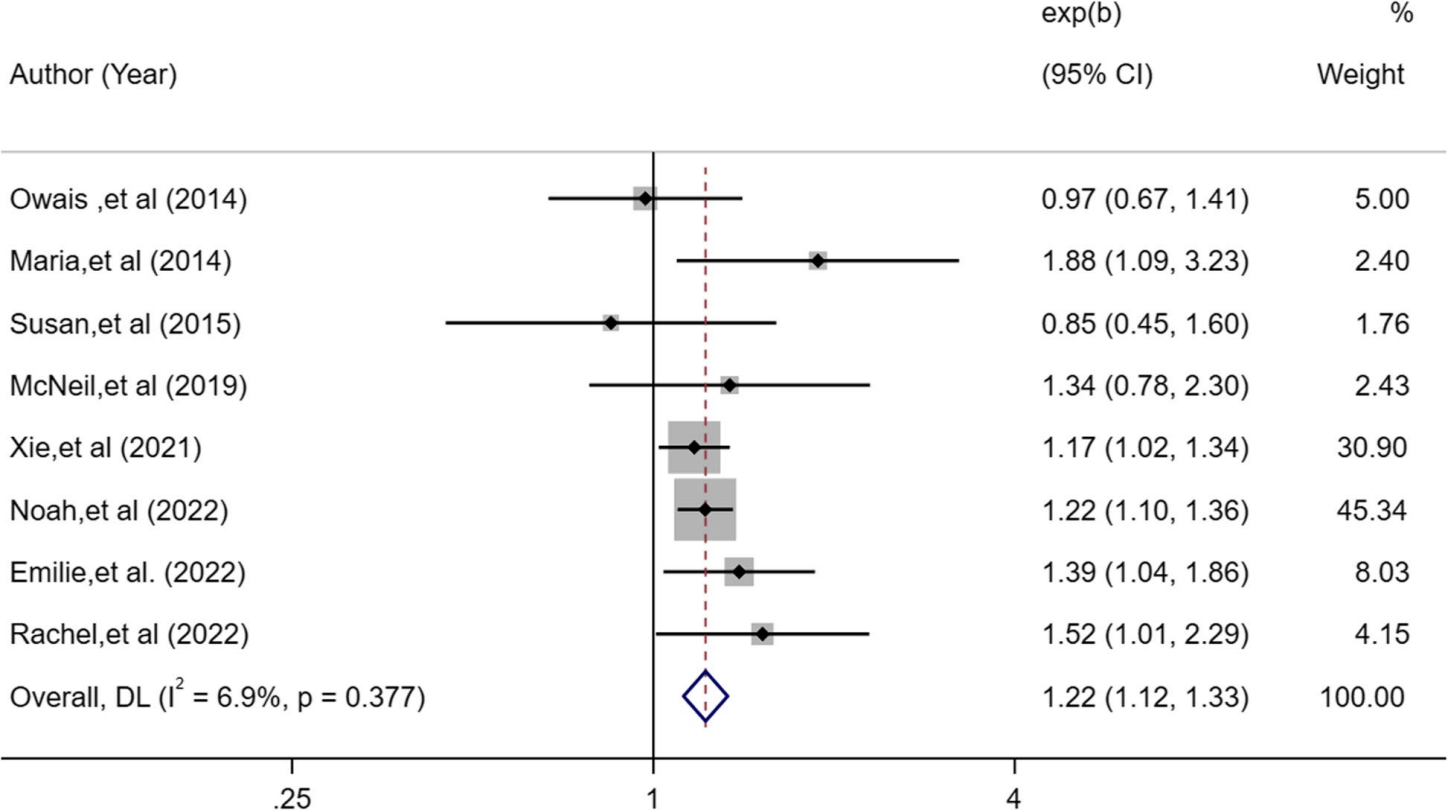
Forest plot of association between long sleep duration and cancer risk

Eight eligible studies [18–24, 26] assessed the predictive association between long sleep disturbances and incidence of lung cancer. Individuals who reported long sleep duration had a 1.22-fold higher risk of developing lung cancer than subjects with normal sleep duration in a fixed-effects model (95%CI: 1.12–1.33, *I*^2^ = 6.9%, *P* < 0.001; Fig. 3).

### Insomnia symptoms and lung cancer risk

**Fig. 4:**
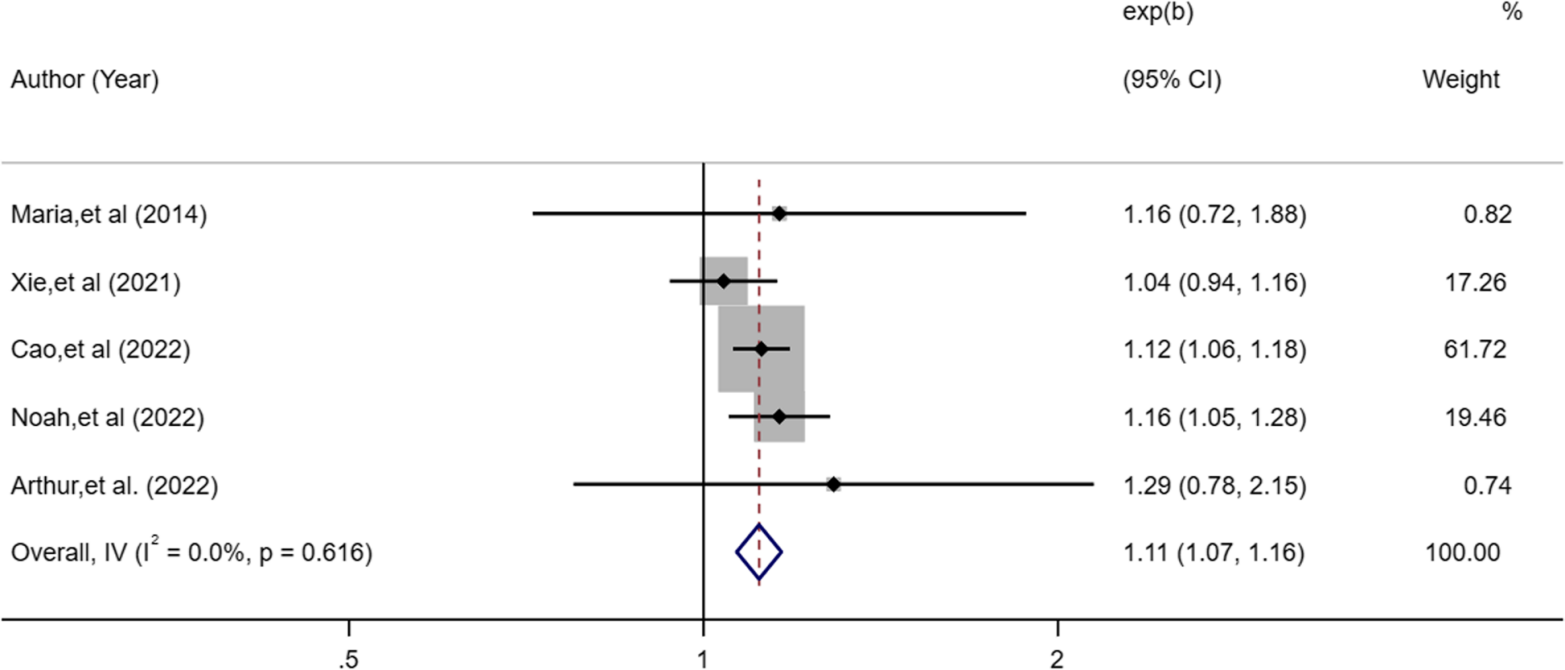
Forest plot of association between insomnia symptoms and cancer risk

The meta-analysis of five eligible studies [17, 19, 22, 24, 25] exploring the association between insomnia symptoms and lung cancer, including 903,731 insomnia patients, indicated that insomnia symptoms increased the risk of lung cancer (OR, 1.11; 95%CI: 1.07–1.16,* P* < 0.001; Fig. 4), which showed low heterogeneity (*I*^2^ = 0%).

### Chronotype and lung cancer risk

**Fig. 5:**
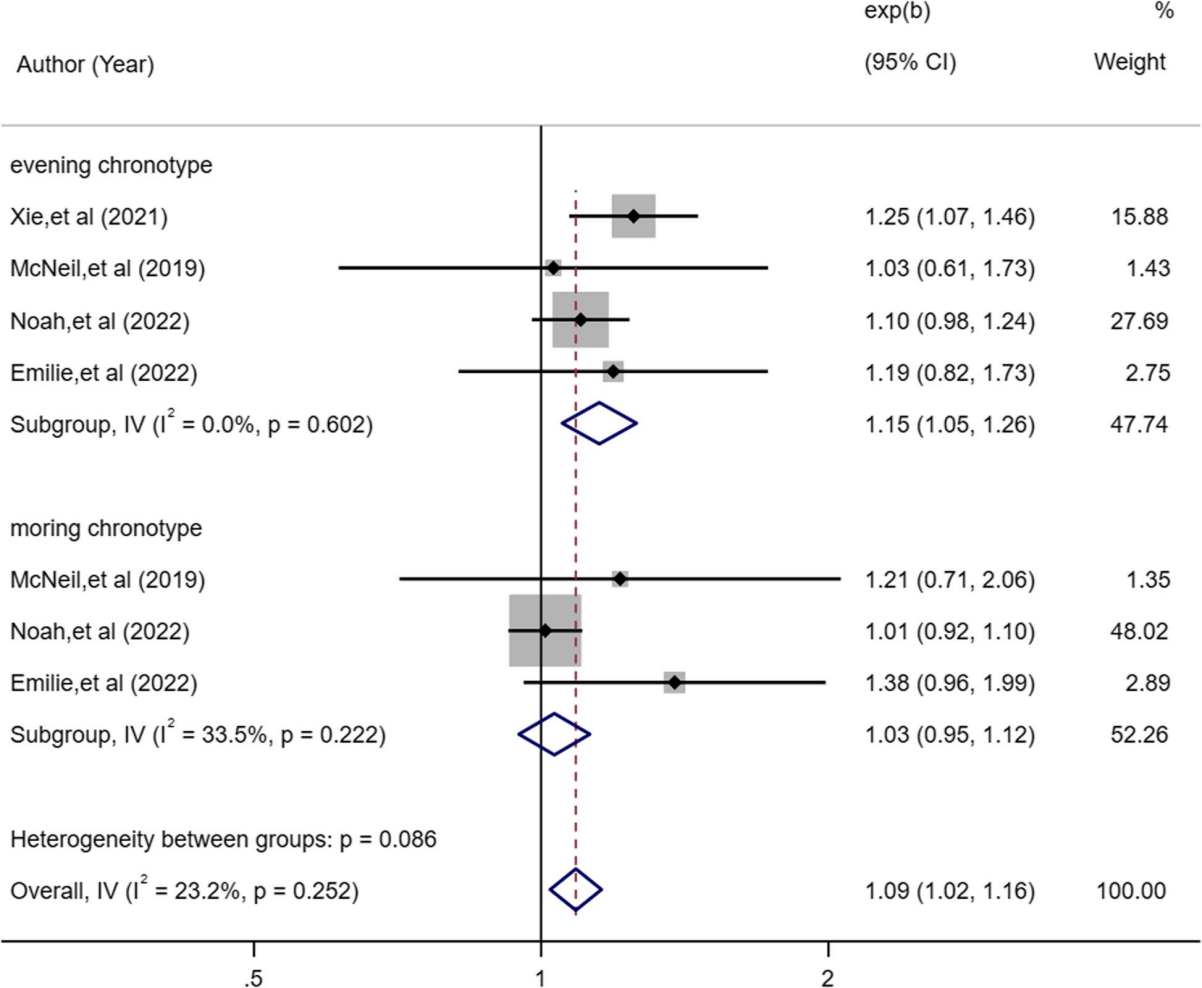
Forest plot of association between chronotype and cancer risk

Four eligible studies [19, 20, 24, 26] provided pooled estimates of morning and evening chronotypes and lung cancer. The incidence of lung cancer was associated with evening chronotype (OR, 1.15; 95%CI, 1.05–1.26, *P* = 0.002; Fig. 5), but not with morning chronotype (OR, 1.03; 95%CI, 0.95–1.12, *P* = 0.466; Fig. 5).

### Sleep disordered breathing and lung cancer risk

**Fig. 6:**
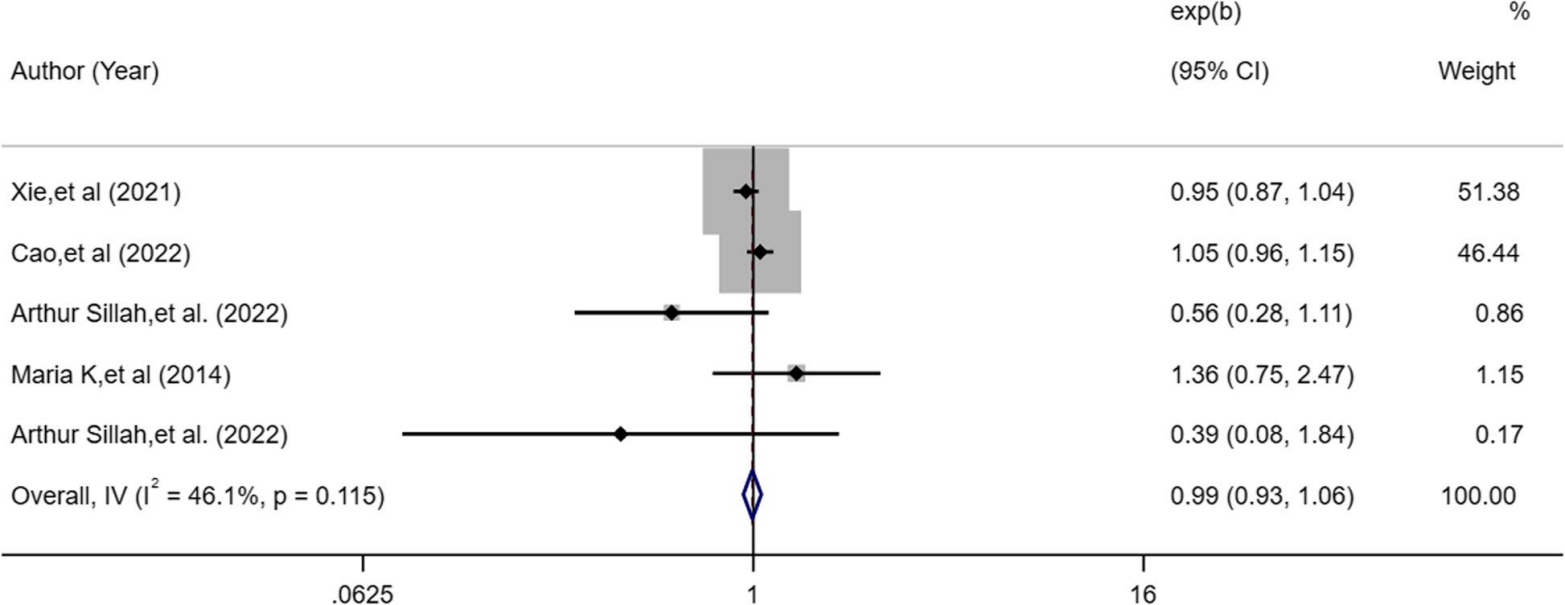
Forest plot of association between subjective sleep disordered and cancer risk

Four studies [17, 19, 22, 25] described exposure variables about subjective sleep disordered breathing. The random-effects model revealed that overall subjective sleep disordered breathing did not increase the risk of lung cancer (OR, 0.99; 95%CI: 0.932–1.059; *I*^2^ = 46.1%, *P* = 0.836; Fig. 6).

### Publication bias

**Fig. 7:**
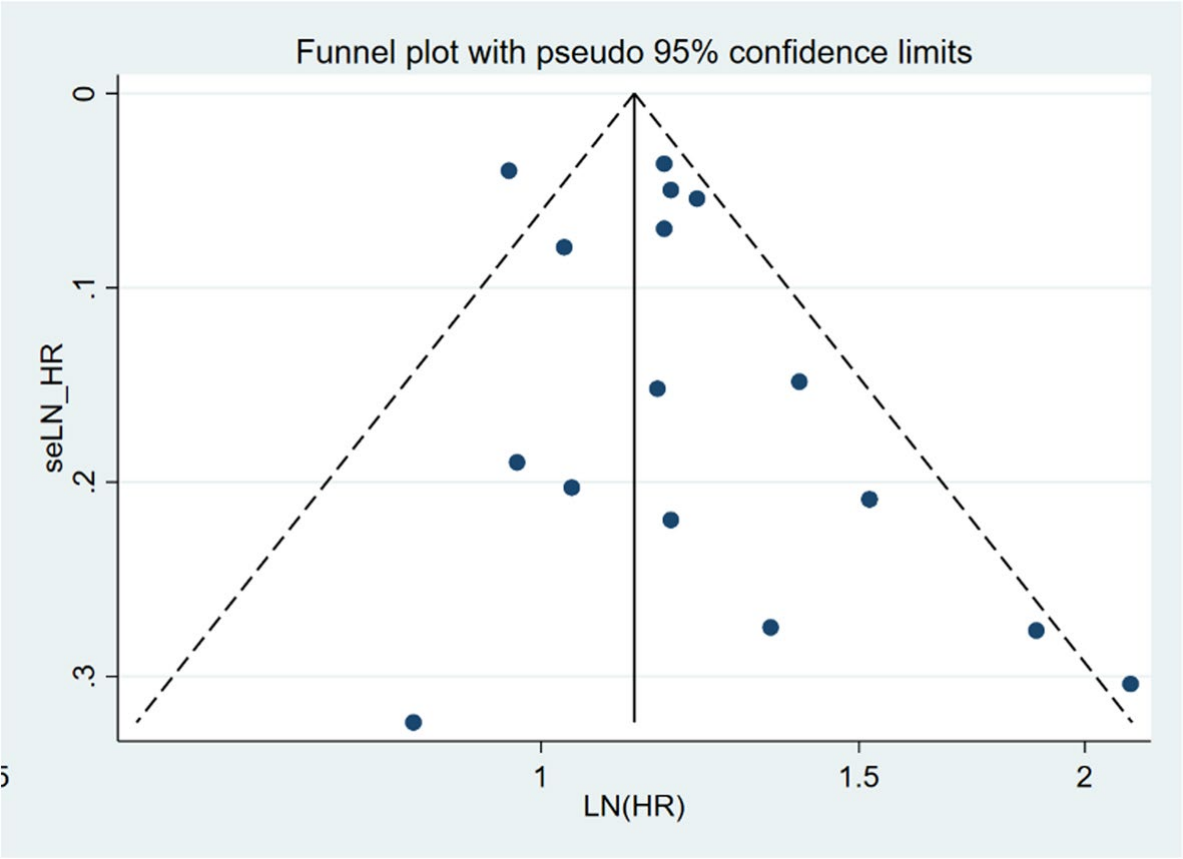
Funnel figure showing the effect of sleep duration on lung cancer

The funnel plot for sleep duration and insomnia symptoms was relatively symmetrical, indicating no publication bias. Egger’s test (*P* = 0.13) revealed no publication bias for sleep duration (Fig. 7).

## Discussion

### Main finding

After conducting a meta-analysis of studies involving 469,691 individuals, we determined that common sleep disturbances, namely extreme sleep duration, symptoms of insomnia, and an evening chronotype, were associated with an increased risk of lung cancer. However, no analogous relationship was observed between morning chronotype or S-SDB and lung cancer risk. Shorter sleep duration was linked to a 13% higher risk of lung cancer than sleep for 7–8 h. Conversely, individuals who slept for longer periods had a surprising 22% increase in lung cancer risk. Meanwhile, those with insomnia symptoms or an evening chronotype exhibited a 1.11–1.15 times higher risk of lung cancer than individuals without sleep disturbances. Overall, our findings suggest that extreme sleep duration, insomnia symptoms, and evening chronotype are significant risk factors for lung cancer.

### Interpretation of findings

Mounting evidence suggests plausible links between abnormal sleep characteristics and various cancers, including skin, ovarian, and colorectal cancer [6, 27, 28]. However, to the best of our knowledge, this systematic review is the first to observe positive associations between multiple sleep disturbances and the incidence of lung cancer, despite no significant increase in the risk of lung cancer reported in previous meta-analyses. As demonstrated by Chen [28], there were no significant association between extreme sleep duration and lung cancer (short: RR = 1.04, 95% CI = 0.88–1.22, *I*^2^ = 46.1%; long: RR = 1.01, 95% CI = 0.83–1.23, *I*^2^ = 41.6%). It is worth noting that Chen's investigation encompassed a limited scope, considering only four studies. To enhance the comprehensiveness and accuracy of our research findings, we expanded our analysis to include six studies conducted between 2018 and 2022. These studies exhibited significant improvements in various aspects, such as sample size, duration of follow-up, and acquisition of sleep-related characteristics. Consequently, our research outcomes were characterized by enhanced precision and a more comprehensive evaluation. Moreover, while Chen's study only focused on sleep duration as a singular characteristic, our research considered multiple dimensions, including insomnia symptom, chronotype and sleep-disordered breathing, thereby providing a multifaceted evaluation of the relationship between sleep and lung cancer. Remarkably, our investigation revealed a positive correlation, further substantiating our conclusions regarding this positive association. Various biologically plausible mechanisms have been proposed to account for the association between sleep disorders and cancer, including impaired antitumor immunity, initiation of inflammation, melatonin decline, and disruption of circadian rhythms.

Sleep quality has been shown to be directly correlated with impaired anti-tumor immunity. Previous animal studies have indicated that sleep deprivation detrimentally affects the number of cytotoxic cells, thereby decreasing the ability of the immune system to respond to tumor growth [29]. Sleep deprivation promotes inflammation, which can initiate the development of lung cancer [30]. This is due to the activation of proinflammatory cytokine secretion, which induces an inflammatory microenvironment in both animal models and humans [31, 32]. The relationship between lung cancer and sleep disturbances has its own particularity, which is mainly mediated by melatonin secretion and the immune-inflammatory balance, which influences the occurrence and development of lung cancer. First, environmental lighting conditions may influence the secretion of melatonin, which can inhibit lung cancer cell migration and proliferation by decreasing TOX3 expression through the direct activation of miR-135b-3p [33]. Melatonin is a promising molecule and is considered a super adjuvant therapy for lung cancer. Adjuvant melatonin following curative intent resection has been reported to increase disease-free survival in patients with late-stage non-small cell lung cancer [10]. Second, the immune-inflammatory balance plays a crucial role in lung cancer risk. Short sleep durations activate the sympathetic effector pathway, which converts the basal gene expression profile into an increased proinflammatory state.

These theories explain why individuals who experience short sleep durations and insomnia symptoms are at a higher risk of developing lung cancer. Interestingly, our findings suggest that long sleep duration is associated with an increased risk of lung cancer, despite prior studies demonstrating its adverse effects on cardiovascular function, neurocognition, and metabolite level [34–36]. However, further experimental exploration is required to uncover the possible pathophysiological mechanisms through which long sleep duration influences lung cancer risk.

Notably, our study showed that individuals with the evening chronotype were at a higher risk of developing lung cancer, whereas no significant association was found in those with the morning chronotype. One explanation for this association is that the extra burden of delayed circadian rhythms caused by the evening chronotype may further exacerbate tumor cell autonomic loss of circadian regulation, leading to the desynchronization of circadian control of cellular metabolism [37–39].

### Implications and limitations

To our knowledge, this meta-analysis is the first to present a comprehensive synthesis of observational studies on multiple sleep characteristics and lung cancer risk. Notably, we excluded the pooled data of cases diagnosed within 2 years of baseline, which complemented the more precise estimates. Moreover, we performed a sensitivity analysis to ensure the stability of the results and enhance the validity of our findings. Our study demonstrated that sleep disturbances should be considered as an independent risk factor for the onset of lung cancer and that appropriate management of sleep disturbances may decrease the risk of lung cancer.

Some limitations of our study should be considered. First, most included studies relied on self-reported questionnaires to identify data on sleep characteristics, which may have introduced a potential bias. Additionally, the adjusted confounders varied among the included studies, which increased the risk of information bias and heterogeneity. Considering that the majority of individuals with lung cancer are often seniors and that a significant correlation exists between sleep parameters and certain occupations, such as nurses and flight attendants, performing subgroup analysis based on age and certain occupations holds significant potential for exploration. Unfortunately, due to the lack of stratified data in the original studies, we were unable to conduct a subgroup analysis of significant risk factors for lung cancer, such as age, occupation, cumulative effect of sleep disturbances, smoking status, and family history of cancer, highlighting the need for further research examining the relationship between sleep traits and other risk factors for lung cancer.

## Conclusion

Our study showed that extreme sleep duration, insomnia symptoms, and evening chronotype were positively associated with the risk of lung cancer. This result suggests that early detection and management of sleep disturbances could be a promising means of mitigating the burden of lung cancer.

### Supplementary Information

**Additional file 1: Table S1. **PubMed (Mar 05, 2023).** Table S2.** Cochrane Library (Mar 05, 2023).** Table S3. **Embase(Mar 05, 2023).** Table S4. **Web of science(Mar 05, 2023).

**Additional file 2: Table S5.** The quality assessment of cohort and case-control studies.

**Additional file 3: Table S6.** Details of the Excluded Literature.

**Additional file 4.** Supplementary Material Lists of excluded literature.

## Data Availability

All the research data and materials in this study were obtained from publicly available sources. The following sources were used: PubMed, EMBASE, Cochrane Library, and Web of Science.

## Abbreviation

HR: Hazard ratio
CI: Confidence interval
PRISMA: Preferred Reporting Items for Systematic Reviews and Mata-Analyses
OR: Odds ratio
RR: Risk ratio
IS: Insomnia symptoms
S-SDB: Subjective sleep disordered breathing
ICD: International Classification of Diseases,10th revision
BMI: Body mass index
