# Supplementary material for: Sleep disturbances and the risk of lung cancer: a meta-epidemiological study
Source: BMC Cancer. 2023 Sep 19;23:884. doi: 10.1186/s12885-023-11392-2 (PMC10510222; doi:10.1186/s12885-023-11392-2)
Supplement: Supplementary file 1 — Additional file 1: Table S1. PubMed (Mar 05, 2023). Table S2. Cochrane Library (Mar 05, 2023). Table S3. Embase(Mar 05, 2023). Table S4. Web of science(Mar 05, 2023). [file 12885_2023_11392_MOESM1_ESM.docx]

**Supplementary Material**

**Table S1~S4: Details of the Literature Search Strategy**

**Table S1:**PubMed (Mar 05, 2023)

| **Search** | **Query** | **Items found** |
| --- | --- | --- |
| #1 | "Sleep"[Mesh] | 95,836 |
| #2 | ((((((Sleep* Habit*[Title/Abstract])) OR (Nap*[Title/Abstract])) OR (Daytime sleep[Title/Abstract])) OR (Siesta[Title/Abstract])) OR (Daytime sleepiness[Title/Abstract])) OR (Daytime somnolence[Title/Abstract]) | 17,845 |
| #3 | #1OR#2 | 107,825 |
| #4 | ("Sleep Initiation and Maintenance Disorders"[Mesh]) OR "Sleep Wake Disorders"[Mesh] | 107,014 |
| #5 | ((Insomnia*[Title/Abstract]) OR ("Disorders of Initiating and Maintaining Sleep"[Title/Abstract])) OR (Early Awakening[Title/Abstract]) | 28,780 |
| #6 | #4 OR #5 | 120,465 |
| #7 | "Lung Neoplasms"[Mesh] | 270,043 |
| #8 | (((Pulmonary Neoplasm*[Title/Abstract]) OR (Lung* Neoplasm*[Title/Abstract])) OR (Lung Cancer*[Title/Abstract])) OR (Pulmonary Cancer*[Title/Abstract]) | 207,721 |
| #9 | #7 OR #8 | 334,225 |
| #10 | #3 OR #6 | 187,933 |
| #11 | #9 AND #10 | 414 |

**Table S2:** Cochrane Library (Mar 05, 2023)

| **Search** | **Query** | **Items found** |
| --- | --- | --- |
| #1 | MeSH descriptor: [Sleep] explode all trees | 7,324 |
| #2 | ("Sleep* Habit*"):ti,ab,kw OR (Nap*):ti,ab,kw OR ("Daytime sleep*"):ti,ab,kw OR (Siesta):ti,ab,kw OR ("Daytime somnolence"):ti,ab,kw | 9,010 |
| #3 | #1 OR #2 | 15,819 |
| #4 | MeSH descriptor: [Sleep Initiation and Maintenance Disorders] explode all trees | 3,200 |
| #5 | MeSH descriptor: [Sleep Wake Disorders] explode all trees | 10,476 |
| #6 | ("Insomnia*"):ti,ab,kw OR ("Disorders of Initiating and Maintaining Sleep"):ti,ab,kw OR ("Early Awakening"):ti,ab,kw | 13,205 |
| #7 | #4 OR #5 OR #6 | 20,701 |
| #8 | MeSH descriptor: [Lung Neoplasms] explode all trees | 10,285 |
| #9 | ("Pulmonary Neoplasm*"):ti,ab,kw OR ("Lung* Neoplasm*"):ti,ab,kw OR ("Lung Cancer*"):ti,ab,kw OR ("Pulmonary Cancer*"):ti,ab,kw | 24,912 |
| #10 | #8 OR #9 | 25,267 |
| #11 | (#3 OR #7) AND #10 | 215 |

**Table S3:** Embase(Mar 05, 2023)

| **Search** | **Query** | **Items found** |
| --- | --- | --- |
| #1 | 'sleep'/exp | 291,158 |
| #2 | 'sleep* habit*':ti,ab,kw OR nap*:ti,ab,kw OR 'daytime sleep*':ti,ab,kw OR siesta:ti,ab,kw OR 'daytime somnolence':ti,ab,kw | 132,428 |
| #3 | #1 OR #2 | 400,149 |
| #4 | 'insomnia'/exp | 84,280 |
| #5 | 'sleep initiation':ti,ab,kw AND 'maintenance disorders':ti,ab,kw OR 'sleep wake disorders':ti,ab,kw OR 'insomnia*':ti,ab,kw OR ('disorders of initiating':ti,ab,kw AND 'maintaining sleep':ti,ab,kw) OR 'early awakening':ti,ab,kw | 49,281 |
| #6 | #4 OR #5 | 93,560 |
| #7 | #3 OR #6 | 457,723 |
| #8 | 'lung tumor'/exp | 535,840 |
| #9 | 'pulmonary neoplasm*':ti,ab,kw OR 'lung* neoplasm*':ti,ab,kw OR 'lung cancer*':ti,ab,kw OR 'pulmonary cancer*':ti,ab,kw | 312,690 |
| #10 | #8 OR #9 | 565,880 |
| #11 | #7 AND #10 | 4,529 |

**Table S4:**Web of science(Mar 05, 2023)

| **Search** | **Query** | **Items found** |
| --- | --- | --- |
| #1 | sleep (Topic) or "Sleep* Habit*" (Topic) or Nap* (Topic) or Daytime sleep* (Topic) or Siesta (Topic) or "Daytime somnolence" (Topic) | 527,705 |
| #2 | "Sleep Initiation and Maintenance Disorders" (Topic) or "Sleep Wake Disorders" (Topic) or Insomnia* (Topic) or "Disorders of Initiating and Maintaining Sleep" (Topic) or "Early Awakening" (Topic) | 38,669 |
| #3 | #1 OR #2 | 542,183 |
| #4 | "Lung Neoplasm" (Topic) or "Pulmonary Neoplasm*" (Topic) or "Lung* Neoplasm*" (Topic) or "Lung Cancer*" (Topic) or "Pulmonary Cancer*" (Topic) | 315,010 |
| #5 | #3 AND #4 | 1,749 |
