# Supplementary material for: Sleep disturbances and the risk of lung cancer: a meta-epidemiological study
Source: BMC Cancer. 2023 Sep 19;23:884. doi: 10.1186/s12885-023-11392-2 (PMC10510222; doi:10.1186/s12885-023-11392-2)
Supplement: Supplementary file 2 — Additional file 2: Table S5. The quality assessment of cohort and case-control studies. [file 12885_2023_11392_MOESM2_ESM.docx]

**Table S5. The quality assessment of cohort and case-control studies.**

| Study | Year | Selection | Comparability | Outcome | Total |
| --- | --- | --- | --- | --- | --- |
| Cohort studies (n=8 ) | | | | | |
| Owais KHAWAJA | 2014 | ** | ** | *** | 7 |
| Maria K Luojus | 2014 | *** | ** | *** | 8 |
| Susan Hurley | 2016 | ** | ** | ** | 6 |
| McNeil J | 2019 | *** | ** | ** | 7 |
| Junxing Xie | 2021 | **** | ** | ** | 8 |
| Q. CAO | 2022 | *** | ** | ** | 7 |
| Noah C | 2022 | *** | ** | *** | 8 |
| Arthur Sillah | 2022 | ** | ** | ** | 6 |
|  |  |  |  |  |  |
|  |  |  |  |  |  |
|  |  |  |  |  |  |
|  |  |  |  |  |  |
|  |  |  |  |  |  |
|  |  |  |  |  |  |
| Case-control studies (n=2) | | | | | |
| Emilie Cordina-Duverger | 2022 | *** | * | ** | 6 |
| Rachel A. Murphy | 2022 | *** | ** | ** | 7 |
|  |  |  |  |  |  |
|  |  |  |  |  |  |
|  |  |  |  |  |  |
|  |  |  |  |  |  |
|  |  |  |  |  |  |

The NOS scale was used to evaluate the quality of the cohort and case-control stud
