# Supplementary material for: Sleep disturbances and the risk of lung cancer: a meta-epidemiological study
Source: BMC Cancer. 2023 Sep 19;23:884. doi: 10.1186/s12885-023-11392-2 (PMC10510222; doi:10.1186/s12885-023-11392-2)
Supplement: Supplementary file 3 — Additional file 3: Table S6. Details of the Excluded Literature. [file 12885_2023_11392_MOESM3_ESM.docx]

**Supplementary Material 3**

**Table S6: Details of the Excluded Literature**

| **The title of excluded literature after reading full-text** | **The reasons for excluding** |
| --- | --- |
| Does obstructive sleep apnea confer risk to induce or enhance tumor malignancy? | review |
| Sleep and cancer: Synthesis of experimental data and meta-analyses of cancer incidence among some 1 500 000 study individuals in 13 countries | review |
| Sleep-disordered breathing and risk of lung cancer: a meta-analysis longitudinal follow-up studies | review |
| Sleep-disordered breathing, hypoxemia and the association with lung cancer | review |
| Key Common Genes in Obstructive Sleep Apnea and Lung Cancer are Associated with Prognosis of Lung Cancer Patients | Genetic studies |
| Genetic Liability to Insomnia and Lung Cancer Risk: A Mendelian Randomization Analysis | Genetic studies |
| Genetically predicted insomnia and lung cancer risk: a Mendelian randomization study | Genetic studies |
| A Sleeping Beauty forward genetic screen identifies novel cancer drivers that cooperate with Pten in lung cancer | Genetic studies |
| Sleep and physical activity in relation to all- cause, cardiovascular disease and cancer mortality risk | No subgroup for lung cancer |
| Prevalence of healthy behaviors among cancer survivors in the United States: How far have we come? | No subgroup for lung cancer |
| Obstructive sleep apnea and the prevalence and incidence  of cancer | No subgroup for lung cancer |
| Obstructive sleep apnea and nocturnal hypoxemia are associated with an increased risk of lung cancer | No sleep disorder data |
| Obstructive Sleep Apnea and Incident Cancer: A Large Retrospective Multicenter Clinical Cohort Study | No sleep disorder data |
| Association Between Nocturnal Hypoxemia and Cancer Incidence in Patients Investigated for OSA | No sleep disorder data |
| Pre-diagnostic Sleep Duration and Sleep Quality in Relation to Subsequent Cancer Survival | No sleep disorder data |
| The risk of cancer among patients with sleep disturbance: a nationwide retrospective study in Taiwan | No sleep disorder data |
| Association between working hours and cancer risk in Japan: The Japan public health center- based prospective study | No sleep disorder data |
| Association between Sedative-hypnotics and Subsequent Cancer in Patients with and without Insomnia: A 14-year Follow-up Study in Taiwan | No sleep disorder data |
| The Incidence of Cancer Is Increased in Hospitalized Adult Patients With Obstructive Sleep Apnea in China: A Retrospective Cohort Study | No available risk estimate |
| Effect of sleep disorders on the risks of cancers and site-specific cancers | No available risk estimate |
| Sleep Duration Across the Adult Lifecourse and Risk of Lung Cancer Mortality: A Cohort Study in Xuanwei, China | No available risk estimate |
| Total and Cause-Specific Mortality of U.S. Nurses Working Rotating Night Shifts | No available risk estimate |
