# Supplementary material for: Sleep disturbances and the risk of lung cancer: a meta-epidemiological study
Source: BMC Cancer. 2023 Sep 19;23:884. doi: 10.1186/s12885-023-11392-2 (PMC10510222; doi:10.1186/s12885-023-11392-2)
Supplement: Supplementary file 4 — Additional file 4. Supplementary Material Lists of excluded literature. [file 12885_2023_11392_MOESM4_ESM.docx]

**Supplementary Material**

**Lists of excluded literature**

This list consists of six parts, including review(n=384)、case or genetic, animal and cell studies(n=571)、non-sleep disorder data(n=2749)、non-lung cancer(n=878)、non-available risk estimate(n=1436)

**Part Ⅰ: Duplicated literature(n=879)**

[1] Chen J B, Kong X F, Lv Y Y, et al. "Real world survey" of hydrogen-controlled cancer: a follow-up report of 82 advanced cancer patients[J]. MEDICAL GAS RESEARCH, 2019,9(3):115-121.

[2] Car J, Zycińska J, Lasota W. [Assessment of psychological distress and depression in cancer patients].[J]. Przegla̧d epidemiologiczny, 2012,66(4):689-695.

[3] Yamanaka S, Sakamoto A, Tomoyasu H. [Synchronous multiple primary lung and colon cancers].[J]. Kyobu geka. The Japanese journal of thoracic surgery, 2013,66(10):882-885.

[4] Kanno T, Tanaka A, Shimizu T, et al. 1-[2-(2-methoxyphenylamino)ethylamino]-3-(naphthalene-1-yloxy)propan-2-ol as a potential anticancer drug[J]. Pharmacology, 2013,91(5-6):339-345.

[5] Kanno T, Tanaka A, Shimizu T, et al. 1-[2-(2-Methoxyphenylamino)ethylamino]-3-(naphthalene-1-yloxy)propan-2-ol as a Potential Anticancer Drug[J]. PHARMACOLOGY, 2013,91(5-6):339-345.

[6] Kanno T, Tanaka A, Shimizu T, et al. 1-[2-(2-Methoxyphenylamino)ethylamino]-3-(naphthalene-1-yloxy)propan-2-ol as a Potential Anticancer Drug[J]. Pharmacology, 2013,91(5-6):339-345.

[7] Nishizaki T, Kanno T, Tsuchiya A, et al. 1-[2-(2-Methoxyphenylamino)ethylamino]-3-(naphthalene-1-yloxy)propan-2-ol May Be a Promising Anticancer Drug[J]. MOLECULES, 2014,19(12):21462-21472.

[8] Roeland E J, Yennu S, Del Fabbro E, et al. 1696TiP Phase Ib study to assess the effect of PF-06946860 on appetite following subcutaneous administration in patients with anorexia and advanced cancer[J]. Annals of Oncology, 2021,32:S1185.

[9] Ong J, Yong P, Lim Y M, et al. 2-Methoxy-1,4-naphthoquinone (MNQ) induces apoptosis of A549 lung adenocarcinoma cells via oxidation-triggered JNK and p38 MAPK signaling pathways[J]. LIFE SCIENCES, 2015,135:158-164.

[10] Sun W, Bao J L, Lin W, et al. 2-Methoxy-6-acetyl-7-methyljuglone (MAM), a natural naphthoquinone, induces NO-dependent apoptosis and necroptosis by H2O2-dependent JNK activation in cancer cells[J]. FREE RADICAL BIOLOGY AND MEDICINE, 2016,92:61-77.

[11] Penthala N R, Ponugoti P R, Kasam V, et al. 5-((1-Aroyl-1H-indol-3-yl)methylene)-2-thioxodihydropyrimidine-4, 6(1H,5H)-diones as potential anticancer agents with anti-inflammatory properties[J]. BIOORGANIC & MEDICINAL CHEMISTRY LETTERS, 2013,23(5):1442-1446.

[12] Celik Y. 6-(1-Oxobutyl)-5,8-dimethoxy-1,4-naphthoquinone inhibits lewis lung cancer by antiangiogenesis and apoptosis[J]. INTERNATIONAL JOURNAL OF CANCER, 2008,122(10):2403.

[13] Lee H J, Lee H J, Song G Y, et al. 6-(1-Oxobutyl)-5,8-dimethoxy-1,4-naphthoquinone inhibits Lewis lung cancer by antiangiogenesis and apoptosis[J]. INTERNATIONAL JOURNAL OF CANCER, 2007,120(11):2481-2490.

[14] Lu T, Denehy L, Cao Y J, et al. A 12-Week Multi-Modal Exercise Program: Feasibility of Combined Exercise and Simplified 8-Style Tai Chi Following Lung Cancer Surgery[J]. INTEGRATIVE CANCER THERAPIES, 2020,19.

[15] Tacha D, Yu C, Bremer R, et al. A 6-antibody panel for the classification of lung adenocarcinoma versus squamous cell carcinoma[J]. Applied Immunohistochemistry and Molecular Morphology, 2012,20(3):201-207.

[16] Hartland M C, Davison K, Nelson M J, et al. A Case Study of Exercise Adherence during Stereotactic Ablative Radiotherapy Treatment in a Previously Active Male with Metastatic Renal Cell Carcinoma[J]. JOURNAL OF SPORTS SCIENCE AND MEDICINE, 2019,18(3):462-470.

[17] Long N H, Thanasilp S, Thato R. A causal model for fatigue in lung cancer patients receiving chemotherapy[J]. European journal of oncology nursing : the official journal of European Oncology Nursing Society, 2016,21:242-247.

[18] Long N H, Thanasilp S, Thato R. A causal model for fatigue in lung cancer patients receiving chemotherapy[J]. EUROPEAN JOURNAL OF ONCOLOGY NURSING, 2016,21:242-247.

[19] Zhang Q, Huang X E, Gao L L. A clinical study on the premedication of paclitaxel liposome in the treatment of solid tumors[J]. BIOMEDICINE & PHARMACOTHERAPY, 2009,63(8):603-607.

[20] Liang J, Wang Y, Zheng L, et al. A Clinical Study on the Use of Yiqi Yangxue Decoction Combined with Chemotherapy to Promote Rapid Postoperative Recovery in Patients with Non-Small Cell Lung Cancer[J]. Emergency Medicine International, 2022,2022.

[21] Liang J Y, Wang Y, Zheng L, et al. A Clinical Study on the Use of Yiqi Yangxue Decoction Combined with Chemotherapy to Promote Rapid Postoperative Recovery in Patients with Non-Small Cell Lung Cancer[J]. EMERGENCY MEDICINE INTERNATIONAL, 2022,2022.

[22] Sakamoto T, Furukawa T, Pham H, et al. A collaborative workflow between pathologists and deep learning for the evaluation of tumour cellularity in lung adenocarcinoma[J]. HISTOPATHOLOGY, 2022,81(6):758-769.

[23] Karlsson A, Cirenajwis H, Ericson-Lindquist K, et al. A combined gene expression tool for parallel histological prediction and gene fusion detection in non-small cell lung cancer[J]. SCIENTIFIC REPORTS, 2019,9.

[24] Kavathiya K, Gurjar M, Patil A, et al. A Comparative Pharmacokinetic Study of 2 Pemetrexed Formulations in Indian Adult Chemonaive Patients With Adenocarcinoma Stage III/IV Non–Small Cell Lung Cancer[J]. Clinical Pharmacology in Drug Development, 2017,6(3):234-239.

[25] Lin P P, Chang Y C, Chen C H, et al. A comparative study on the effects of 2,3,7,8,-tetrachlorodibenzo-p-dioxin polychlorinated biphenyl126 and estrogen in human bronchial epithelial cells[J]. TOXICOLOGY AND APPLIED PHARMACOLOGY, 2004,195(1):83-91.

[26] Vanderbyl B L, Mayer M J, Nash C, et al. A comparison of the effects of medical Qigong and standard exercise therapy on symptoms and quality of life in patients with advanced cancer[J]. SUPPORTIVE CARE IN CANCER, 2017,25(6):1749-1758.

[27] Harle A, Molassiotis A, Buffin O, et al. A cross sectional study to determine the prevalence of cough and its impact in patients with lung cancer: a patient unmet need[J]. BMC CANCER, 2020,20(1).

[28] Gu L, Ye X, Xu Y, et al. A double-blind and randomized controlled clinical trial of traditional Chinese medicine combined with adjuvant chemotherapy for early stage non-small cell lung cancer[J]. 2016,28(6):394-398.

[29] Gu L, Ye X, Xu Y, et al. A double-blind and randomized controlled clinical trial of traditional Chinese medicine combined with adjuvant chemotherapy for early stage non-small cell lung cancer[J]. Cancer Research and Clinic, 2016,28(6):394-398.

[30] Hankal C W. A glimpse beyond.[J]. The American journal of nursing, 1993,93(11):96.

[31] Lee M C, Cai H C, Murray C W, et al. A multiplexed in vivo approach to identify driver genes in small cell lung cancer[J]. CELL REPORTS, 2023,42(1).

[32] Nakagawa R, Tateishi H, Radwan M O, et al. A New 1,2-Naphthoquinone Derivative with Anti-lung Cancer Activity[J]. CHEMICAL & PHARMACEUTICAL BULLETIN, 2022,70(7):477-482.

[33] Wani T A, Darwish I A. A novel 96-microwell-based high-throughput spectrophotometric assay for pharmaceutical quality control of crizotinib, a novel potent drug for the treatment of non-small cell lung cancer[J]. BRAZILIAN JOURNAL OF PHARMACEUTICAL SCIENCES, 2015,51(2):439-447.

[34] Viscardi G, Sparano F, Di Liello R, et al. A novel ImmunoScore, based on clinical and blood biomarkers, as prognostic model for immunotherapy in NSCLC[J]. Annals of Oncology, 2019,30:i31.

[35] Viscardi G, Sparano F, Di Liello R, et al. A novel ImmunoScore, based on clinical and blood biomarkers, as prognostic model for immunotherapy in NSCLC[J]. Annals of Oncology, 2019,30:i31.

[36] Haas N S, Shih R, Gochfeld M. A patient with postoperative mercury contamination of the peritoneum[J]. Journal of Toxicology - Clinical Toxicology, 2003,41(2):175-180.

[37] Haas N S, Shih R, Gochfeld M. A patient with postoperative mercury contamination of the peritoneum[J]. JOURNAL OF TOXICOLOGY-CLINICAL TOXICOLOGY, 2003,41(2):175-180.

[38] Kanthala S P, Liu Y Y, Singh S, et al. A peptidomimetic with a chiral switch is an inhibitor of epidermal growth factor receptor heterodimerization[J]. ONCOTARGET, 2017,8(43):74244-74262.

[39] Fujisaka Y, Yamada Y, Yamamoto N, et al. A Phase 1 clinical study of temsirolimus (CCI-779) in Japanese patients with advanced solid tumors[J]. Japanese Journal of Clinical Oncology, 2010,40(8):732-738.

[40] Burris H A, Gordon M S, Gerber D E, et al. A phase I study of DNIB0600A, an antibody-drug conjugate (ADC) targeting NaPi2b, in patients (pts) with non-small cell lung cancer (NSCLC) or platinum-resistant ovarian cancer (OC)[J]. JOURNAL OF CLINICAL ONCOLOGY, 2014,32(15).

[41] Morita S, Minami H, Mitsuma A, et al. A phase I study of LCL161, a novel oral pan-inhibitor of apoptosis protein (IAP) antagonist, in Japanese patients with advanced solid tumors[J]. Asia-Pacific Journal of Clinical Oncology, 2022,18(5):e427-e434.

[42] Gordon M S, Gerber D E, Infante J R, et al. A phase I study of the safety and pharmacokinetics of DNIB0600A, an anti-NaPi2b antibody-drug-conjugate (ADC), in patients (pts) with non- small cell lung cancer (NSCLC) and platinum-resistant ovarian cancer (OC).[J]. JOURNAL OF CLINICAL ONCOLOGY, 2013,31(15).

[43] Grilley-Olson J E, Bedard P L, Fasolo A, et al. A phase Ib dose-escalation study of the MEK inhibitor trametinib in combination with the PI3K/mTOR inhibitor GSK2126458 in patients with advanced solid tumors[J]. 2016,34(6):740-749.

[44] Becerra C, Spira A I, Conkling P R, et al. A phase Ib/II study of cancer sternness inhibitor napabucasin (BB608) combined with weekly paclitaxel in advanced non-small cell lung cancer.[J]. JOURNAL OF CLINICAL ONCOLOGY, 2016,34(15).

[45] Rumble M E, Keefe F J, Edinger J D, et al. A pilot study investigating the utility of the cognitive-behavioral model of insomnia in early-stage lung cancer patients[J]. J Pain Symptom Manage, 2005,30(2):160-169.

[46] Rumble M E, Keefe F J, Edinger J D, et al. A pilot study investigating the utility of the cognitive-behavioral model of insomnia in early-stage lung cancer patients[J]. JOURNAL OF PAIN AND SYMPTOM MANAGEMENT, 2005,30(2):160-169.

[47] Sun C T, Yu H M, Wang X W, et al. A pilot study of extremely low-frequency magnetic fields in advanced non-small cell lung cancer: Effects on survival and palliation of general symptoms[J]. ONCOLOGY LETTERS, 2012,4(5):1130-1134.

[48] Fouladbakhsh J M, Davis J E, Yarandi H N. A pilot study of the feasibility and outcomes of yoga for lung cancer survivors[J]. Oncology Nursing Forum, 2014,41(2):162-174.

[49] Fouladbakhsh J M, Davis J E, Yarandi H N. A Pilot Study of the Feasibility and Outcomes of Yoga for Lung Cancer Survivors[J]. ONCOLOGY NURSING FORUM, 2014,41(2):162-174.

[50] Lemos R, Areias-Marques S, Ferreira P, et al. A prospective observational study for a Federated Artificial Intelligence solution for moniToring mental Health status after cancer treatment (FAITH): study protocol[J]. BMC PSYCHIATRY, 2022,22(1).

[51] McDonnell K K, Gallerani D G, Newsome B R, et al. A Prospective Pilot Study Evaluating Feasibility and Preliminary Effects of Breathe Easier: A Mindfulness-based Intervention for Survivors of Lung Cancer and Their Family Members (Dyads)[J]. INTEGRATIVE CANCER THERAPIES, 2020,19.

[52] Katpattil S. A prospective study on quality of life among persons with lung cancer, before and after chemotherapy treatment-evidence from South India[J]. Annals of Oncology, 2016,27:x159.

[53] Katpattil S. A Prospective study on quality of life among persons with lung cancer, before and after the chemotherapy treatment-evidence from south India[J]. Journal of Thoracic Oncology, 2017,12(1):S1501-S1502.

[54] Tang Y P, Yu F M, Zhang G M, et al. A Purified Serine Protease from Nereis virens and Its Impaction of Apoptosis on Human Lung Cancer Cells[J]. MOLECULES, 2017,22(7).

[55] Li N, Ou W, Yang H, et al. A randomized phase 2 trial of erlotinib versus pemetrexed as second-line therapy in the treatment of patients with advanced EGFR wild-type and EGFR FISH-positive lung adenocarcinoma[J]. Cancer, 2014,120(9):1379-1386.

[56] O'Brien M, Saini A, Smith I E, et al. A randomized phase II study of SRL172 (Mycobacterium vaccae) combined with chemotherapy in patients with advanced inoperable non-small-cell lung cancer and mesothelioma[J]. BRITISH JOURNAL OF CANCER, 2000,83(7):853-857.

[57] Garon E B, Neidhart J D, Gabrail N Y, et al. A randomized phase II trial of the tumor vascular disrupting agent CA4P (fosbretabulin tromethamine) with carboplatin, paclitaxel, and bevacizumab in advanced nonsquamous non-small-cell lung cancer[J]. OncoTargets and Therapy, 2016,9:7275-7283.

[58] Wang Y, Wang H, Jiang Y, et al. A randomized phase III study of combining erlotinib with bevacizumab and panitumumab versus erlotinib alone as second-line therapy for Chinese patients with non-small-cell lung cancer[J]. Biomedicine and Pharmacotherapy, 2017,89:875-879.

[59] Yoh K, Hosomi Y, Kasahara K, et al. A randomized, double-blind, phase II study of ramucirumab plus docetaxel vs placebo plus docetaxel in Japanese patients with stage IV non-small cell lung cancer after disease progression on platinum-based therapy[J]. Lung Cancer, 2016,99:186-193.

[60] Baine M K, Sinard J H, Cai G, et al. A Semiquantitative Scoring System May Allow Biopsy Diagnosis of Pulmonary Large Cell Neuroendocrine Carcinoma[J]. American Journal of Clinical Pathology, 2020,153(2):165-174.

[61] Baine M K, Sinard J H, Cai G P, et al. A Semiquantitative Scoring System May Allow Biopsy Diagnosis of Pulmonary Large Cell Neuroendocrine Carcinoma Experience With Tissue Microarrays[J]. AMERICAN JOURNAL OF CLINICAL PATHOLOGY, 2020,153(2):165-174.

[62] Cairns B J, Travis R C, Wang X S, et al. A short-term increase in cancer risk associated with daytime napping is likely to reflect pre-clinical disease: Prospective cohort study[J]. British Journal of Cancer, 2012,107(3):527-530.

[63] Wu C N, Wu X H, Yu D N, et al. A single-dose of stellate ganglion block for the prevention of postoperative dysrhythmias in patients undergoing thoracoscopic surgery for cancer: A randomised controlled double-blind trial[J]. European Journal of Anaesthesiology, 2020,37(4):323-331.

[64] Wu C N, Wu X H, Yu D N, et al. A single-dose of stellate ganglion block for the prevention of postoperative dysrhythmias in patients undergoing thoracoscopic surgery for cancer: A randomised controlled double-blind trial[J]. European journal of anaesthesiology, 2020,37(4):323-331.

[65] Dorr C, Weg M, Been R, et al. A Sleeping Beauty forward genetic screen identifies novel cancer drivers that cooperate with Pten in lung cancer[J]. CANCER RESEARCH, 2013,73.

[66] Yang M, Nonaka D. A study of immunohistochemical differential expression in pulmonary and mammary carcinomas[J]. MODERN PATHOLOGY, 2010,23(5):654-661.

[67] Subramanian J, Baggstrom M Q, Gilstrap E, et al. A survey on insomnia in patients with non-small cell lung cancer (NSCLC)[J]. JOURNAL OF CLINICAL ONCOLOGY, 2010,28(15).

[68] Brown J K, Cooley M E, Chernecky C, et al. A symptom cluster and sentinel symptom experienced by women with lung cancer[J]. Oncol Nurs Forum, 2011,38(6):E425-E435.

[69] Brown J K, Cooley M E, Chernecky C, et al. A Symptom Cluster and Sentinel Symptom Experienced by Women With Lung Cancer[J]. ONCOLOGY NURSING FORUM, 2011,38(6):E425-E435.

[70] Simonsen A T, Utke A, Lade-Keller J, et al. A targeted expression panel for classification, gene fusion detection and PD-L1 measurements – Can molecular profiling replace immunohistochemistry in non-small cell lung cancer?[J]. Experimental and Molecular Pathology, 2022,125.

[71] Koh J M, Chung D H, Jeon Y K, et al. A useful guideline for determining histological subtypes of non-small cell lung cancers that show equivocal expression of p40 and Napsin A in small biopsies[J]. VIRCHOWS ARCHIV, 2013,463(2):238-239.

[72] Chen Y Y, Yang H, Cheng Z Q, et al. A whole-slide image (WSI)-based immunohistochemical feature prediction system improves the subtyping of lung cancer[J]. LUNG CANCER, 2022,165:18-27.

[73] Zachara-Szczakowski S, Verdun T, Churg A. Accuracy of classifying poorly differentiated non-small cell lung carcinoma biopsies with commonly used lung carcinoma markers[J]. Human Pathology, 2015,46(5):776-782.

[74] Zachara-Szczakowski S, Verdun T, Churg A. Accuracy of classifying poorly differentiated non-small cell lung carcinoma biopsies with commonly used lung carcinoma markers[J]. HUMAN PATHOLOGY, 2015,46(5):776-782.

[75] Du-Quiton J, Wood P A, Burch J B, et al. Actigraphic assessment of daily sleep-activity pattern abnormalities reflects self-assessed depression and anxiety in outpatients with advanced non-small cell lung cancer[J]. PSYCHO-ONCOLOGY, 2010,19(2):180-189.

[76] Du-Quiton J, Wood P A, Burch J B, et al. Actigraphic assessment of daily sleep-activity pattern abnormalities reflects self-assessed depression and anxiety in outpatients with advanced non-small cell lung cancer[J]. Psycho-Oncology, 2010,19(2):180-189.

[77] Lupi I, Brancatella A, Cetani F, et al. Activating Antibodies to the Calcium-sensing Receptor in Immunotherapy-induced Hypoparathyroidism[J]. Journal of Clinical Endocrinology and Metabolism, 2020,105(5).

[78] Zhang X B, Zou C L, Duan Y X, et al. Activity guided isolation and modification of juglone from Juglans regia as potent cytotoxic agent against lung cancer cell lines[J]. BMC COMPLEMENTARY AND ALTERNATIVE MEDICINE, 2015,15.

[79] Xi Z Q, Wei X Q, Ye Z, et al. Acupuncture for adult lung cancer of patient-reported outcomes: A systematic review and meta-analysis[J]. FRONTIERS IN ONCOLOGY, 2022,12.

[80] Siegel P, de Barros N F, Quispe-Cabanillas J G, et al. Acupuncture for cancer patients undergoing chemotherapy in a Brazilian hospital-An exploratory study[J]. European Journal of Integrative Medicine, 2015,7(1):23-28.

[81] Poulsen T T, Naizhen X, Poulsen H S, et al. Acute damage by naphthalene triggers expression of the neuroendocrine marker PGP9.5 in airway epithelial cells[J]. TOXICOLOGY LETTERS, 2008,181(2):67-74.

[82] Warren K N, Katakam J, Espiridion E D. Acute-onset Mania in a Patient with Non-small Cell Lung Cancer[J]. CUREUS, 2019,11(8).

[83] Seely D, Legacy M, Auer R C, et al. Adjuvant melatonin for the prevention of recurrence and mortality following lung cancer resection (AMPLCaRe): A randomized placebo controlled clinical trial[J]. ECLINICALMEDICINE, 2021,33.

[84] Conzo G, Tricarico A, Belli G, et al. Adrenal incidentalomas in the laparoscopic era and the role of correct surgical indications: observations from 255 consecutive adrenalectomies in an Italian series.[J]. Canadian journal of surgery. Journal canadien de chirurgie, 2009,52(6):E281-E285.

[85] Garcia-Sanmartin J, Larrayoz I M, Martinez A. Adrenomedullin regulates club cell recovery following lung epithelial injury[J]. HISTOLOGY AND HISTOPATHOLOGY, 2016,31(6):663-673.

[86] Baker F, Denniston M, Smith T, et al. Adult cancer survivors: How are they faring?[J]. CANCER, 2005,104(11):2565-2576.

[87] Minai-Tehrani A, Park Y C, Hwang S K, et al. Aerosol delivery of kinase-deficient Akt1 attenuates Clara cell injury induced by naphthalene in the lungs of dual luciferase mice[J]. JOURNAL OF VETERINARY SCIENCE, 2011,12(4):309-317.

[88] Cheung W Y, Le L W, Gagliese L, et al. Age and gender differences in symptom intensity and symptom clusters among patients with metastatic cancer[J]. Supportive Care in Cancer, 2011,19(3):417-423.

[89] Dregan A, Armstrong D. Age, cohort and period effects in the prevalence of sleep disturbances among older people: The impact of economic downturn[J]. SOCIAL SCIENCE & MEDICINE, 2009,69(10):1432-1438.

[90] King-Kallimanis B L, Kanapuru B, Blumenthal G M, et al. Age-related differences in patient-reported outcomes in patients with advanced lung cancer receiving anti-PD-1/PD-L1 therapy[J]. Seminars in Oncology, 2018,45(4):201-209.

[91] King-Kallimanis B L, Kanapuru B, Blumenthal G M, et al. Age-related differences in patient-reported outcomes in patients with advanced lung cancer receiving anti-PD-1/PD-L1 therapy[J]. SEMINARS IN ONCOLOGY, 2018,45(4):201-209.

[92] King-Kallimanis B L, Kanapuru B, Blumenthal G M, et al. Age-related differences in patient-reported outcomes in patients with advanced lung cancer receiving anti-PD-1/PD-L1 therapy[J]. 2018,45(4):201-209.

[93] Kriegsmann K, Cremer M, Zgorzelski C, et al. Agreement of CK5/6, p40, and p63 immunoreactivity in non-small cell lung cancer[J]. PATHOLOGY, 2019,51(3):240-245.

[94] Ignatius Ou S H, Sommers K R, Azada M C, et al. Alectinib induces a durable (>15 months) complete response in an ALK-positive non-small cell lung cancer patient who progressed on crizotinibwith diffuse leptomeningeal carcinomatosis[J]. Oncologist, 2015,20(2):224-226.

[95] Xia J, Wu P, Wang J W, et al. Alerting the illusion of smoking improves quality of life in Chinese male cancer survivors[J]. CANCER MEDICINE, 2019,8(3):1066-1073.

[96] Xia J, Wu P, Wang J, et al. Alerting the illusion of smoking improves quality of life in Chinese male cancer survivors[J]. Cancer Medicine, 2019,8(3):1066-1073.

[97] Duan X Q, Zhao X H, Wang S H. An ALK-positive lung adenocarcinoma with gastric and skin metastasis: a case report and literature review[J]. ANNALS OF PALLIATIVE MEDICINE, 2021,10(5):5797-5807.

[98] Jana S, Bhongade R A, Sharma U H S. An Analytical Survey on Cardiovascular Activity Among the Persons Living in a Passive Smoking Environment[J]. Cardiometry, 2022,24:326-332.

[99] Erman M, Seiden D, Zammit G, et al. An efficacy, safety, and dose-response study of Ramelteon in patients with chronic primary insomnia[J]. Sleep Medicine, 2006,7(1):17-24.

[100] Lei J T, Yang J Y, Dong L, et al. An exercise prescription for patients with lung cancer improves the quality of life, depression, and anxiety[J]. FRONTIERS IN PUBLIC HEALTH, 2022,10.

[101] Lei J, Yang J, Dong L, et al. An exercise prescription for patients with lung cancer improves the quality of life, depression, and anxiety[J]. Frontiers in public health, 2022,10:1050471.

[102] Lal M, Raheja S, Kale S, et al. An experience with 156 patients attending a newly organized pain and palliative care clinic in a tertiary hospital[J]. INDIAN JOURNAL OF CANCER, 2012,49(3):293-297.

[103] de Boer R, Humblet Y, Wolf J, et al. An open-label study of vandetanib with pemetrexed in patients with previously treated non-small-cell lung cancer[J]. 2009,20(3):486-491.

[104] Zenali M J, Weissferdt A, Solis L M, et al. An update on clinicopathological, immunohistochemical, and molecular profiles of colloid carcinoma of the lung[J]. Human Pathology, 2015,46(6):836-842.

[105] Guo R X, Wang H. Analysis of Lung Imaging Intelligent Diagnosis System for Nursing Intervention of Lung Cancer Patients' Quality of Life[J]. CONTRAST MEDIA & MOLECULAR IMAGING, 2021,2021.

[106] Thomas J M, Chakraborty B, Sen D, et al. Analyte-Driven Switching of DNA Charge Transport: De Novo Creation of Electronic Sensors for an Early Lung Cancer Biomarker[J]. JOURNAL OF THE AMERICAN CHEMICAL SOCIETY, 2012,134(33):13823-13833.

[107] Li M M, Zha G L, Chen R J, et al. Anticancer effects of myricetin derivatives in non-small cell lung cancer in vitro and in vivo[J]. PHARMACOLOGY RESEARCH & PERSPECTIVES, 2022,10(1).

[108] Bezerra J, Johanes I, Pinheiro A. Anticancer potential and toxicity of the genus Handroanthus Mattos (Bignoniaceae): A systematic review[J]. TOXICON, 2022,217:131-142.

[109] Yamamoto T, Tsuji S. Anti-Ma2-associated encephalitis and paraneoplastic limbic encephalitis[J]. Brain and Nerve, 2010,62(8):838-851.

[110] Eyong K O, Ketsemen H L, Zhao Z Z, et al. Antiproliferative activity of naphthoquinones and indane carboxylic acids from lapachol against a panel of human cancer cell lines[J]. MEDICINAL CHEMISTRY RESEARCH, 2020,29(6):1058-1066.

[111] Kumar S, Tripathi J, Maurya D K, et al. Anti-proliferative effect and underlying mechanism of ethoxy-substituted phylloquinone (vitamin K1 derivative) from Spinacia oleracea leaf and enhancement of its extractability using radiation technology[J]. 3 BIOTECH, 2022,12(10).

[112] ELIASON J F, KAUFMANN F, TANAKA T, et al. ANTIPROLIFERATIVE EFFECTS OF THE AROTINOID RO-40-8757 ON HUMAN CANCER CELL-LINES INVITRO[J]. BRITISH JOURNAL OF CANCER, 1993,67(6):1293-1298.

[113] Ryu H, Choi H K, Kim H J, et al. Antitumor Activity of a Novel Tyrosine Kinase Inhibitor AIU2001 Due to Abrogation of the DNA Damage Repair in Non-Small Cell Lung Cancer Cells[J]. INTERNATIONAL JOURNAL OF MOLECULAR SCIENCES, 2019,20(19).

[114] Wright B D, Deblock M C, Wagers P O, et al. Anti-tumor activity of lipophilic imidazolium salts on select NSCLC cell lines[J]. MEDICINAL CHEMISTRY RESEARCH, 2015,24(7):2838-2861.

[115] Kitagawa R R, Vilegas W, Carlos I Z, et al. Antitumor and immunomodulatory effects of the naphthoquinone 5-methoxy-3,4-dehydroxanthomegnin[J]. REVISTA BRASILEIRA DE FARMACOGNOSIA-BRAZILIAN JOURNAL OF PHARMACOGNOSY, 2011,21(6):1084-1088.

[116] Cai G S, Wang K, Qu N, et al. Antitumor effect of a liposome-encapsulated beta 1,4-galactosyltransferase inhibitor[J]. INTERNATIONAL JOURNAL OF PHARMACEUTICS, 2018,552(1-2):388-393.

[117] Zhang X B, Yang Y Y, Zeng Y, et al. Anti-tumor effect of endostatin in a sleep-apnea mouse model with tumor[J]. CLINICAL & TRANSLATIONAL ONCOLOGY, 2019,21(5):572-581.

[118] Esteves-Souza A, Lucio K A, Da Cunha A S, et al. Antitumoral activity of new polyamine-naphthoquinone conjugates[J]. ONCOLOGY REPORTS, 2008,20(1):225-231.

[119] Misawa T, Mizusawa H. Anti-VGKC antibody-associated limbic encephalitis/morvan syndrome[J]. Brain and Nerve, 2010,62(4):339-345.

[120] Dawson M I, Hobbs P D, Peterson V J, et al. Apoptosis induction in cancer cells by a novel analogue of 6-[3-(1-adamantyl)-4-hydroxyphenyl]-2-naphthalenecarboxylic acid lacking retinoid receptor transcriptional activation activity[J]. CANCER RESEARCH, 2001,61(12):4723-4730.

[121] Shen Y X, Zhou Y, Hou J J, et al. Application effects of evidence-based nursing in pain nursing of advanced lung cancer[J]. AMERICAN JOURNAL OF TRANSLATIONAL RESEARCH, 2021,13(8):9479-9484.

[122] Jagirdar J. Application of immunohistochemistry to the diagnosis of primary and metastatic carcinoma to the lung[J]. ARCHIVES OF PATHOLOGY & LABORATORY MEDICINE, 2008,132(3):384-396.

[123] Gu M, Hua X L, Li S J, et al. Application of Quality Control Circle Activity in Improving Effectiveness of Drug Intervention in Lung Cancer Patients with Moderate to Severe Pain[J]. CURRENT MEDICAL SCIENCE, 2021,41(5):996-1003.

[124] Gu M, Hua X L, Li S J, et al. Application of Quality Control Circle Activity in Improving Effectiveness of Drug Intervention in Lung Cancer Patients with Moderate to Severe Pain[J]. Current medical science, 2021,41(5):996-1003.

[125] Kadivar M, Boozari B. Applications and limitations of immunohistochemical expression of napsin-a in distinguishing lung adenocarcinoma from adenocarcinomas of other organs[J]. Applied Immunohistochemistry and Molecular Morphology, 2013,21(3):191-195.

[126] Baker D. Approvals, submission, and important labeling changes for US marketed pharmaceuticals[J]. Hospital Pharmacy, 2013,48(7):595-602.

[127] Smith J A, Harle A, Dockry R, et al. Aprepitant for Cough in Lung Cancer A Randomized Placebo-controlled Trial and Mechanistic Insights[J]. AMERICAN JOURNAL OF RESPIRATORY AND CRITICAL CARE MEDICINE, 2021,203(6):737-745.

[128] Ueno T, Linder S, Elmberger G. Aspartic proteinase napsin is a useful marker for diagnosis of primary lung adenocarcinoma[J]. BRITISH JOURNAL OF CANCER, 2003,88(8):1229-1233.

[129] Nowak A K, Stockler M R, Byrne M J. Assessing quality of life during chemotherapy for pleural mesothelioma: Feasibility, validity, and results of using the European organization for research and treatment of cancer core quality of life questionnaire and lung cancer module[J]. JOURNAL OF CLINICAL ONCOLOGY, 2004,22(15):3172-3180.

[130] Chung F, Barnes N, Allen M, et al. Assessing the burden of respiratory disease in the UK[J]. RESPIRATORY MEDICINE, 2002,96(12):963-975.

[131] Kramer A J, Rattanavaraha W, Zhang Z F, et al. Assessing the oxidative potential of isoprene-derived epoxides and secondary organic aerosol[J]. ATMOSPHERIC ENVIRONMENT, 2016,130:211-218.

[132] Jasinska M, Tracz M, Kurczewska U, et al. Assessment of change of quality of life in hospitalized terminally ill cancer patients[J]. WSPOLCZESNA ONKOLOGIA-CONTEMPORARY ONCOLOGY, 2010,14(5):333-339.

[133] Barlesi F, Garon E, Kim D W, et al. Assessment of health-related quality of life (HRQoL) in KEYNOTE-010: A phase 2/3 study of pembrolizumab vs docetaxel in patients with previously treated advanced NSCLC[J]. Annals of Oncology, 2016,27.

[134] Mjid M, Slim A, Hrizi D, et al. Assessment of insomnia among Tunisian patients with lung cancer[J]. REVUE DES MALADIES RESPIRATOIRES, 2018,35(7):716-722.

[135] Bulbul Y, Ozlu T, Arinc S, et al. Assessment of Palliative Care in Lung Cancer in Turkey[J]. MEDICAL PRINCIPLES AND PRACTICE, 2017,26(1):50-56.

[136] Snene H, Brahim D, Kacem M, et al. Assessment of sleep disorders among patients with lung cancer[J]. EUROPEAN RESPIRATORY JOURNAL, 2019,54.

[137] Nishiura M, Tamura A, Nagai H, et al. Assessment of sleep disturbance in lung cancer patients: relationship between sleep disturbance and pain, fatigue, quality of life, and psychological distress[J]. Palliative & supportive care, 2015,13(3):575-581.

[138] Nishiura M, Tamura A, Nagai H, et al. Assessment of sleep disturbance in lung cancer patients: Relationship between sleep disturbance and pain, fatigue, quality of life, and psychological distress[J]. PALLIATIVE & SUPPORTIVE CARE, 2015,13(3):575-581.

[139] Silberfarb P M, Hauri P J, Oxman T E, et al. Assessment of sleep in patients with lung cancer and breast cancer[J]. Journal of Clinical Oncology, 1993,11(5):997-1004.

[140] Silberfarb P M, Hauri P J, Oxman T E, et al. Assessment of sleep in patients with lung cancer and breast cancer[J]. J Clin Oncol, 1993,11(5):997-1004.

[141] Fennell D A, Myrand S P, Nguyen T S, et al. Association between Gene Expression Profiles and Clinical Outcome of Pemetrexed-Based Treatment in Patients with Advanced Non-Squamous Non-Small Cell Lung Cancer: Exploratory Results from a Phase II Study[J]. PLOS ONE, 2014,9(9).

[142] Wei P J, Tsai M J, Tsai Y H, et al. Association between obstructive sleep apnea (OSA) and cancer incidence-a nationwide population-based study[J]. European Respiratory Journal, 2013,42.

[143] Zhuang J M, Liu Y H, Xu X Y, et al. Association between physical activity and health-related quality of life: time to deterioration model analysis in lung adenocarcinoma[J]. JOURNAL OF CANCER SURVIVORSHIP.

[144] Wang J, Tang H, Duan Y, et al. Association between Sleep Traits and Lung Cancer: a Mendelian Randomization Study[J]. 2021,2021:1893882.

[145] Wang J, Tang H B, Duan Y M, et al. Association between Sleep Traits and Lung Cancer: A Mendelian Randomization Study[J]. JOURNAL OF IMMUNOLOGY RESEARCH, 2021,2021.

[146] Wang J, Tang H, Duan Y, et al. Association between Sleep Traits and Lung Cancer: A Mendelian Randomization Study[J]. Journal of Immunology Research, 2021,2021.

[147] Lee H, Kim H H, Kim K Y, et al. Associations among sleep-disordered breathing, sleep quality, and lung cancer in Korean patients[J]. SLEEP AND BREATHING.

[148] Lee H, Kim H H, Kim K Y, et al. Associations among sleep-disordered breathing, sleep quality, and lung cancer in Korean patients[J]. Sleep and Breathing, 2022.

[149] Choi A K, Williamson T J, Kim J C, et al. ASSOCIATIONS BETWEEN DISEASE-SPECIFIC COPING STRATEGIES AND SLEEP OUTCOMES AMONG LUNG CANCER PATIENTS[J]. PSYCHOSOMATIC MEDICINE, 2019,81(4):A116.

[150] Zhou S, Zhu Q Q, Liu H M, et al. Associations of polycyclic aromatic hydrocarbons exposure and its interaction with XRCC1 genetic polymorphism with lung cancer: A case-control study[J]. ENVIRONMENTAL POLLUTION, 2021,290.

[151] Zhou S, Zhu Q, Liu H, et al. Associations of polycyclic aromatic hydrocarbons exposure and its interaction with XRCC1 genetic polymorphism with lung cancer: A case-control study[J]. Environmental Pollution, 2021,290.

[152] Sun S Y, Yue P, Hong W K, et al. Augmentation of tumor necrosis factor-related apoptosis-inducing ligand (TRAIL)-induced apoptosis by the synthetic retinoid 6-[3-(1-adamantyl)-4-hydroxyphenyl]-2-naphthalene carboxylic acid (CD437) through up-regulation of TRAIL receptors in human lung cancer cells[J]. CANCER RESEARCH, 2000,60(24):7149-7155.

[153] Lin L, Zhang Y, Qian H Y, et al. Auricular acupressure for cancer-related fatigue during lung cancer chemotherapy: a randomised trial[J]. BMJ supportive & palliative care, 2021,11(1):32-39.

[154] Lin L, Zhang Y, Qian H Y, et al. Auricular acupressure for cancer-related fatigue during lung cancer chemotherapy: a randomised trial[J]. BMJ SUPPORTIVE & PALLIATIVE CARE, 2021,11(1):32-39.

[155] Tan Q Y, Wang D, Yang J L, et al. Autoantibody profiling identifies predictive biomarkers of response to anti-PD1 therapy in cancer patients[J]. THERANOSTICS, 2020,10(14):6399-6410.

[156] Berner F, Bomze D, Lichtensteiger C, et al. Autoreactive napsin A-specific T cells are enriched in lung tumors and inflammatory lung lesions during immune checkpoint blockade[J]. SCIENCE IMMUNOLOGY, 2022,7(75).

[157] Walker S L, Saltman D L, Colucci R, et al. Awareness of risk factors among persons at risk for lung cancer, chronic obstructive pulmonary disease and sleep apnea: A Canadian population-based study[J]. CANADIAN RESPIRATORY JOURNAL, 2010,17(6):287-294.

[158] Gea J. Basic Research in Pulmonology[J]. ARCHIVOS DE BRONCONEUMOLOGIA, 2008,44(11):621-628.

[159] Kim M J, Shin H C, Shin K C, et al. Best immunohistochemical panel in distinguishing adenocarcinoma from squamous cell carcinoma of lung: Tissue microarray assay in resected lung cancer specimens[J]. Annals of Diagnostic Pathology, 2013,17(1):85-90.

[160] Hoang D H, Song M, Kovale L M, et al. Beta-naphthoflavone and doxorubicin synergistically enhance apoptosis in human lung cancer cells by inducing doxorubicin accumulation, mitochondrial ROS generation, and JNK pathway signaling[J]. BIOCHEMICAL AND BIOPHYSICAL RESEARCH COMMUNICATIONS, 2022,635:37-45.

[161] Perin N, Cindric M, Zlatar I, et al. Biological evaluation of novel bicyclic heteroaromatic benzazole derived acrylonitriles: synthesis, antiproliferative and antibacterial activity[J]. MEDICINAL CHEMISTRY RESEARCH, 2022,31(8):1339-1350.

[162] Van Der Woude C J, Hommes D W. Biologics in Crohn's disease: Searching indicators for outcome[J]. Expert Opinion on Biological Therapy, 2007,7(8):1233-1243.

[163] Porcel J M. Biomarkers in the diagnosis of pleural diseases: a 2018 update[J]. THERAPEUTIC ADVANCES IN RESPIRATORY DISEASE, 2018,12.

[164] Weiss C, Dickerson S, Dubocovich M L, et al. Bright light therapy to reduce fatigue and improve sleep quality in lung cancer survivors[J]. Sleep, 2018,41:A239.

[165] Weiss C, Dickerson S, Dubocovich M L, et al. BRIGHT LIGHT THERAPY TO REDUCE FATIGUE AND IMPROVE SLEEP QUALITY IN LUNG CANCER SURVIVORS.[J]. SLEEP, 2018,41:A239.

[166] Zhang J L, Yang L, Tan Q H. Bukangling combined with chemotherapy improves quality of life in patients with middle-advanced stage non-small cell lung cancer[J]. Journal of Practical Oncology, 2012,27(2):182-184.

[167] Martuzzi M, Mitis F, Bianchi F, et al. Cancer mortality and congenital anomalies in a region of Italy with intense environmental pressure due to waste[J]. OCCUPATIONAL AND ENVIRONMENTAL MEDICINE, 2009,66(11):725-732.

[168] Visovsky C, Schneider S M. Cancer-related fatigue[J]. Online Journal of Issues in Nursing, 2003,8(3):88-112.

[169] Seah G L, Yu J H, Koo B I, et al. Cancer-targeted reactive oxygen species-degradable polymer nanoparticles for near infrared light-induced drug release[J]. JOURNAL OF MATERIALS CHEMISTRY B, 2018,6(46):7737-7749.

[170] de Cerain A L, Marin A, Idoate M A, et al. Carbonyl reductase and NADPH cytochrome P450 reductase activities in human tumoral versus normal tissues[J]. EUROPEAN JOURNAL OF CANCER, 1999,35(2):320-324.

[171] Carter P A. Caregivers' descriptions of sleep changes and depressive symptoms.[J]. Oncology nursing forum, 2002,29(9):1277-1283.

[172] Jassem J, Penrod J R, Goren A, et al. Caring for relatives with lung cancer in Europe: an evaluation of caregivers' experience[J]. QUALITY OF LIFE RESEARCH, 2015,24(12):2843-2852.

[173] Jassem J, Penrod J R, Goren A, et al. Caring for relatives with lung cancer in Europe: an evaluation of caregivers’ experience[J]. Quality of Life Research, 2015,24(12):2843-2852.

[174] Li Q, Zhang X Y, Feng J, et al. Case Report: Next-Generation Sequencing Reveals Tumor Origin in a Female Patient With Brain Metastases[J]. FRONTIERS IN ONCOLOGY, 2021,11.

[175] Qu D, Xu X M, Zhang M, et al. Cbl participates in shikonin-induced apoptosis by negatively regulating phosphoinositide 3-kinase/protein kinase B signaling[J]. MOLECULAR MEDICINE REPORTS, 2015,12(1):1305-1313.

[176] Qu D, Chen Y, Xu X M, et al. Cbl-b-regulated extracellular signal-regulated kinase signaling is involved in the shikonin-induced apoptosis of lung cancer cells in vitro[J]. EXPERIMENTAL AND THERAPEUTIC MEDICINE, 2015,9(4):1265-1270.

[177] BALL J A, WARNER T, REID P, et al. CENTRAL ALVEOLAR HYPOVENTILATION ASSOCIATED WITH PARANEOPLASTIC BRAIN-STEM ENCEPHALITIS AND ANTI-HU ANTIBODIES[J]. JOURNAL OF NEUROLOGY, 1994,241(9):561-566.

[178] DelRosso L, Hoque R. Central apnea at electroencephalographic seizure onset[J]. Sleep Medicine, 2013,14(12):1426-1427.

[179] Chang W P, Lin C C. Changes in the sleep-wake rhythm, sleep quality, mood, and quality of life of patients receiving treatment for lung cancer: A longitudinal study[J]. Chronobiol Int, 2017,34(4):451-461.

[180] Chang W P, Lin C C. Changes in the sleep-wake rhythm, sleep quality, mood, and quality of life of patients receiving treatment for lung cancer: A longitudinal study[J]. CHRONOBIOLOGY INTERNATIONAL, 2017,34(4):451-461.

[181] Chang W P, Lin C C. Changes in the sleep-wake rhythm, sleep quality, mood, and quality of life of patients receiving treatment for lung cancer: A longitudinal study[J]. Chronobiology international, 2017,34(4):451-461.

[182] Lin C C, Chang W P. Changes in the sleep-wake rhythm, sleep quality, mood, and quality of life of patients receiving treatment for lung cancer: A longitudinal study.[J]. JOURNAL OF CLINICAL ONCOLOGY, 2017,35.

[183] Phillips K M, Jim H S, Donovan K A, et al. Characteristics and correlates of sleep disturbances in cancer patients[J]. Supportive Care in Cancer, 2012,20(2):357-365.

[184] Phillips K M, Jim H S, Donovan K A, et al. Characteristics and correlates of sleep disturbances in cancer patients[J]. SUPPORTIVE CARE IN CANCER, 2012,20(2):357-365.

[185] Dat N D, Thuan N T, Hoang N, et al. Characteristics of polycyclic aromatic hydrocarbons in ambient air of a tropical mega-area, Ho Chi Minh City, Vietnam: concentration, distribution, gas/particle partitioning, potential sources and cancer risk assessment[J]. ENVIRONMENTAL SCIENCE AND POLLUTION RESEARCH, 2022,29(29):44054-44066.

[186] Bao P, Xiao K Q, Wang H J, et al. Characterization and Potential Applications of a Selenium Nanoparticle Producing and Nitrate Reducing Bacterium Bacillus oryziterrae sp nov.[J]. SCIENTIFIC REPORTS, 2016,6.

[187] Hu W, Downward G, Wong J, et al. Characterization of outdoor air pollution from solid fuel combustion in Xuanwei and Fuyuan, a rural region of China[J]. SCIENTIFIC REPORTS, 2020,10(1).

[188] Hu W, Downward G, Wong J Y Y, et al. Characterization of outdoor air pollution from solid fuel combustion in Xuanwei and Fuyuan, a rural region of China[J]. Scientific reports, 2020,10(1):11335.

[189] Alzoman N Z, Alshehri J M, Darwish I A, et al. Charge-transfer reaction of 2,3-dichloro-1,4-naphthoquinone with crizotinib: Spectrophotometric study, computational molecular modeling and use in development of microwell assay for crizotinib[J]. SAUDI PHARMACEUTICAL JOURNAL, 2015,23(1):75-84.

[190] Vinothkumar R, Ceasar S A, Divyarupa A. Chemosuppressive effect of plumbagin on human non-small lung cancer cell xenotransplanted zebrafish[J]. INDIAN JOURNAL OF CANCER, 2017,54(1):253-256.

[191] Chou H L, Chao T Y, Chen T C, et al. Chemotherapy agents induce tartrate-resistant acid phosphatase 5a contributing to the symptom distress in lung cancer patients[J]. EUROPEAN JOURNAL OF PHARMACOLOGY, 2019,846:38-48.

[192] Desaulniers G A, Vena C, Hao Z, et al. CHEMOTHERAPY INDUCED PERIPHERAL NEUROPATHY AND SUBJECTIVE SLEEP QUALITY IN NON-SMALL CELL LUNG CANCER[J]. SLEEP, 2010,33:A305-A306.

[193] Roberts E A, Morrison L E, Behman L J, et al. Chromogenic immunohistochemical quadruplex provides accurate diagnostic differentiation of non-small cell lung cancer[J]. ANNALS OF DIAGNOSTIC PATHOLOGY, 2020,45.

[194] Panetta N L, Krachman S, Chatila W M. Chronic obstructive pulmonary disease and its comorbidities[J]. PANMINERVA MEDICA, 2009,51(2):115-123.

[195] De Lorenzo B, Brito R, Leal T P, et al. Chronic Sleep Restriction Impairs the Antitumor Immune Response in Mice[J]. NEUROIMMUNOMODULATION, 2018,25(2):59-67.

[196] Hrushesky W, Grutsch J, Wood P, et al. Circadian Clock Manipulation for Cancer Prevention and Control and the Relief of Cancer Symptoms[J]. INTEGRATIVE CANCER THERAPIES, 2009,8(4):387-397.

[197] Levin R D, Daehler M A, Grutsch J F, et al. Circadian function in patients with advanced non-small-cell lung cancer[J]. British Journal of Cancer, 2005,93(11):1202-1208.

[198] Levin R D, Daehler M A, Grutsch J F, et al. Circadian function in patients with advanced non-small-cell lung cancer[J]. BRITISH JOURNAL OF CANCER, 2005,93(11):1202-1208.

[199] Levin R D, Daehler M A, Grutsch J F, et al. Circadian function in patients with advanced non-small-cell lung cancer[J]. 2005,93(11):1202-1208.

[200] Zhou L, Zhang Z, Nice E, et al. Circadian rhythms and cancers: the intrinsic links and therapeutic potentials[J]. Journal of Hematology and Oncology, 2022,15(1).

[201] Paccagnella A, Favaretto A, Oniga F, et al. Cisplatin versus carboplatin in combination with mitomycin and vinblastine in advanced non small cell lung cancer. A multicenter, randomized phase III trial[J]. LUNG CANCER, 2004,43(1):83-91.

[202] Hirano M, Tanaka S, Asami O. Classification of Polycyclic Aromatic Hydrocarbons Based on Mutagenicity in Lung Tissue Through DNA Microarray[J]. ENVIRONMENTAL TOXICOLOGY, 2013,28(11):652-659.

[203] Dalmau J, Graus F, Villarejo A, et al. Clinical analysis of anti-Ma2-associated encephalitis[J]. Brain, 2004,127(8):1831-1844.

[204] Dalmau J, Graus F, Villarejo A, et al. Clinical analysis of anti-Ma2-associated encephalitis[J]. BRAIN, 2004,127:1831-1844.

[205] Cai G R, Li P W, Jiao L P. Clinical observation of music therapy combined with anti-tumor drugs in treating 116 cases of tumor patients[J]. Zhongguo Zhong xi yi jie he za zhi Zhongguo Zhongxiyi jiehe zazhi = Chinese journal of integrated traditional and Western medicine / Zhongguo Zhong xi yi jie he xue hui, Zhongguo Zhong yi yan jiu yuan zhu ban, 2001,21(12):891-894.

[206] Cai G R, Li P W, Jiao L P. Clinical observation of music therapy combined with anti-tumor drugs in treating 116 cases of tumor patients[J]. 2001,21(12):891-894.

[207] Ren D H, Wu W B, Zhao Q T, et al. Clinical Significance of Preoperative Naples Prognostic Score in Patients With Non-Small Cell Lung Cancer[J]. TECHNOLOGY IN CANCER RESEARCH & TREATMENT, 2022,21.

[208] GOLDTHORPE A M, MILTON R J, MOFFAT R J, et al. CLINICAL TRIAL OF A NEW THEOPHYLLINE PREPARATION[J]. Practitioner, 1964,193:789-792.

[209] Desaulniers G, Higgins M, Vena C. CLINICAL UTILITY OF PULSE OXIMETRY SCREENING FOR SLEEP DISORDERED BREATHING IN PATIENTS WITH NON-SMALL CELL LUNG CANCER[J]. ONCOLOGY NURSING FORUM, 2013,40(6):E446.

[210] Derks J L, Rijnsburger N, Hermans B, et al. Clinical-Pathologic Challenges in the Classification of Pulmonary Neuroendocrine Neoplasms and Targets on the Horizon for Future Clinical Practice[J]. JOURNAL OF THORACIC ONCOLOGY, 2021,16(10):1632-1646.

[211] Inamura K. Clinicopathological Characteristics and Mutations Driving Development of Early Lung Adenocarcinoma: Tumor Initiation and Progression[J]. INTERNATIONAL JOURNAL OF MOLECULAR SCIENCES, 2018,19(4).

[212] Hammer S H, Prall F. Close Relation of Large Cell Carcinoma to Adenocarcinoma by Hierarchical Cluster Analysis: Implications for Histologic Typing of Lung Cancer on Biopsies[J]. APPLIED IMMUNOHISTOCHEMISTRY & MOLECULAR MORPHOLOGY, 2015,23(8):550-557.

[213] Mirabelli D, Cacciatore A M, Ferrante D, et al. Cohort study of workers employed in an Italian tire manufacturing plant, 1962-2004[J]. CANCER CAUSES & CONTROL, 2012,23(12):2023-2029.

[214] Bian T T, Zhao J L, Feng J, et al. Combination of cadherin-17 and SATB homeobox 2 serves as potential optimal makers for the differential diagnosis of pulmonary enteric adenocarcinoma and metastatic colorectal adenocarcinoma[J]. ONCOTARGET, 2017,8(38):63442-63452.

[215] Kriegsmann K, Longuespee R, Hundemer M, et al. Combined Immunohistochemistry after Mass Spectrometry Imaging for Superior Spatial Information[J]. PROTEOMICS CLINICAL APPLICATIONS, 2019,13(1).

[216] Simbolo M, Centonze G, Giudice L, et al. Combined Large Cell Neuroendocrine Carcinomas of the Lung: Integrative Molecular Analysis Identifies Subtypes with Potential Therapeutic Implications[J]. CANCERS, 2022,14(19).

[217] Raghu G, Amatto V C, Behr J, et al. Comorbidities in idiopathic pulmonary fibrosis patients: a systematic literature review[J]. EUROPEAN RESPIRATORY JOURNAL, 2015,46(4):1113-1130.

[218] Nations J A, Nathan S D. Comorbidities of advanced lung disease[J]. Mount Sinai Journal of Medicine, 2009,76(1):53-62.

[219] Zhang C M, Zhang L, Han Y D. Comparative analyses of ultrasound features, clinical indexes, and prognosis in the presence or absence of axillary lymph node metastasis in lung cancer[J]. INTERNATIONAL JOURNAL OF CLINICAL AND EXPERIMENTAL MEDICINE, 2019,12(7):8815-8822.

[220] Jones R A, Franks S E, Moorehead R A. Comparative mRNA and miRNA transcriptome analysis of a mouse model of IGFIR-driven lung cancer[J]. PLOS ONE, 2018,13(11).

[221] Xiu D M, Fung Y L, Lau B, et al. Comparing dyadic cognitive behavioral therapy (CBT) with dyadic integrative body-mind-spirit intervention (I-BMS) for Chinese family caregivers of lung cancer patients: a randomized controlled trial[J]. SUPPORTIVE CARE IN CANCER, 2020,28(3):1523-1533.

[222] Xiu D, Fung Y L, Lau B H, et al. Comparing dyadic cognitive behavioral therapy (CBT) with dyadic integrative body-mind-spirit intervention (I-BMS) for Chinese family caregivers of lung cancer patients: a randomized controlled trial[J]. 2020,28(3):1523-1533.

[223] Xiu D, Fung Y L, Lau B H P, et al. Comparing dyadic cognitive behavioral therapy (CBT) with dyadic integrative body-mind-spirit intervention (I-BMS) for Chinese family caregivers of lung cancer patients: a randomized controlled trial[J]. Supportive Care in Cancer, 2020,28(3):1523-1533.

[224] Eser S, Goksel T, Erbaycu A E, et al. Comparison of generic and lung cancer-specific quality of life instruments for predictive ability of survival in patients with advanced lung cancer[J]. SPRINGERPLUS, 2016,5.

[225] Lee S S, Baek J H, Park S J, et al. Comparison of programmed intermittent epidural bolus injection and continuous epidural injection in controlling nighttime pain and improving sleep quality after thoracotomy[J]. Medicine (United States), 2022,101(45):E31684.

[226] Lee S S, Baek J H, Park S J, et al. Comparison of programmed intermittent epidural bolus injection and continuous epidural injection in controlling nighttime pain and improving sleep quality after thoracotomy[J]. MEDICINE, 2022,101(45).

[227] Nidhal B, Sonia M, Imene B, et al. Comparison of sleep quality before and after chemotherapy in locally advanced non-small-cell lung cancer patients: a prospective study and literature review[J]. EGYPTIAN JOURNAL OF CHEST DISEASES AND TUBERCULOSIS, 2021,70(4):516-525.

[228] Dimitrova M, Ivanov I, Todorova R, et al. Comparison of the activity levels and localization of dipeptidyl peptidase IV in normal and tumor human lung cells[J]. TISSUE & CELL, 2012,44(2):74-79.

[229] Aikawa E, Kawahara A, Hattori S, et al. Comparison of the Expression Levels of Napsin A, Thyroid Transcription Factor-1, and p63 in Nonsmall Cell Lung Cancer Using Cytocentrifuged Bronchial Brushings[J]. CANCER CYTOPATHOLOGY, 2011,119(5):335-345.

[230] de Oliveira P I, Pereira C, Belasco A, et al. Comparison of the quality of life among persons with lung cancer, before and after the chemotherapy treatment[J]. REVISTA LATINO-AMERICANA DE ENFERMAGEM, 2013,21(3):787-794.

[231] de Oliveira P I, Pereira C A, Belasco A G, et al. Comparison of the quality of life among persons with lung cancer, before and after the chemotherapy treatment.[J]. Revista latino-americana de enfermagem, 2013,21(3):787-794.

[232] Xue D, Han S, Jiang S, et al. Comprehensive geriatric assessment and traditional Chinese medicine intervention benefit symptom control in elderly patients with advanced non-small cell lung cancer[J]. Medical Oncology, 2015,32(4):1-7.

[233] Kangara E F, Peega T, Harmse L, et al. Conformational analysis and potential anticancer activity of [Pt( phen)(L-1-kappa S)(2)] studied by single crystal X-ray diffraction and variable temperature H-1 and Pt-195 NMR spectroscopy[J]. NEW JOURNAL OF CHEMISTRY, 2019,43(9):3665-3672.

[234] Yee J, Sadar M D, Sin D D, et al. Connective tissue-activating peptide III: A novel blood biomarker for early lung cancer detection[J]. Journal of Clinical Oncology, 2009,27(17):2787-2792.

[235] Stojsic J, Jovanic I, Markovic J, et al. Contribution of immunohistochemistry in the differential diagnosis of non-small cell lung carcinomas on small biopsy samples[J]. JOURNAL OF BUON, 2013,18(1):176-187.

[236] van de Wiel M, Derijcke S, Galdermans D, et al. Coping Strategy Influences Quality of Life in Patients With Advanced Lung Cancer by Mediating Mood[J]. CLINICAL LUNG CANCER, 2021,22(2):146-152.

[237] Zunic S S, Sekulic S, DjordjevicDenic G V, et al. Correlation analysis of alveolar macrophage cytochemical parameters in smoking and pulmonary oncology[J]. INTERNATIONAL JOURNAL OF BIOLOGICAL MARKERS, 1997,12(2):79-82.

[238] Tangchang W, Kim Y, Oh Y I, et al. Critical diagnostic and cancer stem cell markers in neoplastic cells from canine primary and xenografted pulmonary adenocarcinoma[J]. JOURNAL OF VETERINARY SCIENCE, 2022,23(6).

[239] Baker D. Current FDA-related drug information-new drugs approved by the FDA; New dosage forms and indications approved by the FDA; Agents pending FDA approval; New drug/biologics license applications filed by manufacturer; Significant labeling changes or "dear health professional" letters related to safety[J]. Hospital Pharmacy, 2011,46(5):359-365.

[240] Sun H X, Chung W C, Ryu S H, et al. Cyclic AMP-Responsive Element Binding Protein- and Nuclear Factor-kappa B-Regulated CXC Chemokine Gene Expression in Lung Carcinogenesis[J]. CANCER PREVENTION RESEARCH, 2008,1(5):316-328.

[241] Kim Y J, Kim E A, Chung M L, et al. Cytotoxic Activity and Three-Dimensional Quantitative Structure Activity Relationship of 2-Aryl-1,8-naphthyridin-4-ones[J]. KOREAN JOURNAL OF PHYSIOLOGY & PHARMACOLOGY, 2009,13(6):511-516.

[242] Lee Y J, Cui J, Lee J, et al. Cytotoxic Compounds from Juglans sinensis Dode Display Anti-Proliferative Activity by Inducing Apoptosis in Human Cancer Cells[J]. MOLECULES, 2016,21(1).

[243] Rasol N E, Ahmad F B, Lim X Y, et al. Cytotoxic lactam and naphthoquinone alkaloids from roots of Goniothalamus lanceolatus Miq.[J]. PHYTOCHEMISTRY LETTERS, 2018,24:51-55.

[244] Park H J, Lee H J, Lee E J, et al. Cytotoxicity and DNA topoisomerase inhibitory activity of benz[f]indole-4,9-dione analogs.[J]. Bioscience, biotechnology, and biochemistry, 2003,67(9):1944-1949.

[245] Tinoco A D, Thomas H R, Incarvito C D, et al. Cytotoxicity of a Ti(IV) compound is independent of serum proteins[J]. PROCEEDINGS OF THE NATIONAL ACADEMY OF SCIENCES OF THE UNITED STATES OF AMERICA, 2012,109(13):5016-5021.

[246] Grutsch J, Hrushesky W, Lis C, et al. Daily evening melatonin prolongs survival among patients with advanced non-small-cell lung cancer[J]. BIOLOGICAL RHYTHM RESEARCH, 2022,53(7):1043-1057.

[247] Bird A C, Sanders M D. Defects in supranuclear control of horizontal eye movements.[J]. Transactions of the ophthalmological societies of the United Kingdom, 1970,90:417-432.

[248] Zhang S, Wang Y. Deoxyshikonin inhibits cisplatin resistance of non–small-cell lung cancer cells by repressing Akt-mediated ABCB1 expression and function[J]. Journal of Biochemical and Molecular Toxicology, 2020,34(10).

[249] Kitagawa R, Yasui-Furukori N, Tsushima T, et al. Depression Increases the Length of Hospitalization for Patients Undergoing Thoracic Surgery: A Preliminary Study[J]. PSYCHOSOMATICS, 2011,52(5):428-432.

[250] Evren A E, Yurttas L, Ekselli B, et al. Design and Efficient Synthesis of Novel 4,5-Dimethylthiazole-Hydrazone Derivatives and their Anticancer Activity[J]. LETTERS IN DRUG DESIGN & DISCOVERY, 2021,18(4):372-386.

[251] CHENG C C, DONG Q, LIU D F, et al. DESIGN OF ANTINEOPLASTIC AGENTS ON THE BASIS OF THE 2-PHENYLNAPHTHALENE-TYPE STRUCTURAL PATTERN .2. SYNTHESIS AND BIOLOGICAL-ACTIVITY STUDIES OF BENZO[B]NAPHTHO[2,3-D]FURAN-6,11-DIONE DERIVATIVES[J]. JOURNAL OF MEDICINAL CHEMISTRY, 1993,36(25):4108-4112.

[252] Wang X, Zhang M, Xiong X Q, et al. Design, synthesis and bioactivity of novel naphthalimide-benzotriazole conjugates against A549 cells via targeting BCL2 G-quadruplex and inducing autophagy[J]. LIFE SCIENCES, 2022,302.

[253] Kadela-Tomanek M, Jastrzebska M, Marciniec K, et al. Design, synthesis and biological activity of 1,4-quinone moiety attached to betulin derivatives as potent DT-diaphorase substrate[J]. BIOORGANIC CHEMISTRY, 2021,106.

[254] Peduto A, Pagano B, Petronzi C, et al. Design, synthesis, biophysical and biological studies of trisubstituted naphthalimides as G-quadruplex ligands[J]. BIOORGANIC & MEDICINAL CHEMISTRY, 2011,19(21):6419-6429.

[255] Tiwari M K, Coghi P, Agrawal P, et al. Design, Synthesis, Structure-Activity Relationship and Docking Studies of Novel Functionalized Arylvinyl-1,2,4-Trioxanes as Potent Antiplasmodial as well as Anticancer Agents[J]. CHEMMEDCHEM, 2020,15(13):1216-1228.

[256] Biswas P, Datta H K, Dastidar P. Designing coordination polymers as multi-drug-self-delivery systems for tuberculosis and cancer therapy: in vitro viability and in vivo toxicity assessment[J]. BIOMATERIALS SCIENCE, 2022,10(21):6201-6216.

[257] Hung H Y, Wu L M, Chen K P. Determinants of Quality of Life in Lung Cancer Patients[J]. Journal of nursing scholarship : an official publication of Sigma Theta Tau International Honor Society of Nursing, 2018,50(3):257-264.

[258] Hung H Y, Wu L M, Chen K P. Determinants of Quality of Life in Lung Cancer Patients[J]. JOURNAL OF NURSING SCHOLARSHIP, 2018,50(3):257-264.

[259] Hill T, Conolly R B. Development of a Novel AOP for Cyp2F2-Mediated Lung Cancer in Mice[J]. TOXICOLOGICAL SCIENCES, 2019,172(1):1-10.

[260] de Rooij B H, van den Hurk C, Smaardijk V, et al. Development of an updated, standardized, patient-centered outcome set for lung cancer[J]. LUNG CANCER, 2022,173:5-13.

[261] Ruiz-Banobre J, Perez-Pampin E, Garcia-Gonzalez J, et al. Development of psoriatic arthritis during nivolumab therapy for metastatic non-small cell lung cancer, clinical outcome analysis and review of the literature[J]. LUNG CANCER, 2017,108:217-221.

[262] Ishihara Y, Sakai H, Nukariya N, et al. Development of quality of life (QOL) questionnaire for use of lung cancer patients in palliative therapy - Study of validity and reliability no. 2, the effects of chemotherapeutics in QOL[J]. Japanese Journal of Cancer and Chemotherapy, 1995,22(8):1087-1093.

[263] Shi H X, Du X J, Wu F, et al. Dexmedetomidine improves early postoperative neurocognitive disorder in elderly male patients undergoing thoracoscopic lobectomy[J]. EXPERIMENTAL AND THERAPEUTIC MEDICINE, 2020,20(4):3868-3877.

[264] Pan S L, Zhou Y L, Wang Q S, et al. Discovery and structure-activity relationship studies of 1-aryl-1H-naphtho[2,3-d][1,2,3]triazole-4,9-dione derivatives as potent dual inhibitors of indoleamine 2,3-dioxygenase 1 (IDO1) and trytophan 2,3-dioxygenase (TDO)[J]. EUROPEAN JOURNAL OF MEDICINAL CHEMISTRY, 2020,207.

[265] An X D, Liu H, Xu Z L, et al. Discovery of potent 1H-imidazo[4,5-b]pyridine-based c-Met kinase inhibitors via mechanism-directed structural optimization[J]. Bioorganic and Medicinal Chemistry Letters, 2014.

[266] An X D, Liu H Y, Xu Z L, et al. Discovery of potent 1H-imidazo[4,5-b]pyridine-based c-Met kinase inhibitors via mechanism-directed structural optimization[J]. BIOORGANIC & MEDICINAL CHEMISTRY LETTERS, 2015,25(3):708-716.

[267] Lee J W, Park H S, Ryu S H, et al. Disruption of CREB-CREBBP association by 2-Naphthol AS-E phosphate induces cell cycle arrest and apoptosis in non-small cell lung cancer cells[J]. CANCER RESEARCH, 2013,73(8).

[268] Shinar Y R, Marks A D. Distressing Visions at the End of Life: Case Report and Review of the Literature[J]. The journal of pastoral care & counseling : JPCC, 2015,69(4):251-253.

[269] Lim E S, Rhee Y H, Park M K, et al. DMNQ S-64 induces apoptosis via caspase activation and cyclooxygenase-2 inhibition in human nonsmall lung cancer cells[M]//Diederich M. Cell Signaling World 2006 Conference: 2007:7-18.

[270] Wang S, Liu F, Zhu J, et al. DNA repair genes ERCC1 and BRCA1 expression in non-small cell lung cancer chemotherapy drug resistance[J]. Medical Science Monitor, 2016,22:1999-2005.

[271] Sang C Y, Xu X H, Qin W W, et al. DPMA, a deoxypodophyllotoxin derivative, induces apoptosis and anti-angiogenesis in non-small cell lung cancer A549 cells[J]. BIOORGANIC & MEDICINAL CHEMISTRY LETTERS, 2013,23(24):6650-6655.

[272] Sethi S, Geng L, Shidham V B, et al. Dual color multiplex TTF-1 + Napsin A and p63 + CK5 immunostaining for subcategorizing of poorly differentiated pulmonary non-small carcinomas into adenocarcinoma and squamous cell carcinoma in fine needle aspiration specimens[J]. CytoJournal, 2012,9(1).

[273] Yang J J, Mu X F, Wang Y, et al. Dysbiosis of the Salivary Microbiome Is Associated With Non-smoking Female Lung Cancer and Correlated With lmmunocytochemistry Markers[J]. FRONTIERS IN ONCOLOGY, 2018,8.

[274] Han Y S, Lai Y H, Hung C T, et al. Early Stage Lung Cancer Survivors' Characteristics and Relevant Factors of Sleep Disturbance[J]. PSYCHO-ONCOLOGY, 2013,22:328.

[275] Frank A L, Kreuter M, Schwarzkopf L. Economic burden of incident interstitial lung disease (ILD) and the impact of comorbidity on costs of care[J]. RESPIRATORY MEDICINE, 2019,152:25-31.

[276] Granger C L, Edbrooke L, Antippa P, et al. Effect of a postoperative home-based exercise and self-management programme on physical function in people with lung cancer (CAPACITY): protocol for a randomised controlled trial[J]. 2022,9(1).

[277] Gouez M, Perol O, Perol M, et al. Effect of acute aerobic exercise before immunotherapy and chemotherapy infusion in patients with metastatic non-small-cell lung cancer: protocol for the ERICA feasibility trial[J]. BMJ OPEN, 2022,12(4).

[278] Hu H P, Zhang X, Chen L, et al. Effect of Conventional Nursing Combined with Bedtime Oculomotor Training on Sleep Quality and Body Immunity of Advanced Lung Cancer Patients[J]. JOURNAL OF HEALTHCARE ENGINEERING, 2022,2022.

[279] Hu H, Zhang X, Chen L, et al. Effect of Conventional Nursing Combined with Bedtime Oculomotor Training on Sleep Quality and Body Immunity of Advanced Lung Cancer Patients[J]. Journal of Healthcare Engineering, 2022,2022.

[280] Hu H P, Yang W Y, Liu Z M, et al. Effect of Eye Movement Training on Sleep Quality of Patients with Advanced Lung Cancer Based on Pittsburgh Sleep Quality Index[J]. JOURNAL OF HEALTHCARE ENGINEERING, 2021,2021.

[281] Hu H, Yang W, Liu Z, et al. Effect of Eye Movement Training on Sleep Quality of Patients with Advanced Lung Cancer Based on Pittsburgh Sleep Quality Index[J]. Journal of Healthcare Engineering, 2021,2021.

[282] Fu Y, Lu H L, Wu S M. Effect of integrated nursing care in treatment of lung cancer patients[J]. INTERNATIONAL JOURNAL OF CLINICAL AND EXPERIMENTAL MEDICINE, 2020,13(4):2766-2773.

[283] Pirnia B, Masoudi R, Sefidrood M, et al. Effect of metformin on cigarette withdrawal syndrome and abstinence in lung cancer patients; a double-blind placebo-controlled trial[J]. International Journal of Cancer Management, 2021,14(5).

[284] Tang H K, Wang Y C, Liu J S. EFFECT OF MUSICAL THERAPY SIX STEPS ON PAIN, ANXIETY AND SLEEP QUALITY IN LUNG CANCER PATIENTS UNDERGOING CHEMOTHERAPY[J]. BASIC & CLINICAL PHARMACOLOGY & TOXICOLOGY, 2015,117:8-9.

[285] Gu F, Li X F, Xu J F, et al. Effect of nicotine dependence on quality of life and sleep quality in patients with lung cancer who continue to smoke after diagnosis[J]. JOURNAL OF THORACIC DISEASE, 2018,10(5):2583-2589.

[286] Gu F, Li X F, Xu J F, et al. Effect of nicotine dependence on quality of life and sleep quality in patients with lung cancer who continue to smoke after diagnosis[J]. Journal of Thoracic Disease, 2018,10(5):2583-2589.

[287] Yu J, Huang T, Xu J, et al. Effect of Nursing Method of Psychological Intervention Combined with Health Education on Lung Cancer Patients Undergoing Chemotherapy[J]. JOURNAL OF HEALTHCARE ENGINEERING, 2022,2022.

[288] Kang K, Meng X, Li B, et al. Effect of thoracic paravertebral nerve block on the early postoperative rehabilitation in patients undergoing thoracoscopic radical lung cancer surgery[J]. WORLD JOURNAL OF SURGICAL ONCOLOGY, 2020,18(1).

[289] Wei T, Hou H, Zhou L L, et al. Effect of ultrasound-guided pulsed radiofrequency on intercostal neuralgia after lung cancer surgery A retrospective study[J]. MEDICINE, 2021,100(19).

[290] Yokoyama S, Takahashi S, Kawakami Y, et al. Effect of vitamin D supplementation on pegylated interferon/ribavirin therapy for chronic hepatitis C genotype 1b: a randomized controlled trial[J]. 2014,21(5):348-356.

[291] Chen H M, Tsai C M, Wu Y C, et al. Effect of walking on circadian rhythms and sleep quality of patients with lung cancer: a randomised controlled trial[J]. BRITISH JOURNAL OF CANCER, 2016,115(11):1304-1312.

[292] Chen H M, Tsai C M, Wu Y C, et al. Effect of walking on circadian rhythms and sleep quality of patients with lung cancer: a randomised controlled trial[J]. 2016,115(11):1304-1312.

[293] Tang W R, Chen W J, Yu C T, et al. Effects of acupressure on fatigue of lung cancer patients undergoing chemotherapy: An experimental pilot study[J]. COMPLEMENTARY THERAPIES IN MEDICINE, 2014,22(4):581-591.

[294] Yao Y. Effects of feiji decoction for soothing the liver combined with psychotherapy on quality of life in primary lung cancer patients[J]. 2012,15(1):27-33.

[295] Yao Y. Effects of feiji decoction for soothing the liver combined with psychotherapy on quality of life in primary lung cancer patients[J]. Chinese Journal of Lung Cancer, 2012,15(1):27-33.

[296] Yao Y. Effects of Feiji decoction for soothing the liver combined with psychotherapy on quality of life in primary lung cancer patients[J]. 2012,15(1):27-33.

[297] Yang M, Liu L, Gan C E, et al. Effects of home-based exercise on exercise capacity, symptoms, and quality of life in patients with lung cancer: A meta-analysis[J]. EUROPEAN JOURNAL OF ONCOLOGY NURSING, 2020,49.

[298] Ferreira Da Rosa Silva C, Dickerson S S, Jungquist C, et al. Effects of morning bright light therapy on circadian activity rhythms in lung cancer survivors[J]. Sleep, 2017,40:A258.

[299] Cheng K, Lee D. Effects of pain, fatigue, insomnia, and mood disturbance on functional status and quality of life of elderly patients with cancer[J]. CRITICAL REVIEWS IN ONCOLOGY HEMATOLOGY, 2011,78(2):127-137.

[300] Chao W R, Hobbs P D, Jong L, et al. Effects of receptor class- and subtype-selective retinoids and an apoptosis-inducing retinoid on the adherent growth of the NIH:OVCAR-3 ovarian cancer cell line in culture[J]. CANCER LETTERS, 1997,115(1):1-7.

[301] Miller R R. Effects of smoking on drug action[J]. Clinical Pharmacology and Therapeutics, 1977,22(5):749-756.

[302] Du J. Effects of the Combination of Continuous Nursing Care and Breathing Exercises on Respiratory Function, Self-Efficacy, and Sleep Disorders in Patients with Lung Cancer Discharged from Hospital[J]. Contrast Media and Molecular Imaging, 2022,2022.

[303] Du J. Effects of the Combination of Continuous Nursing Care and Breathing Exercises on Respiratory Function, Self-Efficacy, and Sleep Disorders in Patients with Lung Cancer Discharged from Hospital[J]. CONTRAST MEDIA & MOLECULAR IMAGING, 2022,2022.

[304] Lv X Y, Zhao Y, Wu Y Q. Effects of the Training of Aerobic Function on Clinical Symptoms and Quality of Life in Patients with Medium and Advanced Lung Cancer[J]. JOURNAL OF HEALTHCARE ENGINEERING, 2022,2022.

[305] Fasciolo G, Nicolini A, Vacca N, et al. Efficacy and safety of moguisteine in comparison with levodropropizine in patients with cough assoclated with chronic obstructive pulmonary disease, lung cancer, or pulmonary fibrosis[J]. Current Therapeutic Research - Clinical and Experimental, 1994,55(3):251-261.

[306] Schnitzer T J, Ekman E F, Spierings E L, et al. Efficacy and safety of tanezumab monotherapy or combined with non-steroidal anti-inflammatory drugs in the treatment of knee or hip osteoarthritis pain[J]. 2015,74(6):1202-1211.

[307] Heywood R, McCarthy A L, Skinner T L. Efficacy of Exercise Interventions in Patients With Advanced Cancer: A Systematic Review[J]. ARCHIVES OF PHYSICAL MEDICINE AND REHABILITATION, 2018,99(12):2595-2620.

[308] Paulsen Ø, Klepstad P, Rosland J H, et al. Efficacy of methylprednisolone on pain, fatigue, and appetite loss in patients with advanced cancer using opioids: A randomized, placebo-controlled, double-blind trial[J]. Journal of Clinical Oncology, 2014,32(29):3221-3228.

[309] Esteban R, Pineda J A, Calleja J L, et al. Efficacy of Sofosbuvir and Velpatasvir, With and Without Ribavirin, in Patients With Hepatitis C Virus Genotype 3 Infection and Cirrhosis[J]. 2018,155(4):1120-1127.

[310] Oshima N, Yamashita T, Hyuga S, et al. Efficiently prepared ephedrine alkaloids-free Ephedra Herb extract: a putative marker and antiproliferative effects[J]. JOURNAL OF NATURAL MEDICINES, 2016,70(3):554-562.

[311] Alhoshani A, Alanazi F E, Alotaibi M R, et al. EGFR Inhibitor Gefitinib Induces Cardiotoxicity through the Modulation of Cardiac PTEN/Akt/FoxO3a Pathway and Reactive Metabolites Formation: In Vivo and in Vitro Rat Studies[J]. CHEMICAL RESEARCH IN TOXICOLOGY, 2020,33(7):1719-1728.

[312] Mraihi Z, Ben Amar J, Bouacha H, et al. EGFR mutation status in Tunisian non-small-cell lung cancer patients evaluated by mutation-specific immunohistochemistry[J]. BMC PULMONARY MEDICINE, 2018,18.

[313] Dragonieri S, Pennazza G, Carratu P, et al. Electronic Nose Technology in Respiratory Diseases[J]. LUNG, 2017,195(2):157-165.

[314] Lee G, Gardner B K, Elashoff D A, et al. Elevated levels of CXC chemokine connective tissue activating peptide (CTAP)-III in lung cancer patients[J]. AMERICAN JOURNAL OF TRANSLATIONAL RESEARCH, 2011,3(3):226-233.

[315] Collins B T. Endobronchial ultrasound fine-needle aspiration biopsy of pulmonary non-small cell carcinoma with subclassification by immunohistochemistry panel[J]. Cancer Cytopathology, 2013,121(3):146-154.

[316] Shepherd M D, Liu T, Méndez C, et al. Engineered biosynthesis of gilvocarcin analogues with altered deoxyhexopyranose moieties[J]. Applied and Environmental Microbiology, 2011,77(2):435-441.

[317] Yin K S, Naing M M, Khine N, et al. Epidermal growth factor receptor variants in patients from Myanmar with lung adenocarcinoma[J]. ASIAN BIOMEDICINE, 2020,14(2):75-81.

[318] Bulutay P, Bilir E, Yildiz S, et al. Epidermal growth factor receptor-mutated lung adenocarcinoma diagnosed from endometrial polyp metastasis: A case report and literature review[J]. TURKISH JOURNAL OF OBSTETRICS AND GYNECOLOGY, 2022,19(1):81-86.

[319] Wang H, Zhang Y, Wang B, et al. Epithelial-myoepithelial carcinoma of the parotid gland with primary lung cancer: A rare case report[J]. Medicine, 2020,99(40):e22483.

[320] Ftanou M. ES15.03 Managing Sleep Difficulties and Cancer[J]. Journal of Thoracic Oncology, 2019,14(10):S52.

[321] De Ruysscher D, Nakaerts K. ES23.04 Optimal Supportive Care During and After Concurrent Chemoradiotherapy and I/O[J]. Journal of Thoracic Oncology, 2019,14(10):S71.

[322] Wang C Y, Xu G, Gao C, et al. Esophageal metastases from primary lung cancer: a case report[J]. JOURNAL OF MEDICAL CASE REPORTS, 2021,15(1).

[323] Fusco M, De Angelis R, Senatore G, et al. Estimates of cancer burden in Campania[J]. TUMORI JOURNAL, 2013,99(3):374-381.

[324] Vanderpuye-Orgle J, Erim D, Qian Y, et al. Estimating the Impact of Delayed Access to Oncology Drugs on Patient Outcomes in Canada[J]. ONCOLOGY AND THERAPY, 2022,10(1):195-210.

[325] Ozturk A, Sarihan S, Ercan I, et al. Evaluating Quality of Life and Pulmonary Function of Long-term Survivors of Non-Small Cell Lung Cancer Treated With Radical or Postoperative Radiotherapy[J]. AMERICAN JOURNAL OF CLINICAL ONCOLOGY-CANCER CLINICAL TRIALS, 2009,32(1):65-72.

[326] Myhren-Bennett A R, McDonnell K K, Davis J, et al. EVALUATING SLEEP QUALITY AS A PRELIMINARY OUTCOME OF BREATHE EASIER: A MINDFULNESS-BASED INTERVENTION FOR SURVIVORS OF LUNG CANCER AND FAMILY MEMBERS (DYADS)[J]. ONCOLOGY NURSING FORUM, 2020,47(2).

[327] Weiss M M, Harmange J C, Polverino A J, et al. Evaluation of a series of naphthamides as potent, orally active vascular endothelial growth factor receptor-2 tyrosine kinase inhibitors[J]. JOURNAL OF MEDICINAL CHEMISTRY, 2008,51(6):1668-1680.

[328] Whithaus K, Fukuoka J, Prihoda T J, et al. Evaluation of Napsin A, Cytokeratin 5/6, p63, and Thyroid Transcription Factor 1 in Adenocarcinoma Versus Squamous Cell Carcinoma of the Lung[J]. ARCHIVES OF PATHOLOGY & LABORATORY MEDICINE, 2012,136(2):155-162.

[329] Zhang C, Schmidt L A, Hatanaka K, et al. Evaluation of Napsin A, TTF-1, p63, p40, and CK5/6 Immunohistochemical Stains in Pulmonary Neuroendocrine Tumors[J]. AMERICAN JOURNAL OF CLINICAL PATHOLOGY, 2014,142(3):320-324.

[330] Zhang C, Schmidt L A, Hatanaka K, et al. Evaluation of Napsin A, TTF-1, p63, p40, and CK5/6 Immunohistochemical Stains in Pulmonary Neuroendocrine Tumors[J]. AMERICAN JOURNAL OF CLINICAL PATHOLOGY, 2014,142(3):320-324.

[331] Zhang C, Schmidt L A, Hatanaka K, et al. Evaluation of Napsin A, TTF-1, p63, p40, and CK5/6 Immunohistochemical Stains in Pulmonary Neuroendocrine Tumors[J]. American Journal of Clinical Pathology, 2014,142(3):320-324.

[332] Sunnetcioglu A, Alp H H, Sertogullarindan B, et al. Evaluation of Oxidative Damage and Antioxidant Mechanisms in COPD, Lung Cancer, and Obstructive Sleep Apnea Syndrome[J]. RESPIRATORY CARE, 2016,61(2):205-211.

[333] Imai Y, Imai K, Kimura T, et al. Evaluation of postoperative pregabalin for attenuation of postoperative shoulder pain after thoracotomy in patients with lung cancer, a preliminary result[J]. General Thoracic and Cardiovascular Surgery, 2014.

[334] Guo D, Liu J, Li Y, et al. Evaluation of predictive values of naples prognostic score in patients with unresectable stage iii non-small cell lung cancer[J]. Journal of Inflammation Research, 2021,14:6129-6141.

[335] Cerri M F, Rezende L, Paes M F, et al. Evaluation of relative expression of SLC34A2/NaPi-IIb in lung cancer cell lines treated with estrogen and PKC and PKA pathway modulators[J]. CANCER RESEARCH, 2014,74(19).

[336] Wang J, Zhou B Y, Lian C L, et al. Evaluation of Subjective Sleep Disturbances in Cancer Patients: A Cross-Sectional Study in a Radiotherapy Department[J]. FRONTIERS IN PSYCHIATRY, 2021,12.

[337] Singh B, Spence R, Steele M L, et al. Exercise for Individuals With Lung Cancer: A Systematic Review and Meta-Analysis of Adverse Events, Feasibility, and Effectiveness[J]. SEMINARS IN ONCOLOGY NURSING, 2020,36(5).

[338] Khan S, Ali S, Muhammad. Exhaustive Review on Lung Cancers: Novel Technologies[J]. CURRENT MEDICAL IMAGING, 2019,15(9):873-883.

[339] Khan S, Ali S, Muhammad. Exhaustive Review on Lung Cancers: Novel Technologies[J]. Curr Med Imaging Rev, 2019,15(9):873-883.

[340] Mao R Q, Liu M, Shu X F, et al. Expanding the Immunophenotype Spectrum of SMARCA4-Deficient Non-Small Cell Lung Carcinomas: A Case Series with Neuroendocrine Markers Expression[J]. INTERNATIONAL JOURNAL OF SURGICAL PATHOLOGY, 2022,30(3):251-259.

[341] Kakinuma R, Nishiwaki Y, Yano H, et al. Experience with psychotropic agents Pyrethia and Contomin in the terminal care of lung cancer patients[J]. Gan no rinsho. Japan journal of cancer clinics, 1984,30(4):344-348.

[342] Dickerson S S, Abbu S E, Gothard S, et al. Experiences of Patients With Advanced Lung Cancer: Being Resigned to Sleep-Wake Disturbances While Maintaining Hope for Optimal Treatment Outcomes[J]. Cancer Nurs, 2015,38(5):358-365.

[343] Wise J, Salazar-Gonzalez R A, Habil M R, et al. Expression of arylamine N-acetyltransferase 2 activity in immortalized human bronchial epithelial cells[J]. TOXICOLOGY AND APPLIED PHARMACOLOGY, 2022,442.

[344] Gridelli C, Gallo C, Ceribelli A, et al. Factorial phase III randomised trial of rofecoxib and prolonged constant infusion of gemcitabine in advanced non-small-cell lung cancer: the GEmcitabine-COxib in NSCLC (GECO) study[J]. LANCET ONCOLOGY, 2007,8(6):500-512.

[345] Takemura N, Ho M H, Cheung D, et al. Factors associated with perceived cognitive impairment in patients with advanced lung cancer: a cross-sectional analysis[J]. SUPPORTIVE CARE IN CANCER, 2022,30(11):9607-9614.

[346] Lin H H, Chen K H, Chiu C H, et al. Factors related to quality of life after video-assisted thoracoscopic surgery in patients with stage I adenocarcinoma lung cancer: A longitudinal study[J]. EUROPEAN JOURNAL OF ONCOLOGY NURSING, 2022,61.

[347] Lin H H, Chen K H, Chiu C H, et al. Factors related to quality of life after video-assisted thoracoscopic surgery in patients with stage I adenocarcinoma lung cancer: A longitudinal study[J]. European journal of oncology nursing : the official journal of European Oncology Nursing Society, 2022,61:102225.

[348] Charles C, Boinon D, Fasse L, et al. Feasibility of a self-screening for insomnia and acceptability of an online targeted video-based cognitive behavioral program in cancer outpatients: SLEEP-4-ALL-1 study design[J]. Annals of Oncology, 2019,30:i71.

[349] Chernin T. First in new class for insomnia[J]. Drug Topics, 2004,148(21).

[350] Hakkarainen R, Partonen T, Haukka J, et al. Food and nutrient intake in relation to mental wellbeing[J]. Nutrition Journal, 2004,3.

[351] Hakkarainen R, Partonen T, Haukka J, et al. Food and nutrient intake in relation to mental wellbeing[J]. NUTRITION JOURNAL, 2004,3.

[352] Nakamura Y, Mukai M, Hiraiwa S, et al. Free-floating cancer cells in lymph node sinuses of hilar lymph node-positive patients with non-small cell lung cancer[J]. MOLECULAR MEDICINE REPORTS, 2018,18(1):1081-1087.

[353] Shimohata T, Ozawa T, Nakayama H, et al. Frequency of nocturnal sudden death in patients with multiple system atrophy[J]. Journal of Neurology, 2008,255(10):1483-1485.

[354] Shimohata T, Ozawa T, Nakayama H, et al. Frequency of nocturnal sudden death in patients with multiple system atrophy[J]. JOURNAL OF NEUROLOGY, 2008,255(10):1483-1485.

[355] Fodeh S J, Lazenby M, Bai M, et al. Functional impairments as symptoms in the symptom cluster analysis of patients newly diagnosed with advanced cancer[J]. Journal of Pain and Symptom Management, 2013,46(4):500-510.

[356] Zhang L, Tatsuno T, Hasegawa I, et al. Furanonaphthoquinones from Tabebuia avellanedae induce cell cycle arrest and apoptosis in the human non-small cell lung cancer cell line A549[J]. PHYTOCHEMISTRY LETTERS, 2015,11:9-17.

[357] Ghanimeh M A, Bourbia A, Sadeddin E, et al. Gastric and breast metastasis from lung adenocarcinoma: A case report[J]. American Journal of Gastroenterology, 2015,110:S511.

[358] Kim M J, Hong J H, Park E S, et al. Gastric metastasis from primary lung adenocarcinoma mimicking primary gastric cancer[J]. World Journal of Gastrointestinal Oncology, 2015,7(3):12-16.

[359] Kim M J, Hong J H, Park E S, et al. Gastric metastasis from primary lung adenocarcinoma mimicking primary gastric cancer[J]. WORLD JOURNAL OF GASTROINTESTINAL ONCOLOGY, 2015,7(3).

[360] Zhu M J, Shu J C, Liu X Y, et al. Gastrointestinal hemorrhage caused by duodenal metastasis from a primary lung adenocarcinoma: A case report[J]. MOLECULAR AND CLINICAL ONCOLOGY, 2021,14(3).

[361] Schultz H, Marwitz S, Baron-Luhr B, et al. Generation and evaluation of a monoclonal antibody, designated MAdL, as a new specific marker for adenocarcinomas of the lung[J]. BRITISH JOURNAL OF CANCER, 2011,105(5):673-681.

[362] Shen J, Zhou H, Liu J, et al. Genetic Liability to Insomnia and Lung Cancer Risk: A Mendelian Randomization Analysis[J]. Front Genet, 2021,12:756908.

[363] Shen J, Zhou H, Liu J, et al. Genetic Liability to Insomnia and Lung Cancer Risk: A Mendelian Randomization Analysis[J]. Frontiers in Genetics, 2021,12.

[364] Yuan S, Xiong Y, Michaelsson M, et al. Genetically predicted education attainment in relation to somatic and mental health[J]. SCIENTIFIC REPORTS, 2021,11(1).

[365] Yuan S, Xiong Y, Michaëlsson M, et al. Genetically predicted education attainment in relation to somatic and mental health[J]. Scientific reports, 2021,11(1):4296.

[366] Huo Z Y, Ge F, Li C C, et al. Genetically predicted insomnia and lung cancer risk: a Mendelian randomization study[J]. SLEEP MEDICINE, 2021,87:183-190.

[367] Huo Z, Ge F, Li C, et al. Genetically predicted insomnia and lung cancer risk: a Mendelian randomization study[J]. Sleep Medicine, 2021,87:183-190.

[368] Chan E D, Welsh C H. Geriatric respiratory medicine.[J]. Chest, 1998,114(6):1704-1733.

[369] Fioravanzo A, Simbolo M, Giampiccolo D, et al. Glioblastoma with carcinomatous differentiation or tumour-totumour metastasis?[J]. Virchows Archiv, 2019,475:S379-S380.

[370] Li Q K, Shah P, Li Y, et al. Glycoproteomic Analysis of Bronchoalveolar Lavage (BAL) Fluid Identifies Tumor-Associated Glycoproteins from Lung Adenocarcinonna[J]. JOURNAL OF PROTEOME RESEARCH, 2013,12(8):3689-3696.

[371] Kai Y, Amatya V J, Kushitani K, et al. Glypican-1 is a novel immunohistochemical marker to differentiate poorly differentiated squamous cell carcinoma from solid predominant adenocarcinoma of the lung[J]. TRANSLATIONAL LUNG CANCER RESEARCH, 2021,10(2):766.

[372] Filippini T, Malavolti M, Borrelli F, et al. Green tea (Camellia sinensis) for the prevention of cancer[J]. Cochrane Database of Systematic Reviews, 2020(3).

[373] Filippini T, Malavolti M, Borrelli F, et al. Green tea (Camellia sinensis) for the prevention of cancer[J]. Cochrane Database of Systematic Reviews, 2020,2020(3).

[374] Beattie V. Guideline for telephone follow up for patients undergoing thoracic surgery[J]. Lung Cancer, 2014,83:S75.

[375] Montella M, Gridelli C, Crispo A, et al. Has lung cancer in the elderly different characteristics at presentation?[J]. ONCOLOGY REPORTS, 2002,9(5):1093-1096.

[376] Hollister L E. Health aspects of cannabis[J]. Pharmacological Reviews, 1986,38(1):1-20.

[377] Witlox W, Ramaekers B, Joore M A, et al. Health-related quality of life after prophylactic cranial irradiation for stage III non-small cell lung cancer patients: results from the NVALT-11/DLCRG-02 phase III study[J]. 2020,144:65-71.

[378] Botello-Manilla A E, Lopez-Sanchez G N, Chavez-Tapia N C, et al. Hepatic steatosis and respiratory diseases: a new panorama[J]. ANNALS OF HEPATOLOGY, 2021,24.

[379] Barbareschi M, Cantaloni C, Del Vescovo V, et al. Heterogeneity of Large Cell Carcinoma of the Lung An Immunophenotypic and miRNA-Based Analysis[J]. AMERICAN JOURNAL OF CLINICAL PATHOLOGY, 2011,136(5):773-782.

[380] Barbareschi M, Cantaloni C, Del Vescovo V, et al. Heterogeneity of large cell carcinoma of the lung: An immunophenotypic and miRNA-based analysis[J]. Virchows Archiv, 2011,459:S53-S54.

[381] Wise J T F, Salazar-González R A, Walls K M, et al. Hexavalent chromium increases the metabolism and genotoxicity of aromatic amine carcinogens 4-aminobiphenyl and β-naphthylamine in immortalized human lung epithelial cells[J]. Toxicology and Applied Pharmacology, 2022,449.

[382] Zhang Z X, Ye S, Zhang M, et al. High expression of SLC34A2 is a favorable prognostic marker in lung adenocarcinoma patients[J]. TUMOR BIOLOGY, 2017,39(7).

[383] Szade J, Majewska H I, Zaczek A, et al. HIGH-GRADE NON-SMALL CELL LUNG CARCINOMA: A COMPARATIVE ANALYSIS OF THE PHENOTYPIC PROFILE IN SMALL BIOPSIES WITH THE CORRESPONDING POSTOPERATIVE MATERIAL[J]. POLISH JOURNAL OF PATHOLOGY, 2019,70(2):100-108.

[384] Qian X H, Li Z G, Yang Q. Highly efficient antitumor agents of heterocycles containing sulfur atom: Linear and angular thiazonaphthalimides against human lung cancer cell in vitro[J]. BIOORGANIC & MEDICINAL CHEMISTRY, 2007,15(21):6846-6851.

[385] Li J J, Tian Z Z, Xu Z S, et al. Highly potent half- sandwich iridium and ruthenium complexes as lysosome- targeted imaging and anticancer agents[J]. DALTON TRANSACTIONS, 2018,47(44):15772-15782.

[386] Forest F, Yvorel V, Karpathiou G, et al. Histomolecular profiling of pleomorphic, spindle cell, and giant cell carcinoma of the lung for targeted therapies[J]. HUMAN PATHOLOGY, 2016,49:99-106.

[387] Roden A C, Garcia J J, Wehrs R N, et al. Histopathologic, immunophenotypic and cytogenetic features of pulmonary mucoepidermoid carcinoma[J]. MODERN PATHOLOGY, 2014,27(11):1479-1488.

[388] Roden A C, García J J, Wehrs R N, et al. Histopathologic, immunophenotypic and cytogenetic features of pulmonary mucoepidermoid carcinoma[J]. Modern Pathology, 2014,27(11):1479-1488.

[389] Wang S, Zhang H, Liu C, et al. Human leukocyte antigen-haploidentical donor-derived cytokine-induced killer cells are safe and prolong the survival of patients with advanced non-small cell lung cancer[J]. Oncol Lett, 2014,8(6):2727-2733.

[390] Wang S Y, Zhang H, Liu C, et al. Human leukocyte antigen-haploidentical donor-derived cytokine-induced killer cells are safe and prolong the survival of patients with advanced non-small cell lung cancer[J]. ONCOLOGY LETTERS, 2014,8(6):2727-2733.

[391] Hirano T, Auer C, Maeda M, et al. Human tissue distribution of TA02, which is homologous with a new type of aspartic proteinase, napsin A[J]. JAPANESE JOURNAL OF CANCER RESEARCH, 2000,91(10):1015-1021.

[392] Lin H M, Pan X Y, Biller A, et al. Humanistic burden of living with anaplastic lymphoma kinase-positive non-small-cell lung cancer: findings from the ALKConnect patient insight network and research platform[J]. LUNG CANCER MANAGEMENT, 2021,10(1).

[393] Rojas-Marcos I, Graus F, Sanz G, et al. Hypersomnia as presenting symptom of anti-Ma2-associated encephalitis: Case study[J]. NEURO-ONCOLOGY, 2007,9(1):75-77.

[394] Bailey L A, Nascarella M A, Kerper L E, et al. Hypothesis-based weight-of-evidence evaluation and risk assessment for naphthalene carcinogenesis[J]. CRITICAL REVIEWS IN TOXICOLOGY, 2016,46(1):1-42.

[395] Ozbun L L, You L, Kiang S, et al. Identification of differentially expressed nucleolar TGF-beta 1 target (DENTT) in human lung cancer cells that is a new member of the TSPY/SET/NAP-1 superfamily[J]. GENOMICS, 2001,73(2):179-193.

[396] Ozbun L L, You L, Kiang S, et al. Identification of differentially expressed nucleolar TGF-beta1 target (DENTT) in human lung cancer cells that is a new member of the TSPY/SET/NAP-1 superfamily[J]. Genomics, 2001,73(2):179-193.

[397] Luo B, Gu Y Y, Wang X D, et al. Identification of potential drugs for diffuse large b-cell lymphoma based on bioinformatics and Connectivity Map database[J]. PATHOLOGY RESEARCH AND PRACTICE, 2018,214(11):1854-1867.

[398] Tejada M, Viele C, Kober K M, et al. Identification of subgroups of chemotherapy patients with distinct sleep disturbance profiles and associated co-occurring symptoms[J]. Sleep, 2019,42(10).

[399] Buendia-Roldan I, Mejia M, Navarro C, et al. Idiopathic pulmonary fibrosis: Clinical behavior and aging associated comorbidities[J]. RESPIRATORY MEDICINE, 2017,129:46-52.

[400] King C S, Nathan S D. Idiopathic pulmonary fibrosis: effects and optimal management of comorbidities[J]. LANCET RESPIRATORY MEDICINE, 2017,5(1):72-84.

[401] Cui Y, Yang X, Zhu W, et al. Immune response, clinical outcome and safety of dendritic cell vaccine in combination with cytokine-induced killer cell therapy in cancer patients[J]. Oncology Letters, 2013,6(2):537-541.

[402] Jin L, Liu Y, Wang X, et al. Immunohistochemical analysis and comparison of napsin A, TTF1, SPA and CK7 expression in primary lung adenocarcinoma[J]. BIOTECHNIC & HISTOCHEMISTRY, 2018,93(5):364-372.

[403] Vidarsdottir H, Tran L, Nodin B, et al. Immunohistochemical profiles in primary lung cancers and epithelial pulmonary metastases[J]. HUMAN PATHOLOGY, 2019,84:221-230.

[404] Sakai Y, Nakai T, Ohbayashi C, et al. Immunohistochemical profiling of ALK fusion gene-positive adenocarcinomas of the lung[J]. International Journal of Surgical Pathology, 2013,21(5):476-482.

[405] Righi L, Graziano P, Fornari A, et al. Immunohistochemical subtyping of nonsmall cell lung cancer not otherwise specified in fine-needle aspiration cytology: A retrospective study of 103 cases with surgical correlation[J]. Cancer, 2011,117(15):3416-3423.

[406] Brunnstrom H, Johansson L, Jirstrom K, et al. Immunohistochemistry in the Differential Diagnostics of Primary Lung Cancer An Investigation Within the Southern Swedish Lung Cancer Study[J]. AMERICAN JOURNAL OF CLINICAL PATHOLOGY, 2013,140(1):37-46.

[407] Brunnstrom H, Johansson L, Jirstrom K, et al. Immunohistochemistry in the Differential Diagnostics of Primary Lung Cancer An Investigation Within the Southern Swedish Lung Cancer Study[J]. AMERICAN JOURNAL OF CLINICAL PATHOLOGY, 2013,140(1):37-46.

[408] Rahman M, Arram E O, Hamid A A. Immunohistochemistry of non-small cell type of lung cancer can be used as a separate prognostic factor?[J]. EGYPTIAN JOURNAL OF CHEST DISEASES AND TUBERCULOSIS, 2019,68(4):595-600.

[409] Zarogoulidis P, Petridis D, Kosmidis C, et al. Immunotherapy and Chemotherapy Versus Sleep Disturbances for NSCLC Patients[J]. Current oncology (Toronto, Ont.), 2023,30(2):1999-2006.

[410] Kulkarni A, Jewett P, Blaes A H, et al. Impact of antibiotics on residual cancer burden and pathologic response rates during neoadjuvant pembrolizumab in breast cancer (BC)[J]. Journal of Clinical Oncology, 2020,38(15).

[411] Rodríguez C F, Sánchez C P, Fernández E V, et al. Impact of anxiety and depression on the physical status and daily routines of cancer patients during chemotherapy[J]. Psicothema, 2011,23(3):374-381.

[412] van Seijen M, Brcic L, Gonzales A N, et al. Impact of delayed and prolonged fixation on the evaluation of immunohistochemical staining on lung carcinoma resection specimen[J]. VIRCHOWS ARCHIV, 2019,475(2):191-199.

[413] Tanaka K, Akechi T, Okuyama T, et al. Impact of dyspnea, pain, and fatigue on daily life activities in ambulatory patients with advanced lung cancer[J]. JOURNAL OF PAIN AND SYMPTOM MANAGEMENT, 2002,23(5):417-423.

[414] Gelibter A, Ceribelli A, Pollera C F, et al. Impact of gefitinib ('Iressa') treatment on the quality of life of patients with advanced non-small-cell lung cancer[J]. JOURNAL OF CANCER RESEARCH AND CLINICAL ONCOLOGY, 2005,131(12):783-788.

[415] Gelibter A, Ceribelli A, Pollera C F, et al. Impact of gefitinib ('Iressa') treatment on the quality of life of patients with advanced non-small-cell lung cancer[J]. Journal of Cancer Research and Clinical Oncology, 2005,131(12):783-788.

[416] Peters S, Shaw A T, Besse B, et al. Impact of lorlatinib on patient-reported outcomes in patients with advanced ALK-positive or ROS1-positive non-small cell lung cancer[J]. LUNG CANCER, 2020,144:10-19.

[417] Cao Q, Zhang Q, Li X C, et al. Impact of sleep status on lung adenocarcinoma risk: a prospective cohort study[J]. European Review for Medical and Pharmacological Sciences, 2022,26(20):7641-7648.

[418] Cao Q, Zhang Q, Li X C, et al. Impact of sleep status on lung adenocarcinoma risk: a prospective cohort study[J]. EUROPEAN REVIEW FOR MEDICAL AND PHARMACOLOGICAL SCIENCES, 2022,26(20):7641-7648.

[419] Zhang M, Guan L. Impact on neutrophil-to-lymphocyte ratio and quality of life in the patients of non-small-cell lung cancer treated with grain-size moxibustion: a randomized controlled trial[J]. Zhongguo zhen jiu = Chinese acupuncture & moxibustion, 2016,36(4):342-346.

[420] Lee K, Cho M, Miaskowski C, et al. Impaired sleep and rhythms in persons with cancer[J]. SLEEP MEDICINE REVIEWS, 2004,8(3):199-212.

[421] Sun S Y, Yue P, Shroot B, et al. Implication of c-Myc in apoptosis induced by the retinoid CD437 in human lung carcinoma cells[J]. ONCOGENE, 1999,18(26):3894-3901.

[422] Huang G B, Chen S, Qin Q P, et al. In vitro and in vivo activity of novel platinum(ii) complexes with naphthalene imide derivatives inhibiting human non-small cell lung cancer cells[J]. NEW JOURNAL OF CHEMISTRY, 2019,43(21):8146-8152.

[423] Maciel L, de Freitas W R, Bull E S, et al. In vitro and in vivo anti-proliferative activity and ultrastructure investigations of a copper(II) complex toward human lung cancer cell NCI-H460[J]. JOURNAL OF INORGANIC BIOCHEMISTRY, 2020,210.

[424] Skoulidis F. Inactivating STK11/LKB1 genomic alterations are a major driver of primary resistance to PD-1 axis blockade in non-squamous non-small cell lung cancer[J]. Cancer Research, 2019,79(13).

[425] Varga J L, Schally A V, Horvath J E, et al. Increased activity of antagonists of growth hormone-releasing hormone substituted at positions 8, 9, and 10[J]. PROCEEDINGS OF THE NATIONAL ACADEMY OF SCIENCES OF THE UNITED STATES OF AMERICA, 2004,101(6):1708-1713.

[426] Calio A, Lever V, Rossi A, et al. Increased frequency of bronchiolar histotypes in lung carcinomas associated with idiopathic pulmonary fibrosis[J]. HISTOPATHOLOGY, 2017,71(5):725-735.

[427] Tomioka K, Obayashi K, Saeki K, et al. Increased risk of lung cancer associated with occupational exposure to benzidine and/or beta-naphthylamine[J]. INTERNATIONAL ARCHIVES OF OCCUPATIONAL AND ENVIRONMENTAL HEALTH, 2015,88(4):455-465.

[428] Henoch I, Ploner A, Tishelman C. Increasing stringency in symptom cluster research: a methodological exploration of symptom clusters in patients with inoperable lung cancer[J]. Oncol Nurs Forum, 2009,36(6):E282-E292.

[429] Chen K C, Tsai S W, Shie R H, et al. Indoor Air Pollution Increases the Risk of Lung Cancer[J]. INTERNATIONAL JOURNAL OF ENVIRONMENTAL RESEARCH AND PUBLIC HEALTH, 2022,19(3).

[430] Sun S Y, Yue P, Shroot B, et al. Induction of apoptosis in human non-small cell lung carcinoma cells by the novel synthetic retinoid CD437[J]. JOURNAL OF CELLULAR PHYSIOLOGY, 1997,173(2):279-284.

[431] Kadara H, Schroeder C P, Lotan D, et al. Induction of GDF-15/NAG-1/MIC-1 in human lung carcinoma cells by retinoid-related molecules and assessment of its role in apoptosis[J]. CANCER BIOLOGY & THERAPY, 2006,5(5):518-522.

[432] Frass M, Friehs H, Thallinger C, et al. Influence of adjunctive classical homeopathy on global health status and subjective wellbeing in cancer patients - A pragmatic randomized controlled trial[J]. Complementary Therapies in Medicine, 2015,23(3):309-317.

[433] Suren M, Okan I, Kaya Z, et al. Initial experience with delivery of palliative care to terminal cancer patients[J]. TURKISH JOURNAL OF MEDICAL SCIENCES, 2016,46(2):388-392.

[434] Süren M, Okan İ, Kaya Z, et al. Initial experience with delivery of palliative care to terminal cancer patients[J]. Turkish Journal of Medical Sciences, 2016,46(2):388.

[435] Rodríguez-Torres J, López-López L, Cabrera-Martos I, et al. Inpatient Step Counts, Symptom Severity, and Perceived Health Status after Lung Resection Surgery[J]. Cancer Nursing, 2021,44(5):361-368.

[436] Mercadante S, Valle A, Cartoni C, et al. Insomnia in patients with advanced lung cancer admitted to palliative care services[J]. INTERNATIONAL JOURNAL OF CLINICAL PRACTICE.

[437] Mercadante S, Valle A, Cartoni C, et al. Insomnia in patients with advanced lung cancer admitted to palliative care services[J]. International Journal of Clinical Practice, 2021,75(10).

[438] Bradley T D, Miller Y E, Martinez F J, et al. Interstitial lung disease, lung cancer, lung transplantation, pulmonary vascular disorders, and sleep-disordered breathing in AJRCCM in 2004[J]. AMERICAN JOURNAL OF RESPIRATORY AND CRITICAL CARE MEDICINE, 2005,171(7):675-685.

[439] Dubey A, Park D W, Kwon J E, et al. Investigation of the biological and anti-cancer properties of ellagic acid-encapsulated nano-sized metalla-cages[J]. INTERNATIONAL JOURNAL OF NANOMEDICINE, 2015,10:227.

[440] Akagi K, Sano M, Ogawa K, et al. Involvement of toxicity as an early event in urinary bladder carcinogenesis induced by phenethyl isothiocyanate, benzyl isothiocyanate, and analogues in F344 rats[J]. TOXICOLOGIC PATHOLOGY, 2003,31(4):388-396.

[441] Harper E, Talbot C J. Is it Time to Change Radiotherapy: The Dawning of Chronoradiotherapy?[J]. CLINICAL ONCOLOGY, 2019,31(5):326-335.

[442] Kaur H, Onsare J G, Sharma V, et al. Isolation, purification and characterization of novel antimicrobial compound 7-methoxy-2,2-dimethyl-4-octa-4 ',6 '-dienyl-2H-napthalene-1-one from Penicillium sp and its cytotoxicity studies[J]. AMB EXPRESS, 2015,5.

[443] Gorini G, Moshammer H, Sbrogiò L, et al. Italy and Austria before and after study: Second-hand smoke exposure in hospitality premises before and after 2 years from the introduction of the Italian smoking ban[J]. Indoor Air, 2008,18(4):328-334.

[444] Luo S. Joint analysis of stochastic processes with application to smoking patterns and insomnia[J]. STATISTICS IN MEDICINE, 2013,32(29):5133-5144.

[445] Krasinskas A M, Chiosea S I, Pal T, et al. KRAS mutational analysis and immunohistochemical studies can help distinguish pancreatic metastases from primary lung adenocarcinomas[J]. MODERN PATHOLOGY, 2014,27(2):262-270.

[446] Warth A, Muley T, Herpel E, et al. Large-scale comparative analyses of immunomarkers for diagnostic subtyping of non-small-cell lung cancer biopsies[J]. HISTOPATHOLOGY, 2012,61(6):1017-1025.

[447] Weiss C, Kwon M, Dickerson S, et al. LEVEL OF AGREEMENT BETWEEN OBJECTIVE AND SUBJECTIVE SLEEP MEASURES IN LUNG CANCER SURVIVORS WITH INSOMNIA[J]. SLEEP, 2020,43:A399.

[448] Freo U, Ori C, Ambrosio F. Lidocaine 5% medicated plaster for localized neuropathic pain in thoracic surgical patients[J]. CURRENT MEDICAL RESEARCH AND OPINION, 2017,33(3):489-493.

[449] Harkati I, Hilali M K, Oumghar N, et al. Lifestyle and Sociodemographic and Economic Characteristics of Patients with Lung Cancer in Morocco[J]. CANADIAN RESPIRATORY JOURNAL, 2020,2020.

[450] Murphy R A, Darvishian M, Qi J, et al. Lifestyle factors and lung cancer risk among never smokers in the Canadian Partnership for Tomorrow’s Health (CanPath)[J]. Cancer Causes and Control, 2022,33(6):913-918.

[451] Chen H M, Sun X L, Wang G D, et al. LiGa5O8:Cr-based theranostic nanoparticles for imaging-guided X-ray induced photodynamic therapy of deep-seated tumors[J]. MATERIALS HORIZONS, 2017,4(6):1092-1101.

[452] Bisceglie F, Pelosi G, Orsoni N, et al. Light Triggers the Antiproliferative Activity of Naphthalimide-Conjugated (η6-arene)ruthenium(II) Complexes[J]. International Journal of Molecular Sciences, 2022,23(14).

[453] Milano G, Innocenti F, Minami H. Liposomal irinotecan (Onivyde): Exemplifying the benefits of nanotherapeutic drugs[J]. CANCER SCIENCE, 2022,113(7):2224-2231.

[454] Wu M H, Xu Y, Fitch W L, et al. Liquid chromatography/mass spectrometry methods for measuring dipeptide abundance in non-small-cell lung cancer[J]. RAPID COMMUNICATIONS IN MASS SPECTROMETRY, 2013,27(18):2091-2098.

[455] Ali F, Yamaguchi K, Fukuoka M, et al. Logical design of an anti-cancer agent targeting the plant homeodomain in Pygopus2[J]. CANCER SCIENCE, 2016,107(9):1321-1328.

[456] Tang L L, Pang Y, He Y, et al. Longitudinal study of symptom burden in outpatients with advanced cancers based on electronic Patient-Reported Outcome (ePRO) platform: a single institution, prospective study protocol[J]. BMJ OPEN, 2020,10(11).

[457] Tang L, Pang Y, He Y, et al. Longitudinal study of symptom burden in outpatients with advanced cancers based on electronic Patient-Reported Outcome (ePRO) platform: A single institution, prospective study protocol[J]. BMJ Open, 2020,10(11).

[458] Lin Y Y, Rau K M, Lin C C. Longitudinal study on the impact of physical activity on the symptoms of lung cancer survivors[J]. SUPPORTIVE CARE IN CANCER, 2015,23(12):3545-3553.

[459] Lin Y Y, Rau K M, Lin C C. Longitudinal study on the impact of physical activity on the symptoms of lung cancer survivors[J]. Supportive Care in Cancer, 2015,23(12):3545-3553.

[460] Lin Y, Rau K, Lin C. Longitudinal study on the impact of physical activityon the symptoms of lung cancer[J]. Supportive Care in Cancer, 2015,23(1):S278.

[461] Izumi Y, Mukai M, Kikuchi K, et al. Long-term survival after incomplete resection of immunohistochemically diagnosed T0N1 lung cancer: Report of a case[J]. SURGERY TODAY, 2006,36(3):270-273.

[462] Linnoila R I, Jensen-Taubman S, Kazanjian A, et al. Loss of GFI1 impairs pulmonary neuroendorine cell proliferation, but the neuroendocrine phenotype has limited impact on post-naphthalene airway repair[J]. LABORATORY INVESTIGATION, 2007,87(4):336-344.

[463] Alessandretti M, Buzaid A C, Brandão R, et al. Low-dose bevacizumab is effective in radiation-induced necrosis[J]. Case Reports in Oncology, 2013,6(3):598-601.

[464] Torres M, Martinez-Garcia M Á, Campos-Rodriguez F, et al. Lung cancer aggressiveness in an intermittent hypoxia murine model of postmenopausal sleep apnea[J]. Menopause, 2020,27(6):706-713.

[465] Rossi E D, Wiles A, Vecchione A. Lung cancer and molecular testing in small biopsies versus cytology: The Logics of Worlds[J]. Cancer Cytopathology, 2020,128(9):637-641.

[466] Rowntree R A, Hosseinzadeh H. Lung Cancer and Self-Management Interventions: A Systematic Review of Randomised Controlled Trials[J]. INTERNATIONAL JOURNAL OF ENVIRONMENTAL RESEARCH AND PUBLIC HEALTH, 2022,19(1).

[467] Schwartzberg L S. Lung cancer and younger patients most plagued by insomnia: Commentary[J]. Oncology Report, 2008(FALL):101.

[468] Liang D, Wang J X, Li D J, et al. Lung Cancer in Never-Smokers: A Multicenter Case-Control Study in North China[J]. FRONTIERS IN ONCOLOGY, 2019,9.

[469] Chatkin J M, Zabert G, Zabert I, et al. Lung Disease Associated With Marijuana Use[J]. Archivos de Bronconeumologia, 2017,53(9):510-515.

[470] Otto W R. Lung epithelial stem cells[J]. JOURNAL OF PATHOLOGY, 2002,197(4):527-535.

[471] Ruiz P, Kovarik G. Lung mechanics and gas exchange in one-lung ventilation following contralateral resection[J]. CANADIAN JOURNAL OF ANAESTHESIA-JOURNAL CANADIEN D ANESTHESIE, 2005,52(9):986-989.

[472] Wang F, Shen M H, Cao D, et al. Malignant Ciliated Muconodular Papillary Tumors of the Lung: A Case Report[J]. INTERNATIONAL JOURNAL OF SURGICAL PATHOLOGY, 2021,29(5):520-523.

[473] Iglesias J R, Diez-Manglano J, Garcia F L, et al. Management of the COPD Patient with Comorbidities: An Experts Recommendation Document[J]. INTERNATIONAL JOURNAL OF CHRONIC OBSTRUCTIVE PULMONARY DISEASE, 2020,15:1015-1037.

[474] Merchut M P. Management of voltage-gated potassium channel antibody disorders[J]. Neurologic Clinics, 2010,28(4):941-959.

[475] Chen D Y, Yin Z Y, Fang B. Measurements and status of sleep quality in patients with cancers[J]. SUPPORTIVE CARE IN CANCER, 2018,26(2):405-414.

[476] Sun S Y, Yue P, Wu G S, et al. Mechanisms of apoptosis induced by the synthetic retinoid CD437 in human non-small cell lung carcinoma cells[J]. ONCOGENE, 1999,18(14):2357-2365.

[477] Lokshin A, Peng X J, Campbell P G, et al. Mechanisms of growth stimulation by suramin in non-small-cell lung cancer cell lines[J]. CANCER CHEMOTHERAPY AND PHARMACOLOGY, 1999,43(4):341-347.

[478] Yu H, Moshen R, Ellison K, et al. MERS67 is a Novel anti-NaPi2b Antibody and Demonstrates Differential Expression Patterns in Lung Cancer Histologic Subtypes[J]. JOURNAL OF THORACIC ONCOLOGY, 2018,13(10):S770.

[479] Harihar S, Mone N, Satpute S K, et al. Metal complexes of a pro-vitamin K3 analog phthiocol (2-hydroxy-3-methylnaphthalene-1,4-dione): synthesis, characterization, and anticancer activity[J]. DALTON TRANSACTIONS, 2022,51(45):17338-17353.

[480] Tauchi-Nishi P, Sae-Ow W, Kaneshiro R, et al. Metastatic lung adenocarcinoma to the kidney diagnosed by urine cytology: A case report[J]. Analytical and Quantitative Cytopathology and Histopathology, 2014,36(6):345-350.

[481] Ozdemir H G, Cakmak P. Metastatic lung adenocarcinoma with incidental finding meningioma in a frontal lobe in a young patient[J]. Clinical Neuropathology, 2021,40(1):S72.

[482] McDonnell K K, Owens O L, Umari F. Mindfulness-Based Interventions for Survivors of Lung Cancer and Their Partners: A Systematic Review[J]. INTERNATIONAL JOURNAL OF BEHAVIORAL MEDICINE.

[483] Campelo M, Zhou C C, Ramalingam S S, et al. Mobocertinib (TAK-788) in EGFR Exon 20 Insertion+ Metastatic NSCLC: Patient-Reported Outcomes from EXCLAIM Extension Cohort[J]. JOURNAL OF CLINICAL MEDICINE, 2023,12(1).

[484] Spathis A, Dhillan R, Booden D, et al. Modafinil for the treatment of fatigue in lung cancer: a pilot study[J]. 2009,23(4):325-331.

[485] Spathis A, Dhillan R, Booden D, et al. Modafinil for the treatment of fatigue in lung cancer: a pilot study[J]. PALLIATIVE MEDICINE, 2009,23(4):325-331.

[486] Ha D M, Prochazka A V, Bekelman D B, et al. Modifiable factors associated with health-related quality of life among lung cancer survivors following curative intent therapy[J]. LUNG CANCER, 2022,163:42-50.

[487] Ha D M, Prochazka A V, Bekelman D B, et al. Modifiable factors associated with health-related quality of life among lung cancer survivors following curative intent therapy[J]. Lung Cancer, 2022,163:42-50.

[488] Li Y, Lin B Z, Agadir A, et al. Molecular determinants of AHPN (CD437)-induced growth arrest and apoptosis in human lung cancer cell lines[J]. MOLECULAR AND CELLULAR BIOLOGY, 1998,18(8):4719-4731.

[489] Terasaki Y, Suzuki T, Tonaki K, et al. Molecular hydrogen attenuates gefitinib-induced exacerbation of naphthalene-evoked acute lung injury through a reduction in oxidative stress and inflammation[J]. LABORATORY INVESTIGATION, 2019,99(6):793-806.

[490] Ahmadian N, Mehrnejad F, Amininasab M. Molecular Insight into the Interaction between Camptothecin and Acyclic Cucurbit[4]urils as Efficient Nanocontainers in Comparison with Cucurbit[7]uril: Molecular Docking and Molecular Dynamics Simulation[J]. JOURNAL OF CHEMICAL INFORMATION AND MODELING, 2020,60(3):1791-1803.

[491] Mishur R J, Griffin M E, Battle C H, et al. Molecular recognition and enhancement of aqueous solubility and bioactivity of CD437 by beta-cyclodextrin[J]. BIOORGANIC & MEDICINAL CHEMISTRY LETTERS, 2011,21(2):857-860.

[492] Tremmas I, Petsatodis G, Potoupnis M, et al. Monitoring changes in quality of life in patients with lung cancer under treatment with chemotherapy and co administration of zoledronic acid by using specialized questionnaires[J]. JOURNAL OF CANCER, 2018,9(10):1731-1736.

[493] Tremmas I, Petsatodis G, Potoupnis M, et al. Monitoring changes in quality of life in patients with lung cancer under treatment with chemotherapy and co administration of zoledronic acid by using specialized questionnaires[J]. Journal of Cancer, 2018,9(10):1731-1736.

[494] Lee Y, Lin P Y, Lin M C, et al. Morbidity and associated factors of depressive disorder in patients with lung cancer[J]. CANCER MANAGEMENT AND RESEARCH, 2019,11:7587-7596.

[495] Lee Y, Lin P Y, Lin M C, et al. Morbidity and associated factors of depressive disorder in patients with lung cancer[J]. Cancer Management and Research, 2019,11:7587-7596.

[496] Wright F, Dunn L B, Paul S M, et al. Morning Fatigue Severity Profiles in Oncology Outpatients Receiving Chemotherapy[J]. CANCER NURSING, 2019,42(5):355-364.

[497] Menegozzo S, Comba P, Ferrante D, et al. Mortality study in an asbestos cement factory in Naples, Italy[J]. ANNALI DELL ISTITUTO SUPERIORE DI SANITA, 2011,47(3):296-304.

[498] Micke P, Botling J, Mattsson J, et al. Mucin staining is of limited value in addition to basic immunohistochemical analyses in the diagnostics of non-small cell lung cancer[J]. SCIENTIFIC REPORTS, 2019,9.

[499] Rohit K O, Baby J, Valsalan K P, et al. Mucoepidermoid Carcinoma of Lung Presenting as a Peripheral Cavitary Lesion[J]. JOURNAL OF CLINICAL AND DIAGNOSTIC RESEARCH, 2018,12(11):D3-D5.

[500] Karlsson A, Brunnstrom H, Lindquist K E, et al. Mutational and gene fusion analyses of primary large cell and large cell neuroendocrine lung cancer[J]. ONCOTARGET, 2015,6(26):22028-22037.

[501] Han Y, Kim K, Shim J Y, et al. N-acetylphytosphingosine enhances the radiosensitivity of lung cancer cell line NCI-H460[J]. MOLECULES AND CELLS, 2008,25(2):224-230.

[502] Gopalan B, Ito I, Branch C D, et al. Nanoparticle based systemic gene therapy for lung cancer: Molecular mechanisms and strategies to suppress nanoparticle-mediated inflammatory response[J]. TECHNOLOGY IN CANCER RESEARCH & TREATMENT, 2004,3(6):647-657.

[503] Lewis R J. Naphthalene animal carcinogenicity and human relevancy: Overview of industries with naphthalene-containing streams[J]. REGULATORY TOXICOLOGY AND PHARMACOLOGY, 2012,62(1):131-137.

[504] Yang Q, Yang P, Qian X H, et al. Naphthalimide intercalators with chiral amino side chains: Effects of chirality on DNA binding, photodamage and antitumor cytotoxicity[J]. BIOORGANIC & MEDICINAL CHEMISTRY LETTERS, 2008,18(23):6210-6213.

[505] Mitchell P L, Yu H, Rivalland G, et al. NaPi2b expression in a large surgical non-small cell lung cancer cohort[J]. MOLECULAR CANCER THERAPEUTICS, 2019,18(12).

[506] Heynemann S, Yu H, Churilov L, et al. NaPi2b expression in a large surgical Non-Small Cell Lung Cancer NSCLC cohort[J]. CLINICAL LUNG CANCER, 2022,23(2):E90-E98.

[507] Li S J, Wang H Y, Yang Z, et al. Naples Prognostic Score as a novel prognostic prediction tool in video-assisted thoracoscopic surgery for early-stage lung cancer: a propensity score matching study[J]. SURGICAL ENDOSCOPY AND OTHER INTERVENTIONAL TECHNIQUES, 2021,35(7):3679-3697.

[508] Chen S Q, Liu S C, Xu S W, et al. Naples Prognostic Score is an Independent Prognostic Factor in Patients with Small Cell Lung Cancer and Nomogram Predictive Model Established[J]. JOURNAL OF INFLAMMATION RESEARCH, 2022,15:3719-3731.

[509] Schmidt L A, Myers J L, McHugh J B. Napsin A Is Differentially Expressed in Sclerosing Hemangiomas of the Lung[J]. ARCHIVES OF PATHOLOGY & LABORATORY MEDICINE, 2012,136(12):1580-1584.

[510] Zhou L S, Lv X, Yang J C, et al. Napsin A is negatively associated with EMT-mediated EGFR-TKI resistance in lung cancer cells[J]. MOLECULAR MEDICINE REPORTS, 2018,18(2):1247-1252.

[511] Uchida A, Samukawa T, Kumamoto T, et al. Napsin A levels in epithelial lining fluid as a diagnostic biomarker of primary lung adenocarcinoma[J]. BMC PULMONARY MEDICINE, 2017,17.

[512] Turner B M, Cagle P T, Sainz I M, et al. Napsin A, a new marker for lung adenocarcinoma, is complementary and more sensitive and specific than thyroid transcription factor 1 in the differential diagnosis of primary pulmonary carcinoma: Evaluation of 1674 cases by tissue microarray[J]. Archives of Pathology and Laboratory Medicine, 2012,136(2):163-171.

[513] Nishino M, Hoang M P, Della Pelle P, et al. Napsin A/p40 antibody cocktail for subtyping non-small cell lung carcinoma on cytology and small biopsy specimens[J]. Cancer Cytopathology, 2016,124(7):472-484.

[514] Azulay M, Lifshits S, Fridman A, et al. Naptumomab Estafenatox induces T cell recognition, turning anti-PD-1 unresponsive “cold” tumors into “hot” responsive tumors[J]. Cancer Research, 2018,78(13).

[515] Reynolds S D, Giangreco A, Power J, et al. Neuroepithelial bodies of pulmonary airways serve as a reservoir of progenitor cells capable of epithelial regeneration[J]. AMERICAN JOURNAL OF PATHOLOGY, 2000,156(1):269-278.

[516] Singh K, Paul S M, Kober K M, et al. Neuropsychological Symptoms and Intrusive Thoughts Are Associated With Worse Trajectories of Chemotherapy-Induced Nausea[J]. JOURNAL OF PAIN AND SYMPTOM MANAGEMENT, 2020,59(3):668-678.

[517] Mikumo H, Yanagihara T, Hamada N, et al. Neutrophil elastase inhibitor sivelestat ameliorates gefitinib-naphthalene-induced acute pneumonitis in mice[J]. BIOCHEMICAL AND BIOPHYSICAL RESEARCH COMMUNICATIONS, 2017,486(1):205-209.

[518] Caponigro F, Basile M, de Rosa V, et al. New Drugs in Cancer Therapy, National Tumor Institute, Naples, 17-18 June 2004[J]. ANTI-CANCER DRUGS, 2005,16(2):211-221.

[519] Bir F, Celiker D, Evyapan B F, et al. New immunohistochemical markers in the differential diagnosis of nonsmall cell lung carcinoma[J]. TURKISH JOURNAL OF MEDICAL SCIENCES, 2016,46(6):1854-1861.

[520] Xu W T, Shen G N, Luo Y H, et al. New naphthalene derivatives induce human lung cancer A549cell apoptosis via ROS-mediated MAPKs, Akt, and STAT3 signaling pathways[J]. Chemico-Biological Interactions, 2019,304:148-157.

[521] Netto C D, Da Silva A J M, Salustiano E J S, et al. New pterocarpanquinones: Synthesis, antineoplasic activity on cultured human malignant cell lines and TNF-α modulation in human PBMC cells[J]. Bioorganic and Medicinal Chemistry, 2010,18(4):1610-1616.

[522] Cordina-Duverger E, Tvardik N, Martin D, et al. Night shift work, sleep disorders and lung cancer risk among women: Results from a population-based case-control study in france (The Welca Study)[J]. Occupational and Environmental Medicine, 2021,78(SUPPL 1):A53.

[523] Papadopoulos D, Papadoudis A, Kiagia M, et al. Nonpharmacologic Interventions for Improving Sleep Disturbances in Patients With Lung Cancer: A Systematic Review and Meta-analysis[J]. JOURNAL OF PAIN AND SYMPTOM MANAGEMENT, 2018,55(5):1364.

[524] Brasky T M, Liu J M, White E, et al. Non-steroidal anti-inflammatory drugs and cancer risk in women: Results from the Women's Health Initiative[J]. INTERNATIONAL JOURNAL OF CANCER, 2014,135(8):1869-1883.

[525] Takamochi K, Ohmiya H, Itoh M, et al. Novel biomarkers that assist in accurate discrimination of squamous cell carcinoma from adenocarcinoma of the lung[J]. BMC CANCER, 2016,16.

[526] Lim H S, Kang Y J, Sung B, et al. Novel dihydrobenzofuro[4,5-b][1,8]naphthyridin-6-one derivative, MHY-449, induces cell cycle arrest and apoptosis via the downregulation of Akt in human lung cancer cells[J]. ONCOLOGY REPORTS, 2015,34(5):2431-2438.

[527] Petsri K, Thongsom S, Racha S, et al. Novel mechanism of napabucasin, a naturally derived furanonaphthoquinone: apoptosis and autophagy induction in lung cancer cells through direct targeting on Akt/mTOR proteins[J]. BMC COMPLEMENTARY MEDICINE AND THERAPIES, 2022,22(1).

[528] Cole J, Guiot M C, Gravel M, et al. Novel NAPRT specific antibody identifies small cell lung cancer and neuronal cancers as promising clinical indications for a NAMPT inhibitor/niacin co-administration strategy[J]. ONCOTARGET, 2017,8(44):77846-77859.

[529] Yang P, Yang Q, Qian X H, et al. Novel synthetic isoquinolino[5,4-ab]phenazines: Inhibition toward topoisomerase I, antitumor and DNA photo-cleaving activities[J]. BIOORGANIC & MEDICINAL CHEMISTRY, 2005,13(21):5909-5914.

[530] Rhodes R H, Wightman H R. Nucleus of the tractus solitarius metastasis: Relationship to respiratory arrest?[J]. CANADIAN JOURNAL OF NEUROLOGICAL SCIENCES, 2000,27(4):328-332.

[531] Dean G E, Ferreira Da Rosa Silva C, Jungquist C R, et al. Nurse delivered brief behavioral therapyinsomnia for lung cancer survivors[J]. Sleep, 2017,40:A138.

[532] Dean G E, Silva C, Jungquist C R, et al. NURSE DELIVERED BRIEF BEHAVIORAL THERAPY-INSOMNIA FOR LUNG CANCER SURVIVORS[J]. SLEEP, 2017,40:A138.

[533] Dean G E, Weiss C, Jungquist C R, et al. Nurse-Delivered Brief Behavioral Treatment for Insomnia in Lung Cancer Survivors: a Pilot RCT[J]. 2020,18(6):774-786.

[534] Dean G E, Weiss C, Jungquist C R, et al. Nurse-Delivered Brief Behavioral Treatment for Insomnia in Lung Cancer Survivors: A Pilot RCT[J]. BEHAVIORAL SLEEP MEDICINE, 2020,18(6):774-786.

[535] Dean G E, Weiss C, Jungquist C R, et al. Nurse-Delivered Brief Behavioral Treatment for Insomnia in Lung Cancer Survivors: A Pilot RCT[J]. Behavioral sleep medicine, 2020,18(6):774-786.

[536] Ferreira Da Rosa Silva C, Klimpf M L, Demey Zambrano J A, et al. Nurse-delivered insomnia treatment in lung cancer survivors: pilot study to improve sleep, mood and quality of life[J]. 2015,38:A307.

[537] Domine M, Massuti B, Puente J, et al. Observational Prospective Study to Determine the Evolution of the Symptomatic Profile of Metastatic Non-Small Cell Lung Cancer (NSCLC) Patients and Its Relation to the Control of the Disease[J]. ADVANCES IN THERAPY, 2019,36(6):1497-1508.

[538] Cheng L, Guo H, Zhang Z, et al. Obstructive sleep apnea and incidence of malignant tumors: a meta-analysis[J]. Sleep Med, 2021,84:195-204.

[539] Kendzerska T, Povitz M, Leung R S, et al. Obstructive sleep apnea and incident cancer: A large retrospective multicenter clinical cohort study[J]. Cancer Epidemiology Biomarkers and Prevention, 2021,30(2):295-304.

[540] Cheong A, Tan B, Teo Y H, et al. Obstructive Sleep Apnea and Lung Cancer: A Systematic Review and Meta-Analysis[J]. Ann Am Thorac Soc, 2022,19(3):469-475.

[541] Seijo L M, Pérez-Warnisher M T, Giraldo-Cadavid L F, et al. Obstructive sleep apnea and nocturnal hypoxemia are associated with an increased risk of lung cancer[J]. Sleep Med, 2019,63:41-45.

[542] Chen M X, Chen L D, Zeng A M, et al. Obstructive sleep apnea and the risk of mortality in patients with lung cancer: a meta-analysis[J]. Sleep Breath, 2022,26(2):559-566.

[543] Chen M X, Chen L D, Zeng A M, et al. Obstructive sleep apnea and the risk of mortality in patients with lung cancer: a meta-analysis[J]. SLEEP AND BREATHING, 2022,26(2):559-566.

[544] Saber A N, Zhang H F, Islam A, et al. Occurrence, fates, and carcinogenic risks of substituted polycyclic aromatic hydrocarbons in two coking wastewater treatment systems[J]. SCIENCE OF THE TOTAL ENVIRONMENT, 2021,789.

[545] Hickok J T, Morrow G R, Roscoe J A, et al. Occurrence, severity, and longitudinal course of twelve common symptoms in 1129 consecutive patients during radiotherapy for cancer[J]. JOURNAL OF PAIN AND SYMPTOM MANAGEMENT, 2005,30(5):433-442.

[546] Pistevou-Gombaki K, Eleftheriadis N, Plataniotis G A, et al. Octreotide for palliative treatment of hepatic metastases from non-neuroendocrine primary tumours: evaluation of quality of life using the EORTC QLQ-C30 questionnaire[J]. Palliat Med, 2003,17(3):257-262.

[547] Pistevou-Gombaki K, Eleftheriadis N, Plataniotis G A, et al. Octreotide for palliative treatment of hepatic metastases from non-neuroendocrine primary tumours: evaluation of quality of life using the EORTC QLQ-C30 questionnaire[J]. PALLIATIVE MEDICINE, 2003,17(3):257-262.

[548] Tolbert P E. Oils and cancer[J]. CANCER CAUSES & CONTROL, 1997,8(3):386-405.

[549] Wang W, Lou G, Zhang Y. Olanzapine with ondansetron and dexamethasone for the prevention of cisplatin-based chemotherapy-induced nausea and vomiting in lung cancer[J]. Medicine (Baltimore), 2018,97(37):e12331.

[550] Wang W X, Lou G Y, Zhang Y P. Olanzapine with ondansetron and dexamethasone for the prevention of cisplatin-based chemotherapy-induced nausea and vomiting in lung cancer[J]. MEDICINE, 2018,97(37).

[551] Targhazeh N, Reiter R J, Rahimi M, et al. Oncostatic activities of melatonin: Roles in cell cycle, apoptosis, and autophagy[J]. BIOCHIMIE, 2022,202:34-48.

[552] Fang L T, Mohiyuddin M, Fu Y, et al. OnkoInsight: An end-to-end cancer informatics pipeline to generate insights from large sequencing datasets[J]. Cancer Research, 2017,77(13).

[553] Gaugg M T. On-line Breath Metabolomics in Respiratory Diseases using Secondary Electrospray Ionization-Mass Spectrometry[J]. CHIMIA, 2018,72(4):184-188.

[554] Tuminello S, Wisnivesky J, Schwartz R, et al. Opioids and Sleep Medication Use After Surgery for Early Stage Lung Cancer: A SEER-Medicare Analysis[J]. JOURNAL OF THORACIC ONCOLOGY, 2018,13(10):S355.

[555] Salminen E K, Silvoniemi M, Syrjänen K, et al. Opioids in pain management of mesothelioma and lung cancer patients[J]. Acta Oncologica, 2013,52(1):30-37.

[556] Salminen E K, Silvoniemi M, Syrjanen K, et al. Opioids in pain management of mesothelioma and lung cancer patients[J]. ACTA ONCOLOGICA, 2013,52(1):30-37.

[557] Salminen E K, Silvoniemi M, Syrjanen K, et al. Opioids in pain management of mesothelioma and lung cancer patients[J]. 2013,52(1):30-37.

[558] Troiano V, Scarbaci K, Ettari R, et al. Optimization of peptidomimetic boronates bearing a P3 bicyclic scaffold as proteasome inhibitors[J]. EUROPEAN JOURNAL OF MEDICINAL CHEMISTRY, 2014,83:1-14.

[559] Vela M, Zulet A I, Lopez V R, et al. OSAS and cancer: Is there a relation?[J]. EUROPEAN RESPIRATORY JOURNAL, 2013,42.

[560] Blayney D, Mitchell D, Lelorier Y, et al. P1.01-11 Quality of Life in NSCLC Patients Treated with Docetaxel and Either Plinabulin or Pegfilgrastim for Prevention of Neutropenia[J]. Journal of Thoracic Oncology, 2019,14(10):S359.

[561] Culea M, Cozar O, Culea E. PAHs in cigarette smoke by gas chromatography-mass spectrometry[J]. INDOOR AND BUILT ENVIRONMENT, 2005,14(3-4):283-292.

[562] Sheu H L, Lee W J, Tsai J H, et al. Particle size distribution of polycyclic aromatic hydrocarbons in the ambient air of a traffic intersection[J]. JOURNAL OF ENVIRONMENTAL SCIENCE AND HEALTH PART A-ENVIRONMENTAL SCIENCE AND ENGINEERING & TOXIC AND HAZARDOUS SUBSTANCE CONTROL, 1996,31(6):1293-1316.

[563] Gu Z B, Liao L M, Yao G J, et al. Patient with EGFR-mutant lung cancer harboring de novo MET amplification successfully treated with gefitinib combined with crizotinib[J]. CURRENT PROBLEMS IN CANCER, 2021,45(5).

[564] Mazieres J, Iadeluca L, Shaw A T, et al. Patient-reported outcomes from the randomized phase 3 CROWN study of first-line lorlatinib versus crizotinib in advanced ALK-positive non-small cell lung cancer[J]. Lung Cancer, 2022,174:146-156.

[565] Félix Soares R, Silva R, Rodrigues É, et al. Patient-reported outcomes of early integration palliative care in quality of life and symptom burden in advanced lung cancer – A randomized study[J]. Annals of Oncology, 2020,31:S935.

[566] Wei X, Yu H F, Dai W, et al. Patient-Reported Outcomes of Video-Assisted Thoracoscopic Surgery Versus Thoracotomy for Locally Advanced Lung Cancer: A Longitudinal Cohort Study[J]. ANNALS OF SURGICAL ONCOLOGY, 2021,28(13):8358-8371.

[567] Somayaji D, Mohedat H, Dean G E, et al. Patients' Perceptions at Diagnosis: Lung Cancer Discovery and Provider Relationships[J]. CANCER NURSING, 2022,45(5):397-405.

[568] Yvorel V, Patoir A, Casteillo F, et al. PD-L1 expression in pleomorphic, spindle cell and giant cell carcinoma of the lung is related to TTF-1, p40 expression and might indicate a worse prognosis[J]. PLOS ONE, 2017,12(7).

[569] Patil R, Limaye S, Akolkar D, et al. PD-L1 profiling of circulating tumour cells is a viable companion diagnostic for checkpoint inhibitor therapy in lung cancer[J]. Annals of Oncology, 2019,30:i3.

[570] Shi G, Liu Q S, Chen H, et al. Percutaneous osteoplasty for the management of a humeral head metastasis Two case reports[J]. MEDICINE, 2019,98(20).

[571] Abse D W, Wilkins M M, Van De Castle R L. Personality and behavioral characteristics of lung cancer patients[J]. Journal of Psychosomatic Research, 1974,18(2):101-113.

[572] Soufi G, Bagheri H, Rad L Y, et al. Perylene diimide-POSS network for semi selective solid-phase microextraction of lung cancer biomarkers in exhaled breath[J]. ANALYTICA CHIMICA ACTA, 2022,1198.

[573] Yana T, Negoro S, Takada M, et al. Phase II study of amrubicin in previously untreated patients with extensive-disease small cell lung cancer: West Japan Thoracic Oncology Group (WJTOG) study[J]. INVESTIGATIONAL NEW DRUGS, 2007,25(3):253-258.

[574] Gerber D E, Swanson P, Lopez-Chavez A, et al. Phase II study of olaratumab with paclitaxel/carboplatin (P/C) or P/C alone in previously untreated advanced NSCLC[J]. Lung Cancer, 2017,111:108-115.

[575] Kobayashi H, Aoki N, Niki Y, et al. Phase III double-blind comparative study of BAY 12-8039 (moxifloxacin) versus levofloxacin in patients with community-acquired pneumonia[J]. Japanese Journal of Chemotherapy, 2005,53(SUPPL. 3):27-46.

[576] Makhanya T R, Gengan R M, Pandian P, et al. Phosphotungstic Acid Catalyzed One Pot Synthesis of 4,8,8-Trimethyl-5-phenyl-5,5a,8,9-tetrahydrobenzo[b] [1,8]Naphthyridin-6(7H)-one Derivatives and Their Biological Evaluation Against A549 Lung Cancer Cells[J]. JOURNAL OF HETEROCYCLIC CHEMISTRY, 2018,55(5):1193-1204.

[577] Avancini A, Sartori G, Gkountakos A, et al. Physical Activity and Exercise in Lung Cancer Care: Will Promises Be Fulfilled?[J]. Oncologist, 2020,25(3):e555-e569.

[578] Avancini A, Sartori G, Gkountakos A, et al. Physical Activity and Exercise in Lung Cancer Care: Will Promises Be Fulfilled?[J]. ONCOLOGIST, 2020,25(3):E555-E569.

[579] Ghilotti F, Pesonen A S, Raposo S E, et al. Physical activity, sleep and risk of respiratory infections: A Swedish cohort study[J]. PLOS ONE, 2018,13(1).

[580] Ghilotti F, Pesonen A S, Raposo S E, et al. Physical activity, sleep and risk of respiratory infections: A Swedish cohort study[J]. PLoS ONE, 2018,13(1).

[581] Given B, Given C, Azzouz F, et al. Physical functioning of elderly cancer patients prior to diagnosis and following initial treatment.[J]. Nursing research, 2001,50(4):222-232.

[582] Panda M, Tripathi S K, Biswal B K. Plumbagin promotes mitochondrial mediated apoptosis in gefitinib sensitive and resistant A549 lung cancer cell line through enhancing reactive oxygen species generation[J]. MOLECULAR BIOLOGY REPORTS, 2020,47(6):4155-4168.

[583] Sun S B, Zhang Y, Xu W P, et al. Plumbagin reduction by thioredoxin reductase 1 possesses synergy effects with GLUT1 inhibitor on KEAP1-mutant NSCLC cells[J]. BIOMEDICINE & PHARMACOTHERAPY, 2022,146.

[584] Elovaara E, Mikkola J, Stockmann-Juvala H, et al. Polycyclic aromatic hydrocarbon (PAH) metabolizing enzyme activities in human lung, and their inducibility by exposure to naphthalene, phenanthrene, pyrene, chrysene, and benzo(a)pyrene as shown in the rat lung and liver[J]. ARCHIVES OF TOXICOLOGY, 2007,81(3):169-182.

[585] Carbone M, Shimizu D, Napolitano A, et al. Positive nuclear BAP1 immunostaining helps differentiate nonsmall cell lung carcinomas from malignant mesothelioma[J]. Oncotarget, 2016,7(37):59314-59321.

[586] Onishi H, Onose M, Yamada T, et al. Post-traumatic stress disorder associated with suspected lung cancer and bereavement: 4-year follow-up and review of the literature[J]. SUPPORTIVE CARE IN CANCER, 2003,11(2):123-125.

[587] Rai S, Tanaka H, Espinoza J L, et al. Potent efficacy of chlorpromazine in acute myeloid leukemia harboring KIT-D816V mutation[J]. Leukemia Research Reports, 2021,15.

[588] Xing C, Wang Y, Fujioka N, et al. Potential of kava in reducing lung cancer risk, tobacco use, and associated disparities[J]. Cancer Research, 2019,79(13).

[589] Relja B, Omid N, Schaible A, et al. Pre- or post-treatment with ethanol and ethyl pyruvate results in distinct anti-inflammatory responses of human lung epithelial cells triggered by interleukin-6[J]. Molecular medicine reports, 2015,12(2):2991-2998.

[590] Magnusson K, Turkiewicz A, Timpka S, et al. Prediction of midlife hand osteoarthritis in young men[J]. OSTEOARTHRITIS AND CARTILAGE, 2018,26(8):1027-1032.

[591] Kozachik S L, Bandeen-Roche K. Predictors of patterns of pain, fatigue, and insomnia during the first year after a cancer diagnosis in the elderly[J]. CANCER NURSING, 2008,31(5):334-344.

[592] Kozachik S L, Bandeen-Roche K. Predictors of patterns of pain, fatigue, and insomnia during the first year after a cancer diagnosis in the elderly[J]. Cancer Nursing, 2008,31(5):334-344.

[593] Kim H S, Oh E G, Lee H, et al. Predictors of symptom experience in Korean patients with cancer undergoing chemotherapy[J]. European journal of oncology nursing : the official journal of European Oncology Nursing Society, 2015,19(6):644-653.

[594] Kim H S, Oh E G, Lee H, et al. Predictors of symptom experience in Korean patients with cancer undergoing chemotherapy[J]. EUROPEAN JOURNAL OF ONCOLOGY NURSING, 2015,19(6):644-653.

[595] Viale M, Mariggo M A, Ottone M, et al. Preliminary evaluation in vitro of the inhibition of cell proliferation, cytotoxicity and induction of apoptosis by 1,4-bis(1-naphthyl)-2,3-dinitro-1,3-butadiene[J]. INVESTIGATIONAL NEW DRUGS, 2004,22(4):359-367.

[596] Skaug K, Eide G E, Gulsvik A. Prevalence and predictors of symptoms in the terminal stage of lung cancer: A community study[J]. Chest, 2007,131(2):389-394.

[597] Skaug K, Eide G E, Gulsvik A. Prevalence and predictors of symptoms in the terminal stage of lung cancer: A community study[J]. Chest, 2007,131(2):389-394.

[598] Steinberg T, Roseman M, Kasymjanova G, et al. Prevalence of emotional distress in newly diagnosed lung cancer patients[J]. Supportive Care in Cancer, 2009,17(12):1493-1497.

[599] Tokoz G, Yalug I, Ozdemir S, et al. Prevalence of major depression in patients with cancer and related factors[J]. ANADOLU PSIKIYATRI DERGISI-ANATOLIAN JOURNAL OF PSYCHIATRY, 2008,9(2):59-66.

[600] Seijo L, Perez-Warnisher M, Cabezas E, et al. Prevalence of obstructive sleep apnea among volunteers enrolled in a lung cancer screening program. Results of the prospective sails (sleep apnea in lung cancer screening) study[J]. American Journal of Respiratory and Critical Care Medicine, 2017,195.

[601] NCT. Preventive Skin Analgesia With Lidocaine Patch 5% for Controlling Post-thoracotomy Pain[J]. 2016.

[602] Hatanaka K, Tsuta K, Watanabe K, et al. Primary pulmonary adenocarcinoma with enteric differentiation resembling metastatic colorectal carcinoma: A report of the second case negative for cytokeratin 7[J]. PATHOLOGY RESEARCH AND PRACTICE, 2011,207(3):188-191.

[603] Sharma S, Tayal A, Khatri S, et al. Primary pulmonary epithelial-myoepithelial carcinoma: Report of a rare and under-diagnosed low-grade malignancy[J]. JOURNAL OF CANCER RESEARCH AND THERAPEUTICS, 2022,18(3):795-800.

[604] Li S L, Hou L K, Huang Y, et al. Primary salivary duct carcinoma of the lung: clinicopathological features, diagnosis and practical challenges[J]. JOURNAL OF CLINICAL PATHOLOGY.

[605] Xuan J M, Peng J H, Wang S, et al. Prognostic significance of Naples prognostic score in non-small-cell lung cancer patients with brain metastases[J]. FUTURE ONCOLOGY, 2022,18(13):1545-1555.

[606] Wang X Q, Ma X J, Yang M, et al. Proportion and related factors of depression and anxiety for inpatients with lung cancer in China: a hospital-based cross-sectional study[J]. SUPPORTIVE CARE IN CANCER, 2022,30(6):5539-5549.

[607] Wang X, Ma X, Yang M, et al. Proportion and related factors of depression and anxiety for inpatients with lung cancer in China: a hospital-based cross-sectional study[J]. Supportive Care in Cancer, 2022,30(6):5539-5549.

[608] Wang X S, Shi Q L, Williams L A, et al. Prospective Study of Patient-Reported Symptom Burden in Patients With Non-Small-Cell Lung Cancer Undergoing Proton or Photon Chemoradiation Therapy[J]. JOURNAL OF PAIN AND SYMPTOM MANAGEMENT, 2016,51(5):832-838.

[609] Lin S Y, Tsai S J, Wang L H, et al. Protection by quercetin against cooking oil fumes-induced DNA damage in human lung adenocarcinoma CL-3 cells: Role of COX-2[J]. NUTRITION AND CANCER-AN INTERNATIONAL JOURNAL, 2002,44(1):95-101.

[610] Nishimura T, Nomura M, Tojo H, et al. Proteomic analysis of laser-microdissected paraffin-embedded tissues: (2) MRM assay for stage-related proteins upon non-metastatic lung adenocarcinoma[J]. JOURNAL OF PROTEOMICS, 2010,73(6):1100-1110.

[611] Rossi G, Caroli G, Caruso D, et al. Pseudocarcinomatous Mesothelioma: A Hitherto Unreported Presentation closely simulating primary lung cancer[J]. INTERNATIONAL JOURNAL OF SURGICAL PATHOLOGY, 2021,29(7):775-779.

[612] Papadopoulos D, Kiagia M, Charpidou A, et al. Psychological correlates of sleep quality in lung cancer patients under chemotherapy: A single-center cross-sectional study[J]. PSYCHO-ONCOLOGY, 2019,28(9):1879-1886.

[613] Papadopoulos D, Kiagia M, Charpidou A, et al. Psychological correlates of sleep quality in lung cancer patients under chemotherapy: A single-center cross-sectional study[J]. Psycho-Oncology, 2019,28(9):1879-1886.

[614] Linares-Moya M, Rodriguez-Torres J, Heredia-Ciuro A, et al. Psychological distress prior to surgery is related to symptom burden and health status in lung cancer survivors[J]. SUPPORTIVE CARE IN CANCER, 2022,30(2):1579-1586.

[615] Rasmussen J F, Siersma V, Pedersen J H, et al. Psychosocial consequences in the Danish randomised controlled lung cancer screening trial (DLCST)[J]. LUNG CANCER, 2015,87(1):65-72.

[616] Cigarral Garcia C, Antona Casas C. Psychosocial distress among patients with lung cancer: An approach to assessment and intervention[J]. Journal of Thoracic Oncology, 2012,7(9):S335.

[617] Lazaro S, Lorz C, Enguita A B, et al. Pten and p53 Loss in the Mouse Lung Causes Adenocarcinoma and Sarcomatoid Carcinoma[J]. CANCERS, 2022,14(15).

[618] Lenner R, Padilla M L, Teirstein A S, et al. Pulmonary complications in cardiac transplant recipients[J]. CHEST, 2001,120(2):508-513.

[619] Wang C X, Liu B, Wang Y F, et al. Pulmonary enteric adenocarcinoma: a study of the clinicopathologic and molecular status of nine cases[J]. INTERNATIONAL JOURNAL OF CLINICAL AND EXPERIMENTAL PATHOLOGY, 2014,7(3):1266-1274.

[620] Braun D P, Gupta D, Staren E D. Quality of life assessment as a predictor of survival in non-small cell lung cancer[J]. BMC CANCER, 2011,11.

[621] Billingham L J, Gaunt P, Jarrett H W, et al. Quality of life in advanced non-small cell lung cancer, effects of cisplatin dose and carboplatin in combination with gemcitabine: Results: from BTOG2, a British thoracic oncology group phase III trial in 1363 patients[J]. Thorax, 2011,66:A41.

[622] Svedlund J, Sullivan M, Sjödin I, et al. Quality of life in gastric cancer prior to gastrectomy[J]. Quality of Life Research, 1996,5(2):255-264.

[623] Svedlund J, Sullivan M, Sjodin I, et al. Quality of life in gastric cancer prior to gastrectomy[J]. QUALITY OF LIFE RESEARCH, 1996,5(2):255-264.

[624] Hechtner M, Eichler M, Wehler B, et al. Quality of Life in NSCLC Survivors - A Multicenter Cross-Sectional Study[J]. JOURNAL OF THORACIC ONCOLOGY, 2019,14(3):420-435.

[625] Rodriguez A G, Gregorio M, Rodriguez A M, et al. Quality of life in transplant patients, compared to other stressful health situations in pulmonary patients[J]. PSICOTHEMA, 2008,20(2):266-272.

[626] Si X, Zhang L, Wang H, et al. Quality of life results from a randomized, double-blinded, placebo-controlled, multi-center phase III trial of anlotinib in patients with advanced non-small cell lung cancer[J]. Lung Cancer, 2018,122:32-37.

[627] Si X Y, Zhang L, Wang H P, et al. Quality of life results from a randomized, double-blinded, placebo-controlled, multi-center phase III trial of anlotinib in patients with advanced non-small cell lung cancer[J]. LUNG CANCER, 2018,122:32-37.

[628] Gonzalez-Ling A, Vazquez O G, Bello M E, et al. Quality of life, anxiety, depression, and distress in patients with advanced and metastatic lung cancer[J]. PALLIATIVE & SUPPORTIVE CARE.

[629] Ying H Z, Yu C H, Chen H K, et al. Quinonoids: Therapeutic Potential for Lung Cancer Treatment[J]. BIOMED RESEARCH INTERNATIONAL, 2020,2020.

[630] Jereczek-Fossa B A, Marsiglia H R, Orecchia R. Radiotherapy-related fatigue[J]. CRITICAL REVIEWS IN ONCOLOGY HEMATOLOGY, 2002,41(3):317-325.

[631] Cammarota B, Cascone M T, De Paola L, et al. Radon risk in healthcare facilities: Environmental monitoring and effective dose[J]. Medicina del Lavoro, 2009,100(5):375-383.

[632] Garon E B, Ciuleanu T E, Arrieta O, et al. Ramucirumab plus docetaxel versus placebo plus docetaxel for second-line treatment of stage IV non-small-cell lung cancer after disease progression on platinum-based therapy (REVEL): A multicentre, double-blind, randomised phase 3 trial[J]. The Lancet, 2014,384(9944):665-673.

[633] Tsukada H, Hirose T, Yokoyama A, et al. Randomised comparison of ondansetron plus dexamethasone with dexamethasone alone for the control of delayed cisplatin-induced emesis[J]. European Journal of Cancer, 2001,37(18):2398-2404.

[634] Goswami S, Das A K, Saha U, et al. Rapid detection of hydrazine in a naphthol-fused chromenyl loop and its effectiveness in human lung cancer cells: tuning remarkable selectivity via the reaction altered pathway supported by theoretical studies[J]. ORGANIC & BIOMOLECULAR CHEMISTRY, 2015,13(7):2134-2139.

[635] Chen J B, Kong X F, Lv Y Y, et al. 'Real world survey' of hydrogen-controlled cancer: A follow-up report of 82 advanced cancer patients[J]. Medical Gas Research, 2019,9(3):115-121.

[636] Tong Y, Yue J, Mao M, et al. Recombinant nematode anticoagulant protein c2 inhibits cell invasion by decreasing uPA expression in NSCLC cells[J]. ONCOLOGY REPORTS, 2015,33(4):1815-1822.

[637] Wang T, Du M X, Ji Z Y, et al. Recombinant protein rMBP-NAP restricts tumor progression by triggering antitumor immunity in mouse metastatic lung cancer[J]. CANADIAN JOURNAL OF PHYSIOLOGY AND PHARMACOLOGY, 2018,96(2):113-119.

[638] Sant M, Minicozzi P, Allemani C, et al. Regional inequalities in cancer care persist in Italy and can influence survival[J]. CANCER EPIDEMIOLOGY, 2012,36(6):541-547.

[639] Liu W, Luo M, Fang Y Y, et al. Relationship between Occurrence and Progression of Lung Cancer and Nocturnal Intermittent Hypoxia, Apnea and Daytime Sleepiness[J]. CURRENT MEDICAL SCIENCE, 2019,39(4):568-575.

[640] Wang Y, Cheng X J, Yin A H, et al. Relationship between sleep disorders and lymphocyte subsets and cytokines in patients with lung cancer[J]. Journal of Biological Regulators and Homeostatic Agents, 2018,32(5):1231-1237.

[641] Wang Y, Cheng X J, Yin A H, et al. RELATIONSHIP BETWEEN SLEEP DISORDERS AND LYMPHOCYTE SUBSETS AND CYTOKINES IN PATIENTS WITH LUNG CANCER[J]. JOURNAL OF BIOLOGICAL REGULATORS AND HOMEOSTATIC AGENTS, 2018,32(5):1231-1237.

[642] Franceschini J, Jardim J R, Fernandes A, et al. Relationship between the magnitude of symptoms and the quality of life: a cluster analysis of lung cancer patients in Brazil[J]. JORNAL BRASILEIRO DE PNEUMOLOGIA, 2013,39(1):23-31.

[643] Franceschini J, Jardim J R, Fernandes A L G, et al. Relationship between the magnitude of symptoms and the quality of life: A cluster analysis of lung cancer patients in Brazil[J]. Jornal Brasileiro de Pneumologia, 2013,39(1):23-31.

[644] Takemura N, Cheung D, Fong D, et al. Relationship of subjective and objective sleep measures with physical performance in advanced-stage lung cancer patients[J]. SCIENTIFIC REPORTS, 2021,11(1).

[645] Takemura N, Cheung D, Fong D, et al. Relationship of subjective and objective sleep measures with physical performance in advanced-stage lung cancer patients[J]. 2021,11(1):17208.

[646] Takemura N, Cheung D S T, Fong D Y T, et al. Relationship of subjective and objective sleep measures with physical performance in advanced-stage lung cancer patients[J]. Scientific reports, 2021,11(1):17208.

[647] Kao C H, Sun L M, Liang J A, et al. Relationship of Zolpidem and Cancer Risk: A Taiwanese Population-Based Cohort Study[J]. MAYO CLINIC PROCEEDINGS, 2012,87(5):430-436.

[648] Hoffman A J, Given B A, von Eye A, et al. Relationships among pain, fatigue, insomnia, and gender in persons with lung cancer[J]. ONCOLOGY NURSING FORUM, 2007,34(4):785-792.

[649] Hoffman A J, Given B A, Von Eye A, et al. Relationships among pain, fatigue, insomnia, and gender in persons with lung cancer[J]. Oncology Nursing Forum, 2007,34(4):785-792.

[650] Xie J X, Zhu M, Ji M M, et al. Relationships between sleep traits and lung cancer risk: a prospective cohort study in UK Biobank[J]. SLEEP, 2021,44(9).

[651] Xie J, Zhu M, Ji M, et al. Relationships between sleep traits and lung cancer risk: A prospective cohort study in UK Biobank[J]. Sleep, 2021,44(9).

[652] Chen H M, Wu Y C, Tsai C M, et al. Relationships of circadian rhythms and physical activity with objective sleep parameters in Lung cancer patients[J]. Cancer Nursing, 2015,38(3):215-223.

[653] Chen H M, Wu Y C, Tsai C M, et al. Relationships of Circadian Rhythms and Physical Activity With Objective Sleep Parameters in Lung Cancer Patients[J]. CANCER NURSING, 2015,38(3):215-223.

[654] Chen H M, Cheung D, Lin Y Y, et al. Relationships of exercise timing with sleep, fatigue and rest-activity rhythms of lung cancer patients in Taiwan: an exploratory study[J]. 2020,29(4):e13233.

[655] Chen H M, Cheung D, Lin Y Y, et al. Relationships of exercise timing with sleep, fatigue and rest-activity rhythms of lung cancer patients in Taiwan: An exploratory study[J]. EUROPEAN JOURNAL OF CANCER CARE, 2020,29(4).

[656] Chen H M, Cheung D S T, Lin Y Y, et al. Relationships of exercise timing with sleep, fatigue and rest-activity rhythms of lung cancer patients in Taiwan: An exploratory study[J]. European journal of cancer care, 2020,29(4):e13233.

[657] Chang W P, Lin C C. Relationships of salivary cortisol and melatonin rhythms to sleep quality, emotion, and fatigue levels in patients with newly diagnosed lung cancer[J]. EUROPEAN JOURNAL OF ONCOLOGY NURSING, 2017,29:79-84.

[658] Chang W P, Lin C C. Relationships of salivary cortisol and melatonin rhythms to sleep quality, emotion, and fatigue levels in patients with newly diagnosed lung cancer[J]. European journal of oncology nursing : the official journal of European Oncology Nursing Society, 2017,29:79-84.

[659] Ursavaş A, Karadag M, Burgazlioglu B, et al. Relief from sleep apnea after radiation and chemotherapy[J]. Clinical Lung Cancer, 2007,8(8):502-503.

[660] Ursavas A, Karadag M, Burgazlioglu B, et al. Relief from sleep apnea after radiation and chemotherapy[J]. CLINICAL LUNG CANCER, 2007,8(8):502-503.

[661] Morishita K, Yakushiji N, Ohsawa F, et al. Replacing alkyl sulfonamide with aromatic sulfonamide in sulfonamide-type RXR agonists favors switch towards antagonist activity[J]. BIOORGANIC & MEDICINAL CHEMISTRY LETTERS, 2009,19(3):1001-1003.

[662] Rippon I, Lewison G, Partridge M R. Research outputs in respiratory medicine[J]. THORAX, 2005,60(1):63-67.

[663] Ghanem M M, Porter D, Battelli L A, et al. Respirable coal dust particles modify cytochrome P4501A1 (CYP1A1) expression in rat alveolar cells[J]. AMERICAN JOURNAL OF RESPIRATORY CELL AND MOLECULAR BIOLOGY, 2004,31(2):171-183.

[664] Lou V, Chen E J, Jian H, et al. Respiratory Symptoms, Sleep, and Quality of Life in Patients With Advanced Lung Cancer[J]. JOURNAL OF PAIN AND SYMPTOM MANAGEMENT, 2017,53(2):250.

[665] Peng W, Zhang H, Li Z. Responses of lung cancer survivors undergoing gamma knife surgery to supportive group psychotherapy[J]. Medicine (Baltimore), 2019,98(9):e14693.

[666] Peng W, Zhang H, Li Z. Responses of lung cancer survivors undergoing gamma knife surgery to supportive group psychotherapy[J]. Medicine (United States), 2019,98(9).

[667] Peng W X, Zhang H, Li Z G. Responses of lung cancer survivors undergoing gamma knife surgery to supportive group psychotherapy[J]. MEDICINE, 2019,98(9).

[668] Kim D W, Ahn M J, Shi Y, et al. Results of a global phase II study with crizotinib in advanced ALK-positive non-small-cell lung cancer (NSCLC)[J]. Annals of Oncology, 2012,23:i32-i33.

[669] Luo H M, Yang A M, Schulte B A, et al. Resveratrol Induces Premature Senescence in Lung Cancer Cells via ROS-Mediated DNA Damage[J]. PLOS ONE, 2013,8(3).

[670] Chen Y, Liu Y, Yao Y C, et al. Reverse micelle-based water-soluble nanoparticles for simultaneous bioimaging and drug delivery[J]. ORGANIC & BIOMOLECULAR CHEMISTRY, 2017,15(15):3232-3238.

[671] Wang W, Huang M J, Chan C Y, et al. Risk assessment of non-dietary exposure to polycyclic aromatic hydrocarbons (PAHs) via house PM2.5, TSP and dust and the implications from human hair[J]. ATMOSPHERIC ENVIRONMENT, 2013,73:204-213.

[672] Featherstone I, Sheldon T, Johnson M, et al. Risk factors for delirium in adult patients receiving specialist palliative care: A systematic review and meta-analysis[J]. PALLIATIVE MEDICINE, 2022,36(2):254-267.

[673] Zhang L, Li X, Wu X, et al. Risk Factors for depression and efficacy of comprehensive care in advanced lung cancer patients with chemotherapy[J]. International Journal of Clinical and Experimental Medicine, 2018,11(5):5062-5070.

[674] Gu F, He Y, Mao Y, et al. Risk factors for nicotine dependence in Chinese patients with lung cancer[J]. Journal of International Medical Research, 2019,47(1):391-397.

[675] Gu F, He Y Y, Mao Y J, et al. Risk factors for nicotine dependence in Chinese patients with lung cancer[J]. JOURNAL OF INTERNATIONAL MEDICAL RESEARCH, 2019,47(1):391-397.

[676] Ni J J, Zheng Z Q, Li J, et al. Risk factors of postoperative recurrence and potential candidate of adjuvant radiotherapy in lung adenosquamous carcinoma[J]. JOURNAL OF THORACIC DISEASE, 2020,12(10):5593.

[677] Tomioka K, Saeki K, Obayashi K, et al. Risk of Lung Cancer in Workers Exposed to Benzidine and/or Beta-Naphthylamine: A Systematic Review and Meta-Analysis[J]. JOURNAL OF EPIDEMIOLOGY, 2016,26(9):447-458.

[678] Heffner J E, Holgate S T, Chung K F, et al. Road ahead to respiratory health: Experts chart future research directions[J]. RESPIROLOGY, 2009,14(5):625-636.

[679] Mohammed Salama M E. Role of Napsin A and Survivin Immunohistochemical Expression in Bronchogenic Adenocarcinoma[J]. Asian Pacific journal of cancer prevention : APJCP, 2020,21(11):3345-3348.

[680] Shah D, Joshi M, Patel B M. Role of NIMA-related kinase 2 in lung cancer: Mechanisms and therapeutic prospects[J]. FUNDAMENTAL & CLINICAL PHARMACOLOGY, 2022,36(5):766-776.

[681] Ogawa H, Tanaka Y, Tachihara M, et al. ROS1-rearranged high-PD-L1-expressing lung adenocarcinoma manifesting as mediastinal tumor: A case report[J]. ONCOLOGY LETTERS, 2019,17(1):488-491.

[682] Zhao T, Jin W L, Pan H, et al. Rosbin, a synthetic small molecule, induces A549 cells apoptosis through a ROS-mediated pathway[J]. CELL BIOLOGY INTERNATIONAL, 2017,41(2):221-226.

[683] Teixeira T M, Arraes I G, Abreu D C, et al. Ruthenium complexes show promise when submitted to toxicological safety tests using alternative methodologies[J]. EUROPEAN JOURNAL OF MEDICINAL CHEMISTRY, 2021,216.

[684] Wang R, Li Y G, Hu E Z, et al. S100A7 promotes lung adenocarcinoma to squamous carcinoma transdifferentiation, and its expression is differentially regulated by the Hippo-YAP pathway in lung cancer cells[J]. ONCOTARGET, 2017,8(15):24804-24814.

[685] Shrestha J P, Chang C. Safe and easy route for the synthesis of 1,3-dimethyl-1,2,3-triazolium salt and investigation of its anticancer activities[J]. BIOORGANIC & MEDICINAL CHEMISTRY LETTERS, 2013,23(21):5909-5911.

[686] Werner T L, Kannapel E, Chen J, et al. Safety and PK results from a phase Ib study of AL3818 (anlotinib) hydrochloride in subjects with ovarian, cervical, and endometrial cancers[J]. Journal of Clinical Oncology, 2017,35(15).

[687] Nijakowski K, Surdacki M, Sobieszczanska M. Salivary Melatonin Changes in Oncological Patients: A Systematic Review[J]. METABOLITES, 2022,12(5).

[688] Matsukuma S, Obara K, Kono T, et al. Sebaceous-like clear cell changes in primary lung cancer[J]. THORACIC CANCER, 2013,4(4):469-473.

[689] Gridelli C, Ardizzoni A, Ciardiello F, et al. Second-line treatment of advanced non-small cell lung cancer[J]. JOURNAL OF THORACIC ONCOLOGY, 2008,3(4):430-440.

[690] Dong Y L, Li Y W, Liu R W, et al. Secretagogin, a marker for neuroendocrine cells, is more sensitive and specific in large cell neuroendocrine carcinoma compared with the markers CD56, CgA, Syn and Napsin A[J]. ONCOLOGY LETTERS, 2020,19(3):2223-2230.

[691] Zhang L S, Yan L X, Gao S, et al. Self-assembling peptide-etoposide nanofibers for overcoming multidrug resistance[J]. Chemical Communications, 2020,56(97):15321-15324.

[692] Zhang L S, Yan L X, Gao S, et al. Self-assembling peptide-etoposide nanofibers for overcoming multidrug resistance[J]. CHEMICAL COMMUNICATIONS, 2020,56(97):15321-15324.

[693] Abse D W, Wilkins M M, Kirschner G, et al. Self-frustration, nighttime smoking and lung cancer[J]. Psychosom Med, 1972,34(5):395-404.

[694] Gottfried T, Kamer I, Salant I, et al. Self-reported sleep quality as prognostic for survival in lung cancer patients[J]. CANCER MANAGEMENT AND RESEARCH, 2020,12:313-321.

[695] Sodhi A, Pisani M, Glassberg M K, et al. Sex and Gender in Lung Disease and Sleep Disorders: A State-of-the-Art Review[J]. Chest, 2022,162(3):647-658.

[696] Tang J C, Ren Y G, Zhao J, et al. Shikonin enhances sensitization of gefitinib against wild-type EGFR non-small cell lung cancer via inhibition PKM2/stat3/cyclinD1 signal pathway[J]. LIFE SCIENCES, 2018,204:71-77.

[697] Yang H J, Zhou P, Huang H B, et al. Shikonin exerts antitumor activity via proteasome inhibition and cell death induction in vitro and in vivo[J]. INTERNATIONAL JOURNAL OF CANCER, 2009,124(10):2450-2459.

[698] Dai Y T, Liu Y P, Li J Y, et al. Shikonin inhibited glycolysis and sensitized cisplatin treatment in non-small cell lung cancer cells via the exosomal pyruvate kinase M2 pathway[J]. BIOENGINEERED, 2022,13(5):13906-13918.

[699] Kim H J, Hwang K E, Park D S, et al. Shikonin-induced necroptosis is enhanced by the inhibition of autophagy in non-small cell lung cancer cells[J]. JOURNAL OF TRANSLATIONAL MEDICINE, 2017,15.

[700] Chen Y, Sun L N, Qiao X R, et al. Signal-off/on electrogenerated chemiluminescence deoxyribosensors for assay of early lung cancer biomarker (NAP2) based on target-caused DNA charge transfer[J]. ANALYTICA CHIMICA ACTA, 2020,1103:67-74.

[701] Shah M M, Isrow D, Fareed M M, et al. Single institution experience treating adrenal metastases with stereotactic body radiation therapy[J]. JOURNAL OF CANCER RESEARCH AND THERAPEUTICS, 2019,15:S27-S32.

[702] Engstrom C A, Strohl R A, Rose L, et al. Sleep alterations in cancer patients[J]. CANCER NURSING, 1999,22(2):143-148.

[703] Perantoni E, Michailidis V, Siopi D, et al. Sleep and breathing disorders in newly diagnosed lung cancer patients[J]. EUROPEAN RESPIRATORY JOURNAL, 2013,42.

[704] Huang B H, Duncan M J, Cistulli P A, et al. Sleep and physical activity in relation to all-cause, cardiovascular disease and cancer mortality risk[J]. Br J Sports Med, 2022,56(13):718-724.

[705] Huang B H, Duncan M J, Cistulli P A, et al. Sleep and physical activity in relation to all-cause, cardiovascular disease and cancer mortality risk[J]. BRITISH JOURNAL OF SPORTS MEDICINE, 2022,56(13):718.

[706] Gooneratne N S, Dean G E, Rogers A E, et al. Sleep and quality of life in long-term lung cancer survivors[J]. Lung Cancer, 2007,58(3):403-410.

[707] Gooneratne N S, Dean G E, Rogers A E, et al. Sleep and quality of life in long-term lung cancer survivors[J]. LUNG CANCER, 2007,58(3):403-410.

[708] Martin R E, Loomis D M, Dean G E. Sleep and quality of life in lung cancer patients and survivors[J]. Journal of the American Association of Nurse Practitioners, 2021,34(2):284-291.

[709] Martin R E, Loomis D M, Dean G E. Sleep and quality of life in lung cancer patients and survivors[J]. JOURNAL OF THE AMERICAN ASSOCIATION OF NURSE PRACTITIONERS, 2022,34(2):284-291.

[710] Wang X Y, Zong X Y, Li N, et al. Sleep behaviors and risk of lung cancer in the UK Biobank.[J]. CANCER RESEARCH, 2021,81(13).

[711] Zhao C, Takahashi N, Rajan A. Sleep better on combination therapy: SLFN11 predicts response to veliparib and temozolomide in recurrent small cell lung cancer[J]. TRANSLATIONAL LUNG CANCER RESEARCH, 2018,7:S308-S311.

[712] Li X Y, Huang D H, Liu F H, et al. Sleep Characteristics and Cancer-Related Outcomes: An Umbrella Review of Systematic Reviews and Meta-Analyses of Observational Studies[J]. JOURNAL OF CLINICAL MEDICINE, 2022,11(24).

[713] Novaes E Brito R R, De Lorenzo B P, Xander P, et al. Sleep deprivation reduces TCD8+ and NKT cell numbers and increases the metastatic lung[J]. Brain, Behavior, and Immunity, 2011,25:S232.

[714] Pérez-Warnisher M T, Cabezas E, Troncoso M F, et al. Sleep disordered breathing and nocturnal hypoxemia are very prevalent in a lung cancer screening population and may condition lung cancer screening findings: results of the prospective Sleep Apnea In Lung Cancer Screening (SAILS) study[J]. Sleep Med, 2019,54:181-186.

[715] Cabezas E, Pérez-Warnisher M T, Troncoso M F, et al. Sleep Disordered Breathing Is Highly Prevalent in Patients with Lung Cancer: Results of the Sleep Apnea in Lung Cancer Study[J]. Respiration, 2019,97(2):119-124.

[716] Kaous M, Balachandran D, Pacheco G, et al. Sleep Disorders in Lung Cancer[J]. CHEST, 2017,152(4):1075A.

[717] Davidson J R, MacLean A W, Brundage M D, et al. Sleep disturbance in cancer patients[J]. SOCIAL SCIENCE & MEDICINE, 2002,54(9):1309-1321.

[718] Wang S Y, Chang H J, Lin C C. Sleep disturbances among patients with non-small cell lung cancer in Taiwan: Congruence between sleep log and actigraphy[J]. Cancer Nursing, 2010,33(1):E11-E17.

[719] Mihail E, Heras P, Hatzopoulos A, et al. Sleep disturbances and depression in patients with lung cancer[J]. EJC SUPPLEMENTS, 2009,7(2):510.

[720] Le Guen Y, Gagnadoux F, Hureaux J, et al. Sleep disturbances and impaired daytime functioning in outpatients with newly diagnosed lung cancer[J]. LUNG CANCER, 2007,58(1):139-143.

[721] Le Guen Y, Gagnadoux F, Hureaux J, et al. Sleep disturbances and impaired daytime functioning in outpatients with newly diagnosed lung cancer[J]. Lung Cancer, 2007,58(1):139-143.

[722] Chen M L, Yu C T, Yang C H. Sleep disturbances and quality of life in lung cancer patients undergoing chemotherapy[J]. LUNG CANCER, 2008,62(3):391-400.

[723] Rades D, Kopelke S, Tvilsted S, et al. Sleep Disturbances in Lung Cancer Patients Assigned to Definitive or Adjuvant Irradiation[J]. IN VIVO, 2021,35(6):3333-3337.

[724] Bülbül Y, Özlü T, Arınç S, et al. Sleep disturbances in patients with lung cancer in Turkey[J]. Tuberkuloz ve Toraks, 2018,66(4):297-303.

[725] Bulbul Y, Ozlu T, Arinc S, et al. Sleep disturbances in patients with lung cancer in Turkey[J]. TUBERKULOZ VE TORAK-TUBERCULOSIS AND THORAX, 2018,66(4):297-303.

[726] Bulbul Y, Ozlu T, Arinc S, et al. Sleep disturbances in patients with lung cancer in Turkey[J]. EUROPEAN RESPIRATORY JOURNAL, 2016,48.

[727] Bulbul Y, Ozlu T, Arinc S, et al. Sleep disturbances in patients with lung cancer in Turkey[J]. 2016,48(no pagination).

[728] Bülbül Y, Ozlu T, Arinc S, et al. Sleep disturbances in patients with lung cancer in Turkey[J]. European Respiratory Journal, 2016,48.

[729] Wong J Y, Bassig B A, Vermeulen R, et al. Sleep duration across the adult lifecourse and risk of lung cancer mortality: A cohort study in xuanwei, China[J]. Cancer Prevention Research, 2017,10(6):327-335.

[730] Wong J, Bassig B A, Vermeulen R, et al. Sleep Duration across the Adult Lifecourse and Risk of Lung Cancer Mortality: A Cohort Study in Xuanwei, China[J]. CANCER PREVENTION RESEARCH, 2017,10(6):327-335.

[731] Li J, Cao D, Huang Y, et al. Sleep duration and health outcomes: an umbrella review[J]. Sleep and Breathing, 2022,26(3):1479-1501.

[732] Li J, Cao D H, Huang Y, et al. Sleep duration and health outcomes: an umbrella review[J]. SLEEP AND BREATHING, 2022,26(3):1479-1501.

[733] Luojus M K, Lehto S M, Tolmunen T, et al. Sleep duration and incidence of lung cancer in ageing men[J]. BMC PUBLIC HEALTH, 2014,14.

[734] Khawaja O, Petrone A B, Aleem S, et al. Sleep duration and risk of lung cancer in the physicians' health study[J]. Chinese Journal of Lung Cancer, 2014,17(9):649-655.

[735] Peeri N C, Tao M H, Demissie S, et al. Sleep Duration, Chronotype, and Insomnia and the Risk of Lung Cancer: United Kingdom Biobank Cohort[J]. Cancer Epidemiol Biomarkers Prev, 2022,31(4):766-774.

[736] Peeri N C, Tao M H, Demissie S, et al. Sleep Duration, Chronotype, and Insomnia and the Risk of Lung Cancer: United Kingdom Biobank Cohort[J]. CANCER EPIDEMIOLOGY BIOMARKERS & PREVENTION, 2022,31(4):766-774.

[737] Sillah A, Watson N F, Peters U, et al. Sleep problems and risk of cancer incidence and mortality in an older cohort: The Cardiovascular Health Study (CHS)[J]. CANCER EPIDEMIOLOGY, 2022,76.

[738] Akyuz R G, Ugur O, Elcigil A. Sleep quality in lung cancer patients[J]. Asian Pacific journal of cancer prevention : APJCP, 2013,14(5):2909-2913.

[739] Akyuz R G, Ugur O, Elcigil A. Sleep Quality in Lung Cancer Patients[J]. ASIAN PACIFIC JOURNAL OF CANCER PREVENTION, 2013,14(5):2909-2913.

[740] He Y, Sun L Y, Peng K W, et al. Sleep quality, anxiety and depression in advanced lung cancer: patients and caregivers[J]. BMJ SUPPORTIVE & PALLIATIVE CARE, 2022,12(E2):E194-E200.

[741] Cordina-Duverger E, Uchai S, Tvardik N, et al. Sleep Traits, Night Shift Work and Lung Cancer Risk among Women: Results from a Population-Based Case-Control Study in France (The WELCA Study)[J]. INTERNATIONAL JOURNAL OF ENVIRONMENTAL RESEARCH AND PUBLIC HEALTH, 2022,19(23).

[742] Cordina-Duverger E, Uchai S, Tvardik N, et al. Sleep Traits, Night Shift Work and Lung Cancer Risk among Women: Results from a Population-Based Case-Control Study in France (The WELCA Study)[J]. International Journal of Environmental Research and Public Health, 2022,19(23).

[743] Dreher M, Krüger S, Schulze-Olden S, et al. Sleep-disordered breathing in patients with newly diagnosed lung cancer[J]. BMC Pulm Med, 2018,18(1):72.

[744] Dreher M, Krüger S, Schulze-Olden S, et al. Sleep-disordered breathing in patients with newly diagnosed lung cancer[J]. BMC Pulmonary Medicine, 2018,18(1).

[745] Dean G E, Abu S E, Yingrengreung S, et al. Sleeping with the enemy: sleep and quality of life in patients with lung cancer[J]. Cancer Nurs, 2015,38(1):60-70.

[746] Dean G E, Sabbah E A, Yingrengreung S, et al. Sleeping with the enemy: Sleep and quality of life in patients with lung cancer[J]. Cancer Nursing, 2015,38(1):60-70.

[747] Vena C, Parker K P, Allen R, et al. Sleep-wake disturbances and quality of life in patients with advanced lung cancer[J]. ONCOLOGY NURSING FORUM, 2006,33(4):761-769.

[748] Vena C, Parker K, Allen R, et al. Sleep-wake disturbances and quality of life in patients with advanced lung cancer.[J]. Oncology nursing forum, 2006,33(4):761-769.

[749] Mills D A, Fekrazad H M, Verschraegen C F. SNS-595, a naphthyridine cell cycle inhibitor and stimulator of apoptosis for the treatment of cancers[J]. CURRENT OPINION IN INVESTIGATIONAL DRUGS, 2008,9(6):647-657.

[750] Chabowski M, Polanski J, Mazur G, et al. Sociodemographic and Clinical Determinants of Quality of Life of Patients with Non-small Cell Lung Cancer[M]//Pokorski M. 2017:1-10.

[751] Nurgalieva A K, Popov V E, Skripova V S, et al. Sodium-dependent phosphate transporter NaPi2b as a potential predictive marker for targeted therapy of ovarian cancer[J]. BIOCHEMISTRY AND BIOPHYSICS REPORTS, 2021,28.

[752] Collins K, Alkashash A M, Hwang M, et al. Somatic-Type Yolk Sac Tumor Arising as a Predominant Component of Bladder Urothelial Carcinoma[J]. INTERNATIONAL JOURNAL OF SURGICAL PATHOLOGY, 2022,30(2):207-213.

[753] Norsa A, Martino V. Somatostatin, retinoids, melatonin, vitamin D, bromocriptine, and cyclophosphamide in advanced non-small-cell lung cancer patients with low performance status[J]. CANCER BIOTHERAPY AND RADIOPHARMACEUTICALS, 2006,21(1):68-73.

[754] Laurent E, Begueret H, Bonhomme B, et al. SOX10, GATA3, GCDFP15, Androgen Receptor, and Mammaglobin for the Differential Diagnosis Between Triple-negative Breast Cancer and TTF1-negative Lung Adenocarcinoma[J]. AMERICAN JOURNAL OF SURGICAL PATHOLOGY, 2019,43(3):293-302.

[755] Liu S H, Zhang H J, Wang F, et al. Specific nursing effectively improves dyspnea and sleep quality of patients with lung cancer undergoing chemotherapy[J]. INTERNATIONAL JOURNAL OF CLINICAL AND EXPERIMENTAL MEDICINE, 2020,13(11):8206-8215.

[756] Dupuis C, Filaire M, Filaire E. Spontaneous physical activity and quality of life among patients with lung cancer awaiting for lung resection[J]. SCIENCE & SPORTS, 2015,30(1):47-50.

[757] Osoba D, Hsu M A, Copley-Merriman C, et al. Stated preferences of patients with cancer for health-related quality-of-life (HRQOL) domains during treatment[J]. Qual Life Res, 2006,15(2):273-283.

[758] Osoba D, Hsu M A, Copley-Merriman C, et al. Stated preferences of patients with cancer for health-related quality-of-life (HRQOL) domains during treatment[J]. QUALITY OF LIFE RESEARCH, 2006,15(2):273-283.

[759] Xiang Y, Song J, Yan Y, et al. STATUS SURVEY OF SLEEP QUALITY OF LUNG CANCER PATIENTS AND ANALYSIS OF INFLUENCING FACTORS[J]. ACTA MEDICA MEDITERRANEA, 2022,38(5):3409-3413.

[760] Rao L G. Steroid abnormalities and lung cancer[J]. Lancet, 1970,2(7685):1259.

[761] Johns M W. Steroid abnormalities in lung cancer[J]. Lancet, 1970,2(7683):1139.

[762] Johns M W. Steroid abnormalities in lung cancer.[J]. Lancet, 1970,2(7683):1139.

[763] Barre P V, Padmaja G, Rana S, et al. Stress and quality of life in cancer patients: Medical and psychological intervention[J]. INDIAN JOURNAL OF PSYCHOLOGICAL MEDICINE, 2018,40(3):232-238.

[764] Vennila K N, Selvakumar B, Satish V, et al. Structure-based design, synthesis, biological evaluation, and molecular docking of novel 10-methoxy dibenzo[b,h][1,6]naphthyridinecarboxamides[J]. MEDICINAL CHEMISTRY RESEARCH, 2021,30(1):133-141.

[765] Niu S T, Zhang M Z. Study on the effect of chemotherapy on physical status of patients with non-small-cell lung cancer[J]. BIOMEDICAL RESEARCH-INDIA, 2017,28(6):2466-2470.

[766] Zarogoulidis P, Steiropoulos P, Perantoni E, et al. Subjective sleep quality in lung cancer patients before and after chemotherapy[J]. Thoracic Cancer, 2013,4(2):138-142.

[767] Zarogoulidis P, Steiropoulos P, Perantoni E, et al. Subjective sleep quality in lung cancer patients before and after chemotherapy[J]. THORACIC CANCER, 2013,4(2):138-142.

[768] Beck J, Miller M A, Frank C, et al. Surfactant Protein A and Napsin A in the Immunohistochemical Characterization of Canine Pulmonary Carcinomas: Comparison With Thyroid Transcription Factor-1[J]. VETERINARY PATHOLOGY, 2017,54(5):767-774.

[769] Higashiyama M, Kodama K, Takami K, et al. Surgical treatment of bone metastasis followed by a primary lung cancer lesion: Report of a case[J]. SURGERY TODAY, 2004,34(7):600-605.

[770] Silvoniemi M, Vasankari T, Löyttyniemi E, et al. Symptom assessment for patients with non-small cell lung cancer scheduled for chemotherapy[J]. Anticancer Research, 2016,36(8):4123-4128.

[771] Silvoniemi M, Vasankari T, Loyttyniemi E, et al. Symptom Assessment for Patients with Non-small Cell Lung Cancer Scheduled for Chemotherapy[J]. ANTICANCER RESEARCH, 2016,36(8):4123-4128.

[772] Krishnasamy M, Gough K, Ugalde A, et al. Symptom clusters in patients with lung cancer[J]. Asia-Pacific Journal of Clinical Oncology, 2009,5:A142.

[773] Oi-Ling K, Man-Wah D T, Kam-Hung D N. Symptom distress as rated by advanced cancer patients, caregivers and physicians in the last week of life[J]. PALLIATIVE MEDICINE, 2005,19(3):228-233.

[774] Oi-Ling K, Man-Wah D T S E, Kam-Hung D N G. Symptom distress as rated by advanced cancer patients, caregivers and physicians in the last week of life[J]. Palliative Medicine, 2005,19(3):228-233.

[775] Fagundes C P, Shi Q L, Vaporciyan A A, et al. Symptom recovery after thoracic surgery: Measuring patient-reported outcomes with the MD Anderson Symptom Inventory[J]. JOURNAL OF THORACIC AND CARDIOVASCULAR SURGERY, 2015,150(3):613-619.

[776] Liu J H, Liu X Y, Dong M, et al. Symptom trajectories during chemotherapy in patients with non-small cell lung cancer (NSCLC) and the function of prolonging low dose dexamethasone in promoting enhanced recovery after chemotherapy[J]. THORACIC CANCER, 2021,12(6):783-795.

[777] Tang L, Yu H F, Dai W, et al. Symptom Trajectories Informing Patient Care After Lung Cancer Surgery: A Longitudinal Patient-Reported Outcome Study[J]. ANNALS OF SURGICAL ONCOLOGY.

[778] Duan L R, Cui H X, Zhang W L, et al. Symptoms and experiences of frailty in lung cancer patients with chemotherapy: A mixed-method approach[J]. FRONTIERS IN ONCOLOGY, 2022,12.

[779] Lovgren M, Tishelman C, Sprangers M, et al. Symptoms and problems with functioning among women and men with inoperable lung cancer - A longitudinal study[J]. LUNG CANCER, 2008,60(1):113-124.

[780] Lövgren M, Tishelman C, Sprangers M, et al. Symptoms and problems with functioning among women and men with inoperable lung cancer-A longitudinal study[J]. Lung Cancer, 2008,60(1):113-124.

[781] Shrestha J P, Fosso M Y, Bearss J, et al. Synthesis and anticancer structure activity relationship investigation of cationic anthraquinone analogs[J]. EUROPEAN JOURNAL OF MEDICINAL CHEMISTRY, 2014,77:96-102.

[782] Li M, Wang Y X, Zhang J Y, et al. Synthesis and Biological Evaluation of Novel Aromatic Imide-Polyamine Conjugates[J]. MOLECULES, 2016,21(12).

[783] Thanh N H, Phuong H T, Anh L, et al. Synthesis and Cytotoxic Evaluation of Fluoro and Trifluoromethyl Substituents Containing Novel Naphthoquinone-Fused Podophyllotoxins[J]. NATURAL PRODUCT COMMUNICATIONS, 2022,17(10).

[784] Madda J, Venkatesham A, Naveen Kumar B, et al. Synthesis of novel chromeno-annulated cis-fused pyrano[3,4-c]benzopyran and naphtho pyran derivatives via domino aldol-type/hetero Diels-Alder reaction and their cytotoxicity evaluation[J]. Bioorganic and Medicinal Chemistry Letters, 2014.

[785] Li S, Xu S, Tang Y, et al. Synthesis, anticancer activity and DNA-binding properties of novel 4-pyrazolyl-1,8-naphthalimide derivatives[J]. Bioorganic and Medicinal Chemistry Letters, 2014,24(2):586-590.

[786] Li S H, Xu S J, Tang Y H, et al. Synthesis, anticancer activity and DNA-binding properties of novel 4-pyrazolyl-1,8-naphthalimide derivatives[J]. BIOORGANIC & MEDICINAL CHEMISTRY LETTERS, 2014,24(2):586-590.

[787] Kavaliauskas P, Opazo F S, Acevedo W, et al. Synthesis, Biological Activity, and Molecular Modelling Studies of Naphthoquinone Derivatives as Promising Anticancer Candidates Targeting COX‐2[J]. Pharmaceuticals, 2022,15(5).

[788] Mooney A, Corry A J, Ruairc C N, et al. Synthesis, characterisation and biological evaluation of N-(ferrocenyl)naphthoyl amino acid esters as anticancer agents[J]. DALTON TRANSACTIONS, 2010,39(35):8228-8239.

[789] Wilson J J, Lippard S J. Synthesis, Characterization, and Cytotoxicity of Platinum(IV) Carbamate Complexes[J]. INORGANIC CHEMISTRY, 2011,50(7):3103-3115.

[790] Southerland M R, DeBord M A, Johnson N A, et al. Synthesis, characterization, in vitro SAR study, and preliminary in vivo toxicity evaluation of naphthylmethyl substituted bis-imidazolium salts[J]. BIOORGANIC & MEDICINAL CHEMISTRY, 2021,30.

[791] Tan X J, Wang D, Hei X M, et al. Synthesis, crystal structures, antiproliferative activities and reverse docking studies of eight novel Schiff bases derived from benzil[J]. ACTA CRYSTALLOGRAPHICA SECTION C-STRUCTURAL CHEMISTRY, 2020,76:44.

[792] Konovalov B, Zivkovic M D, Milovanovic J Z, et al. Synthesis, cytotoxic activity and DNA interaction studies of new dinuclear platinum(ii) complexes with an aromatic 1,5-naphthyridine bridging ligand: DNA binding mode of polynuclear platinum(ii) complexes in relation to the complex structure[J]. DALTON TRANSACTIONS, 2018,47(42):15091-15102.

[793] Zhang X K. Targeting Nur77 translocation[J]. EXPERT OPINION ON THERAPEUTIC TARGETS, 2007,11(1):69-79.

[794] Ryczkowska M, Maciejewska N, Olszewski M, et al. Tetrahydroquinolinone derivatives exert antiproliferative effect on lung cancer cells through apoptosis induction[J]. SCIENTIFIC REPORTS, 2022,12(1).

[795] Ahn M J, Won H H, Lee J, et al. The 18p11.22 locus is associated with never smoker non-small cell lung cancer susceptibility in Korean populations[J]. HUMAN GENETICS, 2012,131(3):365-372.

[796] Roh M H, Schmidt L, Placido J, et al. The application and diagnostic utility of immunocytochemistry on direct smears in the diagnosis of pulmonary adenocarcinoma and squamous cell carcinoma[J]. Diagnostic Cytopathology, 2011.

[797] Roh M H, Schmidt L, Placido J, et al. The application and diagnostic utility of immunocytochemistry on direct smears in the diagnosis of pulmonary adenocarcinoma and squamous cell carcinoma[J]. DIAGNOSTIC CYTOPATHOLOGY, 2012,40(11):949-955.

[798] Stone C R, Haig T R, Fiest K M, et al. The association between sleep duration and cancer-specific mortality: a systematic review and meta-analysis[J]. CANCER CAUSES & CONTROL, 2019,30(5):501-525.

[799] Grutsch J F, Ferrans C, Wood P A, et al. The association of quality of life with potentially remediable disruptions of circadian sleep/activity rhythms in patients with advanced lung cancer[J]. BMC CANCER, 2011,11.

[800] Brzecka A, Sarul K, Dyła T, et al. The association of sleep disorders, obesity and sleep-related hypoxia with cancer[J]. Current Genomics, 2020,21(6):444-453.

[801] Li J M, Hsu P C, Kuan F C, et al. The cancer stemness inhibitor napabucasin suppresses small cell lung cancer growth through SOX2 expression[J]. AMERICAN JOURNAL OF CANCER RESEARCH, 2022,12(10):4637.

[802] Zhang Y, Luo Y H, Piao X J, et al. The design of 1,4-naphthoquinone derivatives and mechanisms underlying apoptosis induction through ROS-dependent MAPK/Akt/STAT3 pathways in human lung cancer cells[J]. BIOORGANIC & MEDICINAL CHEMISTRY, 2019,27(8):1577-1587.

[803] Eldridge E, Aenchbacher N, Brooks D, et al. The differential impact of symptom burden on various sleep components in a sample of lung cancer patients[J]. PSYCHO-ONCOLOGY, 2018,27:68.

[804] Lin S C, Chen M F, Li T C, et al. The distribution of yin-deficient symptoms and their relationship on survival rate in cancer patients with Yin-Deficiency[J]. American Journal of Chinese Medicine, 2008,36(4):655-663.

[805] Al-Keilani M S, Alzoubi K H, Jaradat S A. The effect of combined treatment with sodium phenylbutyrate and cisplatin, erlotinib, or gefitinib on resistant NSCLC cells[J]. CLINICAL PHARMACOLOGY-ADVANCES AND APPLICATIONS, 2018,10:135-140.

[806] Yan C M, Liu Y. The Effect of Dezocine Combined with Oxycodone in the Treatment of Advanced Lung Cancer with Bone Metastasis and Severe Cancer Pain on the Numerical Rating Scale Score of Patients[J]. INDIAN JOURNAL OF PHARMACEUTICAL SCIENCES, 2021,83:107-111.

[807] Li L, Wu Y L, Chai Y, et al. THE EFFECT OF GROUP REHABILITATION TRAINING IN THE WARD ON THE PSYCHOLOGICAL STATUS AND QUALITY OF LIFE OF LUNG CANCER PATIENTS[J]. Supportive Care in Cancer, 2022,30:S42.

[808] Ozalevli S, Ilgin D, Karaali H K, et al. The effect of in-patient chest physiotherapy in lung cancer patients[J]. SUPPORTIVE CARE IN CANCER, 2010,18(3):351-358.

[809] Shan M, Zhang L, Fang D, et al. The effect of integrative traditional and Western medicine to the symptom burdened and quality of life of elderly patients with advanced non-small cell lung Cancer[J]. Quality of Life Research, 2017,26(1):60.

[810] Turcott J G, Del Rocío Guillen Núñez M, Flores-Estrada D, et al. The effect of nabilone on appetite, nutritional status, and quality of life in lung cancer patients: a randomized, double-blind clinical trial[J]. Supportive Care in Cancer, 2018,26(9):3029-3038.

[811] Wang H M, Liang Y, Lu D, et al. The effect of targeted nursing on the quality of sleep and life in lung cancer patients undergoing chemotherapy[J]. AMERICAN JOURNAL OF TRANSLATIONAL RESEARCH, 2021,13(5):4825-4834.

[812] Coker S A, Hurwitz H I, Sharma S, et al. The effects of lapatinib on cardiac repolarization: results from a placebo controlled, single sequence, crossover study in patients with advanced solid tumors[J]. Cancer Chemotherapy and Pharmacology, 2019,84(2):383-392.

[813] McNeil J, Heer E, Willemsen R F, et al. The effects of shift work and sleep duration on cancer incidence in Alberta`s Tomorrow Project cohort[J]. Cancer Epidemiology, 2020,67.

[814] McNeil J, Heer E, Willemsen R F, et al. The effects of shift work and sleep duration on cancer incidence in Alberta's Tomorrow Project cohort[J]. CANCER EPIDEMIOLOGY, 2020,67.

[815] Tang H, Chen L, Wang Y, et al. The efficacy of music therapy to relieve pain, anxiety, and promote sleep quality, in patients with small cell lung cancer receiving platinum-based chemotherapy[J]. Supportive Care in Cancer, 2021,29(12):7299-7306.

[816] Tang H, Chen L, Wang Y, et al. The efficacy of music therapy to relieve pain, anxiety, and promote sleep quality, in patients with small cell lung cancer receiving platinum-based chemotherapy[J]. 2021,29(12):7299-7306.

[817] Tang H K, Chen L P, Wang Y C, et al. The efficacy of music therapy to relieve pain, anxiety, and promote sleep quality, in patients with small cell lung cancer receiving platinum-based chemotherapy[J]. SUPPORTIVE CARE IN CANCER, 2021,29(12):7299-7306.

[818] Samukawa T, Hamada T, Mizuno K, et al. The elevation serum napsin A in primary lung adenocarcinoma, compared with CEA[J]. EUROPEAN RESPIRATORY JOURNAL, 2012,40.

[819] Tsang M, Gan S Q, Boscardin W J, et al. The epidemiology of preexisting geriatric and palliative conditions in older adults with poor prognosis cancers[J]. JOURNAL OF THE AMERICAN GERIATRICS SOCIETY, 2022,70(12):3402-3412.

[820] Shames D S, Wistuba I I. The evolving genomic classification of lung cancer[J]. JOURNAL OF PATHOLOGY, 2014,232(2):121-133.

[821] Dickerson S S, Sabbah E A, Ziegler P, et al. The experience of a diagnosis of advanced lung cancer: Sleep is not a priority when living my life[J]. Oncology Nursing Forum, 2012,39(5):492-499.

[822] Dickerson S S, Abu Sabbah E, Ziegler P, et al. The Experience of a Diagnosis of Advanced Lung Cancer: Sleep Is Not a Priority When Living My Life[J]. ONCOLOGY NURSING FORUM, 2012,39(5):492-499.

[823] Ma Y F, Fan M Y, Dai L, et al. The expression of TTF-1 and Napsin A in early-stage lung adenocarcinoma correlates with the results of surgical treatment[J]. TUMOR BIOLOGY, 2015,36(10):8085-8092.

[824] Zunic S S, Sekulic S, Djordjevic-Denic G V, et al. The importance of cytochemical analysis of alveolar macrophages in the evaluation of their biological properties[J]. International Journal of Biological Markers, 1996,11(2):82-89.

[825] Cauli O, Vitale E. The Interdisciplinary Management and Safety Perspectives in Endocrine, Metabolic and Psychiatric Disorder[J]. ENDOCRINE METABOLIC & IMMUNE DISORDERS-DRUG TARGETS, 2022,22(13):1233-1234.

[826] Holik A Z, Filby C E, Pasquet J, et al. The LIM-domain only protein 4 contributes to lung epithelial cell proliferation but is not essential for tumor progression[J]. RESPIRATORY RESEARCH, 2015,16.

[827] Lindskog C, Fagerberg L, Hallstrom B, et al. The lung-specific proteome defined by integration of transcriptomics and antibody-based profiling[J]. FASEB JOURNAL, 2014,28(12):5184-5196.

[828] Acharya B R, Bhattacharyya S, Choudhury D, et al. The microtubule depolymerizing agent naphthazarin induces both apoptosis and autophagy in A549 lung cancer cells[J]. APOPTOSIS, 2011,16(9):924-939.

[829] Liu H M, Ma L L, Li C Y, et al. The molecular mechanism of chronic stress affecting the occurrence and development of breast cancer and potential drug therapy[J]. TRANSLATIONAL ONCOLOGY, 2022,15(1).

[830] Jalbrzykowska K, Chrzanowska A, Roszkowski P, et al. The New Face of a Well-Known Antibiotic: A Review of the Anticancer Activity of Enoxacin and Its Derivatives[J]. CANCERS, 2022,14(13).

[831] Huang H W, Mohan S K, Yu C. The NMR solution structure of human epidermal growth factor (hEGF) at physiological pH and its interactions with suramin[J]. BIOCHEMICAL AND BIOPHYSICAL RESEARCH COMMUNICATIONS, 2010,402(4):705-710.

[832] Ji L Y, Liu X, Zhang S W, et al. The Novel Triazolonaphthalimide Derivative LSS-11 Synergizes the Anti-Proliferative Effect of Paclitaxel via STAT3-Dependent MDR1 and MRP1 Downregulation in Chemoresistant Lung Cancer Cells[J]. MOLECULES, 2017,22(11).

[833] Badaoui S, Shahnam A, McKinnon R A, et al. The predictive utility of patient-reported outcomes and performance status for survival in metastatic lung cancer patients treated with chemoimmunotherapy[J]. TRANSLATIONAL LUNG CANCER RESEARCH, 2022,11(3):432.

[834] Peng S M, Ren J J, Yu N, et al. The prognostic value of the Naples prognostic score for patients with non-small-cell lung cancer[J]. SCIENTIFIC REPORTS, 2022,12(1).

[835] Mystakidou K, Parpa E, Tsilika E, et al. The relationship of subjective sleep quality, pain, and quality of life in advanced cancer patients[J]. SLEEP, 2007,30(6):737-742.

[836] Hu L Y, Chen P M, Hu Y W, et al. The risk of cancer among patients with sleep disturbance: a nationwide retrospective study in Taiwan[J]. Ann Epidemiol, 2013,23(12):757-761.

[837] Tirino V, Camerlingo R, Franco R, et al. The role of CD133 in the identification and characterisation of tumour-initiating cells in non-small-cell lung cancer[J]. EUROPEAN JOURNAL OF CARDIO-THORACIC SURGERY, 2009,36(3):446-453.

[838] Kallet R H. The role of inhaled opioids and furosemide for the treatment of dyspnea[J]. Respiratory Care, 2007,52(7):900-910.

[839] Hwang G H, Ryu J M, Jeon Y J, et al. The role of thioredoxin reductase and glutathione reductase in plumbagin-induced, reactive oxygen species-mediated apoptosis in cancer cell lines[J]. EUROPEAN JOURNAL OF PHARMACOLOGY, 2015,765:384-393.

[840] Sun S Y, Yue P, Chen X Y, et al. The synthetic retinoid CD437 selectively induces apoptosis in human lung cancer cells while sparing normal human lung epithelial cells[J]. CANCER RESEARCH, 2002,62(8):2430-2436.

[841] Reginelli A, Belfiore M P, Monti R, et al. The texture analysis as a predictive method in the assessment of the cytological specimen of CT-guided FNAC of the lung cancer[J]. MEDICAL ONCOLOGY, 2020,37(6).

[842] Simon M, Pop B, Toma I L, et al. The use of EBUS-TBNA and ROSE in the diagnosis of lung cancer[J]. ROMANIAN JOURNAL OF MORPHOLOGY AND EMBRYOLOGY, 2017,58(1):79-87.

[843] Ao M H, Zhang H, Sakowski L, et al. The utility of a novel triple marker (combination of TTF1, napsin A, and p40) in the subclassification of non-small cell lung cancer[J]. HUMAN PATHOLOGY, 2014,45(5):926-934.

[844] van Zyl A, Schubert P T, Koegelenberg C. The utility of TTF-1, napsin A, CK5 and p63 staining in the sub-classification of non-small cell carcinoma of the lung[J]. CYTOPATHOLOGY, 2019,30(6):586-591.

[845] Galindo I, Gomez-Morales M, Diaz-Cano I, et al. The value of desmosomal plaque-related markers to distinguish squamous cell carcinoma and adenocarcinoma of the lung[J]. UPSALA JOURNAL OF MEDICAL SCIENCES, 2020,125(1):19-29.

[846] Zhang Y, Sun S B, Xu W P, et al. Thioredoxin reductase 1 inhibitor shikonin promotes cell necroptosis via SecTRAPs generation and oxygen-coupled redox cycling[J]. FREE RADICAL BIOLOGY AND MEDICINE, 2022,180:52-62.

[847] Yeon S W, Kwon H Y, Nam J I, et al. Three new naphthalenes from the roots of Hibiscus syriacus[J]. PHYTOCHEMISTRY LETTERS, 2019,33:110-113.

[848] Pøhl M, Olsen K E, Holst R, et al. Tissue microarrays in non-small-cell lung cancer: Reliability of immunohistochemically-determined biomarkers[J]. Clinical Lung Cancer, 2014,15(3):222-230.

[849] Pohl M, Olsen K E, Holst R, et al. Tissue Microarrays in Non-Small-Cell Lung Cancer: Reliability of Immunohistochemically-Determined Biomarkers[J]. CLINICAL LUNG CANCER, 2014,15(3):222.

[850] Ding C, Li L, Zhang Y, et al. Toll-like receptor agonist rMBP-NAP enhances antitumor cytokines production and CTL activity of peripheral blood mononuclear cells from patients with lung cancer[J]. EUROPEAN JOURNAL OF IMMUNOLOGY, 2019,49:1660-1661.

[851] Wang C B, Lorente-Macias A, Wells C, et al. Towards a RIOK2 chemical probe: cellular potency improvement of a selective 2-(acylamino)pyridine series[J]. RSC MEDICINAL CHEMISTRY, 2021,12(1):129-136.

[852] Dean G E, Ziegler P, Chen H B, et al. Trajectory of insomnia symptoms in older adults with lung cancer: using mixed methods[J]. SUPPORTIVE CARE IN CANCER, 2019,27(6):2255-2263.

[853] Dean G E, Ziegler P, Chen H, et al. Trajectory of insomnia symptoms in older adults with lung cancer: using mixed methods[J]. Supportive Care in Cancer, 2019,27(6):2255-2263.

[854] Halle I H, Westgaard T K, Wahba A, et al. Trajectory of sleep disturbances in patients undergoing lung cancer surgery: a prospective study[J]. INTERACTIVE CARDIOVASCULAR AND THORACIC SURGERY, 2017,25(2):285-291.

[855] Halle I H, Krystadwestgaard T, Wahba A, et al. Trajectory of sleep disturbances in patients undergoing lung cancer surgery: A prospective study[J]. Interactive Cardiovascular and Thoracic Surgery, 2017,25(2):285-291.

[856] Gan H Z, Xu X X, Bai Y Y. Trametes robiniophila represses angiogenesis and tumor growth of lung cancer via strengthening let-7d-5p and targeting NAP1L1[J]. BIOENGINEERED, 2022,13(3):6698-6710.

[857] Tian X L, Liu Y Y, Ye L, et al. Treatment Activity of a New Mn(II)-Coordination Polymer on Lung Cancer by Activating mTORC1 Signaling Pathway[J]. LATIN AMERICAN JOURNAL OF PHARMACY, 2022,41(9):1758-1763.

[858] Hamada N, Yanagihara T, Suzuki K, et al. Treatment with a programmed cell death-1-specific antibody has little effect on afatinib- and naphthalene-induced acute pneumonitis in mice[J]. BIOCHEMICAL AND BIOPHYSICAL RESEARCH COMMUNICATIONS, 2017,491(3):656-661.

[859] Niu L L, Xu C, Zhou Y T, et al. Tree bark as a biomonitor for assessing the atmospheric pollution and associated human inhalation exposure risks of polycyclic aromatic hydrocarbons in rural China[J]. ENVIRONMENTAL POLLUTION, 2019,246:398-407.

[860] Li S, Shi Y, Shang X Y, et al. Triterpenoids from the roots of Pterospermum heterophyllum Hance[J]. JOURNAL OF ASIAN NATURAL PRODUCTS RESEARCH, 2009,11(7):652-657.

[861] Huang C, He C P, Ruan P, et al. TSPYL5 activates endoplasmic reticulum stress to inhibit cell proliferation, migration and invasion in colorectal cancer[J]. ONCOLOGY REPORTS, 2020,44(2):449-456.

[862] Porcel J M, Palma R, Bielsa S, et al. TTF-1 and napsin A on cell blocks and supernatants of pleural fluids for labeling malignant effusions[J]. RESPIROLOGY, 2015,20(5):831-833.

[863] Liao M, Zeng C F, Liang F P. Two new dimeric naphthoquinones from Arnebia euchroma[J]. PHYTOCHEMISTRY LETTERS, 2020,37:106-109.

[864] Gu C, Zhai M, Lü A, et al. Ultrasound-guided stellate ganglion block improves sleep quality in elderly patients early after thoracoscopic surgery for lung cancer: a randomized controlled study[J]. Nan fang yi ke da xue xue bao = Journal of Southern Medical University, 2022,42(12):1807-1814.

[865] Jeklin A T, Kumarahuru R, Amalgeer M, et al. Understanding the effects of chemotherapy on sleep disturbance in lung cancer patients: A systematic review[J]. ASIA-PACIFIC JOURNAL OF CLINICAL ONCOLOGY, 2021,17:172.

[866] Joshi R, Das Mukherjee D, Chakrabarty S, et al. Unveiling the Potential of Unfused Bichromophoric Naphthalimide To Induce Cytotoxicity by Binding to Tubulin: Breaks Monotony of Naphthalimides as Conventional Intercalators[J]. JOURNAL OF PHYSICAL CHEMISTRY B, 2018,122(14):3680-3695.

[867] Reddy V, Myers B, Brownstone N, et al. Update on Sleep and Pulmonary Comorbidities in Psoriasis[J]. CURRENT DERMATOLOGY REPORTS, 2020,9(1):30-35.

[868] Huang S Y, Li Q, Liu H, et al. Urinary monohydroxylated polycyclic aromatic hydrocarbons in the general population from 26 provincial capital cities in China: Levels, influencing factors, and health risks[J]. ENVIRONMENT INTERNATIONAL, 2022,160.

[869] Taunk K, Porto-Figueira P, Pereira J, et al. Urinary Volatomic Expression Pattern: Paving the Way for Identification of Potential Candidate Biosignatures for Lung Cancer[J]. METABOLITES, 2022,12(1).

[870] Guo R, Tian Y, Zhang N, et al. Use of dual-marker staining to differentiate between lung squamous cell carcinoma and adenocarcinoma[J]. JOURNAL OF INTERNATIONAL MEDICAL RESEARCH, 2020,48(4).

[871] Hirano T, Gong Y B, Yoshida K, et al. Usefulness of TA02 (napsin A) to distinguish primary lung adenocarcinoma from metastatic lung adenocarcinoma[J]. LUNG CANCER, 2003,41(2):155-162.

[872] Sharma R, Wang Y, Chen L, et al. Utility of a novel triple marker (combination of thyroid transcription factor 1, Napsin A, and P40) in the subclassification of non-small cell lung carcinomas using fine-needle aspiration cases[J]. Human Pathology, 2016,54:8-16.

[873] Zhang P, Han Y P, Huang L, et al. Value of napsin A and thyroid transcription factor-1 in the identification of primary lung adenocarcinoma[J]. ONCOLOGY LETTERS, 2010,1(5):899-903.

[874] Chen A, Kem M, Huynh T, et al. Value of p40/Napsin A (NapA) Dual Immunohistochemistry (IHC) in Subtyping Poorly-Differentiated Non-Small Cell Lung Cancer (NSCLC)[J]. MODERN PATHOLOGY, 2018,31:728-729.

[875] Chen A, Kem M, Huynh T, et al. Value of p40/Napsin A (NapA) Dual Immunohistochemistry (IHC) in Subtyping Poorly-Differentiated Non-Small Cell Lung Cancer (NSCLC)[J]. LABORATORY INVESTIGATION, 2018,98:728-729.

[876] Kozono D E, Stinchcombe T, Salama J K, et al. Veliparib (Vel) in combination with chemoradiotherapy (CRT) of carboplatin/paclitaxel (C/P) plus radiation in patients (pts) with stage III non-small cell lung cancer (NSCLC) (M14-360/AFT-07)[J]. Journal of Clinical Oncology, 2019,37.

[877] Messina G, Bove M, Natale G, et al. Ventilation challenge in rigid bronchoscopy: Laser tube as an alternative management in patients with lung cancer and central airway obstruction[J]. THORACIC CANCER, 2023,14(1):24-29.

[878] Eastwood P R, Takahashi K, Lee P, et al. Year in review 2010: Interstitial lung diseases, acute lung injury, sleep, physiology, imaging, bronchoscopic intervention and lung cancer[J]. RESPIROLOGY, 2011,16(3):553-563.

[879] Takahashi K, Eves N D, Piper A, et al. Year in review 2011: Acute lung injury, interstitial lung diseases, physiology, sleep and lung cancer[J]. RESPIROLOGY, 2012,17(3):554-562.

**Part Ⅱ：Review(n=384)**

[1] Marandino L, Trastu F, Ghisoni E, et al. 1626P Time trends (2012-2016 vs 2017-2021) in health-related quality of life (QoL) assessment and reporting in oncology: A systematic review of randomized phase III trials[J]. Annals of Oncology, 2022,33:S1284-S1285.

[2] Yang M, Yang Y, Chen J, et al. A case report of primary signet ring cell carcinoma of the lung: Imaging study and literature review[J]. Translational Lung Cancer Research, 2021,10(9):3840-3849.

[3] Sakshi, Haritash A K. A comprehensive review of metabolic and genomic aspects of PAH-degradation[J]. ARCHIVES OF MICROBIOLOGY, 2020,202(8):2033-2058.

[4] Hashemi M, Hosseinzadeh H. A comprehensive review on biological activities and toxicology of crocetin[J]. Food and Chemical Toxicology, 2019,130:44-60.

[5] Sood A, Barton D L, Bauer B A, et al. A critical review of complementary therapies for cancer-related fatigue[J]. Integrative Cancer Therapies, 2007,6(1):8-13.

[6] Li C X, Zhang L, Yan Y R, et al. A narrative review of exploring potential salivary biomarkers in respiratory diseases: still on its way[J]. JOURNAL OF THORACIC DISEASE, 2021,13(7):4541-4553.

[7] Ho C, Martinusen D, Lo C. A Review of Cannabis in Chronic Kidney Disease Symptom Management[J]. Canadian Journal of Kidney Health and Disease, 2019,6.

[8] Mearini L, Colella R, Zucchi A, et al. A review of penile metastasis[J]. Oncology Reviews, 2012,6(1):80-87.

[9] Tuvel A L, Winiger E A, Ross J M. A Review of the Effects of Adolescent Cannabis Use on Physical Health[J]. Child and Adolescent Psychiatric Clinics of North America, 2023,32(1):85-105.

[10] Esther Kim J E, Dodd M J, Aouizerat B E, et al. A Review of the Prevalence and Impact of Multiple Symptoms in Oncology Patients[J]. Journal of Pain and Symptom Management, 2009,37(4):715-736.

[11] North D W, Abdo K M, Benson J M, et al. A review of whole animal bioassays of the carcinogenic potential of naphthalene[J]. Regulatory Toxicology and Pharmacology, 2008,51(2 SUPLL):6-14.

[12] Ellis P M, Blais N, Soulieres D, et al. A Systematic Review and Canadian Consensus Recommendations on the Use of Biomarkers in the Treatment of Non-small Cell Lung Cancer[J]. JOURNAL OF THORACIC ONCOLOGY, 2011,6(8):1379-1391.

[13] Yu X, Lim C E D, Cheng N C L. A systematic review and meta-analysis of acupuncture and related therapies for palliative cancer care[J]. Focus on Alternative and Complementary Therapies, 2016,21(2):97-98.

[14] Luckett T, San Martin A, Currow D C, et al. A systematic review and meta-analysis of studies comparing burden from lung cancer and chronic obstructive pulmonary disease[J]. Palliative Medicine, 2020,34(10):1291-1304.

[15] Lim Y L, Teoh S E, Yaow C Y L, et al. A Systematic Review and Meta-Analysis of the Clinical Use of Megestrol Acetate for Cancer-Related Anorexia/Cachexia[J]. Journal of Clinical Medicine, 2022,11(13).

[16] Wang T T, Yin J, Miller A H, et al. A systematic review of the association between fatigue and genetic polymorphisms[J]. BRAIN BEHAVIOR AND IMMUNITY, 2017,62:230-244.

[17] Li Z X, Mi D H, Wen Z Z. A SYSTEMATIC REVIEW OF THE ASSOCIATION BETWEEN THE INFLAMMATORY CYTOKINES POLYMORPHISMS AND CANCER- RELATED FATIGUE[J]. ACTA MEDICA MEDITERRANEA, 2017,33(1):41-47.

[18] Wilkinson S T, Radhakrishnan R, D'Souza D C. A systematic review of the evidence for medical marijuana in psychiatric indications[J]. Journal of Clinical Psychiatry, 2016,77(8):1050-1064.

[19] Bradfield J P, Vogelezang S, Felix J F, et al. A trans-ancestral meta-analysis of genome-wide association studies reveals loci associated with childhood obesity[J]. Human Molecular Genetics, 2019,28(19):3327-3338.

[20] Kaine J L. Abatacept for the treatment of rheumatoid arthritis: A review[J]. Current Therapeutic Research - Clinical and Experimental, 2007,68(6):379-399.

[21] Shani P, Walter E. Acceptability and Use of Mind-Body Interventions Among African American Cancer Survivors: An Integrative Review[J]. Integrative Cancer Therapies, 2022,21.

[22] Madsen M T, Huang C, Gögenur I. Actigraphy for measurements of sleep in relation to oncological treatment of patients with cancer: A systematic review[J]. Sleep Medicine Reviews, 2015,20:73-83.

[23] Milanti A, Chan D N S, Li C, et al. Actigraphy-measured rest-activity circadian rhythm disruption in patients with advanced cancer: a scoping review[J]. Supportive Care in Cancer, 2021,29(12):7145-7169.

[24] Wang F, Zhao J, Li Y, et al. Acupuncture and acupressure with improved cancer-related depression of retrospective studies[J]. Frontiers in Oncology, 2022,12.

[25] Lau C, Wu X, Chung V, et al. Acupuncture and Related Therapies for Symptom Management in Palliative Cancer Care: Systematic Review and Meta-Analysis[J]. Medicine (Baltimore), 2016,95(9):e2901.

[26] Lau C, Wu X Y, Chung V, et al. Acupuncture and Related Therapies for Symptom Management in Palliative Cancer Care Systematic Review and Meta-Analysis[J]. MEDICINE, 2016,95(9).

[27] Lau C H Y, Wu X, Chung V C H, et al. Acupuncture and related therapies for symptom management in palliative cancer care: Systematic review and meta-analysis[J]. Medicine (United States), 2016,95(9).

[28] Xi Z, Wei X, Ye Z, et al. Acupuncture for adult lung cancer of patient-reported outcomes: A systematic review and meta-analysis[J]. Front Oncol, 2022,12:921151.

[29] Xi Z, Wei X, Ye Z, et al. Acupuncture for adult lung cancer of patient-reported outcomes: A systematic review and meta-analysis[J]. Frontiers in Oncology, 2022,12.

[30] Gebbia V, Bellavia G, Ferra F, et al. Adherence, compliance and persistence to oral antineoplastic therapy: A review focused on chemotherapeutic and biologic agents[J]. Expert Opinion on Drug Safety, 2012,11(SUPPL. 1):S49-S59.

[31] Rockhill J K. Advances in radiation therapy for oncologic pain[J]. Current Pain and Headache Reports, 2007,11(4):270-275.

[32] Kaushal-Deep S, Raswan U, Kirmani A, et al. Alveolar soft part sarcoma metastasizing to the brain: A rare entity revisited with review of recent literature[J]. Journal of Pediatric Neurosciences, 2019,14(3):158-161.

[33] Culy C R, Spencer C M. Amifostine: An update on its clinical status as a cytoprotectant in patients with cancer receiving chemotherapy or radiotherapy and its potential therapeutic application in myelodysplastic syndrome[J]. Drugs, 2001,61(5):641-684.

[34] Ferreira P M P, Ferreira J R D O, Sousa R W R D, et al. Aminoquinolines as Translational Models for Drug Repurposing: Anticancer Adjuvant Properties and Toxicokinetic-Related Features[J]. Journal of Oncology, 2021,2021.

[35] Duan X, Zhao X, Wang S. An ALK-positive lung adenocarcinoma with gastric and skin metastasis: a case report and literature review[J]. Annals of palliative medicine, 2021,10(5):5797-5807.

[36] Capezuti E, Zadeh R S, Woody N, et al. An Integrative Review of Nonpharmacological Interventions to Improve Sleep among Adults with Advanced Serious Illness[J]. JOURNAL OF PALLIATIVE MEDICINE, 2018,21(5):700-717.

[37] Kumar A, Siwach A, Verma P. An Overview of the Synthetic Route to the Marketed Formulations of Pyrimidine: A Review[J]. Mini-Reviews in Medicinal Chemistry, 2022,22(6):884-903.

[38] Wolff J E. Anemia in placebo arms of cancer studies[J]. Anticancer Research, 2021,41(9):4543-4548.

[39] Li Q, Wu T, Jing L, et al. Angiogenesis inhibitors for the treatment of small cell lung cancer (SCLC): A meta-analysis of 7 randomized controlled trials[J]. Medicine (United States), 2017,96(13).

[40] Lima Bezerra J J, Johanes I, Vieira Pinheiro A A. Anticancer potential and toxicity of the genus Handroanthus Mattos (Bignoniaceae): A systematic review[J]. Toxicon, 2022,217:131-142.

[41] Ghimire P, Khanal U P, Gajurel B P, et al. Anti-LGI1, anti-GABABR, and Anti-CASPR2 encephalitides in Asia: A systematic review[J]. Brain and Behavior, 2020,10(10).

[42] Hou Y, Wang W, Bartolo P. Application of additively manufactured 3D scaffolds for bone cancer treatment: a review[J]. Bio-Design and Manufacturing, 2022,5(3):556-579.

[43] Ordóñez N G. Application of immunohistochemistry in the diagnosis of epithelioid mesothelioma: A review and update[J]. Human Pathology, 2013,44(1):1-19.

[44] Duivon M, Giffard B, Desgranges B, et al. Are Sleep Complaints Related to Cognitive Functioning in Non-Central Nervous System Cancer? A Systematic Review[J]. NEUROPSYCHOLOGY REVIEW, 2022,32(3):483-505.

[45] Winstock A R, Ford C, Witton J. Assessment and management of cannabis use disorders in primary care[J]. BMJ (Online), 2010,340(7750):800-804.

[46] Dun A S, Zhao X, Jin X, et al. Association Between Night-Shift Work and Cancer Risk: Updated Systematic Review and Meta-Analysis[J]. FRONTIERS IN ONCOLOGY, 2020,10.

[47] Teba P P, Esther M G, Raquel S G. Association between physical activity and patient-reported outcome measures in patients with lung cancer: a systematic review and meta-analysis[J]. QUALITY OF LIFE RESEARCH, 2022,31(7):1963-1976.

[48] Wang J, Yang H R, Wang D J, et al. Association between the gut microbiota and patient responses to cancer immune checkpoint inhibitors (Review)[J]. Oncology Letters, 2020,20(6).

[49] Tan B K J, Teo Y H, Tan N K W, et al. Association of obstructive sleep apnea and nocturnal hypoxemia with all-cancer incidence and mortality: a systematic review and meta-analysis[J]. Journal of Clinical Sleep Medicine, 2022,18(5):1427-1440.

[50] Lu H B, Ma R C, Yin Y Y, et al. Auricular Acupressure for Improving Sleep Quality in Patients With Lung Cancer A Systematic Review and Meta-analysis[J]. HOLISTIC NURSING PRACTICE, 2022,36(4):E27-E37.

[51] Lu H B, Ma R C, Yin Y Y, et al. Auricular Acupressure for Improving Sleep Quality in Patients With Lung Cancer: A Systematic Review and Meta-analysis[J]. Holist Nurs Pract, 2022,36(4):E27-E37.

[52] Lu H B, Ma R C, Yin Y Y, et al. Auricular Acupressure for Improving Sleep Quality in Patients With Lung Cancer: A Systematic Review and Meta-analysis[J]. Holistic nursing practice, 2022,36(4):E27-E37.

[53] Vu H L, Hoang T M N, Favier B, et al. Aurora kinases and passenger proteins as targets for cancer therapy: An update[J]. Current Enzyme Inhibition, 2010,6(1):19-25.

[54] Alexander S P H. Barriers to the wider adoption of medicinal Cannabis[J]. British Journal of Pain, 2020,14(2):122-132.

[55] Hanauer S B, Sandborn W J, Vakil N, et al. Best of DDW 2006[J]. Reviews in Gastroenterological Disorders, 2006,6(3):153-189.

[56] Grob N M, Aytekin M, Dweik R A. Biomarkers in exhaled breath condensate: a review of collection, processing and analysis[J]. JOURNAL OF BREATH RESEARCH, 2008,2(3).

[57] Rowbottom L, Chan S, Wan B A, et al. Biomarkers relating to cancer-related symptom burden and quality of life[J]. Journal of Pain Management, 2017,10(3):237-253.

[58] Navarro-Olvera J L, Ariñez-Barahona E, Esqueda-Liquidano M A, et al. Brain metastases: Literature review[J]. Revista Medica del Hospital General de Mexico, 2017,80(1):60-66.

[59] Jeklin A T, Alamgeer M, Stirling R G, et al. Burning the Candle at Both Ends-Sleep Quality Before and After Chemotherapy in Lung Cancer Patients - A Systematic Review and Meta-Analysis[J]. JOURNAL OF THORACIC ONCOLOGY, 2022,17(9):S136-S137.

[60] Charalambous A, Berger A M, Matthews E, et al. Cancer-related fatigue and sleep deficiency in cancer care continuum: concepts, assessment, clusters, and management[J]. SUPPORTIVE CARE IN CANCER, 2019,27(7):2747-2753.

[61] Mitchell S A, Berger A M. Cancer-related fatigue: The evidence base for assessment and management[J]. Cancer Journal, 2006,12(5):374-387.

[62] Andrade C. Cannabis and neuropsychiatry, 1: Benefits and risks[J]. Journal of Clinical Psychiatry, 2016,77(5):e551-e554.

[63] Rosewall T, Feuz C, Bayley A. Cannabis and Radiation Therapy: A Scoping Review of Human Clinical Trials[J]. Journal of Medical Imaging and Radiation Sciences, 2020,51(2):342-349.

[64] Cheng S, Evans W K, Stys-Norman D, et al. Chemotherapy for relapsed small cell lung cancer: A systematic review and practice guideline[J]. Journal of Thoracic Oncology, 2007,2(4):348-354.

[65] Zhou H, Zeng C, Wang L Y, et al. Chemotherapy with or without gefitinib in patients with advanced non-small-cell lung cancer: A meta-analysis of 6844 patients[J]. Chinese Medical Journal, 2013,126(17):3348-3355.

[66] Almaida-Pagan P F, Torrente M, Campos M, et al. Chronodisruption and Ambulatory Circadian Monitoring in Cancer Patients: Beyond the Body Clock[J]. CURRENT ONCOLOGY REPORTS, 2022,24(2):135-149.

[67] Hrushesky W J M, Grutsch J, Wood P, et al. Circadian clock manipulation for cancer prevention and control and the relief of cancer symptoms[J]. Integrative Cancer Therapies, 2009,8(4):387-397.

[68] Ahabrach H, El Mlili N, Errami M, et al. Circadian rhythm and concentration of melatonin in breast cancer patients[J]. Endocrine, Metabolic and Immune Disorders - Drug Targets, 2021,21(10):1869-1881.

[69] Wilking M, Ndiaye M, Mukhtar H, et al. Circadian Rhythm Connections to Oxidative Stress: Implications for Human Health[J]. ANTIOXIDANTS & REDOX SIGNALING, 2013,19(2):192-208.

[70] Papagiannakopoulos T, Bauer M R, Davidson S M, et al. Circadian Rhythm Disruption Promotes Lung Tumorigenesis[J]. Cell Metab, 2016,24(2):324-331.

[71] Bollinger T, Schibler U. Circadian rhythms - From genes to physiology and disease[J]. Swiss Medical Weekly, 2014,144.

[72] Zhou L, Zhang Z, Nice E, et al. Circadian rhythms and cancers: the intrinsic links and therapeutic potentials[J]. JOURNAL OF HEMATOLOGY & ONCOLOGY, 2022,15(1).

[73] Huang C, Madsen M T, Gögenur I. Circadian rhythms measured by actigraphy during oncological treatments: A systematic review[J]. Biological Rhythm Research, 2015,46(3):329-348.

[74] Liu J, Shi Y, Liu X, et al. Clinical characteristics and outcomes of immune checkpoint inhibitor-induced diabetes mellitus[J]. Translational Oncology, 2022,24.

[75] Kuo C C, Wang C C, Chang W L, et al. Clinical Effects of Baduanjin Qigong Exercise on Cancer Patients: A Systematic Review and Meta-Analysis on Randomized Controlled Trials[J]. Evidence-based Complementary and Alternative Medicine, 2021,2021.

[76] Misra R N. Clinical progress of selective cyclin-dependent kinase (CDK) inhibitors[J]. Drugs of the Future, 2006,31(1):43-52.

[77] Agnieszka W, Fiammetta C. Clinical utility of demoralization: A systematic review of the literature[J]. Clinical Psychology Review, 2023,99.

[78] Hasegawa H, Jentoft M E, Young W F, et al. Collision of Craniopharyngioma and Pituitary Adenoma: Comprehensive Review of an Extremely Rare Sellar Condition[J]. World Neurosurgery, 2021,149:e51-e62.

[79] Raghu G, Amatto V C, Behr J, et al. Comorbidities in idiopathic pulmonary fibrosis patients: a systematic literature review[J]. Eur Respir J, 2015,46(4):1113-1130.

[80] Raghu G, Amatto V C, Behr J, et al. Comorbidities in idiopathic pulmonary fibrosis patients: a systematic literature review[J]. EUROPEAN RESPIRATORY JOURNAL, 2015,46(4):1113-1130.

[81] Nieder C, Kämpe T A. Contribution of patient-reported symptoms before palliative radiotherapy to development of multivariable prognostic models[J]. Anticancer Research, 2018,38(3):1705-1709.

[82] Davidson D. CORR Insights (R): Which Bone-Modifying Agent is Associated with Better Outcomes in Patients with Metastatic Bone Disease from Lung Cancer? A Systematic Review and Network Meta-Analysis[J]. CLINICAL ORTHOPAEDICS AND RELATED RESEARCH, 2021,479(9):2058-2060.

[83] Huang Z H, Yu T, Wu S Y, et al. Correlates of stigma for patients with cancer: a systematic review and meta-analysis[J]. SUPPORTIVE CARE IN CANCER, 2021,29(3):1195-1203.

[84] Tayyar Y, Jubair L, Fallaha S, et al. Critical risk-benefit assessment of the novel anti-cancer aurora a kinase inhibitor alisertib (MLN8237): A comprehensive review of the clinical data[J]. Critical Reviews in Oncology/Hematology, 2017,119:59-65.

[85] Guo Z L, Li M X, Li X L, et al. Crocetin: A Systematic Review[J]. Frontiers in Pharmacology, 2021,12.

[86] Stigliano A, Cerquetti L, Sampaoli C, et al. Current and emerging therapeutic options in adrenocortical cancer treatment[J]. Journal of Oncology, 2012.

[87] Chung K F. Current and future prospects for drugs to suppress cough[J]. IDrugs, 2003,6(8):781-786.

[88] Krueger G G. Current concepts and review of alefacept in the treatment of psoriasis[J]. Dermatologic Clinics, 2004,22(4):407-426.

[89] Berger A M, Sankaranarayanan J, Watanabe-Galloway S. Current methodological approaches to the study of sleep disturbances and quality of life in adults with cancer: A systematic review[J]. PSYCHO-ONCOLOGY, 2007,16(5):401-420.

[90] Daily E B, Aquilante C L. Cytochrome P450 2C8 pharmacogenetics: A review of clinical studies[J]. Pharmacogenomics, 2009,10(9):1489-1510.

[91] Hu X, Chen Y, Ru G, et al. Cytological Features of Pulmonary Papillary Adenoma with Malignant Transformation and Literature Review[J]. Analytical Cellular Pathology, 2020,2020.

[92] Storage S S, Agrawal H, Furst D E. Description of the efficacy and safety of three new biologics in the treatment of rheumatoid arthritis[J]. Korean Journal of Internal Medicine, 2010,25(1):1-17.

[93] Sachdeva H, Saquib M, Tanwar K. Design and Development of Triazole Derivatives as Prospective Anticancer Agents: A Review[J]. Anti-Cancer Agents in Medicinal Chemistry, 2022,22(19):3269-3279.

[94] DSilva F, Singh P, Javeth A. Determinants of Cancer-Related Fatigue among Cancer Patients: A Systematic Review[J]. JOURNAL OF PALLIATIVE CARE.

[95] Yang G Y, Hunter J, Bu F L, et al. Determining the safety and effectiveness of Tai Chi: a critical overview of 210 systematic reviews of controlled clinical trials[J]. Systematic Reviews, 2022,11(1).

[96] Liu W, Zhou L, Zhao D, et al. Development and Validation of a Prognostic Nomogram in Lung Cancer With Obstructive Sleep Apnea Syndrome[J]. FRONTIERS IN MEDICINE, 2022,9.

[97] Kyritsis A P, Markoula S, Alexiou G, et al. Diagnosis and treatment of limbic encephalitis in the cancer patient[J]. Future Oncology, 2020,16(22):1647-1655.

[98] Layfield L J, Pearson L, Walker B S, et al. Diagnostic Accuracy of Fine-Needle Aspiration Cytology for Discrimination of Squamous Cell Carcinoma from Adenocarcinoma in Non-Small Cell Lung Cancer: A Systematic Review and Meta-Analysis[J]. ACTA CYTOLOGICA, 2018,62(5-6):318-326.

[99] Valabrega G, Scotto G, Tuninetti V, et al. Differences in parp inhibitors for the treatment of ovarian cancer: Mechanisms of action, pharmacology, safety, and efficacy[J]. International Journal of Molecular Sciences, 2021,22(8).

[100] Gonzalez McQuire S, Kucmin-Bemelmans I, Hensen M. Disease burden of idiopathic chronic cough (ICC) and chronic cough (CC) in COPD, IPF and lung cancer (LC): An exploratory literature review[J]. Value in Health, 2013,16(3):A231.

[101] Paulsen Ø, Aass N, Kaasa S, et al. Do corticosteroids provide analgesic effects in cancer patients? A systematic literature review[J]. Journal of Pain and Symptom Management, 2013,46(1):96-105.

[102] Cortés-Jofré M, Rueda J R, Asenjo-Lobos C, et al. Drugs for preventing lung cancer in healthy people[J]. Cochrane Database of Systematic Reviews, 2020,2020(3).

[103] Ruaro B, Baratella E, Confalonieri M, et al. Editorial: Obstructive sleep apnea syndrome (OSAS). What's new?[J]. Frontiers in Medicine, 2022,9.

[104] Divani A, Heidari M E, Ghavampour N, et al. Effect of cancer treatment on sleep quality in cancer patients: A systematic review and meta-analysis of Pittsburgh Sleep Quality Index[J]. SUPPORTIVE CARE IN CANCER, 2022,30(6):4687-4697.

[105] Xiao P, Ding S, Duan Y, et al. Effect of Light Therapy on Cancer-Related Fatigue: A Systematic Review and Meta-Analysis[J]. Journal of Pain and Symptom Management, 2022,63(2):e188-e202.

[106] Takemura N, Cheung D, Smith R, et al. Effectiveness of aerobic exercise and mind-body exercise in cancer patients with poor sleep quality: A systematic review and meta-analysis of randomized controlled trials[J]. SLEEP MEDICINE REVIEWS, 2020,53.

[107] Yokoe T, Hayashida T, Nagayama A, et al. Effectiveness of Antiemetic Regimens for Highly Emetogenic Chemotherapy-Induced Nausea and Vomiting: A Systematic Review and Network Meta-Analysis[J]. Oncologist, 2019,24(6):e347-e357.

[108] Lavin-Perez A M, Collado-Mateo D, Mayo X, et al. Effects of high-intensity training on the quality of life of cancer patients and survivors: a systematic review with meta-analysis[J]. SCIENTIFIC REPORTS, 2021,11(1).

[109] Yang M, Liu L, Gan C E, et al. Effects of home-based exercise on exercise capacity, symptoms, and quality of life in patients with lung cancer: A meta-analysis[J]. Eur J Oncol Nurs, 2020,49:101836.

[110] Yang M, Liu L, Gan C E, et al. Effects of home-based exercise on exercise capacity, symptoms, and quality of life in patients with lung cancer: A meta-analysis[J]. European journal of oncology nursing : the official journal of European Oncology Nursing Society, 2020,49:101836.

[111] Hong Y, Li X, Wan B, et al. Efficacy and Safety of Eltrombopag for Aplastic Anemia: A Systematic Review and Meta-analysis[J]. Clinical Drug Investigation, 2019,39(2):141-156.

[112] Luo N, Tan S, Li X, et al. Efficacy and Safety of Opioids in Treating Cancer-Related Dyspnea: A Systematic Review and Meta-Analysis Based on Randomized Controlled Trials[J]. Journal of Pain and Symptom Management, 2021,61(1):198-210.

[113] Li L, Huang Q, Sun J, et al. Efficacy and safety of osimertinib for patients with EGFR-mutated NSCLC: a systematic review and meta-analysis of randomized controlled studies[J]. Acta Oncologica, 2022,61(11):1347-1353.

[114] Xie J, Zhang C, Li S, et al. Efficacy and Safety of Thalidomide As a Pre-Medication of Chemotherapy-Induced Nausea and Vomiting (CINV) Following Highly Emetogenic Chemotherapy (HEC): A Systematic Review and Meta-Analysis[J]. Frontiers in Oncology, 2022,11.

[115] Heywood R, McCarthy A L, Skinner T L. Efficacy of Exercise Interventions in Patients With Advanced Cancer: A Systematic Review[J]. Archives of Physical Medicine and Rehabilitation, 2018,99(12):2595-2620.

[116] Chan E W C, Wong C W, Wong S K, et al. Emodin and shikonin (quinones): An overview of their chemistry, plant sources, pharmacology and cytotoxic activities against lung cancer[J]. Journal of Chinese Pharmaceutical Sciences, 2020,29(1):1-12.

[117] Prasad K T, Sehgal I S, Gupta N, et al. Endoscopic ultrasound (with an echobronchoscope)-guided fine-needle aspiration for diagnosis of a mediastinal lesion in a mechanically ventilated patient: A case report and systematic review of the literature[J]. Indian Journal of Critical Care Medicine, 2016,20(10):608-612.

[118] Nervi C, De Marinis E, Codacci-Pisanelli G. Epigenetic treatment of solid tumours: A review of clinical trials[J]. Clinical Epigenetics, 2015,7(1).

[119] Ftanou M. ES15.03 Managing Sleep Difficulties and Cancer[J]. 2019,14(10):S52.

[120] Quist M. ES26.05 Early Intervention and Rehabilitation for Patients Newly Diagnosed with Thoracic Malignancies[J]. Journal of Thoracic Oncology, 2019,14(10):S75-S76.

[121] Di Martile M, Garzoli S, Ragno R, et al. Essential oils and their main chemical components: The past 20 years of preclinical studies in Melanoma[J]. Cancers, 2020,12(9):1-45.

[122] Woolacott N, Hawkins N, Mason A, et al. Etanercept and efalizumab for the treatment of psoriasis: A systematic review[J]. Health Technology Assessment, 2006,10(46):90.

[123] Manheimer E, Wieland S, Kimbrough E, et al. Evidence from the cochrane collaboration for traditional chinese medicine therapies[J]. Journal of Alternative and Complementary Medicine, 2009,15(9):1001-1014.

[124] Singh B, Spence R, Steele M L, et al. Exercise for Individuals With Lung Cancer: A Systematic Review and Meta-Analysis of Adverse Events, Feasibility, and Effectiveness[J]. Seminars in oncology nursing, 2020,36(5):151076.

[125] Dittus K L, Gramling R E, Ades P A. Exercise interventions for individuals with advanced cancer: A systematic review[J]. Preventive Medicine, 2017,104:124-132.

[126] Lewis P, Korf H W, Kuffer L, et al. Exercise time cues (zeitgebers) for human circadian systems can foster health and improve performance: a systematic review[J]. BMJ OPEN SPORT & EXERCISE MEDICINE, 2018,4(1).

[127] Chen Y J, Li X X, Ma H K, et al. Exercise Training for Improving Patient-Reported Outcomes in Patients With Advanced-Stage Cancer: A Systematic Review and Meta-Analysis[J]. JOURNAL OF PAIN AND SYMPTOM MANAGEMENT, 2020,59(3):734.

[128] Ligibel J A, Bohlke K, May A M, et al. Exercise, Diet, and Weight Management during Cancer Treatment: ASCO Guideline[J]. Journal of Clinical Oncology, 2022,348.

[129] Carnio S, Di Stefano R F, Novello S. Fatigue in lung cancer patients: Symptom burden and management of challenges[J]. Lung Cancer: Targets and Therapy, 2016,7:73-82.

[130] Naguy A, Husain K, Alamiri B. Galantamine beyond Alzheimer's disease - A fact or artefact?[J]. CNS Spectrums, 2022,27(3):268-271.

[131] Ahmad M F. Ganoderma lucidum: A rational pharmacological approach to surmount cancer[J]. Journal of Ethnopharmacology, 2020,260.

[132] Williams L, Cleeland C, Bamidele O, et al. Generation of symptom burden patient-reported outcomes for patients with lung cancer[J]. Journal of Thoracic Oncology, 2017,12(11):S2024.

[133] Schreiner C A. Genetic toxicity of naphthalene: A review[J]. Journal of Toxicology and Environmental Health - Part B: Critical Reviews, 2003,6(2):161-183.

[134] Fritz H, Seely D, Kennedy D A, et al. Green tea and lung cancer: A systematic review[J]. Integrative Cancer Therapies, 2013,12(1):7-24.

[135] Spada G E, Masiero M, Pizzoli S, et al. Heart Rate Variability Biofeedback in Cancer Patients: A Scoping Review[J]. BEHAVIORAL SCIENCES, 2022,12(10).

[136] Talwar A, Sharma A, Jamil S F, et al. Hepatoid variant of primary adenocarcinoma lung: A series of four cases with review of literature[J]. Indian Journal of Pathology and Microbiology, 2019,62(5):S95.

[137] Arslan D, Tural D, Akar E. Herbal administration and interaction of cancer treatment[J]. Journal of Palliative Medicine, 2013,16(11):1466-1476.

[138] Herbal medicine[J]. Focus on Alternative and Complementary Therapies, 2005,10(3):222.

[139] Sermer D J, Woodley J L, Thomas C A, et al. Herpes simplex encephalitis as a complication of whole-brain radiotherapy: A case report and review of the literature[J]. Case Reports in Oncology, 2014,7(3):774-779.

[140] Wagner J M, Hackanson B, Lübbert M, et al. Histone deacetylase (HDAC) inhibitors in recent clinical trials for cancer therapy[J]. Clinical Epigenetics, 2010,1(3-4):117-136.

[141] Marks P A, Richon V M, Miller T, et al. Histone deacetylase inhibitors[Z]. 2004: 91, 137-168.

[142] Somech R, Izraeli S, Simon A J. Histone deacetylase inhibitors - A new tool to treat cancer[J]. Cancer Treatment Reviews, 2004,30(5):461-472.

[143] Piekarz R L, Sackett D L, Bates S E. Histone deacetylase inhibitors and demethylating agents: Clinical development of histone deacetylase inhibitors for cancer therapy[J]. Cancer Journal, 2007,13(1):30-39.

[144] Rasheed W K, Johnstone R W, Prince H M. Histone deacetylase inhibitors in cancer therapy[J]. Expert Opinion on Investigational Drugs, 2007,16(5):659-678.

[145] Rasheed W, Bishton M, Johnston R W, et al. Histone deacetylase inhibitors in lymphoma and solid malignancies[J]. Expert Review of Anticancer Therapy, 2008,8(3):413-432.

[146] Bagella L, Federico M. Histone deacetylase inhibitors in the treatment of hematological malignancies and solid tumors[J]. Journal of Biomedicine and Biotechnology, 2011,2011.

[147] Ma X, Ezzeldin H H, Diasio R B. Histone deacetylase inhibitors: Current status and overview of recent clinical trials[J]. Drugs, 2009,69(14):1911-1934.

[148] Choo Q Y, Ho P C, Lin H S. Histone deacetylase inhibitors: New hope for rheumatoid arthritis?[J]. Current Pharmaceutical Design, 2008,14(8):803-820.

[149] Jeffus S K, Gardner J M, Steliga M A, et al. Hyalinizing clear cell carcinoma of the lung: Case report and review of the literature[J]. American Journal of Clinical Pathology, 2017,148(1):73-80.

[150] Chew C Y, Mar A, Nikpour M, et al. Hydroxychloroquine in dermatology: New perspectives on an old drug[J]. Australasian Journal of Dermatology, 2020,61(2):e150-e157.

[151] Bradley P J, Hoskin D. Hypercalcaemia in head and neck squamous cell carcinoma[J]. Current Opinion in Otolaryngology and Head and Neck Surgery, 2006,14(2):51-54.

[152] Dong X, Zeng Y W, Zhang Z Q, et al. Hypericin-mediated photodynamic therapy for the treatment of cancer: a review[J]. JOURNAL OF PHARMACY AND PHARMACOLOGY, 2021,73(4):425-436.

[153] Dong X, Zeng Y, Zhang Z, et al. Hypericin-mediated photodynamic therapy for the treatment of cancer: A review[J]. Journal of Pharmacy and Pharmacology, 2021,73(4):425-436.

[154] Mah P M, Webster J. Hyperprolactinemia: Etiology, diagnosis, and management[J]. Seminars in Reproductive Medicine, 2002,20(4):365-373.

[155] Ramalingam S S, Shaw A T. Hypogonadism related to crizotinib therapy: Implications for patient care[J]. Cancer, 2012,118(21):E1-E2.

[156] Rhomberg L R, Bailey L A, Goodman J E. Hypothesis-based weight of evidence: A tool for evaluating and communicating uncertainties and inconsistencies in the large body of evidence in proposing a carcinogenic mode of actionnaphthalene as an example[J]. Critical Reviews in Toxicology, 2010,40(8):671-696.

[157] Ji Q, Ding J, Hao M, et al. Immune Checkpoint Inhibitors Combined With Chemotherapy Compared With Chemotherapy Alone for Triple-Negative Breast Cancer: A Systematic Review and Meta-Analysis[J]. Frontiers in Oncology, 2021,11.

[158] Stelow E B, Yaziji H. Immunohistochemistry, carcinomas of unknown primary, and incidence rates[J]. Seminars in Diagnostic Pathology, 2018,35(2):95-107.

[159] Lee K, Cho M, Miaskowski C, et al. Impaired sleep and rhythms in persons with cancer[J]. Sleep Medicine Reviews, 2004,8(3):199-212.

[160] Wu J, Sin D D. Improved patient outcome with smoking cessation: When is it too late?[J]. International Journal of COPD, 2011,6(1):259-267.

[161] Alejos R M, Harrison S L, Crespo A, et al. In the spotlight: Early career member awardee 2020, ers lung science conference 2021, and sleep and breathing 2021[J]. Breathe, 2020,16(4):1-5.

[162] Witlox W J A, Ramaekers B L T, Lacas B, et al. Individual patient data meta-analysis of prophylactic cranial irradiation in locally advanced non-small cell lung cancer[J]. Radiotherapy and Oncology, 2021,158:40-47.

[163] Jung W, Kwon S, Im J, et al. Influence of herbal complexes containing licorice on potassium levels: A retrospective study[J]. Evidence-based Complementary and Alternative Medicine, 2014,2014.

[164] Kienle G S, Kiene H. Influence of Viscum album L (European Mistletoe) Extracts on Quality of Life in Cancer Patients: A Systematic Review of Controlled Clinical Studies[J]. Integrative Cancer Therapies, 2010,9(2):142-157.

[165] Chicoisneau M, Paesmans M, Ameye L, et al. Initiation of a new anti-cancer medical treatment in ICU: a retrospective study[J]. Acta Clinica Belgica: International Journal of Clinical and Laboratory Medicine, 2022,77(2):337-345.

[166] Kaplow R. Innovations in antineoplastic therapy[J]. Nursing Clinics of North America, 2005,40(1):77-94.

[167] Kiss I, Kuhn M, Hrusak K, et al. Insomnia in patients treated with checkpoint inhibitors for cancer: A meta-analysis[J]. Frontiers in Oncology, 2022,12.

[168] Savard J, Morin C M. Insomnia in the context of cancer: A review of a neglected problem[J]. JOURNAL OF CLINICAL ONCOLOGY, 2001,19(3):895-908.

[169] Kut V, Boasberg P, Sagar S, et al. Integrative tumor board: Advanced breast cancer[J]. Integrative Cancer Therapies, 2003,1(2):179-214.

[170] Makinson A, Pujol J L, Le Moing V, et al. Interactions between cytotoxic chemotherapy and antiretroviral treatment in human immunodeficiency virus-infected patients with lung cancer[J]. Journal of Thoracic Oncology, 2010,5(4):562-571.

[171] Woll P J, Pettengell R. Interferons in oncology[J]. British Journal of Clinical Practice, 1997,51(2):111-115.

[172] Gabay M P, Thakkar J P, Stachnik J M, et al. Intra-CSF administration of chemotherapy medications[J]. Cancer Chemotherapy and Pharmacology, 2012,70(1):1-15.

[173] Graziani G, Tentori L, Navarra P. Ipilimumab: A novel immunostimulatory monoclonal antibody for the treatment of cancer[J]. Pharmacological Research, 2012,65(1):9-22.

[174] Harris B, Sanchez-Reilly S, Lee S, et al. Is it really worth undergoing treatment? Exploring quality of life variables among older adults with lung cancer[J]. Journal of Pain and Symptom Management, 2015,49(2):430.

[175] Fishbain D A, Cole B, Cutler R B, et al. Is pain fatiguing? A structured evidence-based review[J]. PAIN MEDICINE, 2003,4(1):51-62.

[176] Carpinelli P, Moll J. Is there a future for Aurora kinase inhibitors for anticancer therapy?[J]. Current Opinion in Drug Discovery and Development, 2009,12(4):533-542.

[177] Schwartz R A, Micali G, Nasca M R, et al. Kaposi sarcoma: A continuing conundrum[J]. Journal of the American Academy of Dermatology, 2008,59(2):179-206.

[178] Bredlau A L, Thakur R, Korones D N, et al. Ketamine for Pain in Adults and Children with Cancer: A Systematic Review and Synthesis of the Literature[J]. Pain Medicine (United States), 2013,14(10):1505-1517.

[179] He D, Han K, Gao X, et al. Laquinimod for multiple sclerosis[J]. Cochrane Database of Systematic Reviews, 2013,2013(8).

[180] Galustian C, Dalgleish A. Lenalidomide: A novel anticancer drug with multiple modalities[J]. Expert Opinion on Pharmacotherapy, 2009,10(1):125-133.

[181] Daffonchio L, Clavenna G, Fedele G, et al. Levodropropizine[J]. Drugs of Today, 1995,31(5):299-305.

[182] Anderson N E, Barber P A. Limbic encephalitis - a review[J]. JOURNAL OF CLINICAL NEUROSCIENCE, 2008,15(9):961-971.

[183] Morita A, Kamei S. Limbic encephalitis with antibodies against intracellular antigens[J]. Brain and Nerve, 2010,62(4):347-355.

[184] Nicolson G L. Lipid replacement therapy: A nutraceutical approach for reducing cancer-associated fatigue and the adverse effects of cancer therapy while restoring mitochondrial function[J]. Cancer and Metastasis Reviews, 2010,29(3):543-552.

[185] Milano G, Innocenti F, Minami H. Liposomal irinotecan (Onivyde): Exemplifying the benefits of nanotherapeutic drugs[J]. Cancer Science, 2022,113(7):2224-2231.

[186] Davis M P, Goforth H W. Long-term and Short-term Effects of Insomnia in Cancer and Effective Interventions[J]. CANCER JOURNAL, 2014,20(5):330-344.

[187] Zhong Y J, Zhang Q H, Deng W J, et al. Long-term survival for 93 months of limited-stage small cell lung cancer: A case report and literature review[J]. THORACIC CANCER, 2014,5(4):349-353.

[188] Caviglia R, Boškoski I, Cicala M. Long-term treatment with infliximab in inflammatory bowel disease: Safety and tolerability issues[J]. Expert Opinion on Drug Safety, 2008,7(5):617-632.

[189] Rowntree R A, Hosseinzadeh H. Lung Cancer and Self-Management Interventions: A Systematic Review of Randomised Controlled Trials[J]. Int J Environ Res Public Health, 2022,19(1).

[190] Rowntree R A, Hosseinzadeh H. Lung Cancer and Self-Management Interventions: A Systematic Review of Randomised Controlled Trials[J]. International Journal of Environmental Research and Public Health, 2022,19(1).

[191] Lang S M, Täuscher D, Schiffl H. Lung cancer in elderly patients[J]. Atemwegs- und Lungenkrankheiten, 2016,42(5):264-272.

[192] Ahuja R, Weibel S B, Leone F T. Lung cancer: The oncologist's role in smoking cessation[J]. Seminars in Oncology, 2003,30(1):94-103.

[193] White J, Kearins O, Dodwell D, et al. Male breast carcinoma: Increased awareness needed[J]. Breast Cancer Research, 2011,13(5).

[194] Donthireddy K R, Ailawadhi S, Nasser E, et al. Malignant gastroparesis: Pathogenesis and management of an underrecognized disorder[J]. Journal of Supportive Oncology, 2007,5(8):355-363.

[195] Bedor M, Alexander C, Edelman M J. Management of common symptoms of advanced lung cancer[J]. Current Treatment Options in Oncology, 2005,6(1):61-68.

[196] Warr D. Management of highly emetogenic chemotherapy[J]. Current Opinion in Oncology, 2012,24(4):371-375.

[197] Hallak B, Bouayed S, Ghika J A, et al. Management Strategy of Intracranial Complications of Sinusitis: Our Experience and Review of the Literature[J]. Allergy and Rhinology, 2022,13.

[198] Marino J L, McNamara H C, Hickey M. Managing menopausal symptoms after cancer: An evidence-based approach for primary care[J]. Medical Journal of Australia, 2018,208(3):127-132.

[199] Haque N, Parveen S, Tang T, et al. Marine Natural Products in Clinical Use[J]. Marine Drugs, 2022,20(8).

[200] Chen D, Yin Z, Fang B. Measurements and status of sleep quality in patients with cancers[J]. Supportive Care in Cancer, 2018,26(2):405-414.

[201] Nguyen T D, Bordeau B M, Balthasar J P. Mechanisms of ADC Toxicity and Strategies to Increase ADC Tolerability[J]. Cancers, 2023,15(3).

[202] Tritos N A, Biller B M K. Medical Management of Cushing Disease[J]. Neurosurgery Clinics of North America, 2019,30(4):499-508.

[203] Kramer J L. Medical marijuana for cancer[J]. CA Cancer Journal for Clinicians, 2015,65(2):110-122.

[204] De Sousa Monteiro L, Bastos K X, Barbosa-Filho J M, et al. Medicinal plants and other living organisms with antitumor potential against lung cancer[J]. Evidence-based Complementary and Alternative Medicine, 2014,2014.

[205] Vijaya Bhargava K. Medicinal uses and pharmacological properties of Crocus sativus linn (saffron)[J]. International Journal of Pharmacy and Pharmaceutical Sciences, 2011,3(SUPPL. 3):22-26.

[206] Jarius S, Wildemann B. 'Medusa head ataxia': The expanding spectrum of Purkinje cell antibodies in autoimmune cerebellar ataxia. Part 2: Anti-PKC-gamma, anti-GluR-delta2, anti-Ca/ARHGAP26 and anti-VGCC[J]. Journal of Neuroinflammation, 2015,12(1).

[207] Abraham R, Basser R L. Megakaryocyte growth and development factor: A review of early clinical studies[J]. Oncologist, 1997,2(5):311-318.

[208] Pandi-Perumal S R, Srinivasan V, Maestroni G J M, et al. Melatonin: Nature's most versatile biological signal?[J]. FEBS Journal, 2006,273(13):2813-2838.

[209] Leelaviwat N, Mekraksakit P, Cross K M, et al. Melatonin: Translation of Ongoing Studies Into Possible Therapeutic Applications Outside Sleep Disorders[J]. Clinical Therapeutics, 2022,44(5):783-812.

[210] Zhang G Q, Chen J L, Luo Y, et al. Menopausal hormone therapy and women's health: An umbrella review[J]. PLoS Medicine, 2021,18(8).

[211] Archer D F, Sturdee D W, Baber R, et al. Menopausal hot flushes and night sweats: Where are we now?[J]. Climacteric, 2011,14(5):515-528.

[212] Chen D, Xie X, Gao Y. Meta-analysis of randomized clinical trials of thalidomide-chemotherapy combination regimen for non-small cell lung cancer patients in China[J]. Chinese Journal of Clinical Oncology, 2012,39(22):1818-1823.

[213] Varlotto J M, Bosetti C, Bronson D, et al. Meta-analysis of rates and causes of local recurrence in surgically-resected NSCLC and differences in recurrence and survival between Asian and non-Asian populations[J]. Journal of Clinical Oncology, 2022,40(16).

[214] Lim S J M, Iyer N G, Ooi L L, et al. Metastasectomy for metachronous pulmonary and hepatic metastases from nasopharyngeal carcinoma: Report of 6 cases and review of the literature[J]. Head and Neck, 2016,38(2):E37-E40.

[215] Serpico R, Brown J, Blank A, et al. Metastasis of Osteosarcoma to the Abdomen: A Report of Two Cases and a Review of the Literature[J]. Case Reports in Oncology, 2021,14(1):647-658.

[216] Sumida L C, Chen D, Kaneshiro J, et al. Metastatic papillary thyroid carcinoma diagnosed by pleural effusion cytology: A report of cases and literature review[J]. American Journal of Clinical Pathology, 2013,140:A7.

[217] Gholami S K, Santiago C, Bhojaraja V S, et al. MIND-BODY THERAPIES AND ITS EFFECT ON THE IMMUNE SYSTEM IN CHRONIC DISEASES: A LITERATURE REVIEW[J]. Journal of Health and Translational Medicine, 2022,25(1):97-107.

[218] McDonnell K K, Owens O L, Umari F. Mindfulness-Based Interventions for Survivors of Lung Cancer and Their Partners: A Systematic Review[J]. International journal of behavioral medicine, 2022.

[219] Xie C Y, Dong B, Wang L H, et al. Mindfulness-based stress reduction can alleviate cancer- related fatigue: A meta-analysis[J]. JOURNAL OF PSYCHOSOMATIC RESEARCH, 2020,130.

[220] Tashiro M, Fukuda H, Itoh M, et al. Molecular imaging at Tohoku University: From cancer to neuroreceptors[J]. Current Medical Imaging Reviews, 2008,4(1):8-13.

[221] Walker W H, Borniger J C. Molecular mechanisms of cancer-induced sleep disruption[J]. International Journal of Molecular Sciences, 2019,20(11).

[222] Dudek A Z, Pawlak W Z, Kirstein Pharm M N. Molecular targets in the inhibition of angiogenesis[J]. Expert Opinion on Therapeutic Targets, 2003,7(4):527-541.

[223] Wilhelm C, Neubauer A. Molecular tumour therapy. Antibodies and small molecules[J]. Internist, 2008,49(5):581-592.

[224] Siebert J C, Walker E B. Monitoring cytokine profiles during immunotherapy[J]. Immunotherapy, 2010,2(6):799-816.

[225] Baughman R P, Meyer K C, Nathanson I, et al. Monitoring of nonsteroidal immunosuppressive drugs in patients with lung disease and lung transplant recipients: American College of Chest Physicians evidence-based clinical practice guidelines[J]. Chest, 2012,142(5):e1S-e111S.

[226] Schwenk E S, Gupta R K. Mortality associated with long-term opioid use after lung cancer surgery: An infographic[J]. Regional Anesthesia and Pain Medicine, 2022,47(11):684.

[227] Takahashi H, Ikeda M, Kumada T, et al. Multicenter cooperative case survey of hepatitis B virus reactivation by chemotherapeutic agents[J]. Hepatology Research, 2015,45(12):1220-1227.

[228] Basarkar V, Govardhane S, Shende P. Multifaceted Applications of Genetically Modified Micro-organisms: A Biotechno-logical Revolution[J]. Current Pharmaceutical Design, 2022,28(22):1822-1842.

[229] Sudeshna G, Parimal K. Multiple non-psychiatric effects of phenothiazines: A review[J]. European Journal of Pharmacology, 2010,648(1-3):6-14.

[230] Crawford J, Armitage J, Balducci L, et al. Myeloid growth factors[J]. JNCCN Journal of the National Comprehensive Cancer Network, 2013,11(10):1266-1290.

[231] Schwalfenberg G K. N-Acetylcysteine: A Review of Clinical Usefulness (an Old Drug with New Tricks)[J]. Journal of Nutrition and Metabolism, 2021,2021.

[232] Pramono A A, Rather G M, Herman H, et al. NAD-and NADPH-contributing enzymes as therapeutic targets in cancer: An overview[J]. Biomolecules, 2020,10(3).

[233] Kamal A, Bolla N R, Srikanth P S, et al. Naphthalimide derivatives with therapeutic characteristics: A patent review[J]. Expert Opinion on Therapeutic Patents, 2013,23(3):299-317.

[234] Ingrassia L, Lefranc F, Kiss R, et al. Naphthalimides and azonafides as promising anti-cancer agents[J]. Current Medicinal Chemistry, 2009,16(10):1192-1213.

[235] Ahmadi E S, Tajbakhsh A, Iranshahy M, et al. Naphthoquinone derivatives isolated from plants: Recent advances in biological activity[J]. Mini-Reviews in Medicinal Chemistry, 2020,20(19):2019-2035.

[236] Rahman M M, Islam M R, Akash S, et al. Naphthoquinones and derivatives as potential anticancer agents: An updated review[J]. Chemico-Biological Interactions, 2022,368.

[237] Ordóñez N G. Napsin A expression in lung and kidney neoplasia: A review and update[J]. Advances in Anatomic Pathology, 2012,19(1):66-73.

[238] HooKim K, Kavuri S, Lauer S R, et al. Napsin A expression in small cell carcinoma of the lung: A cytologic study with review of differentials[J]. Journal of the American Society of Cytopathology, 2014,3(2):90-95.

[239] Robinson M K, Alpaugh R K, Borghaei H. Naptumomab estafenatox: A new immunoconjugate[J]. Expert Opinion on Biological Therapy, 2010,10(2):273-279.

[240] Dennis T, Fanous M, Mousa S. Natural products for chemopreventive and adjunctive therapy in oncologic disease[J]. Nutrition and Cancer, 2009,61(5):587-597.

[241] Haughney A, Ferrell B R, Coyle N. Nausea & vomiting in end-stage cancer[J]. American Journal of Nursing, 2004,104(11):40-49.

[242] Zell J A, Chang J C. Neoplastic fever: A neglected paraneoplastic syndrome[J]. Supportive Care in Cancer, 2005,13(11):870-877.

[243] Hardeland R. Neurobiology, pathophysiology, and treatment of melatonin deficiency and dysfunction[J]. The Scientific World Journal, 2012,2012.

[244] Kuriakose S, Umadevi N, Mathew S, et al. Neuroendocrine carcinoma of the cervix presenting as intractable hyponatremic seizures due to paraneoplastic SIADH - A rare case report and brief review of the literature[J]. ecancermedicalscience, 2014,8(1).

[245] Faraut B, Bayon V, Léger D. Neuroendocrine, immune and oxidative stress in shift workers[J]. Sleep Medicine Reviews, 2013,17(6):433-444.

[246] Lee E Q, Arrillaga-Romany I C, Wen P Y. Neurologic complications of cancer drug therapies[J]. CONTINUUM Lifelong Learning in Neurology, 2012,18(2):355-365.

[247] Schiff D, Wen P Y, van den Bent M J. Neurological adverse effects caused by cytotoxic and targeted therapies[J]. Nature Reviews Clinical Oncology, 2009,6(10):596-603.

[248] Khasraw M, Posner J B. Neurological complications of systemic cancer[J]. The Lancet Neurology, 2010,9(12):1214-1227.

[249] Rees J. Neurological oncology[J]. Medicine, 2008,36(11):609-615.

[250] Gotti C, Clementi F. Neuronal nicotinic receptors: From structure to pathology[J]. Progress in Neurobiology, 2004,74(6):363-396.

[251] Milch R A. Neuropathic pain: Implications for the surgeon[J]. Surgical Clinics of North America, 2005,85(2):225-236.

[252] Timotheadou E. New agents targeting angiogenesis in glioblastoma[J]. Chemotherapy Research and Practice, 2011,2011.

[253] Cang S, Ma Y, Liu D. New clinical developments in histone deacetylase inhibitors for epigenetic therapy of cancer[J]. Journal of Hematology and Oncology, 2009,2.

[254] Baker D E. New drugs approved by the FDA - New dosage forms and indications. Agents pending FDA approval: Significant labeling changes related to safety[J]. Hospital Pharmacy, 2005,40(3):264-274.

[255] Parekh S, Weniger M A, Wiestner A. New molecular targets in mantle cell lymphoma[J]. Seminars in Cancer Biology, 2011,21(5):335-346.

[256] Hongthanakorn C, Lok A S F. New Pharmacologic Therapies in Chronic Hepatitis B[J]. Gastroenterology Clinics of North America, 2010,39(3):659-680.

[257] Hancox R J, Jones S, Baggott C, et al. New Zealand COPD Guidelines: Quick Reference Guide[J]. New Zealand Medical Journal, 2021,134(1530):76-110.

[258] Oh T K, Kim H G, Song I A. New, long-term opioid use after lung cancer surgery is associated with reduced 2-year survival: a retrospective population-based cohort study in South Korea[J]. REGIONAL ANESTHESIA AND PAIN MEDICINE, 2022,47(11):678-683.

[259] Oh T K, Kim H G, Song I A. New, long-term opioid use after lung cancer surgery is associated with reduced 2-year survival: A retrospective population-based cohort study in South Korea[J]. Regional Anesthesia and Pain Medicine, 2022,47(11):678-683.

[260] Chan J K C. Newly available antibodies with practical applications in surgical pathology[J]. International Journal of Surgical Pathology, 2013,21(6):553-572.

[261] Cahill K, Stead L F, Lancaster T. Nicotine receptor partial agonists for smoking cessation[J]. Cochrane Database of Systematic Reviews, 2008(3).

[262] Gallagher R, Williscroft D. Nicotine withdrawal as an unusual cause of terminal delirium[J]. Canadian Family Physician, 2022,68(8):591-593.

[263] Yuan X, Zhu C J, Wang M N, et al. Night Shift Work Increases the Risks of Multiple Primary Cancers in Women: A Systematic Review and Meta-analysis of 61 Articles[J]. CANCER EPIDEMIOLOGY BIOMARKERS & PREVENTION, 2018,27(1):25-40.

[264] Nicita-Mauro V, Maltese G, Nicita-Mauro C, et al. Non smoking for successful aging: Therapeutic perspectives[J]. Current Pharmaceutical Design, 2010,16(7):775-782.

[265] Papadopoulos D, Papadoudis A, Kiagia M, et al. Nonpharmacologic Interventions for Improving Sleep Disturbances in Patients With Lung Cancer: A Systematic Review and Meta-analysis[J]. J Pain Symptom Manage, 2018,55(5):1364-1381.

[266] Papadopoulos D, Papadoudis A, Kiagia M, et al. Nonpharmacologic Interventions for Improving Sleep Disturbances in Patients With Lung Cancer: A Systematic Review and Meta-analysis[J]. Journal of Pain and Symptom Management, 2018,55(5):1364-1381.

[267] Goodwin S D, Glenny R W. Nonsteroidal anti-inflammatory drug-associated pulmonary infiltrates with eosinophilia: Review of the literature and Food and Drug Administration Adverse Drug Reaction reports[J]. Archives of Internal Medicine, 1992,152(7):1521-1524.

[268] Zilbermint M F, Dobs A S. Nonsteroidal selective androgen receptor modulator Ostarine™ in cancer cachexia[J]. Future Oncology, 2009,5(8):1211-1220.

[269] Sonpavde G, Hutson T E. Novel antiangiogenic agents in the treatment of refractory renal cell carcinoma[J]. Clinical Genitourinary Cancer, 2008,6(SUPPLEMENT 1):S29-S36.

[270] Robak T. Novel drugs for chronic lymphoid leukemias: Mechanism of action and therapeutic activity[J]. Current Medicinal Chemistry, 2009,16(18):2212-2234.

[271] Tan J, Cang S, Ma Y, et al. Novel histone deacetylase inhibitors in clinical trials as anti-cancer agents[J]. Journal of Hematology and Oncology, 2010,3.

[272] Cheng X J, Wei S G, Zhang H P, et al. Nurse-led interventions on quality of life for patients with cancer A meta-analysis[J]. MEDICINE, 2018,97(34).

[273] Talas M S, Kapucu S, Bagcivan G, et al. Nursing studies on the symptom control of patients who have received chemotherapy for a cancer diagnosis in Turkey in the last 10 years: A systematic review[J]. Asia-Pacific Journal of Clinical Oncology, 2014,10:231.

[274] Schloss J M, Colosimo M, Airey C, et al. Nutraceuticals and chemotherapy induced peripheral neuropathy (CIPN): Asystematic review[J]. Clinical Nutrition, 2013,32(6):888-893.

[275] Marrone O, Bonsignore M R. Obstructive sleep apnea and cancer: a complex relationship[J]. CURRENT OPINION IN PULMONARY MEDICINE, 2020,26(6):657-667.

[276] Martinez-Garcia M A, Gozal D. Obstructive sleep apnea and cancer: what's next?[Z]. 2021: 84, 403-404.

[277] Cheng L J, Guo H, Zhang Z L, et al. Obstructive sleep apnea and incidence of malignant tumors: a meta-analysis[J]. SLEEP MEDICINE, 2021,84:195-204.

[278] Cheong A, Tan B, Teo Y H, et al. Obstructive Sleep Apnea and Lung Cancer A Systematic Review and Meta-Analysis[J]. ANNALS OF THE AMERICAN THORACIC SOCIETY, 2022,19(3):469-475.

[279] Cheong A, Tan B, Teo Y H, et al. OBSTRUCTIVE SLEEP APNEA AND LUNG CANCER: A SYSTEMATIC REVIEW AND META-ANALYSIS OF 4,885,518 PARTICIPANTS[J]. SLEEP MEDICINE, 2022,100:S260.

[280] Chen M X, Chen L D, Zeng A M, et al. Obstructive sleep apnea and the risk of mortality in patients with lung cancer: a meta-analysis[J]. Sleep and Breathing, 2022,26(2):559-566.

[281] Tonolini M, Ferrario S. Occult lung adenocarcinoma mimicking diabetes insipidus due to single pituitary metastasis: Case report and literature review[J]. European Journal of Oncology, 2011,16(1):55-58.

[282] Guidotti T L, Cottle M K W. Occupational health problems among transit workers[J]. Public Health Reviews, 1987,15(1-2):29-44.

[283] Zhao J, Shi X, Castranova V, et al. Occupational toxicology of nickel and nickel compounds[J]. Journal of Environmental Pathology, Toxicology and Oncology, 2009,28(3):177-208.

[284] Hazin R, Abuzetun J Y, Daoud Y J, et al. Ocular complications of cancer therapy: A primer for the ophthalmologist treating cancer patients[J]. Current Opinion in Ophthalmology, 2009,20(4):308-317.

[285] Tolbert P E. Oils and cancer[J]. Cancer Causes and Control, 1997,8(3):386-405.

[286] Sutherland A, Naessens K, Plugge E, et al. Olanzapine for the prevention and treatment of cancer-related nausea and vomiting in adults[J]. Cochrane Database of Systematic Reviews, 2018,2018(9).

[287] Paredes-Ruiz K J, Chavira-Ramos K, Orozco-Morales M, et al. On the Biomedical Properties of Endocannabinoid Degradation and Reuptake Inhibitors: Pre-clinical and Clinical Evidence[J]. Neurotoxicity Research, 2021,39(6):2072-2097.

[288] Rivera P. Onco-Pulmonologist[J]. Journal of Thoracic Oncology, 2018,13(10):S226-S227.

[289] Vargas-Bermúdez A, Cardenal F, Porta-Sales J. Opioids for the management of dyspnea in cancer patients: Evidence of the last 15 years - A systematic review[J]. Journal of Pain and Palliative Care Pharmacotherapy, 2015,29(4):341-352.

[290] Wang Q, Wang Q, Wang S F, et al. Oral Chinese herbal medicine as maintenance treatment after chemotherapy for advanced non-small-cell lung cancer: A systematic review and meta-analysis[J]. Current Oncology, 2017,24(4):e269-e276.

[291] Jeklin A T, Alamgeer M, Stirling R G, et al. P2.08-02 Burning the Candle at Both Ends-Sleep Quality Before and After Chemotherapy in Lung Cancer Patients - A Systematic Review and Meta-Analysis[J]. Journal of Thoracic Oncology, 2022,17(9):S136-S137.

[292] Johnson M J. Pain[J]. Journal of Thoracic Oncology, 2013,8:S33-S34.

[293] Seamark D A, Seamark C J, Halpin D M G. Palliative care in chronic obstructive pulmonary disease: A review for clinicians[J]. Journal of the Royal Society of Medicine, 2007,100(5):225-233.

[294] de Oliveira Zanuso B, de Oliveira Dos Santos A R, Miola V F B, et al. Panax ginseng and aging related disorders: A systematic review[J]. Experimental Gerontology, 2022,161.

[295] Purgato M, Papola D, Gastaldon C, et al. Paroxetine versus other anti-depressive agents for depression[J]. Cochrane Database of Systematic Reviews, 2014,2014(4).

[296] Graupner C, Kimman M L, Mul S, et al. Patient outcomes, patient experiences and process indicators associated with the routine use of patient-reported outcome measures (PROMs) in cancer care: a systematic review[J]. Supportive Care in Cancer, 2021,29(2):573-593.

[297] Hall E T, Singhal S, Dickerson J, et al. Patient-Reported Outcomes for Cancer Patients Receiving Checkpoint Inhibitors: Opportunities for Palliative Care—A Systematic Review[J]. Journal of Pain and Symptom Management, 2019,58(1):137-156.

[298] Haspel J A, Anafi R, Brown M K, et al. Perfect timing: circadian rhythms, sleep, and immunity - an NIH workshop summary[J]. JCI INSIGHT, 2020,5(1).

[299] Aix S P, Crama L, NúñezBenjumea F J, et al. Performance status and activity level of lung cancer patients using wearable devices as passive monitoring: A scoping review[J]. Journal of Clinical Oncology, 2021,39(15 SUPPL).

[300] Feliciano J L, Waldfogel J M, Sharma R, et al. Pharmacologic Interventions for Breathlessness in Patients with Advanced Cancer: A Systematic Review and Meta-analysis[J]. JAMA Network Open, 2021,4(2).

[301] Zhang Z, Bai J, Zeng Y, et al. Pharmacology, toxicity and pharmacokinetics of acetylshikonin: a review[J]. Pharmaceutical Biology, 2020,58(1):950-958.

[302] Robijns J, Nair R G, Lodewijckx J, et al. Photobiomodulation therapy in management of cancer therapy-induced side effects: WALT position paper 2022[J]. Frontiers in Oncology, 2022,12.

[303] Amarasena I U, Walters J A E, Wood-Baker R, et al. Platinum versus non-platinum chemotherapy regimens for small cell lung cancer[J]. Cochrane Database of Systematic Reviews, 2008(4).

[304] Chapman E J, Martino E D, Edwards Z, et al. Practice review: Evidence-based and effective management of fatigue in patients with advanced cancer[J]. Palliative Medicine, 2022,36(1):7-14.

[305] Kaushik M, Mahendru S, Chaudhary S, et al. Prerequisite of a holistic blend of traditional and modern approaches of cancer management[J]. Current Cancer Therapy Reviews, 2019,15(1):56-64.

[306] Ma Y X, He B, Jiang M Y, et al. Prevalence and risk factors of cancer-related fatigue: A systematic review and meta-analysis[J]. INTERNATIONAL JOURNAL OF NURSING STUDIES, 2020,111.

[307] Amiri S, Hosseini S M. Prevalence of current and former smoking in industrial workers worldwide: a systematic review and meta-analysis[J]. JOURNAL OF ADDICTIVE DISEASES, 2021,39(3):288-306.

[308] Al Maqbali M, Al Sinani M, Alsayed A, et al. Prevalence of Sleep Disturbance in Patients With Cancer: A Systematic Review and Meta-Analysis[J]. CLINICAL NURSING RESEARCH, 2022,31(6):1107-1123.

[309] Lemos M, Lourenço A, Ribeiro M. Psychiatric manifestations of paraneoplastic syndromes[J]. European Psychiatry, 2022,65:S661.

[310] Fehri R, Rejiba R, Mahjoub N. Pure bronchioloalveolar carcinoma (BAC): A case report and review of literature[J]. Tunisie Medicale, 2018,96(4):214.

[311] Matsuda A, Yamaoka K, Tango T. Quality of life in advanced non-small cell lung cancer patients receiving palliative chemotherapy: A meta-analysis of randomized controlled trials[J]. Experimental and Therapeutic Medicine, 2012,3(1):134-140.

[312] Mody G, Carlson R, Espey J, et al. Quality of Life in Patients Undergoing Lung Cancer Surgery: A Qualitative Review of the Literature[J]. Quality of Life Research, 2022,31:S131-S132.

[313] Gayatri D, Efremov L, Kantelhardt E J, et al. Quality of life of cancer patients at palliative care units in developing countries: systematic review of the published literature[J]. Quality of Life Research, 2021,30(2):315-343.

[314] Hechtner M, Eichler M, Buhl R, et al. Quality of life of survivors of non-small cell lung cancer: Overview of associated factors and requirements for aftercare[J]. Onkologe, 2018,24(12):1015-1022.

[315] Shuboni-Mulligan D D, Breton G, Smart D, et al. Radiation chronotherapy-clinical impact of treatment time-of-day: a systematic review[J]. JOURNAL OF NEURO-ONCOLOGY, 2019,145(3):415-427.

[316] Pawłowska E, Romanowska A, Jassem J. Radiotherapy for Leptomeningeal Carcinomatosis in Breast Cancer Patients: A Narrative Review[J]. Cancers, 2022,14(16).

[317] Ulbricht C, Abrams T R, Bent S, et al. Reishi mushroom (Ganoderma lucidum): Systematic review by the natural standard research collaboration[J]. Journal of the Society for Integrative Oncology, 2010,8(4):148-159.

[318] Patton R, Paval D R, McDonald J J, et al. Relationship between cytokines and symptoms in people with incurable cancer: A systematic review[J]. CRITICAL REVIEWS IN ONCOLOGY HEMATOLOGY, 2021,159.

[319] Ruffin R, Bardin P G. Respirology year-in-review 2008: clinical science[J]. Respirology, 2009,14(2):159-166.

[320] Featherstone I, Sheldon T, Johnson M, et al. Risk factors for delirium in adult patients receiving specialist palliative care: A systematic review and meta-analysis[J]. Palliative Medicine, 2022,36(2):254-267.

[321] Tomioka K, Saeki K, Obayashi K, et al. Risk for lung cancer in workers exposed to benzidine and/or beta-naphthylamine: A protocol for systematic review and meta-analysis[J]. Systematic Reviews, 2014,3(1).

[322] Tomioka K, Saeki K, Obayashi K, et al. Risk of Lung Cancer in Workers Exposed to Benzidine and/or Beta-Naphthylamine: A Systematic Review and Meta-Analysis[J]. Journal of epidemiology, 2016,26(9):447-458.

[323] Ugalde P. Role of Pneumonectomy in N2 Disease[J]. Journal of Thoracic Oncology, 2018,13(10):S211-S212.

[324] Zhang T, Sun J, Li J, et al. Safety and efficacy profile of mogamulizumab (Poteligeo) in the treatment of cancers: an update evidence from 14 studies[J]. BMC Cancer, 2021,21(1).

[325] Heywood R, McCarthy A L, Skinner T L. Safety and feasibility of exercise interventions in patients with advanced cancer: a systematic review[J]. SUPPORTIVE CARE IN CANCER, 2017,25(10):3031-3050.

[326] Nishijima T F, Shachar S S, Nyrop K A, et al. Safety and tolerability of PD-1/PD-L1 inhibitors compared with chemotherapy in patients with advanced cancer: A meta-analysis[J]. Oncologist, 2017,22(4):470-479.

[327] Hendriks L E L, Schoenmaekers J, Zindler J D, et al. Safety of cranial radiotherapy concurrent with tyrosine kinase inhibitors in non-small cell lung cancer patients: A systematic review[J]. Cancer Treatment Reviews, 2015,41(7):634-645.

[328] Ma W, Xu M, Liu Y, et al. Safety profile of combined therapy inhibiting EFGR and VEGF pathways in patients with advanced non-small-cell lung cancer: A meta-analysis of 15 phase II/III randomized trials[J]. International Journal of Cancer, 2015,137(2):409-419.

[329] Nijakowski K, Surdacki M, Sobieszczańska M. Salivary Melatonin Changes in Oncological Patients: A Systematic Review[J]. Metabolites, 2022,12(5).

[330] Veldhuijzen E, Walraven I, Belderbos J. Selecting a subset based on the patient-reported outcomes version of the common terminology criteria for adverse events for patient-reported symptom monitoring in lung cancer treatment: Mixed methods study[J]. JMIR Cancer, 2021,7(3).

[331] Hammer M J, Ercolano E A, Wright F, et al. Self-management for adult patients with cancer an integrative review: An integrative review[J]. Cancer Nursing, 2015,38(2):E10-E26.

[332] Khan S, Duan P, Yao L, et al. Shiftwork-mediated disruptions of circadian rhythms and sleep homeostasis cause serious health problems[J]. International Journal of Genomics, 2018,2018.

[333] Martin T D, Green M S, Whitehead M T, et al. Six weeks of oral Echinacea purpurea supplementation does not enhance the production of serum erythropoietin or erythropoietic status in recreationally active males with above-average aerobic fitness[J]. 2019,44(7):791-795.

[334] Sateia M J, Lang B J. Sleep and cancer: Recent developments[J]. Current Oncology Reports, 2008,10(4):309-318.

[335] Gozal D, Almendros I, Phipps A I, et al. Sleep Apnoea Adverse Effects on Cancer: True, False, or Too Many Confounders?[J]. INTERNATIONAL JOURNAL OF MOLECULAR SCIENCES, 2020,21(22).

[336] Li X, Huang D, Liu F, et al. Sleep Characteristics and Cancer-Related Outcomes: An Umbrella Review of Systematic Reviews and Meta-Analyses of Observational Studies[J]. Journal of Clinical Medicine, 2022,11(24).

[337] Li J, Cao D, Huang Y, et al. Sleep duration and health outcomes: an umbrella review[J]. Sleep Breath, 2022,26(3):1479-1501.

[338] Kemple M, O'Toole S, O'Toole C. Sleep quality in patients with chronic illness[J]. JOURNAL OF CLINICAL NURSING, 2016,25(21-22):3363-3372.

[339] Ma H, Zhang X, Han J, et al. Sleep-disordered breathing and risk of lung cancer: a meta-analysis longitudinal follow-up studies[J]. Eur J Cancer Prev, 2022,31(3):245-252.

[340] Ma H F, Zhang X F, Han J, et al. Sleep-disordered breathing and risk of lung cancer: a meta-analysis longitudinal follow-up studies[J]. EUROPEAN JOURNAL OF CANCER PREVENTION, 2022,31(3):245-252.

[341] Tobin M J. Sleep-Disordered Breathing, Control of Breathing, Respiratory Muscles, Pulmonary Function Testing in AJRCCM 2003[J]. American Journal of Respiratory and Critical Care Medicine, 2004,169(2):254-264.

[342] Wiest C, Arzt M, Schulz C, et al. Sleep-disordered breathing, hypoxemia and the association with lung cancer[J]. ZEITSCHRIFT FUR PNEUMOLOGIE, 2022,19(4):224-229.

[343] Jayes L, Haslam P L, Gratziou C G, et al. SmokeHaz Systematic Reviews and Meta-analyses of the Effects of Smoking on Respiratory Health[J]. CHEST, 2016,150(1):164-179.

[344] Tønnesen P, Carrozzi L, Fagerström K O, et al. Smoking cessation in patients with respiratory diseases: A high priority, integral component of therapy[J]. European Respiratory Journal, 2007,29(2):390-417.

[345] Simoff M J, Lally B, Slade M G, et al. Symptom management in patients with lung cancer: Diagnosis and management of lung cancer, 3rd ed: American college of chest physicians evidence-based clinical practice guidelines[J]. Chest, 2013,143(5 SUPPL):e455S-e497S.

[346] Otte J L, Carpenter J S, Manchanda S, et al. Systematic review of sleep disorders in cancer patients: can the prevalence of sleep disorders be ascertained?[J]. CANCER MEDICINE, 2015,4(2):183-200.

[347] Liu B, Lee K, Sun C, et al. Systematic review on factors associated with self-perceived burden among cancer patients[J]. Supportive Care in Cancer, 2022,30(10):8417-8428.

[348] Fortner R T, Damms-Machado A, Kaaks R. Systematic review: Tumor-associated antigen autoantibodies and ovarian cancer early detection[J]. GYNECOLOGIC ONCOLOGY, 2017,147(2):465-480.

[349] Yang L, Winters-Stone K, Rana B, et al. Tai Chi for cancer survivors: A systematic review toward consensus-based guidelines[J]. CANCER MEDICINE, 2021,10(21):7447-7456.

[350] Lu A, Xue C, Bian Z, et al. TCM Zheng classification and clinical trials 2014[J]. Evidence-based Complementary and Alternative Medicine, 2015,2015.

[351] Zhang Y, Schmidt-Wolf I G H. Ten-year update of the international registry on cytokine-induced killer cells in cancer immunotherapy[J]. Journal of Cellular Physiology, 2020,235(12):9291-9303.

[352] Zikos E, Coens C, Quinten C, et al. The Added Value of Analyzing Pooled Health-Related Quality of Life Data: A Review of the EORTC PROBE Initiative[J]. Journal of the National Cancer Institute, 2016,108(5).

[353] Stone C R, Haig T R, Fiest K M, et al. The association between sleep duration and cancer-specific mortality: a systematic review and meta-analysis[J]. Cancer Causes Control, 2019,30(5):501-525.

[354] Stone C R, Haig T R, Fiest K M, et al. The association between sleep duration and cancer-specific mortality: a systematic review and meta-analysis[J]. Cancer Causes and Control, 2019.

[355] Brzecka A, Sarul K, Dyla T, et al. The Association of Sleep Disorders, Obesity and Sleep-Related Hypoxia with Cancer[J]. CURRENT GENOMICS, 2020,21(6):444-453.

[356] Liu Q, Zhang Y, Liu M, et al. The benefits and risks of pembrolizumab in combination with chemotherapy as first-line therapy in small-cell lung cancer: a single-arm meta-analysis of noncomparative clinical studies and randomized control trials[J]. World Journal of Surgical Oncology, 2021,19(1).

[357] Gutenbrunner C, Girke M, Dimeo F, et al. The Cancer Fatigue Syndrome - An Overview[J]. PHYSIKALISCHE MEDIZIN REHABILITATIONSMEDIZIN KURORTMEDIZIN, 2010,20(2):86-91.

[358] So W, Law B, Chan D, et al. The Effect of Nonpharmacological Interventions on Managing Symptom Clusters Among Cancer Patients A Systematic Review[J]. CANCER NURSING, 2020,43(6):E304-E327.

[359] Xiao W L, Chow K M, So W, et al. The Effectiveness of Psychoeducational Intervention on Managing Symptom Clusters in Patients With Cancer A Systematic Review of Randomized Controlled Trials[J]. CANCER NURSING, 2016,39(4):279-291.

[360] Reid K F, Bannuru R R, Wang C, et al. The Effects of Tai Chi Mind-Body Approach on the Mechanisms of Gulf War Illness: an Umbrella Review[J]. Integrative Medicine Research, 2019,8(3):167-172.

[361] Zhang J, Xie Y, Kwong J S W, et al. The Efficacy and Safety of Revefenacin for the Treatment of Chronic Obstructive Pulmonary Disease: A Systematic Review[J]. Frontiers in Pharmacology, 2021,12.

[362] Ganbat D, Jugder B E, Ganbat L, et al. The Efficacy of Vitamin K, A Member Of Naphthoquinones in the Treatment of Cancer: A Systematic Review and Meta-Analysis[J]. Current Cancer Drug Targets, 2021,21(6):495-513.

[363] Ganbat D, Jugder B E, Ganbat L, et al. The Efficacy of Vitamin K, A Member Of Naphthoquinones in the Treat-ment of Cancer: A Systematic Review and Meta-Analysis[J]. CURRENT CANCER DRUG TARGETS, 2021,21(6):495-513.

[364] Li L, Li X, Yin J, et al. The high diagnostic accuracy of combined test of thyroid transcription factor 1 and napsin a to distinguish between lung adenocarcinoma and squamous cell carcinoma: A meta-analysis[J]. PLoS ONE, 2014,9(7).

[365] Li L, Li X R, Yin J Y, et al. The High Diagnostic Accuracy of Combined Test of Thyroid Transcription Factor 1 and Napsin A to Distinguish between Lung Adenocarcinoma and Squamous Cell Carcinoma: A Meta-Analysis[J]. PLOS ONE, 2014,9(7).

[366] Garin M C, Burns C M, Kaul S, et al. The human experience with ghrelin administration[J]. Journal of Clinical Endocrinology and Metabolism, 2013,98(5):1826-1837.

[367] Martelli C, Rana R, Alfano F, et al. The impact of gender differences on pulmonary diseases and their clinical implications[J]. MINERVA RESPIRATORY MEDICINE, 2022,61(3):120-137.

[368] Tian X, Yi L J, Liang C, et al. The Impact of Mindfulness-Based Stress Reduction (MBSR) on Psychological Outcomes and Quality of Life in Patients With Lung Cancer: A Meta-Analysis[J]. FRONTIERS IN PSYCHOLOGY, 2022,13.

[369] Marquis P, Caron M, Emery M P, et al. The role of health-related quality of life data in the drug approval processes in the us and Europe: A review of guidance documents and authorizations of medicinal products from 2006 to 2010[J]. Pharmaceutical Medicine, 2011,25(3):147-160.

[370] Römer M, Dörfler J, Huebner J. The use of ketogenic diets in cancer patients: a systematic review[J]. Clinical and Experimental Medicine, 2021,21(4):501-536.

[371] Yang C P, Liu Y C. Therapeutics for Inflammatory-Related Diseases Based on Plasmon-Activated Water: A Review[J]. Int J Mol Sci, 2018,19(6).

[372] Yang C P, Liu Y C. Therapeutics for Inflammatory-Related Diseases Based on Plasmon-Activated Water: A Review[J]. INTERNATIONAL JOURNAL OF MOLECULAR SCIENCES, 2018,19(6).

[373] Lu C L, Li X, Zhou H M, et al. Traditional Chinese Medicine in Cancer Care: An Overview of 5834 Randomized Controlled Trials Published in Chinese[J]. Integrative Cancer Therapies, 2021,20.

[374] Jeklin A T, Kumarahuru R, Amalgeer M, et al. Understanding the effects of chemotherapy on sleep disturbance in lung cancer patients: A systematic review[J]. Asia-Pacific Journal of Clinical Oncology, 2021,17(SUPPL 9):172.

[375] Reddy V, Myers B, Brownstone N, et al. Update on Sleep and Pulmonary Comorbidities in Psoriasis[J]. Current Dermatology Reports, 2020,9(1):30-35.

[376] Rondanelli M, Faliva M A, Perna S, et al. Update on the role of melatonin in the prevention of cancer tumorigenesis and in the management of cancer correlates, such as sleep-wake and mood disturbances: Review and remarks[J]. Aging Clinical and Experimental Research, 2013,25(5):499-510.

[377] Howlett C, Gonzalez R, Yerram P, et al. Use of naloxone for reversal of life-threatening opioid toxicity in cancer-related pain[J]. Journal of Oncology Pharmacy Practice, 2016,22(1):114-120.

[378] Ganbat D, Jugder B E, Ganbat L, et al. Use of the Naphthoquinone YM155 (Sepantronium Bromide) in the Treatment of Cancer: A Systematic Review and Meta-Synthesis[J]. ONCOLOGIE, 2022,24(2):195-225.

[379] Leaviss J, Sullivan W, Ren S, et al. What is the clinical effectiveness and cost-effectiveness of cytisine compared with varenicline for smoking cessation? a systematic review and economic evaluation[J]. Health Technology Assessment, 2014,18(33):1-119.

[380] Banik K, Khatoon E, Harsha C, et al. Wogonin and its analogs for the prevention and treatment of cancer: A systematic review[J]. Phytotherapy Research, 2022,36(5):1854-1883.

[381] Dong B, Lin L, Chen Q, et al. Wrist-ankle acupuncture has a positive effect on cancer pain: a meta-analysis[J]. BMC Complementary Medicine and Therapies, 2021,21(1).

[382] Eastwood P R, Takahashi K, Lee P, et al. Year in review 2010: interstitial lung diseases, acute lung injury, sleep, physiology, imaging, bronchoscopic intervention and lung cancer[J]. Respirology, 2011,16(3):553-563.

[383] Takahashi K, Eves N D, Piper A, et al. Year in review 2011: acute lung injury, interstitial lung diseases, physiology, sleep and lung cancer[J]. Respirology, 2012,17(3):554-562.

[384] Maher T M, Piper A, Song Y, et al. Year in review 2014: Interstitial lung disease, physiology, sleep and ventilation, acute respiratory distress syndrome, cystic fibrosis, bronchiectasis and rare lung disease[J]. Respirology, 2015,20(5):834-845.

**Part Ⅲ：****Case or genetic, animal and cell studies(n=571)**

[1] Ito M, Tanaka J, Kubota K, et al. [Obstructive sleep apnea syndrome in a patient with superior vena cava syndrome caused by lung cancer][J]. Nihon Kokyuki Gakkai Zasshi, 2000,38(6):471-475.

[2] Yamanaka S, Sakamoto A, Tomoyasu H. [Synchronous multiple primary lung and colon cancers][J]. Kyobu geka. The Japanese journal of thoracic surgery, 2013,66(10):882-885.

[3] Yamanaka S, Sakamoto A, Tomoyasu H. [Synchronous multiple primary lung and colon cancers].[J]. Kyobu geka. The Japanese journal of thoracic surgery, 2013,66(10):882-885.

[4] Yoda A, Nakayama S, Abe K, et al. [Two cases of pleomorphic carcinoma with severe systemic inflammation].[J]. Nihon Kokyūki Gakkai zasshi = the journal of the Japanese Respiratory Society, 2009,47(8):751-757.

[5] Arca K N, Smith J H. “ear burn” as a positional manifestation of gastroesophageal reflux disease[J]. Headache, 2019,59:149-150.

[6] Ai M, Li S S, Chen H, et al. 1,25(OH)(2) D(3) attenuates sleep disturbance in mouse models of Lewis lung cancer, in silico and in vivo[J]. J Cell Physiol, 2021,236(11):7473-7490.

[7] Ai M, Li S S, Chen H, et al. 1,25(OH)(2)D-3 attenuates sleep disturbance in mouse models of Lewis lung cancer, in silico and in vivo[J]. JOURNAL OF CELLULAR PHYSIOLOGY, 2021,236(11):7473-7490.

[8] Ai M, Li S S, Chen H, et al. 1,25(OH)2D3 attenuates sleep disturbance in mouse models of Lewis lung cancer, in silico and in vivo[J]. Journal of Cellular Physiology, 2021,236(11):7473-7490.

[9] Schwartzberg L, Wu A, Hartman J, et al. 1135P Adverse event (AE) burden of nivolumab-based immuno-oncology (IO) therapy with/without chemotherapy (chemo) for first-line (1L) advanced non-small cell lung cancer (aNSCLC)[J]. Annals of Oncology, 2022,33:S1069-S1070.

[10] Xiang Z. 125I brachytherapy in the palliation of painful bone metastases from lung cancer after failure or rejection of conventional treatments[J]. Brachytherapy, 2017,16(3):S93.

[11] Quintanal-Villalonga Á, Taniguchi H, Zhan Y A, et al. 1800O Multi-omic characterization of lung tumors implicates AKT and MYC signaling in adenocarcinoma to squamous cell transdifferentiation[J]. Annals of Oncology, 2021,32:S1226.

[12] Quintanal-Villalonga Á, Taniguchi H, Zhan Y A, et al. 1MO Multi-omic characterization of lung tumors identify AKT and EZH2 as potential therapeutic targets in adenocarcinoma-to-squamous transdifferentiation[J]. Annals of Oncology, 2021,32:S1345.

[13] Quintanal-Villalonga Á, Taniguchi H, Hao Y, et al. 2MO XPO1 inhibition strongly sensitizes to first-line and second-line therapy in small cell lung cancer[J]. Annals of Oncology, 2021,32:S1345.

[14] Liu Z, Zhang Z, Zhang W, et al. 2-Substituted-1-(2-morpholinoethyl)-1H-naphtho[2,3-d]imidazole-4,9-diones: Design, synthesis and antiproliferative activity[J]. Bioorganic and Medicinal Chemistry Letters, 2018,28(14):2454-2458.

[15] Huang Y, Wang J, Huang S L, et al. 5-Lipoxygenase Contributes to Benzo[a]pyrene-Induced Cytotoxicity and DNA Damage in Human Bronchial Epithelial Cells[J]. INTERNATIONAL JOURNAL OF TOXICOLOGY.

[16] Celik Y. 6-(1-Oxobutyl)-5,8-dimethoxy-1,4-naphthoquinone inhibits lewis lung cancer by antiangiogenesis and apoptosis[J]. International Journal of Cancer, 2008,122(10):2403.

[17] Hyo J L, Lee H J, Song G Y, et al. 6-(1-Oxobutyl)-5,8-dimethoxy-1,4-naphthoquinone inhibits Lewis lung cancer by antiangiogenesis and apoptosis[J]. International Journal of Cancer, 2007,120(11):2481-2490.

[18] Oteo M, Romero E, Camara J A, et al. 68Ga-DOTA-NAPamide for PET imaging of melanoma metastases[J]. Molecular Imaging and Biology, 2012,14:S244.

[19] Jandova J, Wondrak G T. 705 Bioluminescent identification of a novel EMT-directed experimental therapeutic blocking invasion and metastasis in human malignant melanoma[J]. Journal of Investigative Dermatology, 2020,140(7):S94.

[20] Shertzer H G, Genter M B, Talaska G, et al. 7H-dibenzo[c,g]carbazole metabolism by the mouse and human CYP1 family of enzymes[J]. Carcinogenesis, 2007,28(6):1371-1378.

[21] Yu Q, Ma Y, Feng T. A 41-Year-Old Woman with a Late Cerebral Metastasis 16 Years After an Initial Diagnosis of Cutaneous Melanoma[J]. American Journal of Case Reports, 2022,23(1).

[22] Imanishi J, Yazawa Y, Meguro S, et al. A bone metastasis of non-small cell lung carcinoma with prominent clear cell features[J]. BMJ Case Reports, 2014.

[23] Argel M, Ferro R, Guerra S, et al. A CASE OF ABDOMINAL PAIN THAT LED TO NOT ONE BUT TWO DIAGNOSES[J]. Chest, 2022,161(1):A312.

[24] Park C W, Lee C H, Whang O W, et al. A case of adenocarinoma of the lung associated with multi-oragn infarctions[J]. Tuberculosis and Respiratory Diseases, 1997,44(5):1177-1183.

[25] Hagihara N, Abe T, Wakamiya T, et al. A case of brain metastasis from pulmonary giant cell carcinoma[J]. Kurume Medical Journal, 2010,57(1-2):39-41.

[26] Galliazzo S, Morando F, Sartorato P, et al. A Case of Cancer-Associated Hyponatraemia: Primary Adrenal Insufficiency Secondary to Nivolumab[J]. Endocrine, Metabolic and Immune Disorders - Drug Targets, 2022,22(3):363-366.

[27] Uday Sumathy S, Bector S, Khan Z, et al. A Case of Delayed Diagnosis: SMARCA-4 Undifferentiated Lung Tumor[J]. American Journal of Respiratory and Critical Care Medicine, 2021,203(9).

[28] Sowmya K S. A case of hypercalcemia[J]. CPD Bulletin Clinical Biochemistry, 2000,2(3):115-116.

[29] Quddus A, Smith A, Mchlane M, et al. A case of inferior vena cava mass on echocardiography[J]. Cardiology (Switzerland), 2016,134:148.

[30] Shen S H, Lee S H. A Case of Lung Cancer with Brain Metastasis following Late-Onset Bipolar Disorder[J]. BEHAVIOURAL NEUROLOGY, 2021,2021.

[31] Yasui O, Kato Y, Oomoto T, et al. A case of lung metastasis occurring four years after pancreaticoduodenectomy for pancreatic cancer[J]. Japanese Journal of Cancer and Chemotherapy, 2019,46(10):1581-1585.

[32] Ju M, Aoyama T, Endo K, et al. A Case of Metastatic Small Intestinal Tumor Diagnosed with Intestinal Obstruction[J]. Gan to kagaku ryoho. Cancer & chemotherapy, 2020,47(13):2373-2375.

[33] Fukushima K, Shibuya H, Shimizu I, et al. A case of metastatic thoracic lymph node carcinoma of unknown origin[J]. Japanese Journal of Lung Cancer, 2013,53(1):35-41.

[34] Shi G, Feng F, Hao C, et al. A case of multilevel percutaneous vertebroplasty for vertebral metastases resulting in temporary paraparesis[J]. Journal of International Medical Research, 2019,48(2).

[35] Nakashima Y, Komatsu Y, Taki R, et al. A case of occult lung cancer detected seven years after the appearance of brain metastases[J]. Japanese Journal of Lung Cancer, 2017,57(7):838-842.

[36] Onishi Y, Shinohara Y, Fujiyama H. A case of paraneoplastic limbic encephalitis during the course of depression[J]. Journal of Neuropsychiatry and Clinical Neurosciences, 2011,23(1):E25.

[37] Hye S P, Yun S S, So Y L, et al. A case of persistent hiccup in a patient with non-small cell lung cancer[J]. Tuberculosis and Respiratory Diseases, 2008,64(1):39-43.

[38] Mizoguchi T, Yano H, Suzui N, et al. A case of primary lung carcinoma solitarily metastasizing to the third ventricle[J]. Interdisciplinary Neurosurgery: Advanced Techniques and Case Management, 2021,24.

[39] Tsuchiya N, Miyagi K, Fujita J, et al. A Case of Pulmonary Enteric Adenocarcinoma with Calcification[J]. Japanese Journal of Lung Cancer, 2021,61(7):979-984.

[40] Asai K, Sato K, Kubo Y, et al. A case of rapidly progressed primary pleural synovial sarcoma[J]. Chest, 2015,148(4).

[41] Nakada T, Koshiishi H, Imaizumi K, et al. A case of resectable lung metastasis one year six months after surgery for pancreatic cancer[J]. Japanese Journal of Cancer and Chemotherapy, 2014,41(12):2160-2162.

[42] Asada T, Ayabe T, Chosa E, et al. A case of resection for pulmonary metastasis derived from intrahepatic cholangiocarcinoma[J]. Journal of Hepato-Biliary-Pancreatic Sciences, 2017,24:A294.

[43] Werda I, Feki J, Khemiri S, et al. A case of severe paraneoplastic itch resistant to antihistamines and responding to serotonin reuptake inhibitors[J]. Clinical Case Reports, 2021,9(7).

[44] Salama A, Shata M, Hussein A, et al. A CASE OF STEMI SECONDARY TO CAPECITABINE. HAVE YOU CHECKED THE MEDICATION HISTORY?[J]. Journal of the American College of Cardiology, 2022,79(9):3021.

[45] Nakazawa T, Hirono Y, Koneri K, et al. A case of stomach metastasis of pleomorphic carcinoma of the lung with hypercalcemia[J]. Journal of Japanese Society of Gastroenterology, 2012,109(7):1204-1212.

[46] Eguchi K, Tsujita A, Ono S, et al. A case of syncope induced in the supine position[J]. International Journal of Hypertension, 2011,2011.

[47] Di Paolo A, Ciofi L, Bacca A, et al. A case report of a TDM-guided optimization of mitotane for a safe and effective long-term treatment[J]. Journal of Chemotherapy, 2019,31(2):105-108.

[48] Giscombe L, Keating M, Dominguez M, et al. A Case Report of Discordant Markers of Lung Cancer Tumor Cells: An Unusual Immunophenotype of Uncertain Significance[J]. Journal of Thoracic Oncology, 2018,13(10):S560.

[49] Yang M, Yang Y, Chen J, et al. A case report of primary signet ring cell carcinoma of the lung: Imaging study and literature review[J]. Translational Lung Cancer Research, 2021,10(9):3840-3849.

[50] Hartland M C, Davison K, Nelson M J, et al. A Case Study of Exercise Adherence during Stereotactic Ablative Radiotherapy Treatment in a Previously Active Male with Metastatic Renal Cell Carcinoma[J]. J Sports Sci Med, 2019,18(3):462-470.

[51] Hartland M C, Davison K, Nelson M J, et al. A Case Study of Exercise Adherence during Stereotactic Ablative Radiotherapy Treatment in a Previously Active Male with Metastatic Renal Cell Carcinoma[J]. Journal of sports science & medicine, 2019,18(3):462-470.

[52] Pidugu L, Mbimba J, Ahmad M, et al. A direct interaction between NQO1 and a chemotherapeutic dimeric naphthoquinone[J]. BMC STRUCTURAL BIOLOGY, 2016,16.

[53] Foster M M, Brown J R, Wang L C, et al. A disaccharide precursor of sialyl Lewis X inhibits metastatic potential of tumor cells[J]. CANCER RESEARCH, 2003,63(11):2775-2781.

[54] Brown J R, Fuster M M, Li R, et al. A disaccharide-based inhibitor of glycosylation attenuates metastatic tumor cell dissemination[J]. Clinical Cancer Research, 2006,12(9):2894-2901.

[55] Fernandez L, Sua L F, Garcia L, et al. A fatal case of giant cell pulmonary sarcomatoid carcinoma diagnosed in a pregnant woman: Case report[J]. American Journal of Respiratory and Critical Care Medicine, 2015,191.

[56] Duca M, Lim D W T, Subbiah V, et al. A First-in-Human, Phase I, Multicenter, Open-Label, Dose-Escalation Study of PCA062: An Antibody-Drug Conjugate Targeting P-Cadherin, in Patients With Solid Tumors[J]. Molecular Cancer Therapeutics, 2022,21(4):625-634.

[57] Hankal C W. A glimpse beyond.[J]. The American journal of nursing, 1993,93(11):96.

[58] Sclater K, Natarajan A, Thi L, et al. A heart full of cancer: a case of malignant tamponade[J]. American Journal of the Medical Sciences, 2023,365:S126-S127.

[59] Dai J, Li B, Shi J, et al. A humanized anti-osteopontin antibody inhibits breast cancer growth and metastasis in vivo[J]. Cancer Immunology, Immunotherapy, 2010,59(3):355-366.

[60] Chiu H W, Hung S W, Chiu C F, et al. A Mitochondrion-Targeting Protein (B2) Primes ROS/Nrf2-Mediated Stress Signals, Triggering Apoptosis and Necroptosis in Lung Cancer[J]. BIOMEDICINES, 2023,11(1).

[61] Lee M C, Cai H, Murray C W, et al. A multiplexed in vivo approach to identify driver genes in small cell lung cancer[J]. Cell Reports, 2023,42(1).

[62] Lee M C, Cai H, Murray C W, et al. A MULTIPLEXED IN VIVO APPROACH TO IDENTIFY DRIVER GENES IN SMALL CELL LUNG CANCER[Z]. 2022.

[63] Ma J, Li L, Yue K, et al. A naphthalimide-polyamine conjugate preferentially accumulates in hepatic carcinoma metastases as a lysosome-targeted antimetastatic agent[J]. European Journal of Medicinal Chemistry, 2021,221.

[64] Bergstrom D, Bodyak N, Yurkovetskiy A, et al. A NAPI2B antibody-drug conjugate induces durable complete tumor regressions in patient-derived xenograft models of NSCLC[J]. Journal of Thoracic Oncology, 2017,12(1):S396-S397.

[65] Shukla A, Thakur R, Pandeti S, et al. A novel alkannin derivative suppresses breast cancer proliferation and angiogenesis through modulation of STAT3 signaling[J]. Molecular Cancer Therapeutics, 2018,17(1).

[66] Gavrancic T, Park Y H A. A novel approach using sorafenib in alpha fetoprotein-producing hepatoid adenocarcinoma of the lung[J]. JNCCN Journal of the National Comprehensive Cancer Network, 2015,13(4):387-391.

[67] Lei L, Chen Y, Yang S, et al. A novel aza-naphthoquinone inhibits tumor growth by inducing apoptosis[J]. Journal of Chinese Pharmaceutical Sciences, 2018,27(9):600-607.

[68] Sato A, McNulty L, Cox K, et al. A novel class of in vivo active anticancer agents: Achiral seco-amino- and seco-hydroxycyclopropylbenz[e]indolone (seco-CBI) analogues of the duocarmycins and CC-1065[J]. Journal of Medicinal Chemistry, 2005,48(11):3903-3918.

[69] Song Y H, Shin E, Wang H, et al. A novel in situ hydrophobic ion paring (HIP) formulation strategy for clinical product selection of a nanoparticle drug delivery system[J]. Journal of Controlled Release, 2016,229:106-119.

[70] Wang T, Du G, Niu M, et al. A novel intergenic region (chr2: 30,193,816)-ALK fusion shows sensitivity to Alectinib in lung adenocarcinoma[J]. BMC Pulmonary Medicine, 2023,23(1).

[71] Zhang G, An Y, Lu X, et al. A novel naphthalimide compound restores p53 function in non-small cell lung cancer by reorganizing the Bak·Bcl-xl complex and triggering transcriptional regulation[J]. Journal of Biological Chemistry, 2016,291(8):4211-4225.

[72] Ma Y L, Lin S W, Fang H C, et al. A novel poly-naphthol compound ST104P suppresses angiogenesis by attenuating matrix metalloproteinase-2 expression in endothelial cells[J]. International Journal of Molecular Sciences, 2014,15(9):16611-16627.

[73] Lee J W, Park H S, Park S A, et al. A Novel Small-Molecule Inhibitor Targeting CREB-CBP Complex Possesses Anti-Cancer Effects along with Cell Cycle Regulation, Autophagy Suppression and Endoplasmic Reticulum Stress[J]. PLOS ONE, 2015,10(4).

[74] Muramatsu N. A patient with a lung cancer exhibited extremely favorable effect of fever by naproxen: a case report[J]. Shigaku. Odontology; journal of Nippon Dental College, 1988,76(4):816-820.

[75] Haas N S, Shih R, Gochfeld M. A patient with postoperative mercury contamination of the peritoneum[J]. J Toxicol Clin Toxicol, 2003,41(2):175-180.

[76] Kanthala S P, Liu Y Y, Singh S, et al. A peptidomimetic with a chiral switch is an inhibitor of epidermal growth factor receptor heterodimerization[J]. Oncotarget, 2017,8(43):74244-74262.

[77] Xin X, Zhu B, Shen J, et al. A primary spinal extradural atypical teratoid/rhabdoid tumor of the cervical spine with bony involvement[J]. Journal of Child Neurology, 2014,29(5):670-673.

[78] Tang Y, Yu F, Zhang G, et al. A Purified Serine Protease from Nereis virens and Its Impaction of Apoptosis on Human Lung Cancer Cells[J]. Molecules (Basel, Switzerland), 2017,22(7).

[79] Kyu-K A, Chen K, Ayers M, et al. A rare benign pulmonary neoplasm: A case report of benign pulmonary papillary adenoma[J]. Chest, 2017,152(4):A713.

[80] Rudrappa M, Kokatnur L. A rare case of bladder metastasis from primary lung adenocarcinoma and definitive diagnosis by immunohistochemistry[J]. Chest, 2017,152(4):A649.

[81] Torio J D. A RARE CASE OF ENDOBRONCHIAL METASTASIS FROM ENDOMETRIAL CARCINOMA[J]. Chest, 2019,156(4):A802.

[82] Bouanzoul M, Ketfi A. A Rare Case of Giant Cell Pleomorphic Carcinoma of the Lung Presenting as a Voluminous Cavitary Lesion[J]. American Journal of Respiratory and Critical Care Medicine, 2022,205(1).

[83] Young M, Beg M, Arshad H. A rare case of lymphangitic carcinomatosis from adenocarcinoma of the lung presenting as ARDS[J]. American Journal of Respiratory and Critical Care Medicine, 2017,195.

[84] Ahmed A, Donna P D, Nasir U M, et al. A rare case of poorly differentiated lung carcinoma with duodenal metastasis[J]. American Journal of Gastroenterology, 2019,114:S1464-S1466.

[85] Malik S A, Keshava K. A rare case of primary hepatoid adenocarcinoma of the lung in a female patient[J]. American Journal of Respiratory and Critical Care Medicine, 2019,199(9).

[86] Shen C, Wang X, Che G. A rare case of primary peripheral epithelial myoepithelial carcinoma of lung Case report and literature review[J]. Medicine (United States), 2016,95(35).

[87] Yunina D, Fazio R M, Zahir I, et al. A rare case of pulmonary adenocarcinoma metastasizing to the common bile duct[J]. American Journal of Gastroenterology, 2020,115(SUPPL):S812.

[88] Pozarskis A. A rare cause of the secondary hypogonadism[J]. Journal of Sexual Medicine, 2016,13(5):S203.

[89] Rana R, Zubairi A, Ahmed A. A RARE TUMOR OF PLEURA IN A GENTLEMAN FROM AFGHANISTAN[J]. Chest, 2021,160(4):A1655.

[90] Mearini L, Colella R, Zucchi A, et al. A review of penile metastasis[J]. Oncology Reviews, 2012,6(1):80-87.

[91] Dorr C, Weg M, Been R, et al. A Sleeping Beauty forward genetic screen identifies novel cancer drivers that cooperate with Pten in lung cancer[J]. Cancer Research, 2013,73(19):135D.

[92] Hashimoto Y. A study on the mechanism of activation of peritoneal exudate macrophages from MRL/MpJ-lpr/lpr mice[J]. [Hokkaido igaku zasshi] The Hokkaido journal of medical science, 1988,63(5):781-790.

[93] Murakami S, Asada T, Kubota T, et al. A Successful Case of Switching Treatment from Ketamine to Methadone for Complex Neuropathic Pain[J]. Journal of Palliative Medicine, 2022,25(4):686-689.

[94] Williams J, Eapen G. A tale of two primary malignancies: Synchronous breast and lung cancers in two women[J]. Chest, 2017,152(4):A686.

[95] Gürlevik E, Fleischmann-Mundt B, Armbrecht N, et al. A transposon-based primary tumor resection model of intrahepatic cholangiocarcinoma (ICC) with extrahepatic distant metastases[J]. Journal of Hepatology, 2013,58:S39.

[96] Vashi P G, Gupta D, Dahlk S. A unique case of a nonfunctional metastatic pancreatic neuroendocrine tumor transforming into an insulin-secreting tumor with an unusual clinical course[J]. Pancreas, 2011,40(5):781-784.

[97] Li Y, Linnoila I. Achaete-scute homolog 1 (Ascl1) lineage in the lung gives rise to multiple cell types[J]. Cancer Research, 2012,72(8).

[98] Jensen-Taubman S, Wang X Y, Linnoila R I. Achaete-scute homologue-1 tapers neuroendocrine cell differentiation in lungs after exposure to naphthalene[J]. Toxicological Sciences, 2010,117(1):238-248.

[99] Albalbissi A A, Treece J, Alazzeh A, et al. Achromobacter denitrificans-a rare cause of pneumonia[J]. American Journal of Respiratory and Critical Care Medicine, 2018,197(MeetingAbstracts).

[100] Poulsen T T, Naizhen X, Poulsen H S, et al. Acute damage by naphthalene triggers expression of the neuroendocrine marker PGP9.5 in airway epithelial cells[J]. Toxicology Letters, 2008,181(2):67-74.

[101] van Londen M, Roosma E, Vogels S, et al. Acute kidney injury and long-term renal effects of alectinib in anaplastic lymphoma kinase-positive non-small cell lung carcinoma: a case report[J]. Journal of Medical Case Reports, 2022,16(1).

[102] Neumann M, Livak V, Paul H W, et al. Acute psychosis after administration of bupropion hydrochloride (Zyban™)[J]. Pharmacopsychiatry, 2002,35(6):247-248.

[103] Abhijit D, Niharika P, Lucy P, et al. Adenocarcinoma of lung with brain metastasis and metastasis to thyroid: A case report[J]. Journal of Cancer Research and Therapeutics, 2014,10:S37.

[104] Shastri K, Chen T, Peris-Celda M, et al. Adenocarcinoma of the lung presenting as isolated hypoglossal nerve palsy[J]. Journal of Neurological Surgery Part B: Skull Base, 2017,78.

[105] Almendros I, Gileles-Hillel A, Khalyfa A, et al. Adipose tissue macrophage polarization by intermittent hypoxia in a mouse model of OSA: effect of tumor microenvironment[J]. Cancer Lett, 2015,361(2):233-239.

[106] Kanti M M, Striessnig-Bina I, Wieser B I, et al. Adipose triglyceride lipase-mediated lipid catabolism is essential for bronchiolar regeneration[J]. JCI Insight, 2022,7(9).

[107] García-Sanmartín J, Larrayoz I M, Martínez A. Adrenomedullin regulates club cell recovery following lung epithelial injury[J]. Histology and histopathology, 2016,31(6):663-673.

[108] Arash M T, Park Y C, Hwang S K, et al. Aerosol delivery of kinase-deficient Akt1 attenuates Clara cell injury induced by naphthalene in the lungs of dual luciferase mice[J]. Journal of Veterinary Science, 2011,12(4):309-317.

[109] Torres M, Campillo N, Nonaka P N, et al. Aging Reduces Intermittent Hypoxia-induced Lung Carcinoma Growth in a Mouse Model of Sleep Apnea[J]. Am J Respir Crit Care Med, 2018,198(9):1234-1236.

[110] Flores A, Cabañas M, Elizondo G. AHR inactivation by alpha-naphthoflavone and resveratrol inhibits cell proliferation by increasing P53 and apoptosis levels in hela cells[J]. Drug Metabolism Reviews, 2014,45:232.

[111] Inoue T, Takahashi M, Hosoda M, et al. Akathisia causing secondary severe depression in a cancer patient[J]. Primary Care Companion to the Journal of Clinical Psychiatry, 2010,12(4):e1.

[112] Ignatius Ou S H, Sommers K R, Azada M C, et al. Alectinib induces a durable (>15 months) complete response in an ALK-positive non-small cell lung cancer patient who progressed on crizotinibwith diffuse leptomeningeal carcinomatosis[J]. 2015,20(2):224-226.

[113] Tan Y, Lu X, Li Y, et al. ALK-positive pulmonary adenocarcinoma with signet ring features (PASRF) and polygonal cell morphology simultaneously co-expressing TTF-1/p63/P40: A case report[J]. Translational Cancer Research, 2021,10(8):3864-3869.

[114] Forkert P G, Lord J A, Parkinson A. Alterations in expression of CYP1A1 and NADPH-cytochrome P450 reductase during lung tumor development in SWR/J mice[J]. Carcinogenesis, 1996,17(1):127-132.

[115] Blakley B R. Alterations in urethan-induced adenoma formation in mice exposed to selenium and arsenic[J]. Drug Nutr Interact, 1987,5(2):97-102.

[116] Blakley B R. Alterations in urethan-induced adenoma formation in mice exposed to selenium and nickel[J]. J Appl Toxicol, 1987,7(6):387-390.

[117] Blakley B R. Alterations in urethan-induced adenoma formation in mice exposed to selenium and nickel[J]. Journal of Applied Toxicology, 1987,7(6):387-390.

[118] Kaushal-Deep S, Raswan U, Kirmani A, et al. Alveolar soft part sarcoma metastasizing to the brain: A rare entity revisited with review of recent literature[J]. Journal of Pediatric Neurosciences, 2019,14(3):158-161.

[119] Duan X, Zhao X, Wang S. An ALK-positive lung adenocarcinoma with gastric and skin metastasis: a case report and literature review[J]. Annals of palliative medicine, 2021,10(5):5797-5807.

[120] Yorizumi K, Nakamura H, Sakaida H, et al. An autopsy case of malignant melanoma, misdiagnosed as pulmonary cancer: Effect of naproxen on tumor fever on malignant melanoma[J]. IRYO - Japanese Journal of National Medical Services, 1988,42(4):291-357.

[121] Patel N P, Gooneratne N. An elderly man with a lung mass and neurological symptoms[J]. Journal of Respiratory Diseases, 2007,28(12):558-564.

[122] Liu R, He S L, Hirasaki Y, et al. An elderly patient with advanced lung cancer achieved long-term survival using Chinese medicine: An alternative treatment strategy for cancer patients aged 80 or older without a tissue confirmed diagnosis[J]. Chinese Journal of Integrative Medicine, 2016,22(7):545-548.

[123] Girola N, Matsuo A L, Figueiredo C R, et al. An immunoglobulin VH CDR-3-derived peptide attenuates Hsp90 activity, binds to an adhesion GPCR and promotes hyperadherence, motility arrest and anti-melanoma metastatic activity[J]. European Journal of Cancer, 2015,51:S666.

[124] Mayne K J, Lewis E, Vickers L. An incidental finding of testicular seminoma in the context of acute pulmonary embolism: a case report[J]. Journal of Medical Case Reports, 2021,15(1).

[125] Mathews T, Gigliotti B. An uncommon cause of thyrotoxicosis after in-vitro fertilization[J]. Thyroid, 2021,31(SUPPL 1):A54.

[126] Amaechi E L, Mubarak Y, Murthy S. An Unusual Case of Metastatic Adenocarcinoma of Lung Origin with No Identifiable Lung Mass[J]. American Journal of Respiratory and Critical Care Medicine, 2022,205(1).

[127] Udaykumar A, Archana B, Vindhya P, et al. An unusual presentation of Ca lung[J]. Lung India, 2022,39(SUPPL 1):S203-S204.

[128] Firsova D, Levashov I, Reshetnikova D, et al. Analysis of the sodium-dependent phosphate transporter NaPi2b expression in human tumor cell lines[J]. European Journal of Clinical Investigation, 2022,52:114-115.

[129] Daroszewski J, Paczkowska K, Jawiarczyk-Przybyłowska A, et al. Anaplastic thyroid carcinoma with rapid thyrotoxicosis - A case report and the literature review[J]. Endokrynologia Polska, 2018,69(1):28-31.

[130] Cho J W, Jeong M A, Choi J H, et al. Anesthetic consideration for patients with severe tracheal obstruction caused by thyroid cancer - A report of 2 cases[J]. Korean Journal of Anesthesiology, 2010,58(4):396-400.

[131] Ward J, Standage C. Angina pain precipitated by a continuous subcutaneous infusion of ketamine [4][J]. Journal of Pain and Symptom Management, 2003,25(1):6-7.

[132] Nath A G, Rema P, Suchetha S, et al. Angiosarcoma Ovary and Kasabach–Merritt Syndrome in a Pre-pubertal Girl: An Exceedingly Rare Case Report[J]. Indian Journal of Gynecologic Oncology, 2020,18(3).

[133] Kim A, Im M, Ma J Y. Anisi stellati fructus extract attenuates the in vitro and in vivo metastatic and angiogenic potential of malignant cancer cells by downregulating proteolytic activity and pro-angiogenic factors[J]. International Journal of Oncology, 2014,45(5):1937-1948.

[134] Jesuthasan A, McColgan P, Sharma R, et al. Anti-amphiphysin associated paraneoplastic diencephalitis secondary to a thymic neuroendocrine tumour[J]. Neurological Sciences, 2023,44(2):745-748.

[135] Li M, Zha G, Chen R, et al. Anticancer effects of myricetin derivatives in non-small cell lung cancer in vitro and in vivo[J]. Pharmacology Research and Perspectives, 2022,10(1).

[136] Zhang Y, Talalay P. Anticarcinogenic activities of organic isothiocyanates: Chemistry and mechanisms[J]. Cancer Research, 1994,54(7 SUPPL.):1976s-1981s.

[137] Park M S, Bae J H, Jeong H B, et al. Anti-Ma2 antibody encephalitis manifesting as cognitive impairment and psychosis[J]. Journal of Neuropsychiatry and Clinical Neurosciences, 2015,27(3):e221-e222.

[138] Yamamoto T, Tsuji S. Anti-Ma2-associated encephalitis and paraneoplastic limbic encephalitis[J]. Brain and Nerve, 2010,62(8):838-851.

[139] Byun W S, Shin Y, Lee S K, et al. Antitumor activity of marin natural product derived Psammaplin A analogs in human lung cancer cells[J]. Cancer Research, 2017,77(13).

[140] Shin Y, Lee S K, Park H G, et al. Antitumor activity of marin natural product derived Psammaplin A analogs in human lung cancer cells Woong sub Byun[J]. Cancer Research, 2017,77(13).

[141] Li B, Yuan Z, Jiang J, et al. Anti-tumor activity of Shikonin against afatinib resistant non-small cell lung cancer via negative regulation of PI3K/Akt signaling pathway[J]. Bioscience Reports, 2018,38(6).

[142] Sun L, McPhail A T, Hamel E, et al. Antitumor agents. 139. Synthesis and biological evaluation of thiocolchicine analogs 5,6-dihydro-6(S)-(acyloxy)- and 5,6-dihydro-6(S)- [(aroyloxy)methyl]-1,2,3-trimethoxy-9-(methylthio)-8H- cyclohepta[a]naphthalen-8-ones as novel cytotoxic and antimitotic agents[J]. Journal of Medicinal Chemistry, 1993,36(5):544-551.

[143] Kitagawa R R, Vilegas W, Carlos I Z, et al. Antitumor and immunomodulatory effects of the naphthoquinone 5-methoxy-3,4-dehydroxanthomegnin[J]. Revista Brasileira de Farmacognosia, 2011,21(6):1084-1088.

[144] Zhang X B, Yang Y Y, Zeng Y, et al. Anti-tumor effect of endostatin in a sleep-apnea mouse model with tumor[J]. Clin Transl Oncol, 2019,21(5):572-581.

[145] Esteves-Souza A, Araújo Lúcio K, Da Cunha A S, et al. Antitumoral activity of new polyamine-naphthoquinone conjugates[J]. Oncology Reports, 2008,20(1):225-231.

[146] Jones W P, Lobo-Echeverri T, Mi Q, et al. Antitumour activity of 3-chlorodeoxylapachol, a naphthoquinone from Avicennia germinans collected from an experimental plot in southern Florida[J]. Journal of Pharmacy and Pharmacology, 2005,57(9):1101-1108.

[147] Ifdil I, Fadli R P, Rangka I B, et al. Anxiety, Depression, Psychological Symptoms, Negative Effects, and Other Symptoms of Nicotine Withdrawal[J]. Addictive Disorders and their Treatment, 2021,20(4):591-592.

[148] Liu Y, Chen X, Gao X, et al. Apatinib-induced hyperammonemic encephalopathy[J]. Journal of Oncology Pharmacy Practice, 2020,26(2):465-470.

[149] Royer T. Are softer catheters always best?[J]. JAVA - Journal of the Association for Vascular Access, 2005,10(1):14-15.

[150] Walker W H, Kvadas R M, May L E, et al. Artificial Light at Night Reduces Anxiety-like Behavior in Female Mice with Exacerbated Mammary Tumor Growth[J]. CANCERS, 2021,13(19).

[151] Codipietro L, Maino P. Aseptic arachnoiditis in a patient treated with intrathecal morphine infusion: Symptom resolution on switch to ziconotide[J]. Neuromodulation, 2015,18(3):217-220.

[152] Wei P J, Tsai M J, Tsai Y H, et al. Association between obstructive sleep apnea (OSA) and cancer incidence - A nationwide population-based study[J]. EUROPEAN RESPIRATORY JOURNAL, 2013,42.

[153] Martinez-Garcia M A, Campos-Rodriguez F, Duran-Cantolla J, et al. Association between sleep apnoea and cancer mortality. Longitudinal muticenter study in 5,467 patients from the Spanish cohort[J]. EUROPEAN RESPIRATORY JOURNAL, 2012,40.

[154] Wang J, Tang H, Duan Y, et al. Association between Sleep Traits and Lung Cancer: A Mendelian Randomization Study[J]. J Immunol Res, 2021,2021:1893882.

[155] Di Lucca G, Rossini C, Morena R, et al. Association between the development of autoimmune hypothyroidism and objective response to nivolumab: report of two cases[J]. Annals of Oncology, 2017,28:i99.

[156] Wada S, Iwamoto K, Ozaki N. Atezolizumab, an immune checkpoint inhibitor, caused precedent depressive symptoms related to limbic encephalitis[J]. Psychiatry and Clinical Neurosciences, 2022,76(4):125-126.

[157] Yamaguchi Y, Nagasawa H, Katagiri Y, et al. Atezolizumab-associated encephalitis in metastatic lung adenocarcinoma: a case report[J]. Journal of Medical Case Reports, 2020,14(1).

[158] Shinjo T, Okada M. Atropine eyedrops for death rattle in a terminal cancer patient[J]. Journal of Palliative Medicine, 2013,16(2):212-213.

[159] Monjaras-Romo A, Villarreal E G, Diaz-Arizpe O, et al. Atypical Case of Early-Onset Shapiro Syndrome: Diagnostic Approach and Therapeutic Challenges[J]. Journal of Pediatric Neurology, 2022,20(3):227-230.

[160] Brunet De Courssou J B, Castilla-Lievre M A, Maillot J, et al. Autoimmune cerebellar hypermetabolism: Report of three cases and literature overview[J]. Revue Neurologique, 2022,178(4):337-346.

[161] Brunet De Courssou J B, Castilla-Lievre M A, Maillot J, et al. Autoimmune vermian hypermetabolism: Regarding three cases[J]. European Journal of Neurology, 2020,27:718.

[162] Guidi A, Violati M, Blasi M, et al. Autoimmune-related encephalitis during treatment with nivolumab for advanced head and neck cancer: a case report[J]. Tumori, 2020,106(6):P23-P28.

[163] Chaudhary U, Desai P A, Takahashi N, et al. Automated detection and segmentation of small cell lung cancer liver metastases on CT[J]. Journal of Clinical Oncology, 2022,40(16).

[164] Hayward E, Healy D, Wimbush S. Awareness, dreaming or steroid-induced psychosis? [19][J]. Anaesthesia, 2006,61(11):1127-1128.

[165] Jay Boniface J, Baichwal V R, Cimbora D M, et al. Basal NAD levels and Nampt expression correlate with in vitro and in vivo sensitivity of tumor cell lines to the Nampt inhibitor MPC-9528[J]. Cancer Research, 2011,71(8).

[166] Gerber D. Basic Science[J]. Journal of Thoracic Oncology, 2018,13(10):S262-S263.

[167] Perrin C, Fabre C, Raoul J L, et al. Behavioral disorders secondary to profound hypomagnesemia in a patient given cetuximab for metastatic colorectal cancer hypomagnesemia due to cetuximab treatment [3][J]. Acta Oncologica, 2006,45(8):1135-1136.

[168] Sen S, Aydin O N, Aydin K. Beneficial effect of low-dose ketamine addition to epidural administration of morphine-bupivacaine mixture for cancer pain in two cases[J]. Pain Medicine, 2006,7(2):166-169.

[169] Cheu J, Talaska G, Miller M, et al. Benzo[a]pyrene coated ferric oxide and aluminum oxide particles: Uptake, metabolism and DNA binding in hamster pulmonary alveolar macrophages and tracheal epithelial cells in vitro[J]. Carcinogenesis, 1997,18(1):167-175.

[170] Gupte R, Patil R, Liu J, et al. Benzyl and naphthalene methylphosphonic acid inhibitors of autotaxin with anti-invasive and anti-metastatic activity[J]. ChemMedChem, 2011,6(5):922-935.

[171] Zhang W Q, Meyfeldt J, Wang H B, et al. beta-Catenin mutations as determinants of hepatoblastoma phenotypes in mice[J]. JOURNAL OF BIOLOGICAL CHEMISTRY, 2019,294(46):17524-17542.

[172] Wong Y P, Tan G C, Aziz S, et al. Beta-human chorionic gonadotropin-secreting lung adenocarcinoma[J]. Malaysian Journal of Medical Sciences, 2015,22(4):76-80.

[173] Giza D E, Iliescu C, Kalhor N, et al. Bilateral multiple sclerosing hemangioma: A clinical and imagistic diagnostic challenge[J]. American Journal of Respiratory and Critical Care Medicine, 2017,195.

[174] Aslam E, Imran M, Faridi N M. Bilateral parietal extradural metastatic Ewing's sarcoma simulating acute epidural hematoma[J]. Journal of the College of Physicians and Surgeons Pakistan, 2006,16(8):543-544.

[175] Falkson S R, Zhang K, Bhambhvani H P, et al. Biliary cancer brain metastases: a multi-institution case series with case reports[J]. Journal of Gastrointestinal Oncology, 2022,13(2):822-832.

[176] de Alvarez G B. Bio energy man produced technique to control the EMF of the mitochondria in order to re establish cell’s normal functions[J]. Current Chemical Biology, 2016,10(1):9-17.

[177] Qanash S, Myers R. Bizarre symptoms due to an exceptionally rare endotracheal tumor[J]. Chest, 2015,148(4).

[178] Xie S Q, Li Q, Zhang Y H, et al. BND-12, a novel nonhaematotoxic naphthalimide derivative, inhibits tumour growth and metastasis of hepatocellular carcinoma[J]. Journal of Pharmacy and Pharmacology, 2012,64(10):1483-1490.

[179] Wang J, Li Q, Cheng X, et al. Bone marrow-derived myofibroblasts promote gastric cancer metastasis by activating tgf-β1 and il-6/stat3 signalling loop[J]. OncoTargets and Therapy, 2020,13:10567-10580.

[180] Durán Botía F, Fernández-Aceñero M J, Ruiz Adelantado I, et al. Bone metastasis of papillary thyroid carcinoma simulating a pulmonary origin. Unusual immunohistochemistry leading to misdiagnosis[J]. Revista Espanola de Patologia, 2020,53(4):264-267.

[181] Katsumata R, Monobe Y, Akagi A, et al. Brain and Adrenal Metastasis From Unknown Primary Tumor: A Case Report[J]. CUREUS JOURNAL OF MEDICAL SCIENCE, 2022,14(6).

[182] Jicheng D, Haiyan Y, Kai L, et al. Brain metastases from hepatocellular carcinoma after hepatectomy[J]. Journal of Medical Colleges of PLA, 2010,25(6):368-372.

[183] Alvarez Bravo G, Foronda Bengoa J, Bravo J J, et al. Brainstem encephalitis secondary to antibodies against SOX1 mimicking Bickerstaff's encephalitis[J]. European Journal of Neurology, 2019,26:499-500.

[184] Kostoglou A, Vlastos D, Bakalis A, et al. Breast cancer-associated opsoclonus-myoclonus syndrome: a case report[J]. World Journal of Surgical Oncology, 2021,19(1).

[185] Wang B, Jiang Y, Li S Y, et al. Breast metastases from primary lung cancer: A retrospective case series on clinical, ultrasonographic, and immunohistochemical features[J]. Translational Lung Cancer Research, 2021,10(7):3226-3235.

[186] Walsh E, Montgomery R. Breathless: A case of central neurogenic hyperventilation[J]. Journal of General Internal Medicine, 2017,32(2):S460.

[187] Shrestha P, George M K, Baidya S, et al. Bullous pemphigoid associated with squamous cell lung carcinoma showing remarkable response to carboplatin-based chemotherapy: a case report[J]. JOURNAL OF MEDICAL CASE REPORTS, 2022,16(1).

[188] Divya K P, Cherian A, Prabhakaran P K, et al. Calcific Miliary Brain Metastasis in Adenocarcinoma Lung Treated with Gefitinib[J]. Neurology India, 2022,70(3):1197-1199.

[189] Weiser K C, Justice M J. Cancer biology: Sleeping Beauty awakens[J]. Nature, 2005,436(7048):184-186.

[190] Park J, Qiu J, Nakasone E S, et al. Cancer cell-secreted CXCL1 chemokine acts on neutrophils to support metastasis[J]. Cancer Research, 2013,73(3).

[191] Ganguly A, Michael M, Gorschin S, et al. Cancer Pain and Opioid Use Disorder[J]. ONCOLOGY (United States), 2022(3609):535-541.

[192] Sousa J P, Neves C, Jesus E, et al. Cancer-associated thrombotic diathesis: One of your worst nightmares[J]. European Heart Journal Cardiovascular Imaging, 2020,21:i533.

[193] Seah G L, Yu J H, Koo B I, et al. Cancer-targeted reactive oxygen species-degradable polymer nanoparticles for near infrared light-induced drug release[J]. Journal of materials chemistry. B, 2018,6(46):7737-7749.

[194] Kanisawa M, Katoh H, Aiso K. Carcinogenicity of potassium 1 methyl 7[2 (5 nitro 2 furyl) vinyl] 4 oxo 1,4 dihydro 1,8 naphthyridine 3 carboxylate in ICR mice[J]. Gann, The Japanese Journal of Cancer Research, 1974,65(1):1-11.

[195] Padappayil R, Tiperneni R, Rajamohan A, et al. Carcinoma of Unknown Primary in the Lung : A Tumor with an Identity Crisis![J]. American Journal of Respiratory and Critical Care Medicine, 2022,205(1).

[196] Langer P, Perez-Cruz P E, Escarate C C, et al. Caregivers' accuracy in reporting patients' symptom: A preliminary report[J]. Journal of Clinical Oncology, 2019,34(26):64.

[197] Minematsu T, Iwai M, Sugimoto K, et al. Carrier-mediated uptake of 1 -(2-methoxyethyl)-2-methyl-4,9-dioxo-3- (pyrazin-2-ylmethyl)-4,9-dihydro-1H-naphtho[2,3-d]imidazolium Bromide (YM155 monobromide), a novel small-molecule survivin suppressant, into human solid tumor and lymphoma cells[J]. Drug Metabolism and Disposition, 2009,37(3):619-628.

[198] Neilan T G, Price M C, Sanborn D Y, et al. Case 33-2018: A 57-year-old man with confusion, fever, malaise, and weight loss[J]. New England Journal of Medicine, 2018,379(17):1658-1669.

[199] Sydorchuk L, Sydorchuk A, Sydorchuk I, et al. Case of insulin allergy in patient with diabetes mellitus, tuberculosis and lung cancer[J]. Allergy: European Journal of Allergy and Clinical Immunology, 2010,65:617.

[200] Tamai S, Kinoshita M, Sabit H, et al. Case of metastatic glioblastoma with primitive neuronal component to the lung[J]. Neuropathology, 2019,39(3):218-223.

[201] Cuoco J A, Kortz M W, McCray E, et al. Case Report: Metastatic Bronchopulmonary Carcinoid Tumor to the Pineal Region[J]. Frontiers in Endocrinology, 2021,12.

[202] Li Q, Zhang X, Feng J, et al. Case Report: Next-Generation Sequencing Reveals Tumor Origin in a Female Patient With Brain Metastases[J]. Frontiers in Oncology, 2021,11.

[203] Deldar R, Chen G L, Rizvi I. Case report: Primary lung cancer with solitary metastasis to rectosigmoid colon[J]. Diseases of the Colon and Rectum, 2019,62(6):e159.

[204] Kang H M, Seth P, Ponampalam R, et al. Case report: Unusual radiation risks in the emergency department − 125I beads[J]. Hong Kong Journal of Emergency Medicine, 2016,23(5):279-284.

[205] Aguiar-Bujanda D, Aguiar-Morales J, Bohn-Sarmiento U. Central Nervous System Listeriosis Confused with Leptomeningeal Carcinomatosis in Cancer Patients [2][J]. American Journal of Clinical Oncology: Cancer Clinical Trials, 2004,27(2):211-212.

[206] Vazquez G S, Cazarez R G, Pizarro E M, et al. Central Nervous System Miliary Brain Metastasis Secondary to Breast Cancer: Case Report[J]. CUREUS, 2020,12(8).

[207] Tan Kendrick A. Cerebral metastasis proven 1 year after an embolic cerebral infarct from pleuropulmonary blastoma[J]. Pediatric Radiology, 2004,34(3):283.

[208] Keshava N, Woodall G, Keshava C, et al. Chemically-induced mouse lung tumors: Applications to human health assessments[J]. Environmental and Molecular Mutagenesis, 2014,55:S52.

[209] Kurbacher C M, Mallmann P K. Chemoprotection in anticancer therapy: The emerging role of amifostine (WR-2721)[J]. Anticancer Research, 1998,18(3 C):2203-2210.

[210] Vinothkumar R, Ceasar S A, Divyarupa A. Chemosuppressive effect of plumbagin on human non-small lung cancer cell xenotransplanted zebrafish[J]. Indian Journal of Cancer, 2017,54(1):253-256.

[211] Teng A E, Noor B, Ajijola O A, et al. Chemotherapy and Radiation-Associated Cardiac Autonomic Dysfunction[J]. Current Oncology Reports, 2021,23(2).

[212] Carnio S, Galetta D, Scotti V, et al. Chemotherapy-Induced Nausea and Vomiting (CINV) in Italian lung cancer patients: Assessment by physician, nurse and patient[J]. Journal of Thoracic Oncology, 2017,12(1):S898.

[213] Mushtaq M, Wani M A, Asimi R, et al. Choriocarcinoma presenting as intracerebral hemarrhage and no evident primary: A rare presentation[J]. Indian Journal of Public Health Research and Development, 2016,7(1):148-151.

[214] Bhama S, Henegan J C. Choriocarcinoma syndrome[J]. Journal of Investigative Medicine, 2018,66(2):436.

[215] De Lorenzo B, Novaes E B R, Paslar L T, et al. Chronic Sleep Restriction Impairs the Antitumor Immune Response in Mice[J]. Neuroimmunomodulation, 2018,25(2):59-67.

[216] Mocellin S, Tropea S, Benna C, et al. Circadian pathway genetic variation and cancer risk: evidence from genome-wide association studies[J]. BMC MEDICINE, 2018,16.

[217] Kang H H, Kwon H Y, Kim I K, et al. CIRCULATING EXOSOMES IN OBSTRUCTIVE SLEEP APNEA INCREASED THE GROWTH AND THE PROLIFERATION OF LUNG CANCER CELLS THROUGH ACTIVATION OF ERK/C-MYC AXIS AND DECREASED CYTOTOXICITY OF CISPLATIN[J]. RESPIROLOGY, 2019,24:207.

[218] Ocvirk J, Boc M, Rebersek M, et al. Cisplatin-induced non-convulsive posterior reversible encephalopathy syndrome in a 41-year-old woman with metastatic malignant melanoma[J]. Radiology and Oncology, 2009,43(2):120-125.

[219] Spatola M, Sabater L, Planaguma J, et al. Clinical findings, IGG subclass, and antibody effects in encephalitis associated with metabotropic glutamate receptor 5 (MGLUR5) antibodies[J]. Neurology, 2018,90(15).

[220] Lupi I, Brancatella A, Cosottini M, et al. Clinical heterogeneity of hypophysitis secondary to PD-1/PD-l1 blockade: Insights from four cases[J]. Endocrinology, Diabetes and Metabolism Case Reports, 2019,2019(1).

[221] Zhang B, Zhang X, Zhou T, et al. Clinical observation of liver cancer patients treated with axitinib and cabozantinib after failed sorafenib treatment: A case report and literature review[J]. Cancer Biology and Therapy, 2015,16(2):215-218.

[222] Madden K, Tanco K, Bruera E. Clinically significant drug-drug interaction between methadone and cannabidiol[J]. Pediatrics, 2020,145(6).

[223] Koga M, Nakadozono M, Nukariya K, et al. Clonazepam for chemotherapy-induced nausea and vomiting (CINV)[J]. Anticancer Research, 2008,28(4 C):2433-2436.

[224] Ghanem M M, Battelli L A, Law B F, et al. Coal dust alters β-naphthoflavone-induced aryl hydrocarbon receptor nuclear translocation in alveolar type II cells[J]. Particle and Fibre Toxicology, 2009,6.

[225] Tsuzuki T, Ninomiya H, Natori Y, et al. Coalescent pleural malignant mesothelioma and adenocarcinoma of the lung, involving only minor asbestos exposure[J]. Pathology International, 2008,58(7):451-455.

[226] Ohlfest J R, Demorest Z L, Motooka Y, et al. Combinatorial antiangiogenic gene therapy by nonviral gene transfer using the Sleeping Beauty transposon causes tumor regression and improves survival in mice bearing intracranial human glioblastoma[J]. MOLECULAR THERAPY, 2005,12(5):778-788.

[227] Siripongboonsitti T. Community-acquired monomicrobial Pseudomonas aeruginosa necrotizing fasciitis: A case report[J]. Journal of the Medical Association of Thailand, 2018,101(6):S193-S198.

[228] Jones R A, Franks S E, Moorehead R A. Comparative mRNA and miRNA transcriptome analysis of a mouse model of IGFIR-driven lung cancer[J]. PLoS ONE, 2018,13(11).

[229] Mazzoccoli G, Sothern R B, Parrella P, et al. Comparison of circadian characteristics for cytotoxic lymphocyte subsets in non-small cell lung cancer patients versus controls[J]. CLINICAL AND EXPERIMENTAL MEDICINE, 2012,12(3):181-194.

[230] Zhu S, Shi J, Zhang K, et al. Comparison of napsin a expression in tumors with polyclonal and monoclonal antibodies[J]. Laboratory Investigation, 2012,92:494A.

[231] Stoner G D, Conran P B, Greisiger E A. Comparison of two routes of chemical administration on the lung adenoma response in strain A/J mice[J]. Toxicology and Applied Pharmacology, 1986,82(1):19-31.

[232] Kleef R, Moss R, Szasz A M, et al. Complete Clinical Remission of Stage IV Triple-Negative Breast Cancer Lung Metastasis Administering Low-Dose Immune Checkpoint Blockade in Combination With Hyperthermia and Interleukin-2[J]. Integrative Cancer Therapies, 2018,17(4):1297-1303.

[233] Valenza C, Porta F M, Rappa A, et al. Complex Differential Diagnosis between Primary Breast Cancer and Breast Metastasis from EGFR-Mutated Lung Adenocarcinoma: Case Report and Literature Review[J]. CURRENT ONCOLOGY, 2021,28(5):3384-3392.

[234] Bracamontes M D C V, Danielson L, Patel P. COMPOUNDED EFFECT: A CASE OF INTERCOSTAL LUNG HERNIATION[J]. Chest, 2022,162(4):A1820.

[235] Chen B, Wei W, Ma L, et al. Computational Discovery of Niclosamide Ethanolamine, a Repurposed Drug Candidate That Reduces Growth of Hepatocellular Carcinoma Cells In Vitro and in Mice by Inhibiting Cell Division Cycle 37 Signaling[J]. GASTROENTEROLOGY, 2017,152(8):2022-2036.

[236] Jassim S H, Khiyami A, Nguyen J K, et al. Concordant clear cell "mesonephric" carcinoma of the bladder and lung adenocarcinoma with clear cell features - multiple primaries versus metastatic neoplasms: a case report[J]. Journal of Medical Case Reports, 2017,11(1).

[237] Bobko A A, Evans J, Denko N C, et al. Concurrent Longitudinal EPR Monitoring of Tissue Oxygenation, Acidosis, and Reducing Capacity in Mouse Xenograft Tumor Models[J]. Cell biochemistry and biophysics, 2017,75(2):247-253.

[238] Cristian M, Bosoteanu M, Aschie M, et al. Concurrent Tumors Revealed by an Autopsy-A Case Report and Literature Review[J]. CASE REPORTS IN GASTROINTESTINAL MEDICINE, 2022,2022.

[239] Pichel J G, Pais R S, Zubeldia J M, et al. Conditional deletion of insulin-like growth factor 1 receptor in the lung epithelium of mutant mice[J]. FEBS Journal, 2012,279:535.

[240] Gu C, Jun J C. Continuous positive airway pressure titration: A minor change can make a major difference[J]. Annals of the American Thoracic Society, 2018,15(9):1105-1107.

[241] Pacenta H L, Kaddoum R N, Pereiras L A, et al. Continuous tunnelled femoral nerve block for palliative care of a patient with metastatic osteosarcoma[J]. Anaesthesia and Intensive Care, 2010,38(3):563-565.

[242] Bonneau A. Cough in the palliative care setting[J]. Canadian Family Physician, 2009,55(6):600-602.

[243] Flitsch J, Schröder F, Hagel C, et al. Cranial neuroendocrine carcinoma primarily diagnosed as malignant paraganglioma with rapid progress to a fatal outcome[J]. Acta Neurochirurgica, 2001,143(5):523-524.

[244] Tangchang W, Kim Y, Oh Y I, et al. Critical diagnostic and cancer stem cell markers in neoplastic cells from canine primary and xenografted pulmonary adenocarcinoma[J]. Journal of Veterinary Science, 2022,23(6).

[245] Kamala L H, Ranjith S, Benson R. Cutaneous Acral Metastasis from Renal Cell Carcinoma—a Case Report[J]. Indian Journal of Surgical Oncology, 2019.

[246] Zhang X, Fu Z, Yan C. Cytokine release syndrome induced by pembrolizumab: A case report[J]. Medicine (United States), 2022,101(49):E31998.

[247] Hu X, Chen Y, Ru G, et al. Cytological Features of Pulmonary Papillary Adenoma with Malignant Transformation and Literature Review[J]. Analytical Cellular Pathology, 2020,2020.

[248] Abu-Salah A K, Segura S, Cramer H M. Cytomorphologic findings of SMARCA4-deficient thoracic sarcoma/carcinoma: Report of two cases[J]. American Journal of Clinical Pathology, 2021,156(SUPPL 1):S39.

[249] Hayashi T, Haba R, Tanizawa J, et al. Cytopathologic features and differential diagnostic considerations of primary lymphoepithelioma-like carcinoma of the lung[J]. Diagnostic Cytopathology, 2012,40(9):820-825.

[250] Hayashi T, Haba R, Kushida Y, et al. Cytopathologic findings and differential diagnostic considerations of primary clear cell carcinoma of the lung[J]. Diagnostic Cytopathology, 2013,41(6):550-554.

[251] Snuderl M, Triscott J, Northcott P A, et al. Deep sequencing identifies IDH1 R132S mutation in adult medulloblastoma[J]. Journal of Clinical Oncology, 2015,33(6):e27-e31.

[252] Sim B L H, Loh S R H, Ng A C W, et al. Delayed nocturnal stridor from bilateral vocal cord paralysis post thyroidectomy surgery masquerading as obstructive sleep apnea[J]. Otolaryngology Case Reports, 2023,26.

[253] Shrotriya S, Rai M P, Alratroot A, et al. Delayed presentation of isolated adrenocorticotropin insufficiency after nivolumab therapy for advanced non-small-cell lung carcinoma (NSCLC)[J]. BMJ Case Reports, 2018,2018.

[254] Blackburn K, Vernino S. Delirium and tremulousness in a patient with cancer[J]. Journal of Clinical Neuromuscular Disease, 2017,18:S15-S16.

[255] Quintana M D, Bautista J M S, López M F, et al. Delirium masking syndrome[J]. European Geriatric Medicine, 2022,13:S209.

[256] Takeuchi N, Makino T, Nishihara M. Delirium with visual hallucinations induced by low-dose olanzapine[J]. Psychogeriatrics, 2022,22(3):415-416.

[257] Laaksonen R, Niiranen A, Iivanainen M, et al. Dementia-like, largely reversible syndrome after cranial irradiation and prolonged interferon treatment[J]. Annals of Clinical Research, 1988,20(3):201-203.

[258] Hallur V, Singh G, Rudramurthy S M, et al. Demodex mite infestation of unknown significance in a patient with rhinocerebral mucormycosis due to Apophysomyces elegans species complex[J]. Journal of Medical Microbiology, 2013,62(PART6):926-928.

[259] Brown J R, Yang F, Sinha A, et al. Deoxygenated disaccharide analogs as specific inhibitors of β1-4-galactosyltransferase 1 and selectin-mediated tumor metastasis[J]. Journal of Biological Chemistry, 2009,284(8):4952-4959.

[260] Xiao Y, Elkins K, Durieux J K, et al. Dependence of tumor cell lines and patient-derived tumors on the NAD salvage pathway renders them sensitive to NAMPT inhibition with GNE-6181[J]. Neoplasia (United States), 2013,15(10):1151-1160.

[261] Asghar-Ali A A, Wagle K C, Braun U K. Depression in terminally ILL patients: Dilemmas in diagnosis and treatment[J]. Journal of Pain and Symptom Management, 2013,45(5):926-933.

[262] Guevara E, Schreiber N. Depression, or something else?[J]. Current Psychiatry, 2020,19(3):43-49.

[263] Ge C, Chang L, Zhao Y, et al. Design, Synthesis and Evaluation of Naphthalimide Derivatives as Potential Anticancer Agents for Hepatocellular Carcinoma[J]. Molecules (Basel, Switzerland), 2017,22(2).

[264] Dai F, Li Q, Wang Y, et al. Design, Synthesis, and Biological Evaluation of Mitochondria-Targeted Flavone-Naphthalimide-Polyamine Conjugates with Antimetastatic Activity[J]. Journal of Medicinal Chemistry, 2017,60(5):2071-2083.

[265] Peduto A, Pagano B, Petronzi C, et al. Design, synthesis, biophysical and biological studies of trisubstituted naphthalimides as G-quadruplex ligands[J]. Bioorganic and Medicinal Chemistry, 2011,19(21):6419-6429.

[266] Biswas P, Datta H K, Dastidar P. Designing coordination polymers as multi-drug-self-delivery systems for tuberculosis and cancer therapy: in vitro viability and in vivo toxicity assessment[J]. Biomaterials Science, 2022,10(21):6201-6216.

[267] Iveson P, Morrison M, Barnett J. Development of a 99mTc-labelled RGD peptide as an angiogenesis imaging agent[J]. Nuclear Medicine and Biology, 2010,37(6):707.

[268] Hill T, Conolly R B. Development of a Novel AOP for Cyp2F2-Mediated Lung Cancer in Mice[J]. Toxicological Sciences, 2019,172(1):1-10.

[269] Nagaraj S K, Manjunath Y, Radhakrishnan V, et al. Development of CTC-derived xenograft (CDX) mouse models from early-stage non-small cell lung cancer patients[J]. Clinical Cancer Research, 2020,26(11 SUPPL).

[270] Leban J J, Kull Jr. F C, Landavazo A, et al. Development of potent gastrin-releasing peptide antagonists having a D- Pro-Ψ(CH2NH)-Phe-NH2 C terminus[J]. Proceedings of the National Academy of Sciences of the United States of America, 1993,90(5):1922-1926.

[271] Ruiz-Bañobre J, Pérez-Pampín E, García-González J, et al. Development of psoriatic arthritis during nivolumab therapy for metastatic non-small cell lung cancer, clinical outcome analysis and review of the literature[J]. Lung Cancer, 2017,108:217-221.

[272] Hsiao C H, Wang C Y, Chung M T, et al. Diabetes insipidus due to pituitary metastasis in a woman with lung adenocarcinoma: A case report[J]. Central European Journal of Medicine, 2011,6(4):475-479.

[273] Gil S M, Picon N, Ballarino C. Diabetes insipidus in a patient with leukemia and lung cancer[J]. Endocrine Reviews, 2017,38(3).

[274] Baqir A W, Khader S, Lajara S. Diagnosis of Large Cell Neuroendocrine Carcinomas on Cytology Specimens: How Reliable Are We?[J]. Journal of the American Society of Cytopathology, 2022,11(6):S50-S51.

[275] Kumar S, Amara S, Sankhyan P, et al. Diagnostic Dilemma of an Anterior Mediastinal Mass[J]. American Journal of Respiratory and Critical Care Medicine, 2021,203(9).

[276] Zhang Y Y, Ding J N, Shen G F, et al. Dietary and inhalation exposure to polycyclic aromatic hydrocarbons and urinary excretion of monohydroxy metabolites - A controlled case study in Beijing, China[J]. ENVIRONMENTAL POLLUTION, 2014,184:515-522.

[277] Fukami T, Nakajima M, Zen Y, et al. Differences in CYP2A13 expression level in various types of human lung cancer[J]. Drug Metabolism Reviews, 2009,41:31.

[278] Khanduja K L, Jnagal S, Hundal M K, et al. Differential effects of diclofenac and naproxen on N-nitrosodiethylamine induced lung tumorigenesis[J]. Medecine Biologie Environnement, 1999,27(1):83-87.

[279] Ozbun L L, Martinez A, Angdisen J, et al. Differentially expressed nucleolar TGF-beta 1 target (DENTT) in mouse development[J]. DEVELOPMENTAL DYNAMICS, 2003,226(3):491-511.

[280] Ozbun L L, Martínez A, Angdisen J, et al. Differentially expressed nucleolar TGF-beta1 target (DENTT) in mouse development[J]. Dev Dyn, 2003,226(3):491-511.

[281] Ozbun L L, Martínez A, Angdisen J, et al. Differentially expressed nucleolar TGF-β1 target (DENTT) in mouse development[J]. Developmental Dynamics, 2003,226(3):491-511.

[282] Plowman K, Ruthman C. Diffuse arterial embolization secondary to pulmonary vein thrombosis[J]. Respiratory Medicine Case Reports, 2022,39.

[283] Kapuria D, Strasser K, Qasem A. Diffuse large B-cell lymphoma causing acute liver failure: A rare case of survival[J]. BMJ Case Reports, 2015,2015.

[284] Bodyak N, Yurkovetskiy A, Yin M, et al. Discovery and preclinical development of a highly potent NaPi2b-targeted antibody-drug conjugate (ADC) with significant activity in patientderived non-small cell lung cancer (NSCLC) xenograft models[J]. Cancer Research, 2016,76(14).

[285] Pan S, Zhou Y, Wang Q, et al. Discovery and structure-activity relationship studies of 1-aryl-1H-naphtho[2,3-d][1,2,3]triazole-4,9-dione derivatives as potent dual inhibitors of indoleamine 2,3-dioxygenase 1 (IDO1) and trytophan 2,3-dioxygenase (TDO)[J]. European Journal of Medicinal Chemistry, 2020,207.

[286] Shi C, Wang Q, Liao X, et al. Discovery of 6-(2-(dimethylamino)ethyl)-N-(5-fluoro-4-(4-fluoro-1-isopropyl-2-methyl-1H-benzo[d]imidazole-6-yl)pyrimidin-2-yl)-5,6,7,8-tetrahydro-1,6-naphthyridin-2-amine as a highly potent cyclin-dependent kinase 4/6 inhibitor for treatment of cancer[J]. European Journal of Medicinal Chemistry, 2019,178:352-364.

[287] Delaye M, Laurent S, Try M, et al. Discussion on methadone initiation protocols: A case report[J]. Journal of Opioid Management, 2022,18(5):487-491.

[288] Shinar Y R, Marks A D. Distressing Visions at the End of Life: Case Report and Review of the Literature[J]. J Pastoral Care Counsel, 2015,69(4):251-253.

[289] Sephton S E, Lush E, Dedert E A, et al. Diurnal cortisol rhythm as a predictor of lung cancer survival[J]. Brain Behav Immun, 2013,30 Suppl:S163-S170.

[290] Lee J H, Lee E O, Lee H J, et al. DMNQ S-53 induces apoptosis and inhibits the growth of Lewis lung carcinoma cells in vitro and in vivo[J]. Journal of Cardiothoracic-Renal Research, 2006,1(1):73-79.

[291] Yohannan S, Alhariri S, Deoker A. Don't look at my belly, look at my heart-pericardial effusion from an occult malignancy[J]. Journal of Investigative Medicine, 2022,70(2):567.

[292] Veneroni L, Ferrari A, Proserpio T, et al. Dreams and illusions in adolescents with terminal cancer[J]. Tumori, 2018,104(6):413-414.

[293] Ann-Yi S, Azhar A, Bruera E. Dying Alone during a Pandemic[J]. Journal of Palliative Medicine, 2021,24(12):1905-1908.

[294] Izzotti A, Balansky R, Ganchev G, et al. Early and late effects of aspirin and naproxen on microRNAs in the lung and blood of mice, either unexposed or exposed to cigarette smoke[J]. Oncotarget, 2017,8(49):85716-85748.

[295] Atreya S. Early integration of palliative medicine into emergency care: Is it a feasible option[J]. Indian Journal of Medical and Paediatric Oncology, 2016,37(3):202-205.

[296] Guo X F, Liu Y, Kim J L, et al. Effect of cyclical intermittent hypoxia on Ad5CMVCre induced solitary lung cancer progression and spontaneous metastases in the Kras(G12D+); p53(fl/fl); myristolated p110(fl/fl) ROSA-gfp mouse[J]. PLOS ONE, 2019,14(2).

[297] Stowell C P, Nollie D L, Jean-Baptiste M, et al. Effectiveness of antiglucocorticoid therapy in a patient with psychotic depression and ectopic ACTH production[J]. Journal of Psychiatric Practice, 2003,9(4):321-323.

[298] Araya-Maturana R, Cardona W, Cassels B K, et al. Effects of 9,10-dihydroxy-4,4-dimethyl-5,8-dihydro-1(4H)-anthracenone derivatives on tumor cell respiration[J]. Bioorganic and Medicinal Chemistry, 2006,14(13):4664-4669.

[299] Jalbert G, Castonguay A. Effects of NSAIDs on NNK-induced pulmonary and gastric tumorigenesis in A/J mice[J]. Cancer Letters, 1992,66(1):21-28.

[300] Chao W R, Hobbs P D, Jong L, et al. Effects of receptor class- and subtype-selective retinoids and an apoptosis-inducing retinoid on the adherent growth of the NIH:OVCAR-3 ovarian cancer cell line in culture[J]. Cancer Letters, 1997,115(1):1-7.

[301] Fang T, Huang H, Li X, et al. Effects of siRNA silencing of TUG1 and LCAL6 long non-coding RNAs on patient-derived xenograft of non-small cell lung cancer[J]. Anticancer Research, 2018,38(1):179-186.

[302] McRipley R J, Burns-Horwitz P E, Czerniak P M, et al. Efficacy of DMP 840: A novel bis-naphthalimide cytotoxic agent with human solid tumor xenograft selectivity[J]. Cancer Research, 1994,54(1):159-164.

[303] Alhoshani A, Alanazi F E, Alotaibi M R, et al. EGFR Inhibitor Gefitinib Induces Cardiotoxicity through the Modulation of Cardiac PTEN/Akt/FoxO3a Pathway and Reactive Metabolites Formation: In Vivo and in Vitro Rat Studies[J]. Chemical Research in Toxicology, 2020,33(7):1719-1728.

[304] Harada C, Kawaguchi T, Ogata-Suetsugu S, et al. EGFR tyrosine kinase inhibition worsens acute lung injury in mice with repairing airway epithelium[J]. American Journal of Respiratory and Critical Care Medicine, 2011,183(6):743-751.

[305] Harada C, Kawaguchi T, Ogata-Suetsugu S, et al. EGFR Tyrosine Kinase Inhibition Worsens Acute Lung Injury in Mice with Repairing Airway Epithelium[J]. AMERICAN JOURNAL OF RESPIRATORY AND CRITICAL CARE MEDICINE, 2011,183(6):743-751.

[306] Grabske R, Azevedo A, Smith R E. Elevated proteinase activities in mouse lung tumors quantitated by synthetic fluorogenic substrates[J]. Journal of Histochemistry and Cytochemistry, 1979,27(11):1505-1508.

[307] Oliver J R, Kushwah R, Wu J, et al. Elf3 plays a role in regulating bronchiolar epithelial repair kinetics following clara cell-specific injury[J]. Laboratory Investigation, 2011,91(10):1514-1529.

[308] Feng K, Ma C, Liu Y, et al. Encapsulation of LXR ligand by D-Nap-GFFY hydrogel enhances anti-tumorigenic actions of LXR and removes LXR-induced lipogenesis[J]. Theranostics, 2021,11(6):2634-2654.

[309] Martínez-Vila C, Laguna J C, Segui E, et al. Encephalitis associated with immune checkpoint inhibitor treatment in patients with melanoma[J]. Journal of Immunotherapy, 2021,44(5):204-207.

[310] Schimmel M E, Van Nostrand K. Endobronchial liposuction: Excision of endobronchial lipoma in patient with pulmonary hamartoma[J]. American Journal of Respiratory and Critical Care Medicine, 2018,197(MeetingAbstracts).

[311] Ko H L, Beuth J, Tunggal L, et al. Enhancement of immune responses to suramin correlates with antineoplastic activity in BALB/c-mice[J]. In Vivo, 1994,8(2):173-176.

[312] Xu X, Fan Y. EP08.02-165 Small Cell Transformation After Lorlatinib ALK Resistance - A Case Report with Clonal Evolution[J]. Journal of Thoracic Oncology, 2022,17(9):S485.

[313] Tachibana T, Omori T, Uchida O, et al. EP1.09-16 A Case of Pulmonary Primary Enteric Adenocarcinoma Diagnosed Preoperatively[J]. Journal of Thoracic Oncology, 2019,14(10):S1004.

[314] Dai J Y, Hong A L, Wang Y. Epidural Block Treatment on Postherpetic Neuralgia and Comorbid Spine Metastasis of Malignant Tumor: Two Cases of Report[J]. International Journal of Dermatology and Venereology, 2021,4(1):53-55.

[315] Wang H H, Zhang Y Y, Wang B, et al. Epithelial-myoepithelial carcinoma of the parotid gland with primary lung cancer A rare case report[J]. MEDICINE, 2020,99(40).

[316] Wang H, Zhang Y, Wang B, et al. Epithelial-myoepithelial carcinoma of the parotid gland with primary lung cancer: A rare case report[J]. Medicine, 2020,99(40):e22483.

[317] Karasawa T, Kudo K, Tanita K, et al. Epstein-Barr Virus–Negative Granulomatous Disease Due to SAP Deficiency[J]. Journal of Clinical Immunology, 2021,41(6):1372-1375.

[318] Schiopu S R I, Käsmann L, Schönermarck U. Erratum: Pembrolizumab-induced myocarditis in a patient with malignant mesothelioma: Plasma exchange as a successful emerging therapy-case report (Transl Lung Cancer Res (2021) 10 (1039-1046) DOI: 10.21037/tlcr-20-1095)[J]. Translational Lung Cancer Research, 2021,10(6):3029.

[319] King J. ES19.05 Incorporating Patient Reported Outcomes into the Lung Cancer Registry[J]. Journal of Thoracic Oncology, 2021,16(3):S86.

[320] Gu X, Zhang J, Shi Y H, et al. ESM1/HIF-1 alpha pathway modulates chronic intermittent hypoxia-induced non-small-cell lung cancer proliferation, stemness and epithelial-mesenchymal transition[J]. ONCOLOGY REPORTS, 2021,45(3):1226-1234.

[321] Huang M, Jin J, Wu Y, et al. Establishment and biological characteristics of a gefitinib-resistant poorly differentiated lung carcinoma cell line[J]. Journal of Practical Oncology, 2020,35(2):127-133.

[322] Jiang Y, Zhao J, Zhang Y, et al. Establishment of lung cancer patient-derived xenograft models and primary cell lines for lung cancer study[J]. Journal of Translational Medicine, 2018,16(1).

[323] Johnson K N, Gonzalez P M, Sepulveda M, et al. Establishment,characterization and utilization of models of central nervous system metastasis[J]. Cancer Research, 2014,74(19).

[324] Stelck R L, Baker G L, Sutherland K M, et al. Estrous cycle alters naphthalene metabolism in female mouse airways[J]. DRUG METABOLISM AND DISPOSITION, 2005,33(11):1597-1602.

[325] Khalyfa A, Almendros I, Gileles-Hillel A, et al. Exosomes released into the circulation under chronic sleep fragmentation potentiate tumor malignancy in a mouse model of sleep apnea[J]. American Journal of Respiratory and Critical Care Medicine, 2015,191.

[326] Kakinuma R, Nishiwaki Y, Yano H, et al. Experience with psychotropic agents Pyrethia and Contomin in the terminal care of lung cancer patients[J]. Gan no rinsho. Japan journal of cancer clinics, 1984,30(4):344-348.

[327] Martinez A, Ozbun L L, Angdisen J, et al. Expression of differentially expressed nucleolar transforming growth factor-beta 1 target (DENTT) in adult mouse tissues[J]. DEVELOPMENTAL DYNAMICS, 2002,224(2):186-199.

[328] Martínez A, Ozbun L L, Angdisen J, et al. Expression of differentially expressed nucleolar transforming growth factor-beta1 target (DENTT) in adult mouse tissues[J]. Dev Dyn, 2002,224(2):186-199.

[329] Martínez A, Ozbun L L, Angdisen J, et al. Expression of differentially expressed nucleolar transforming growth factor-β1 target (DENTT) in adult mouse tissues[J]. Developmental Dynamics, 2002,224(2):186-199.

[330] Liu Y L, Lu M Z, Chen J A, et al. Extracellular vesicles derived from lung cancer cells exposed to intermittent hypoxia upregulate programmed death ligand 1 expression in macrophages[J]. SLEEP AND BREATHING, 2022,26(2):893-906.

[331] Devereux T R, Anderson M W, Belinsky S A. Factors regualting activation and DNA alkylation by 4-(N-methyl-N-nitrosamino)-1-(3-pyridyl)-1-butanone and nitrosodimethylamine in rat lung and isolated lung cells, and the relationship to carcinogenicity[J]. Cancer Research, 1988,48(15):4215-4221.

[332] Jacobs S, Maldonado-Slootjes S, Verhulst D, et al. Fatal encephalopathy with brainstem involvement under dabrafenib and trametinib in a BRAF-positive metastatic melanoma[J]. Revue Neurologique, 2021,177(9):1195-1198.

[333] Zawadzki M, Magdalan J, Szpot P. Fatal iatrogenic vinorelbine poisoning: A case report[J]. Anti-Cancer Drugs, 2018,30(1):89-90.

[334] Hassam T, Ramasubbu K. Fatal myocarditis: A rare but life threatening adverse effect of nivolumab chemotherapy[J]. Chest, 2017,152(4):A692.

[335] Bramness J G, Arnestad M, Karinen R, et al. Fatal overdose of zopiclone in an elderly woman with bronchogenic carcinoma[J]. Journal of Forensic Sciences, 2001,46(5):1247-1249.

[336] Willemsen A E C A, Van Herpen C M L, Wesseling P, et al. Fatal thrombotic microangiopathy after a single dose of gemcitabine as fourth-line palliative treatment for metastasized ductal breast carcinoma[J]. Acta Oncologica, 2011,50(3):462-465.

[337] Yomo S, Hayashi M. Fatal tumoral hemorrhage after stereotactic radiosurgery for metastatic brain tumors: Report of three cases and review of literature[J]. Acta Neurochirurgica, 2012,154(9):1685-1690.

[338] Sola F, Farao M, Ciomei M, et al. FCE 27266, a sulfonic distamycin derivative, inhibits experimental and spontaneous lung and liver metastasis[J]. Invasion and Metastasis, 1995,15(5-6):222-231.

[339] Cynkier P. Fear of imminent death – use of evidence in forensic-psychiatric expertise[J]. Psychiatria Polska, 2019,53(1):191-201.

[340] Santos I R, Raiter J, Lamego É C, et al. Feline pulmonary carcinoma: Gross, histological, metastatic, and immunohistochemical aspects[J]. Veterinary Pathology, 2023,60(1):8-20.

[341] Anderson L M, Ruskie S, Carter J, et al. Fetal mouse susceptibility to transplacental carcinogenesis: Differential influence of Ah receptor phenotype on effects of 3-methylcholanthrene, 12-dimethylbenz[a]anthracene, and benzo[a]pyrene[J]. Pharmacogenetics, 1995,5(6):364-372.

[342] Bhandari B S, Rawal H, Yeramareddy S, et al. Finding tumor: The misadventures of a papillary adenocarcinoma of lung[J]. American Journal of Respiratory and Critical Care Medicine, 2019,199(9).

[343] Lallu S, Naran S, Bethwaite P. Fine needle aspiration cytology of pulmonary papillary adeno-carcinoma mimicking papillary thyroid carcinoma[J]. New Zealand Journal of Medical Laboratory Science, 2018,72(3):107-110.

[344] Song H, Yao E, Lin C, et al. Functional characterization of pulmonary neuroendocrine cells in lung development, injury,and tumorigenesis[J]. Proceedings of the National Academy of Sciences of the United States of America, 2012,109(43):17531-17536.

[345] Tow S, Carozza D, Barker K. Functional Impairments in a Patient with Morvan Syndrome: A Case Presentation[J]. PM and R, 2018,10(7):766-769.

[346] Walters M A, Roe F J, Mitchley B C, et al. Further tests for carcinogenesis using newborn mice: 2-naphthylamine, 2-naphthylhydroxylamine, 2-acetylaminofluorene and ethyl methane sulphonate.[J]. British journal of cancer, 1967,21(2):367-372.

[347] Hernández J L, Pajarón M, García-Regata O, et al. Gabapentin for intractable hiccup [2][J]. American Journal of Medicine, 2004,117(4):279-281.

[348] Ross J R, Waight C, Riley J. Gabapentin: Resistant neuropathic pain and malignancy [3][J]. Palliative Medicine, 2001,15(4):348-349.

[349] Ghanimeh M A, Bourbia A, Sadeddin E, et al. Gastric and breast metastasis from lung adenocarcinoma: A case report[J]. American Journal of Gastroenterology, 2015,110:S511.

[350] Catalano M, Marini A, Ferrari K, et al. Gastric and colonic metastasis from NSCLC A very unusual case report[J]. MEDICINE, 2022,101(2).

[351] Zhu M, Shu J, Liu X, et al. Gastrointestinal hemorrhage caused by duodenal metastasis from a primary lung adenocarcinoma: A case report[J]. Molecular and Clinical Oncology, 2021,14(3):1-5.

[352] Gouloumis A R, Tsakiraki Z, Zacharatou A, et al. Gastrointestinal stromal tumour metastasis to the lung with chondrosarcomatous differentiation. Report of a case[J]. Virchows Archiv, 2021,479(SUPPL 1):S212-S213.

[353] Shaker N, Hanline C, Tynski I M, et al. GATA3 expression in pulmonary mucinous adenocarcinoma presenting as a distant metastasis: A case report[J]. Human Pathology Reports, 2022,28.

[354] Beigi M, Häberle M, Gschwendtner A, et al. Generalized chronic itch as a first sign of malignancy resembling paraneoplastic sensomotoric neuropathy[J]. Acta Dermato-Venereologica, 2018,98(5):526-527.

[355] Kashima J, Hishima T, Tonooka A, et al. Genetic and immunohistochemical analyses of ciliated muconodular papillary tumors of the lung: A report of five cases[J]. SAGE Open Medical Case Reports, 2019,7.

[356] Weiss J B, Weber S J, Torres E, et al. Genetic inhibition of Anaplastic Lymphoma Kinase rescues cognitive impairments in Neurofibromatosis 1 mutant mice[J]. BEHAVIOURAL BRAIN RESEARCH, 2017,321:148-156.

[357] Shen J Y, Zhou H Q, Liu J Q, et al. Genetic Liability to Insomnia and Lung Cancer Risk: A Mendelian Randomization Analysis[J]. FRONTIERS IN GENETICS, 2021,12.

[358] Huo Z, Ge F, Li C, et al. Genetically predicted insomnia and lung cancer risk: a Mendelian randomization study[J]. Sleep Med, 2021,87:183-190.

[359] Kurtzberg L S, Roth S, Krumbholz R, et al. Genz-644282, a novel non-camptothecin topoisomerase I inhibitor for cancer treatment[J]. Clinical Cancer Research, 2011,17(9):2777-2787.

[360] Fioravanzo A, Simbolo M, Giampiccolo D, et al. Glioblastoma with carcinomatous differentiation or tumor-to-tumor metastasis?[J]. Clinical Neuropathology, 2019,38(3):132-133.

[361] Fioravanzo A, Simbolo M, Giampiccolo D, et al. Glioblastoma with carcinomatous differentiation or tumour-totumour metastasis?[J]. Virchows Archiv, 2019,475:S379-S380.

[362] Nielsen Z J K. Hemidiaphragmatic paresis after interscalene supplementation of insufficient axillary block with 3 mL of 2% mepivacaine[J]. Acta Anaesthesiologica Scandinavica, 2000,44(9):1160-1162.

[363] Qian X, Li Z, Yang Q. Highly efficient antitumor agents of heterocycles containing sulfur atom: Linear and angular thiazonaphthalimides against human lung cancer cell in vitro[J]. Bioorganic and Medicinal Chemistry, 2007,15(21):6846-6851.

[364] Matsuzaki O. Histogenesis and growing patterns of lung tumors induced by potassium 1-methyl-1,4-dihydro-7-(2-(5-nitrofuryl)vinyl)-4-oxo-1,8-naphthyridine-3-carboxylate in ICR mice.[J]. Gann = Gan, 1975,66(3):259-267.

[365] Laricchiuta P, Russo V, Costagliola A, et al. Histological and immunohistochemical characterisation of uterine adenocarcinoma in an Asian elephant (Elephas Maximus)[J]. Folia morphologica, 2018,77(4):771-774.

[366] Terasaki Y, Suzuki T, Ohsawa I, et al. Hydrogen-supplemented drinking water protects against naphthalene-gefitinib induced lung injury from inflammation-associated oxidative stress[J]. American Journal of Respiratory and Critical Care Medicine, 2014,189.

[367] Shopova M, Wohrle D, Stoichkova N, et al. Hydrophobic Zn(II)-naphthalocyanines as photodynamic therapy agents for Lewis lung carcinoma[J]. Journal of Photochemistry and Photobiology B: Biology, 1994,23(1):35-42.

[368] Rojas-Marcos I, Graus F, Sanz G, et al. Hypersomnia as presenting symptom of anti-Ma2-associated encephalitis: Case study[J]. Neuro-Oncology, 2007,9(1):75-77.

[369] Bailey L A, Nascarella M A, Kerper L E, et al. Hypothesis-based weight-of-evidence evaluation and risk assessment for naphthalene carcinogenesis[J]. Critical Reviews in Toxicology, 2016,46(1):1-42.

[370] Azarnier R G, Zuchelkowski A, Chung P E, et al. Identification of oncogenic/metastatic driver genes that cooperate with p53 or p53/Rb-loss to induce triple-negative breast cancer[J]. Cancer Research, 2017,77(13).

[371] Tomita T, Sakurai Y, Ishibashi S, et al. Imbalance of Clara cell-mediated homeostatic inflammation is involved in lung metastasis[J]. Oncogene, 2011,30(31):3429-3439.

[372] Eng H L, Liu H, Lin T M. Immune modulation of breast cancer with zoledronic acid in mouse model[J]. American Journal of Clinical Pathology, 2018,149:S148.

[373] Arcila M E, Rekhtman N, Teruja-Feldstein J. Immunohistochemical analysis of Napsin a in 77 lung carcinomas and 509 non-pulmonary malignancies[J]. Laboratory Investigation, 2010,90:397A.

[374] Ramos-Vara J A, Frank C B, DuSold D, et al. Immunohistochemical Detection of Pax8 and Napsin A in Canine Thyroid Tumours: Comparison with Thyroglobulin, Calcitonin and Thyroid Transcription Factor 1[J]. Journal of Comparative Pathology, 2016,155(4):286-298.

[375] Machado F C, Girola N, Maia V S C, et al. Immunomodulatory Protective Effects of Rb9 Cyclic-Peptide in a Metastatic Melanoma Setting and the Involvement of Dendritic Cells[J]. Frontiers in Immunology, 2020,10.

[376] Navas T, Pfister T D, Lawrence S M, et al. Impact of HGF knockin microenvironment on epithelial-mesenchymal transition and cancer stem cells in a non-small cell lung cancer xenograft model[J]. Cancer Research, 2015,75(15).

[377] Phipps A I, Bhatti P, Neuhouser M L, et al. Impact of sleep duration and quality on cancer survival[J]. Sleep, 2015,38:A293.

[378] Huang G B, Chen S, Qin Q P, et al. In vitro and in vivo activity of novel platinum(ii) complexes with naphthalene imide derivatives inhibiting human non-small cell lung cancer cells[J]. New Journal of Chemistry, 2019,43(21):8146-8152.

[379] Maciel L L F, de Freitas W R, Bull E S, et al. In vitro and in vivo anti-proliferative activity and ultrastructure investigations of a copper(II) complex toward human lung cancer cell NCI-H460[J]. Journal of Inorganic Biochemistry, 2020,210.

[380] Abe Y, Tsuda T, Okajima T. In vitro angiotensin-converting enzyme and interleukin-1 production by epithelioid cells isolated from induced rabbit lung granuloma[J]. Experimental Lung Research, 1990,16(5):489-505.

[381] Skoulidis F. Inactivating STK11/LKB1 genomic alterations are a major driver of primary resistance to PD- 1 axis blockade in non-squamous non-small cell lung cancer[J]. Cancer Research, 2019,79(13).

[382] Skoulidis F. Inactivating STK11/LKB1 genomic alterations are a major driver of primary resistance to PD-1 axis blockade in non-squamous non-small cell lung cancer[J]. Cancer Research, 2019,79(13).

[383] Varga J L, Schally A V, Horvath J E, et al. Increased activity of antagonists of growth hormone-releasing hormone substituted at positions 8, 9, and 10[J]. Proceedings of the National Academy of Sciences of the United States of America, 2004,101(6):1708-1713.

[384] Palmatier R N, Henry T, Rennard S I, et al. Induction and prevention of squamous transformation of bronchial epithelial cells by phenolic compounds[J]. Xenobiotica, 1997,27(12):1201-1214.

[385] Arita M, Watanabe S, Takahashi M, et al. Inflammation of the lung enhances antitumor effects of anti-PD-1 immunotherapy[J]. Cancer Science, 2018,109:947.

[386] Blakley B R. Influence of copper and zinc on urethan-induced adenoma development in mice[J]. Drug Nutr Interact, 1988,5(4):395-401.

[387] Zhang B, Liu Z, Hu X. Inhibiting cancer metastasis via targeting NAPDH oxidase 4[J]. Biochemical Pharmacology, 2013,86(2):253-266.

[388] Ito H, Wang J Z, Shimura K. Inhibition of lung metastasis by a calmodulin antagonist, N-(6-aminohexyl)-5-chloro-1-naphthalenesulfonamide (W-7), in mice bearing Lewis lung carcinoma[J]. Anticancer Research, 1991,11(1):249-252.

[389] Castonguay A, Rioux N, Duperron C, et al. Inhibition of lung tumorigenesis by NSAIDs: A working hypothesis[J]. Experimental Lung Research, 1998,24(4):605-615.

[390] Sampath D, Zabka T S, Misner D L, et al. Inhibition of nicotinamide phosphoribosyltransferase (NAMPT) as a therapeutic strategy in cancer[J]. Pharmacology and Therapeutics, 2015,151:16-31.

[391] De Azevedo Silva E E, De Morais Carvalho I M, Maciel E A. Inhibitory action of niketamide and chlordiazepoxide on lung carcinogenic activity of ethyl uretan in mice. Comments on iatrogenic carcinogenesis and anti carcinogenesis[J]. Revista Brasileira de Medicina, 1977,34(2):63-68.

[392] Kuroda K, Kanisawa M, Akao M. Inhibitory effect of fumaric acid on forestomach and lung carcinogenesis by a 5-nitrofuran naphthyridine derivative in mice[J]. Journal of the National Cancer Institute, 1982,69(6):1317-1320.

[393] Siripong P, Yahuafai J, Piyaviriyakul S, et al. Inhibitory effect of liposomal rhinacanthin-N isolated from Rhinacanthus nasutus on pulmonary metastasis in mice[J]. Biological and Pharmaceutical Bulletin, 2012,35(7):1197-1200.

[394] Shi J, Xia Y, Song Q, et al. Interaction of prostate cancer cells with tumor microenvironment promotes EMT and DTCs activation[J]. Cancer Research, 2014,74(19).

[395] Huang M H, Zhang X B, Wang H L, et al. Intermittent hypoxia enhances the tumor programmed death ligand 1 expression in a mouse model of sleep apnea[J]. ANNALS OF TRANSLATIONAL MEDICINE, 2019,7(5).

[396] Kang H S, Kwon H Y, Kim I K, et al. Intermittent hypoxia exacerbates tumor progression in a mouse model of lung cancer[J]. Sci Rep, 2020,10(1):1854.

[397] Kang H S, Kwon H Y, Kim I K, et al. Intermittent hypoxia exacerbates tumor progression in a mouse model of lung cancer[J]. SCIENTIFIC REPORTS, 2020,10(1).

[398] Almendros I, Montserrat J M, Torres M, et al. Intermittent hypoxia increases melanoma metastasis to the lung in a mouse model of sleep apnea[J]. Respir Physiol Neurobiol, 2013,186(3):303-307.

[399] Li L, Ren F, Qi C, et al. Intermittent hypoxia promotes melanoma lung metastasis via oxidative stress and inflammation responses in a mouse model of obstructive sleep apnea[J]. Respir Res, 2018,19(1):28.

[400] Almendros I, Wang Y, Becker L, et al. Intermittent hypoxia-induced changes in tumor-associated macrophages and tumor malignancy in a mouse model of sleep apnea[J]. Am J Respir Crit Care Med, 2014,189(5):593-601.

[401] Dubey A, Park D W, Kwon J E, et al. Investigation of the biological and anti-cancer properties of ellagic acid-encapsulated nano-sized metalla-cages[J]. International Journal of Nanomedicine, 2015,10:227-240.

[402] López I P, Piñeiro-Hermida S, Pais R S, et al. Involvement of Igf1r in bronchiolar epithelial regeneration: Role during repair kinetics after selective club cell ablation[J]. PLoS ONE, 2016,11(11).

[403] Akagi K, Sano M, Ogawa K, et al. Involvement of toxicity as an early event in urinary bladder carcinogenesis induced by phenethyl isothiocyanate, benzyl isothiocyanate, and analogues in F344 rats[J]. Toxicologic Pathology, 2003,31(4):388-396.

[404] Jian-Sheng L, Yuan-Yuan L, Ying-Ying X, et al. Isolation and identification of secondary metabolites from marine-derived actinomycete WBF16[J]. Chinese Journal of Antibiotics, 2012,37(4):258-260.

[405] Isoprene[Z]. 1999: 71 III, 1015-1025.

[406] Wang W J, He L R, Ouyang C, et al. Key Common Genes in Obstructive Sleep Apnea and Lung Cancer are Associated with Prognosis of Lung Cancer Patients[J]. INTERNATIONAL JOURNAL OF GENERAL MEDICINE, 2021,14:5381-5396.

[407] Buzalaf M A R, Taga E M, Granjeiro J M, et al. Kinetic characterization of bovine lung low-molecular-weight protein tyrosine phosphatase[J]. Experimental Lung Research, 1998,24(3):269-272.

[408] Chen Y, Wu Y, Li S, et al. Large-scale isolation and antitumor mechanism evaluation of compounds from the traditional Chinese medicine Cordyceps Militaris[J]. European Journal of Medicinal Chemistry, 2021,212.

[409] Skoulidis F, Hellman M D, Awad M, et al. LKB1 loss is a novel genomic predictor of De novo resistance to PD-1/ PD-L1 axis blockade in KRAS-mutant lung adenocarcinoma[J]. Annals of Oncology, 2017,28:v467.

[410] Ali F, Yamaguchi K, Fukuoka M, et al. Logical design of an anti-cancer agent targeting the plant homeodomain in Pygopus2[J]. Cancer Science, 2016,107(9):1321-1328.

[411] Xiong L, Cao F, Cao X, et al. Long-term-stable near-infrared polymer dots with ultrasmall size and narrow-band emission for imaging tumor vasculature in vivo[J]. Bioconjugate Chemistry, 2015,26(5):817-821.

[412] Linnoila R I, Jensen-Taubman S, Kazanjian A, et al. Loss of GFI1 impairs pulmonary neuroendorine cell proliferation, but the neuroendocrine phenotype has limited impact on post-naphthalene airway repair[J]. Laboratory Investigation, 2007,87(4):336-344.

[413] Torres M, Martinez-Garcia M A, Campos-Rodriguez F, et al. Lung cancer aggressiveness in an intermittent hypoxia murine model of postmenopausal sleep apnea[J]. MENOPAUSE-THE JOURNAL OF THE NORTH AMERICAN MENOPAUSE SOCIETY, 2020,27(6):706-713.

[414] Li Z, Feiyue Z, Gaofeng L, et al. Lung cancer and oncolytic virotherapy——enemy's enemy[J]. Translational Oncology, 2023,27.

[415] Dvorak K, Roessler C, Palting J, et al. Lung cancer classification using new immunohistochemical assay with anti-p40 (BC28) mouse monoclonal antibody: Comparison with the p40 Echelon assay and anti-p63 (4A4) antibody[J]. Cancer Research, 2015,75(15).

[416] Bonner-Weir S, Sharma A. Lung epithelial stem cells[J]. Journal of Pathology, 2002,197(4):527-535.

[417] Yokohira M, Hashimoto N, Yamakawa K, et al. Lung proliferative lesion-promoting effects of left pulmonary ligation in A/J female mice[J]. PATHOLOGY INTERNATIONAL, 2020,70(6):340-347.

[418] Stoner G D, Adam-Rodwell G, Morse M A. Lung tumors in strain A mice: Application for studies in cancer chemoprevention[J]. Journal of Cellular Biochemistry, 1993,52(SUPPL. 17 F):95-103.

[419] STONER G D, ADAMRODWELL G, MORSE M A. LUNG-TUMORS IN STRAIN A MICE - APPLICATION FOR STUDIES IN CANCER CHEMOPREVENTION[J]. JOURNAL OF CELLULAR BIOCHEMISTRY, 1993:95-103.

[420] Lee S J, Sakurai H, Koizumi K, et al. MAPK regulation and caspase activation are required in DMNQ S-52 induced apoptosis in Lewis lung carcinoma cells[J]. Cancer Letters, 2006,233(1):57-67.

[421] Yu H, Mosher R, Ellison K, et al. MERS67 is a Novel anti-NaPi2b Antibody and Demonstrates Differential Expression Patterns in Lung Cancer Histologic Subtypes[J]. Journal of Thoracic Oncology, 2018,13(10):S770.

[422] Bellamri M, Le Hégarat L, Turesky R, et al. Metabolism and bioactivation of 2-amino-9Hpyrido[ 2,3-b]indole, a tobacco carcinogen, in human hepatocytes[J]. Drug Metabolism Reviews, 2016,48:125-126.

[423] Nathoo N, Toms S A, Barnett G H. Metastases to the brain: Current management perspectives[J]. Expert Review of Neurotherapeutics, 2004,4(4):633-640.

[424] Gadoth A, Kryzer T J, Fryer J, et al. Microtubule-associated protein 1B: Novel paraneoplastic biomarker[J]. Annals of Neurology, 2017,81(2):266-277.

[425] Hirose M, Fukushima S, Imaida K, et al. Modifying effects of phytic acid and γ-Oryzanol on the promotion stage of rat carcinogenesis[J]. Anticancer Research, 1999,19(5 A):3665-3670.

[426] Maestra S L, D'Agostini F, Izzotti A, et al. Modulation by aspirin and naproxen of nucleotide alterations and tumors in the lung of mice exposed to environmental cigarette smoke since birth[J]. Carcinogenesis, 2015,36(12):1531-1538.

[427] Terasaki Y, Suzuki T, Tonaki K, et al. Molecular hydrogen attenuates gefitinib-induced exacerbation of naphthalene-evoked acute lung injury through a reduction in oxidative stress and inflammation[J]. Laboratory Investigation, 2019,99(6):793-806.

[428] Powis G, Wipf P, Lynch S M, et al. Molecular pharmacology and antitumor activity of palmarumycin-based inhibitors of thioredoxin reductase[J]. Molecular Cancer Therapeutics, 2006,5(3):630-636.

[429] Wiedmann M W, Mössner J. Molecular targeted therapy of hepatocellular carcinoma - results of the first clinical studies[J]. Current Cancer Drug Targets, 2011,11(6):714-733.

[430] Castaldi A, Zamani P, Xie M, et al. More than a tight junction: Crucial role of claudin-18 in the response to airway injury[J]. American Journal of Respiratory and Critical Care Medicine, 2019,199(9).

[431] Milione M, Maisonneuve P, Grillo F, et al. Morphology and ki-67 proliferative index drive prognosis in combined large cell neuroendocrine carcinomas of the lung[J]. Modern Pathology, 2020,33(3):1804-1805.

[432] Xia Y, Yeddula N, Ke E, et al. Mouse models of lung cancer mediated by lentiviral gene delivery[J]. Cancer Research, 2014,74(19).

[433] Cruzan G, Bus J, Banton M, et al. Mouse specific lung tumors from CYP2F2-mediated cytotoxic metabolism: An endpoint/toxic response where data from multiple chemicals converge to support a mode of action[J]. Regulatory Toxicology and Pharmacology, 2009,55(2):205-218.

[434] Arakawa K, Endo Y, Kimura M, et al. Multifunctional anti-angiogenic activity of the cyclic peroxide ANO-2 with antitumor activity[J]. International Journal of Cancer, 2002,100(2):220-227.

[435] Suvilesh K N, Manjunath Y, Radhakrishnan V, et al. Multi-omics characterization of tumor tissues from CTC derived xenograft (CDX) mouse models of early-stage NSCLC patients reveals novel diagnostic/therapeutic target(s)[J]. Cancer Research, 2020,80(16 SUPPL).

[436] Gopalan B, Ito I, Branch C D, et al. Nanoparticle based systemic gene therapy for lung cancer: Molecular mechanisms and strategies to suppress nanoparticle-mediated inflammatory response[J]. Technology in Cancer Research and Treatment, 2004,3(6):647-657.

[437] Cline B, Xie J. Nanoscintillator-Based X-Ray-Induced Photodynamic Therapy[Z]. 2022: 2394, 811-822.

[438] Yoshioka Y, Kosaka N, Ochiya T. NAPG can regulate tumour-specific EV secretion[J]. Journal of Extracellular Vesicles, 2017,6:10.

[439] Wu X, He J, Li P, et al. Naphthalene allyl trifluoromethyl benzocyclopentanone inhibits proliferation and induces apoptosis of lung cancer A549 cells in vitro[J]. Nan fang yi ke da xue xue bao = Journal of Southern Medical University, 2022,42(2):201-206.

[440] Jeffrey Lewis R. Naphthalene animal carcinogenicity and human relevancy: Overview of industries with naphthalene-containing streams[J]. Regulatory Toxicology and Pharmacology, 2012,62(1):131-137.

[441] Carratt S A, Morin D, Buckpitt A R, et al. Naphthalene cytotoxicity in microsomal epoxide hydrolase deficient mice[J]. Toxicology Letters, 2016,246:35-41.

[442] Yang Q, Yang P, Qian X, et al. Naphthalimide intercalators with chiral amino side chains: Effects of chirality on DNA binding, photodamage and antitumor cytotoxicity[J]. Bioorganic and Medicinal Chemistry Letters, 2008,18(23):6210-6213.

[443] Accardo A, Morisco A, Tesauro D, et al. Naposomes: A new class of peptide-derivatized, target-selective multimodal nanoparticles for imaging and therapeutic applications[J]. Therapeutic Delivery, 2011,2(2):235-257.

[444] Kumar G, Madka V, Logsdon C, et al. Naproxen blocks spontaneous lung adenoma progression to adenocarcinoma in Kras-G12V mice[J]. Cancer Research, 2019,79(13).

[445] Kumar G, Madka V, Singh A, et al. Naproxen inhibits spontaneous lung adenocarcinoma formation in KrasG12V mice[J]. Neoplasia (United States), 2021,23(6):574-583.

[446] Yokohira M, Kishi S, Yamakawa K, et al. Napsin A is possibly useful marker to predict the tumorigenic potential of lung bronchiolo-alveolar hyperplasia in F344 rats[J]. Experimental and Toxicologic Pathology, 2014,66(2-3):117-123.

[447] Azulay M, Lifshits S, Fridman A, et al. Naptumomab Estafenatox induces T cell recognition, turning anti-PD-1 unresponsive “cold” tumors into “hot” responsive tumors[J]. Cancer Research, 2018,78(13).

[448] Forsberg G, Skartved N J, Wallén-Öhman M, et al. Naptumomab estafenatox, an engineered antibody-superantigen fusion protein with low toxicity and reduced antigenicity[J]. Journal of Immunotherapy, 2010,33(5):492-499.

[449] Rettman P, Blunt M, Bastidas-Legarda L, et al. Natural killer cells target XPO1: a therapeutic opportunity for HCC[J]. Journal of Hepatology, 2020,73:S647-S648.

[450] Qin J J, Nag S, Voruganti S, et al. Natural product MDM2 inhibitors: Anticancer activity and mechanisms of action[J]. Current Medicinal Chemistry, 2012,19(33):5705-5725.

[451] Xiong Y, He L, Shay C, et al. Nck-associated protein 1 associates with HSP90 to drive metastasis in human non-small-cell lung cancer[J]. Journal of Experimental and Clinical Cancer Research, 2019,38(1).

[452] Linnoila I, Li Y. Neuroendocrine transcription factor ascl1 defines multiple progenitors in the lung during the development and repair[J]. Journal of Thoracic Oncology, 2011,6(6):S372-S373.

[453] Reynolds S D, Giangreco A, Power J H T, et al. Neuroepithelial bodies of pulmonary airways serve as a reservoir of progenitor cells capable of epithelial regeneration[J]. American Journal of Pathology, 2000,156(1):269-278.

[454] Neuropeptides 2015 Conference[J]. Neuropeptides, 2017,65.

[455] Mikumo H, Hamada N, Inoshima I, et al. Neutrophil elastase inhibitor sivelestat ameliorates gefitinib, naphthalene-induced acute pneumonitis in mice[J]. European Respiratory Journal, 2015,46.

[456] Mikumo H, Yanagihara T, Hamada N, et al. Neutrophil elastase inhibitor sivelestat ameliorates gefitinib-naphthalene-induced acute pneumonitis in mice[J]. Biochemical and Biophysical Research Communications, 2017,486(1):205-209.

[457] Hudish T M, Opincariu L I, Mozer A B, et al. N-nitroso-trischloroethylurea induces premalignant squamous dysplasia in mice[J]. Journal of Thoracic Oncology, 2011,6(6):S1095-S1096.

[458] Hudish T M, Opincariu L I, Mozer A B, et al. N-nitroso-tris-chloroethylurea induces premalignant squamous dysplasia in mice[J]. Cancer Prevention Research, 2012,5(2):283-289.

[459] Hudish T M, Opincariu L I, Mozer A B, et al. N-nitroso-tris-chloroethylurea induces pre-malignant squamous dysplasia in mice[J]. Proceedings of the American Thoracic Society, 2012,9(2):80.

[460] Tsukinoki R, Murakami Y. Non-communicable disease epidemic: epidemiology in action (EuroEpi 2013 and NordicEpi 2013): Aarhus, Denmark from 11 August to 14 August 2013[J]. European Journal of Epidemiology, 2013,28(1):1-270.

[461] Wang Y, Zhang X, Zhao J, et al. Nonhematotoxic naphthalene diimide modified by polyamine: Synthesis and biological evaluation[J]. Journal of Medicinal Chemistry, 2012,55(7):3502-3512.

[462] Vicker N, Burgess L, Chuckowree I S, et al. Novel angular benzophenazines: Dual topoisomerase I and topoisomerase II inhibitors as potential anticancer agents[J]. Journal of Medicinal Chemistry, 2002,45(3):721-739.

[463] Chu H L, Yip B S, Chen K H, et al. Novel antimicrobial peptides with high anticancer activity and selectivity[J]. PLoS ONE, 2015,10(5).

[464] Shepard J B, Wilkinson R A, Starkey J R, et al. Novel guanide-substituted compounds bind to CXCR4 and inhibit breast cancer metastasis[J]. Anti-Cancer Drugs, 2014,25(1):8-16.

[465] Li X, Bian J, Wang N, et al. Novel naphtho[2,1-d]oxazole-4,5-diones as NQO1 substrates with improved aqueous solubility: Design, synthesis, and in vivo antitumor evaluation[J]. Bioorganic and Medicinal Chemistry, 2016,24(5):1006-1013.

[466] Cole J, Guiot M C, Gravel M, et al. Novel NAPRT specific antibody identifies small cell lung cancer and neuronal cancers as promising clinical indications for a NAMPT inhibitor/niacin co-administration strategy[J]. Oncotarget, 2017,8(44):77846-77859.

[467] Frew T, Powis G, Berggren M, et al. Novel quinone antiproliferative inhibitors of phosphatidylinositol-3-kinase[J]. Anti-Cancer Drug Design, 1995,10(4):347-359.

[468] Yang P, Yang Q, Qian X, et al. Novel synthetic isoquinolino[5,4-ab]phenazines: Inhibition toward topoisomerase I, antitumor and DNA photo-cleaving activities[J]. Bioorganic and Medicinal Chemistry, 2005,13(21):5909-5914.

[469] Yuan Y, Liao Y M, Hsueh C T, et al. Novel targeted therapeutics: Inhibitors of MDM2, ALK and PARP[J]. Journal of Hematology and Oncology, 2011,4.

[470] Park J K, Kang A R, Kwon J H, et al. Novel β-Apopicropodophyllin derivative, JNC1043, exerts anti-cancer and radiosensitizing effects[J]. Cancer Research, 2022,82(12).

[471] Zakaria M A, Rajab N F, Chua E W, et al. NTCU induced pre-malignant and malignant stages of lung squamous cell carcinoma in mice model[J]. SCIENTIFIC REPORTS, 2021,11(1).

[472] Prasad B, Imayama I, Ahmed K, et al. Obstructive Sleep Apnea and Positive Airway Pressure Therapy Use Are Not Associated with Mortality in Veterans with Lung Cancer[J]. AMERICAN JOURNAL OF RESPIRATORY AND CRITICAL CARE MEDICINE, 2019,199.

[473] Adkins Jr. B, Van Stee E W, Simmons J E, et al. Oncogenic response of strain A/J mice to inhaled chemicals[J]. Journal of Toxicology and Environmental Health, 1986,17(2-3):311-322.

[474] Zhang C, Zhang Y, Wang C Y, et al. Pharmacodynamics of neptinib based on the human-derived tumor xenograft (PDX) model of lung adenocarcinoma[J]. Chinese Journal of New Drugs, 2020,29(20):2381-2388.

[475] Quanz M, Merz C, Bernthaler A, et al. Pharmacological characterization of a novel potent nicotinamide phosphoribosyltransferase (NAMPT) inhibitor with robust in vivo efficacy and increased therapeutic index with niacin supplementation[J]. Cancer Research, 2016,76(14).

[476] Beerepoot L V, Radema S A, Witteveen E O, et al. Phase I clinical evaluation of weekly administration of the novel vascular-targeting agent, ZD6126, in patients with solid tumors[J]. Journal of Clinical Oncology, 2006,24(10):1491-1498.

[477] Kao C C, Ho C L, Yang M H, et al. Phase I Targeted Combination Trial of Sorafenib and GW5074 in Patients with Advanced Refractory Solid Tumors[J]. Journal of Clinical Medicine, 2022,11(8).

[478] Chien C M, Yang J C, Wu P H, et al. Phytochemical naphtho[1,2-b] furan-4,5‑dione induced topoisomerase II-mediated DNA damage response in human non-small-cell lung cancer[J]. Phytomedicine, 2019,54:109-119.

[479] Adepoju Y, Epling J, Olajide O. Pituitary metastasis causing polyuria-a rare case of central diabetes insipidus[J]. Endocrine Practice, 2018,24:176.

[480] Doebele R. PL01.01 Tumor-Agnostic Biologically Driven Treatments: An Endless Dream?[J]. Journal of Thoracic Oncology, 2019,14(10):S2.

[481] Hsu Y L, Cho C Y, Kuo P L, et al. Plumbagin (5-hydroxy-2-methyl-1,4-naphthoquinone) induces apoptosis and cell cycle arrest in A549 cells through p53 accumulation via c-Jun NH 2-terminal kinase-mediated phosphorylation at serine 15 in vitro and in vivo[J]. Journal of Pharmacology and Experimental Therapeutics, 2006,318(2):484-494.

[482] Hafeez B B, Zhong W, Fischer J W, et al. Plumbagin, a medicinal plant (Plumbago zeylanica)-derived 1,4-naphthoquinone, inhibits growth and metastasis of human prostate cancer PC-3M-luciferase cells in an orthotopic xenograft mouse model[J]. Molecular Oncology, 2013,7(3):428-439.

[483] Elovaara E, Mikkola J, Stockmann-Juvala H, et al. Polycyclic aromatic hydrocarbon (PAH) metabolizing enzyme activities in human lung, and their inducibility by exposure to naphthalene, phenanthrene, pyrene, chrysene, and benzo(a)pyrene as shown in the rat lung and liver[J]. Archives of Toxicology, 2007,81(3):169-182.

[484] Chen A W, Seleen J. Potential benefits of Ling Zhi or Reishi mushroom Ganoderma lucidum (W. Curt.: Fr.) P. Karst. (Aphyllophoromycetideae) to breast cancer patients[J]. International Journal of Medicinal Mushrooms, 2007,9(1):29-38.

[485] Xing C, Wang Y, Fujioka N, et al. Potential of kava in reducing lung cancer risk, tobacco use, and associated disparities[J]. Cancer Research, 2019,79(13).

[486] Lin K, Rubinfeld B, Zhang C, et al. Preclinical development of an anti-NaPi2b (SLC34A2) antibody-drug conjugate as a therapeutic for non-small cell lung and ovarian cancers[J]. Clinical Cancer Research, 2015,21(22):5139-5150.

[487] Seijo L, Perez-Warnisher M, Cabezas E, et al. Prevalence Of Obstructive Sleep Apnea Among Volunteers Enrolled In A Lung Cancer Screening Program. Results Of The Prospective Sails (sleep Apnea In Lung Cancer Screening) Study[J]. AMERICAN JOURNAL OF RESPIRATORY AND CRITICAL CARE MEDICINE, 2017,195.

[488] Lázaro S, Lorz C, Enguita A B, et al. Pten and p53 Loss in the Mouse Lung Causes Adenocarcinoma and Sarcomatoid Carcinoma[J]. Cancers, 2022,14(15).

[489] Faccin M, Cestari F K, Matos M R, et al. Pulmonary adenocarcinoma in mare[J]. Brazilian Journal of Veterinary Pathology, 2018,11(3):108-112.

[490] Theis J C, Shimkin M B, Weisburger E K. Pulmonary adenoma response of strain A mice to sulfonic acid derivatives of 1- and 2-naphthylamines[J]. Journal of the National Cancer Institute, 1981,67(6):1299-1302.

[491] Alshabani K, Mukhopadhyay S, Lam L S. Rare presentation of sclerosing pneumocytoma manifesting as an endobronchial lesion[J]. American Journal of Respiratory and Critical Care Medicine, 2018,197(MeetingAbstracts).

[492] Gong Q, Yang F, Hu J, et al. Rational designed highly sensitive NQO1-activated near-infrared fluorescent probe combined with NQO1 substrates in vivo: An innovative strategy for NQO1-overexpressing cancer theranostics[J]. European Journal of Medicinal Chemistry, 2021,224.

[493] Tong Y, Yue J, Mao M, et al. Recombinant nematode anticoagulant protein c2 inhibits cell invasion by decreasing uPA expression in NSCLC cells[J]. Oncology Reports, 2015,33(4):1815-1822.

[494] Wang T, Ding C, Ji Z, et al. Recombinant protein MBP-NAP restricts tumor progression by triggering T-cell immunity in mouse metastatic melanoma model[J]. Cancer Research, 2017,77(13).

[495] Wang T, Du M, Ji Z, et al. Recombinant protein rMBP-NAP restricts tumor progression by triggering antitumor immunity in mouse metastatic lung cancer[J]. Canadian Journal of Physiology and Pharmacology, 2018,96(2):113-119.

[496] Rioux N, Castonguay A. Recovery from 4-(methylnitrosamino)-1-(3-pyridyl)-1-butanone-induced immunosuppression in A/J mice by treatment with nonsteroidal anti- inflammatory drugs[J]. Journal of the National Cancer Institute, 1997,89(12):874-880.

[497] Werner J L, Escolero S, Zeng M, et al. Regulation of granuloma formation via Myd88 and Nox2 in a model of pulmonary sarcoidosis[J]. American Journal of Respiratory and Critical Care Medicine, 2014,189.

[498] Werner J, Escolero S, Hewitt J, et al. Regulation of granuloma formation via Myd88 and Nox2 in a model of pulmonary sarcoidosis (MPF5P.741)[J]. Journal of Immunology, 2015,194(1).

[499] Mosher R, Poling L, Qin L, et al. Relationship of NaPi2b expression and efficacy of XMT-1536, a NaPi2b targeting antibody-drug conjugate (ADC), in an unselected panel of human primary ovarian mouse xenograft models[J]. Molecular Cancer Therapeutics, 2018,17(1).

[500] Forkert P G, Parkinson A, Thaete L G, et al. Resistance of murine lung tumors to xenobiotic-induced cytotoxicity[J]. Cancer Research, 1992,52(24):6797-6803.

[501] Thompson P J, Chung F. Respirology year-in-review 2006: Basic science[J]. RESPIROLOGY, 2007,12(2):184-190.

[502] Bosch A, Bertran S P, Lu Y, et al. Reversal by RARα agonist Am580 of c-Myc-induced imbalance in RARα/RARγ expression during MMTV-Myc tumorigenesis[J]. Breast Cancer Research, 2012,14(4).

[503] Campillo N, Torres M, Vilaseca A, et al. Role of Cyclooxygenase-2 on Intermittent Hypoxia-Induced Lung Tumor Malignancy in a Mouse Model of Sleep Apnea[J]. Sci Rep, 2017,7:44693.

[504] Li L, Wang J, Feng L, et al. Rubioncolin C, a natural naphthohydroquinone dimer isolated from Rubia yunnanensis, inhibits the proliferation and metastasis by inducing ROS-mediated apoptotic and autophagic cell death in triple-negative breast cancer cells[J]. Journal of Ethnopharmacology, 2021,277.

[505] Teixeira T M, Arraes I G, Abreu D C, et al. Ruthenium complexes show promise when submitted to toxicological safety tests using alternative methodologies[J]. European Journal of Medicinal Chemistry, 2021,216.

[506] Shimura H, Kodama M, Yoshihara K, et al. Screening of cancer driver genes involved in sarcomagenesis and metastasis of uterine leiomyosarcoma using sleeping beauty transposon[J]. Journal of Obstetrics and Gynaecology Research, 2020,46(8):1564.

[507] Xu X, Zhang J F, Zhang L, et al. Selective detection of monohydroxy metabolites of polycyclic aromatic hydrocarbons in urine using liquid chromatography/triple quadrupole tandem mass spectrometry[J]. RAPID COMMUNICATIONS IN MASS SPECTROMETRY, 2004,18(19):2299-2308.

[508] Verschoyle R D, Martin J, Dinsdale D. Selective inhibition and induction of CYP activity discriminates between the isoforms responsible for the activation of butylated hydroxytoluene and naphthalene in mouse lung[J]. Xenobiotica, 1997,27(8):853-864.

[509] Balansky R, Ganchev G, Iltcheva M, et al. Selective inhibition by aspirin and naproxen of mainstream cigarette smoke-induced genotoxicity and lung tumors in female mice[J]. Archives of Toxicology, 2016,90(5):1251-1260.

[510] Subbiah V, Velcheti V, Tuch B B, et al. Selective RET kinase inhibition for patients with RET-altered cancers[J]. Annals of Oncology, 2018,29(8):1869-1876.

[511] Huang H Y, Lin S W, Chuang L P, et al. SEVERE OBSTRUCTIVE SLEEP APNEA ASSOCIATED WITH STAGE III-IV LUNG CANCER MORTALITY: A COHORT STUDY[J]. SLEEP MEDICINE, 2019,64:S161.

[512] Tang J C, Ren Y G, Zhao J, et al. Shikonin enhances sensitization of gefitinib against wild-type EGFR non-small cell lung cancer via inhibition PKM2/stat3/cyclinD1 signal pathway[J]. Life Sciences, 2018,204:71-77.

[513] Yang H, Zhou P, Huang H, et al. Shikonin exerts antitumor activity via proteasome inhibition and cell death induction in vitro and in vivo[J]. International Journal of Cancer, 2009,124(10):2450-2459.

[514] Dai Y, Liu Y, Li J, et al. Shikonin inhibited glycolysis and sensitized cisplatin treatment in non-small cell lung cancer cells via the exosomal pyruvate kinase M2 pathway[J]. Bioengineered, 2022,13(5):13906-13918.

[515] Wang F, Mayca Pozo F, Tian D, et al. Shikonin Inhibits Cancer Through P21 Upregulation and Apoptosis Induction[J]. Frontiers in Pharmacology, 2020,11.

[516] Lee H J, Lee H J, Magesh V, et al. Shikonin, acetylshikonin, and isobutyroylshikonin inhibit VEGF-induced angiogenesis and suppress tumor growth in Lewis lung carcinoma-bearing mice[J]. Yakugaku Zasshi, 2008,128(11):1681-1688.

[517] Kim H J, Hwang K E, Park D S, et al. Shikonin-induced necroptosis is enhanced by the inhibition of autophagy in non-small cell lung cancer cells[J]. Journal of Translational Medicine, 2017,15(1).

[518] Liu Y, Lao M, Chen J, et al. Short-term prognostic effects of circulating regulatory T-Cell suppressive function and vascular endothelial growth factor level in patients with non-small cell lung cancer and obstructive sleep apnea[J]. Sleep Med, 2020,70:88-96.

[519] Mantareva V, Shopova M, Spassova G, et al. Si(IV)-methoxyethylene-glycol-naphthalocyanine: Synthesis and pharmacokinetic and photosensitizing properties in different tumour models[J]. Journal of Photochemistry and Photobiology B: Biology, 1997,40(3):258-262.

[520] Dean G, Dickerson S, Jungquist C. Sleep and Circadian Activity Rhythms in Individuals with Lung Cancer Compared to Matched Healthy Controls[J]. NURSING RESEARCH, 2016,65(2):E45.

[521] Wang X, Zong X, Li N, et al. Sleep behaviors and risk of lung cancer in the UK Biobank[J]. Cancer Research, 2021,81(13 SUPPL).

[522] Warnisher M, Giraldo-Cadavid L F, Oliveros H, et al. Sleep disordered breathing and nocturnal hypoxemia are associated with an increased risk of lung cancer[J]. EUROPEAN RESPIRATORY JOURNAL, 2018,52.

[523] De Lorenzo B H, Novaes E, Brito R R, et al. Sleep restriction increased murine melanoma lung metastasis formation by reducing the number of cytotoxic cells and inducing a suppressive tumor microenvironment[J]. Brain, Behavior, and Immunity, 2017,66:e40.

[524] Kodama M, Shimura H, Nakae A, et al. Sleeping beauty transposon insertional mutagenesis identified driver genes of uterine leiomyosarcoma[J]. International Journal of Gynecological Cancer, 2018,28:178.

[525] Kodama M, Kodama T, Newberg J Y, et al. Sleeping Beauty transposon mutagenesis screen identifies cancer genes of uterine leiomyosarcoma driving sarcomagenesis and lung metastasis[J]. Cancer Research, 2018,78(13).

[526] Kodama M, Nakae A, Shimura H, et al. Sleeping Beauty transposon mutagenesis screen of uterine leiomyosarcoma identifies driver genes of sarcomagenesis[J]. Cancer Science, 2018,109:1169.

[527] Sishtla K, Corson T W. Small molecules that selectively inhibit growth of MYCNARB1+/+ retinoblastoma cells[J]. Investigative Ophthalmology and Visual Science, 2014,55(13):3073.

[528] Rana B, Harper A, Shen-Tu G, et al. Social jetlag and lung cancer incidence in the Alberta's tomorrow project: A prospective cohort study[J]. Cancer Research, 2020,80(16 SUPPL).

[529] Tsioumpekou M, Cunha S I, Ma H, et al. Specific targeting of PDGFRβ in the stroma inhibits growth and angiogenesis in tumors with high PDGF-BB expression[J]. Theranostics, 2020,10(3):1122-1135.

[530] Kobayashi H, Fukuda Y, Yoshida R, et al. Suppressing effects of dietary supplementation of soybean trypsin inhibitor on spontaneous, experimental and peritoneal disseminated metastasis in mouse model[J]. International Journal of Cancer, 2004,112(3):519-524.

[531] Vere J, Jude E. Syndrome of inappropriate antidiuretic hormone secretion and treatment with tolvaptan a case series[J]. Lung Cancer, 2013,79:S47.

[532] Zeng X, Yang X, Zhang Y, et al. Synthesis and antitumor activity of 1-mesityl-3-(2-naphthoylmethano)-1H-imidazolium bromide[J]. Bioorganic and Medicinal Chemistry Letters, 2010,20(6):1844-1847.

[533] Deady L W, Desneves J, Kaye A J, et al. Synthesis and antitumor activity of some indeno[1,2-b]quinoline-based bis carboxamides[J]. Bioorganic and Medicinal Chemistry, 2000,8(5):977-984.

[534] Li Q, Zhai Y, Luo W, et al. Synthesis and biological properties of polyamine modified flavonoids as hepatocellular carcinoma inhibitors[J]. European Journal of Medicinal Chemistry, 2016,121:110-119.

[535] Deady L W, Rogers M L, Zhuang L, et al. Synthesis and cytotoxic activity of carboxamide derivatives of benzo[b][1,6]naphthyridin-(5H)ones[J]. Bioorganic and Medicinal Chemistry, 2005,13(4):1341-1355.

[536] Deady L W, Rodemann T, Zhuang L, et al. Synthesis and cytotoxic activity of carboxamide derivatives of benzo[b][1,6]naphthyridines[J]. Journal of Medicinal Chemistry, 2003,46(6):1049-1054.

[537] Wang Y, Zhang J, Li M, et al. Synthesis and evaluation of novel amonafide–polyamine conjugates as anticancer agents[J]. Chemical Biology and Drug Design, 2017,89(5):670-680.

[538] Lu Y T, Chen T L, Chang K S, et al. Synthesis of novel C4-benzazole naphthalimide derivatives with potent anti-tumor properties against murine melanoma[J]. Bioorganic and Medicinal Chemistry, 2017,25(2):789-794.

[539] Shelton K L, DeBord M A, Wagers P O, et al. Synthesis, anti-proliferative activity, SAR study, and preliminary in vivo toxicity study of substituted N,N′-bis(arylmethyl)benzimidazolium salts against a panel of non-small cell lung cancer cell lines[J]. Bioorganic and Medicinal Chemistry, 2017,25(1):421-439.

[540] Southerland M R, DeBord M A, Johnson N A, et al. Synthesis, characterization, in vitro SAR study, and preliminary in vivo toxicity evaluation of naphthylmethyl substituted bis-imidazolium salts[J]. Bioorganic and Medicinal Chemistry, 2021,30.

[541] Müller S, Mantareva V, Stoichkova N, et al. Tetraamido-substituted 2,3-naphthalocyanine zinc (II) complexes as phototherapeutic agents: Synthesis, comparative photochemical and photobiological studies[J]. Journal of Photochemistry and Photobiology B: Biology, 1996,35(3):167-174.

[542] Qiao X, Gan M, Wang C, et al. Tetracenomycin X exerts antitumour activity in lung cancer cells through the downregulation of cyclin D1[J]. 2019,17(1).

[543] Ueno T, Elmberger G, Weaver T E, et al. The aspartic protease napsin a suppresses tumor growth independent of its catalytic activity[J]. Laboratory Investigation, 2008,88(3):256-263.

[544] Li J M, Hsu P C, Kuan F C, et al. The cancer stemness inhibitor napabucasin suppresses small cell lung cancer growth through SOX2 expression[J]. American Journal of Cancer Research, 2022,12(10):4637-4651.

[545] Bodyak N D, Mosher R, Yurkovetskiy A V, et al. The dolaflexin-based antibody-drug conjugate XMT-1536 targets the solid tumor lineage antigen SLC34A2/NaPi2b[J]. Molecular Cancer Therapeutics, 2021,20(5):896-905.

[546] Lee K, Johnson V J, Blakley B R. The effect of exposure to a commercial 2,4-D herbicide formulation during gestation on urethan-induced lung adenoma formation in CD-1 mice[J]. Veterinary and Human Toxicology, 2000,42(3):129-132.

[547] Wang Y, Yang W, Pu Q, et al. The effects and mechanisms of SLC34A2 in tumorigenesis and progression of human non-small cell lung cancer[J]. Journal of Biomedical Science, 2015,22(1).

[548] Feng K, Yang X, Ma C, et al. The hydrogel-encapsulated liver X receptor ligand, T0901317, enhances its antitumorigenic effects but eliminates lipogenesis[J]. FASEB Journal, 2019,33(SUPPL 1):793-795.

[549] Donarelli M A. The interaction between alcohol and drugs[J]. Adverse Drug Reaction Bulletin, 2004(226):i-iv.

[550] Vasconcellos M C, Bezerra D P, Fonseca A M, et al. The in-vitro and in-vivo inhibitory activity of biflorin in melanoma[J]. Melanoma Research, 2011,21(2):106-114.

[551] Holik A Z, Filby C E, Pasquet J, et al. The LIM-domain only protein 4 contributes to lung epithelial cell proliferation but is not essential for tumor progression[J]. Respiratory Research, 2015,16(1).

[552] Salem G, Jambeih R, Hussein H, et al. THE LINK BETWEEN OBSTRUCTIVE SLEEP APNEA AND LUNG CANCER: A RETROSPECTIVE CASE CONTROL STUDY[J]. JOURNAL OF INVESTIGATIVE MEDICINE, 2016,64(4).

[553] Van Der Vliet A, Gorissen S, Hristova M, et al. The NADPH oxidase duox1 mediates airway epithelial regeneration following naphthalene-induced injury[J]. American Journal of Respiratory and Critical Care Medicine, 2012,185.

[554] Tirino V, Camerlingo R, Franco R, et al. The role of CD133 in the identification and characterisation of tumour-initiating cells in non-small-cell lung cancer[J]. European Journal of Cardio-thoracic Surgery, 2009,36(3):446-453.

[555] Gao L, Ge C, Wang S, et al. The role of p53-mediated signaling in the therapeutic response of colorectal cancer to 9F, a spermine-modified naphthalene diimide derivative[J]. Cancers, 2020,12(3).

[556] de Bono J S, Concin N, Hong D S, et al. Tisotumab vedotin in patients with advanced or metastatic solid tumours (InnovaTV 201): a first-in-human, multicentre, phase 1–2 trial[J]. The Lancet Oncology, 2019,20(3):383-393.

[557] Abdo K M, Grumbein S, Chou B J, et al. Toxicity and carcinogenicity study in F344 rats following 2 years of whole-body exposure to naphthalene vapors[J]. Inhalation Toxicology, 2001,13(10):931-950.

[558] Kovalchuk N, Zhang Q Y, Kelty J, et al. Toxicokinetic Interaction between Hepatic Disposition and Pulmonary Bioactivation of Inhaled Naphthalene Studied Using Cyp2abfgs-Null and CYP2A13/2F1-Humanized Mice with Deficient Hepatic Cytochrome P450 Activity[J]. Drug metabolism and disposition: the biological fate of chemicals, 2019,47(12):1469-1478.

[559] Gan H, Xu X, Bai Y. Trametes robiniophila represses angiogenesis and tumor growth of lung cancer via strengthening let-7d-5p and targeting NAP1L1[J]. Bioengineered, 2022,13(3):6698-6710.

[560] Hamada N, Yanagihara T, Suzuki K, et al. Treatment with a programmed cell death-1-specific antibody has little effect on afatinib- and naphthalene-induced acute pneumonitis in mice[J]. Biochemical and Biophysical Research Communications, 2017,491(3):656-661.

[561] Preziosi M, Monga S. Understanding the contribution of macrophage-specific Wnts to hepatic tumorigenesis and cancer progression[J]. FASEB Journal, 2017,31(1).

[562] Arteaga De Murphy C, Pedraza-López M, Ferro-Flores G, et al. Uptake of 188Re-β-naphthyl-peptide in cervical carcinoma tumours in athymic mice[J]. Nuclear Medicine and Biology, 2001,28(3):319-326.

[563] Thomas R S, Bao W, Chu T M, et al. Use of short-term transcriptional profiles to assess the long-term cancer-related safety of environmental and industrial chemicals[J]. Toxicological Sciences, 2009,112(2):311-321.

[564] Lo L H, Chiu A P, Li X X, et al. Using transposon elements to elucidate the genetic mechanisms of HCC-associated lung metastases[J]. Cancer Research, 2018,78(13).

[565] Lo L H, Chiu A P, Li X X, et al. Utilizing transposon mutagenesis to elucidate the mechanisms of metastasis-associated HCC[J]. Cancer Science, 2018,109:863.

[566] Jarrett Z S, Lamb C, Houle M. VANISHING CANCER: A CASE OF SMOKING-RELATED ORGANIZING PNEUMONIA[J]. Chest, 2022,162(4):A1766.

[567] Vertrees R A, Zwischenberger J B, Woodson L C, et al. Veno-venous perfusion-induced systemic hyperthermia: Case report with perfusion considerations[J]. Perfusion, 2001,16(3):243-248.

[568] Hoch U, Lynch J, Sato Y, et al. Voreloxin, formerly SNS-595, has potent activity against a broad panel of cancer cell lines and in vivo tumor models[J]. Cancer Chemotherapy and Pharmacology, 2009,64(1):53-65.

[569] Reid T T, Demme R A, Quill T E. When there are no good choices: Illuminating the borderland between proportionate palliative sedation and palliative sedation to unconsciousness[J]. Pain Management, 2011,1(1):31-40.

[570] Miyazaki E, Tsuda T, Abe Y, et al. α-Naphthyl acetate esterase-1 (ANAE-1) secreted by epithelioid cells from induced rabbit lung granuloma showed MIF activity[J]. Experimental Lung Research, 1992,18(6):795-811.

[571] Wu Y, Wang X, Chang S, et al. β-lapachone induces NAD(P)H:quinone oxidoreductase-1- and oxidative stress-dependent heat shock protein 90 cleavage and inhibits tumor growth and angiogenesiss[J]. Journal of Pharmacology and Experimental Therapeutics, 2016,357(3):466-475.

**Part Ⅳ：Non-sleep disorder data(n=2749)**

[1] Chen J B, Kong X F, Lv Y Y, et al. "Real world survey" of hydrogen-controlled cancer: a follow-up report of 82 advanced cancer patients[J]. Med Gas Res, 2019,9(3):115-121.

[2] Gea J. [Basic research in pulmonology][J]. Arch Bronconeumol, 2008,44(11):621-628.

[3] Byszewska D, Broniek A, Jereczek B, et al. [Chemotherapy versus chemoradiotherapy in patients with limited small cell lung carcinoma] CHEMIOTERAPIA VERSUS NAPRZEMIENNA CHEMIO-RADIOTERAPIA U CHORYCH Z ORGANICZONA POSTACIA DROBNOKOMORKOWEGO RAKA PLUCA[J]. 1997,65(5‐6):318-325.

[4] Cai G R, Li P W, Jiao L P. [Clinical observation of music therapy combined with anti-tumor drugs in treating 116 cases of tumor patients][J]. Zhongguo Zhong Xi Yi Jie He Za Zhi, 2001,21(12):891-894.

[5] Boselli G. [Clinical study of the activity and tolerance of hydropropizine, a drug with antitussive activity][J]. Minerva Med, 1972,63(51):2782-2788.

[6] Ishihara Y, Sakai H, Nukariya N, et al. [Development of quality of life (QOL) questionnaire for use of lung cancer patients in palliative therapy--study of validity and reliability no. 2, the effects of chemotherapeutics in QOL][J]. Gan To Kagaku Ryoho, 1995,22(8):1087-1093.

[7] Otsuka H. [Diagnosis of respiratory failure by clinical symptoms][J]. Nihon Naika Gakkai Zasshi, 1990,79(6):706-709.

[8] Losonczy G. [Editorial comment. Thematic issue: Pulmonary medicine][J]. Orv Hetil, 2012,153(23):883.

[9] Yao Y. [Effects of Feiji decoction for soothing the liver combined with psychotherapy on quality of life in primary lung cancer patients][J]. Zhongguo Fei Ai Za Zhi, 2012,15(1):27-33.

[10] Croitoru A, Bogdan M A. [Evidences related to pulmonary rehabilitation in the respiratory pathology][J]. Pneumologia, 2014,63(2):88-90, 92-95.

[11] Kakinuma R, Nishiwaki Y, Yano H, et al. [Experience with psychotropic agents Pyrethia and Contomin in the terminal care of lung cancer patients][J]. Gan No Rinsho, 1984,30(4):344-348.

[12] Beaufort F, Lammer J, Stammberger H. [High-dose methylprednisolone in the treatment of terminal cancer patients][J]. Wien Klin Wochenschr, 1984,96(14):549-551.

[13] Fernández R C, Padierna S C, Villoria F E, et al. [Impact of anxiety and depression on the physical status and daily routines of cancer patients during chemotherapy][J]. Psicothema, 2011,23(3):374-381.

[14] Cao J, Wang Y, Zhang L, et al. [Investigation of the change of quality of life and depression in lung cancer patients before and after chemotherapy][J]. Zhongguo Fei Ai Za Zhi, 2011,14(4):358-361.

[15] Mehić B. [Nicotine addiction][J]. Med Arh, 2001,55(3):165-169.

[16] Wepner U. [Not only a lung disease, but also a systemic disease. COPD as a threat to heart and bones][J]. MMW Fortschr Med, 2004,146(18):4-5.

[17] Kreuter M, Kreuter C, Herth F. [Pneumological aspects of wind instrument performance--physiological, pathophysiological and therapeutic considerations][J]. Pneumologie, 2008,62(2):83-87.

[18] Ishiguro N. [Pulmonary complications of chronic renal failure][J]. Nihon Rinsho, 2004,62 Suppl 6:62-65.

[19] Wachowicz M, Furmanik F. [Quality of life assessment in patients with non-small cell lung cancer treated surgically or with pre-operative chemotherapy followed by surgery][J]. Pneumonol Alergol Pol, 2003,71(3-4):121-131.

[20] Didilescu C. [Respiratory pathology in clinical practice (National Symposium with international participation, second edition), Târgu-Jiu, April 28-29, 2011][J]. Pneumologia, 2011,60(3):180-181.

[21] Lao I, Chen Q, Yu L, et al. [Sarcomatoid malignant mesothelioma: a clinicopathologic and immunohistochemical analysis of 22 cases][J]. Zhonghua bing li xue za zhi Chinese journal of pathology, 2014,43(6):364-369.

[22] Lu J, Ma L, Wang X, et al. [Screening for prodromes of chemotherapy-induced vomiting and correlation between prodromes and chemotherapy-induced vomiting in lung cancer patients][J]. Zhonghua Zhong Liu Za Zhi, 2014,36(7):511-515.

[23] Huber R M, Häussinger K, Pforte A. [Superfluous pneumologic diagnosis][J]. Internist (Berl), 1986,27(9):601-607.

[24] Gu C, Zhai M, Lü A, et al. [Ultrasound-guided stellate ganglion block improves sleep quality in elderly patients early after thoracoscopic surgery for lung cancer: a randomized controlled study][J]. Nan Fang Yi Ke Da Xue Xue Bao, 2022,42(12):1807-1814.

[25] Newton J C, O Connor M, Saunders C, et al. “Who can I ring? Where can I go?” Living with advanced cancer whilst navigating the health system: a qualitative study[J]. Supportive Care in Cancer, 2022,30(8):6817-6826.

[26] Bottiglieri A, Provenzano L, Spagnoletti A, et al. 1056P KRAS and LKB1 mutation conferring prognostic and predictive role on liquid biopsy in advanced NSCLC[J]. Annals of Oncology, 2022,33:S1037-S1038.

[27] Xiang Z, Mo Z, Li G, et al. 125I brachytherapy in the palliation of painful bone metastases from lung cancer after failure or rejection of conventional treatments[J]. Oncotarget, 2016,7(14):18384-18393.

[28] Xiang Z, Wang L, Yan H, et al. 125I seed brachytherapy versus external beam radiation therapy for the palliation of painful bone metastases of lung cancer after one cycle of chemotherapy progression[J]. OncoTargets and Therapy, 2018,11:5183-5193.

[29] Roeland E J, Fintelmann F J, Yang R, et al. 1263O Evaluation of weight gain and overall survival of male vs. female patients with advanced non-small cell lung cancer (NSCLC) receiving first-line chemotherapy[J]. Annals of Oncology, 2022,33:S1125.

[30] Medina J E, Roussos Torres E T, Leal A, et al. 1669P Monitoring immune checkpoint inhibition in advanced solid tumors using genome-wide cfDNA fragmentomes[J]. Annals of Oncology, 2022,33:S1306-S1307.

[31] Roeland E J, Yennu S, Del Fabbro E, et al. 1696TiP Phase Ib study to assess the effect of PF-06946860 on appetite following subcutaneous administration in patients with anorexia and advanced cancer[J]. 2021,32:S1185.

[32] Sun W, Bao J, Lin W, et al. 2-Methoxy-6-acetyl-7-methyljuglone (MAM), a natural naphthoquinone, induces NO-dependent apoptosis and necroptosis by H2O2-dependent JNK activation in cancer cells[J]. Free Radical Biology and Medicine, 2016,92:61-77.

[33] Gettinger S, Schenker M, De Langen J, et al. 2MO First-line (1L) nivolumab (NIVO) + ipilimumab (IPI) in metastatic non-small cell lung cancer (mNSCLC): Clinical outcomes and biomarker analyses from CheckMate 592[J]. Immuno-Oncology and Technology, 2022,16.

[34] Carpagnano G E, Foschino-Barbaro M P, Mule G, et al. 3p microsatellite alterations in exhaled breath condensate from patients with non-small cell lung cancer[J]. AMERICAN JOURNAL OF RESPIRATORY AND CRITICAL CARE MEDICINE, 2005,172(6):738-744.

[35] Nurgalieva A K, Safina S, Shakirova E, et al. 53P Expression of sodium-dependent phosphate transporter NaPi2b is downregulated in malignant ovarian tumors after neoadjuvant chemotherapy[J]. Annals of Oncology, 2021,32:S1361-S1362.

[36] Kim S H, Lu J. 6-(1-Oxobutyl)-5,8-dimethoxy-1,4-naphthoquinone inhibits lewis lung cancer by antiangiogenesis and apoptosis - Reply to the letter to the editor[J]. INTERNATIONAL JOURNAL OF CANCER, 2008,122(10):2404.

[37] Provenzano L, Bottiglieri A, Spagnoletti A, et al. 72P Treatments response in non-small cell lung cancer patients according to BRCA status on liquid biopsy: A retrospective analysis[J]. Immuno-Oncology and Technology, 2022,16.

[38] Lu T, Denehy L, Cao Y, et al. A 12-Week Multi-Modal Exercise Program: Feasibility of Combined Exercise and Simplified 8-Style Tai Chi Following Lung Cancer Surgery[J]. Integrative Cancer Therapies, 2020,19.

[39] Tacha D, Yu C, Bremer R, et al. A 6-Antibody Panel for the Classification of Lung Adenocarcinoma Versus Squamous Cell Carcinoma[J]. APPLIED IMMUNOHISTOCHEMISTRY & MOLECULAR MORPHOLOGY, 2012,20(3):201-207.

[40] Rakshit H, Chatterjee P, Roy D. A bidirectional drug repositioning approach for Parkinson's disease through network-based inference[J]. Biochemical and Biophysical Research Communications, 2015,457(3):280-287.

[41] Kawai S, Watanabe K, Asai M, et al. A case series on the safety of immunotherapy with reduced blood testing frequency in lung cancer patients[J]. International Journal of Clinical Oncology, 2021,26(5):851-857.

[42] Jiang T, Song H, Peng X, et al. A case-control study on non-smoking primary lung cancers in Sichuan, China[J]. Chinese Journal of Lung Cancer, 2010,13(5):511-516.

[43] Long N H, Thanasilp S, Thato R. A causal model for fatigue in lung cancer patients receiving chemotherapy[J]. Eur J Oncol Nurs, 2016,21:242-247.

[44] Zarogoulidis P, Trakada G, Zarogoulidis K. A chrono-target chemotherapy treatment model for lung cancer treatment[J]. Therapeutic Delivery, 2013,4(1):5-8.

[45] Liang J, Wang Y, Zheng L, et al. A Clinical Study on the Use of Yiqi Yangxue Decoction Combined with Chemotherapy to Promote Rapid Postoperative Recovery in Patients with Non-Small Cell Lung Cancer[J]. Emerg Med Int, 2022,2022:7073893.

[46] Sakamoto T, Furukawa T, Pham H H N, et al. A collaborative workflow between pathologists and deep learning for the evaluation of tumour cellularity in lung adenocarcinoma[J]. Histopathology, 2022,81(6):758-769.

[47] Karlsson A, Cirenajwis H, Ericson-Lindquist K, et al. A combined gene expression tool for parallel histological prediction and gene fusion detection in non-small cell lung cancer[J]. Scientific reports, 2019,9(1):5207.

[48] Pozzi P, Munarini E, Bravi F, et al. A combined smoking cessation intervention within a lung cancer screening trial: A pilot observational study[J]. Tumori, 2015,101(3):306-311.

[49] Mason M, Lapuente-Santana Ó, Halkola A S, et al. A Community Challenge to Predict Clinical Outcomes After Immune Checkpoint Blockade in Non-Small Cell Lung Cancer[Z]. 2022.

[50] Kavathiya K, Gurjar M, Patil A, et al. A Comparative Pharmacokinetic Study of 2 Pemetrexed Formulations in Indian Adult Chemonaive Patients With Adenocarcinoma Stage III/IV Non-Small Cell Lung Cancer[J]. 2017,6(3):234-239.

[51] Prabhakaran S, Xing G, Hocking A, et al. A comparison of diagnostic panels in the immunohistochemical analysis of lung cancer[J]. PATHOLOGY AND LABORATORY MEDICINE INTERNATIONAL, 2019,11:7-15.

[52] Depierre A, Lebeau B, D'Allens H. A comparison of ondansetron with alizapride plus methylprednisolone in the control of cisplatin-induced emesis[J]. Oncology, 1992,49(4):305-311.

[53] Vanderbyl B L, Mayer M J, Nash C, et al. A comparison of the effects of medical Qigong and standard exercise therapy on symptoms and quality of life in patients with advanced cancer[J]. Supportive Care in Cancer, 2017,25(6):1749-1758.

[54] Koh J, Go H, Kim M Y, et al. A comprehensive immunohistochemistry algorithm for the histological subtyping of small biopsies obtained from non-small cell lung cancers[J]. Histopathology, 2014,65(6):868-878.

[55] Harle A, Molassiotis A, Buffin O, et al. A cross sectional study to determine the prevalence of cough and its impact in patients with lung cancer: a patient unmet need[J]. BMC Cancer, 2020,20(1):9.

[56] Niiranen A, Mattson K. A cross-over comparison of nabilone and prochlorperazine for emesis induced by cancer chemotherapy[J]. American Journal of Clinical Oncology: Cancer Clinical Trials, 1985,8(4):336-340.

[57] Ali M, Grever M R. A cytotoxic naphthoquinone from Lawsonia inermis[J]. Fitoterapia, 1998,69(2):181-183.

[58] Maestri T, Anderson D, Calderon-Abbo J, et al. A description of antipsychotic prescribing patterns based on race in the inpatient behavioral health setting[J]. Therapeutic Advances in Psychopharmacology, 2021,11.

[59] A double-blind and randomized controlled clinical trial of traditional Chinese medicine combined with adjuvant chemotherapy for early stage non-small cell lung cancer[J]. 2016.

[60] Goldman J W, Raju R N, Gordon G A, et al. A first in human, safety, pharmacokinetics, and clinical activity phase I study of once weekly administration of the Hsp90 inhibitor ganetespib (STA-9090) in patients with solid malignancies[J]. BMC Cancer, 2013,13.

[61] Infante J R, Mendelson D S, Burris H A, et al. A first-in-human dose-escalation study of the oral proteasome inhibitor oprozomib in patients with advanced solid tumors[J]. Investigational New Drugs, 2016,34(2):216-224.

[62] Delzell E, Macaluso M, Cole P. A follow-up study of workers at a dye and resin manufacturing plant[J]. Journal of Occupational Medicine, 1989,31(3):273-278.

[63] Kim D W, Blackhall F, Soria J C, et al. A global phase 2 study including efficacy, safety and patientreported outcomes (PROS) with crizotinib in patients (pts) with ALK-positive non-small cell lung cancer (NSCLC)[J]. European Journal of Cancer, 2011,47:S617.

[64] Duse G. A good treatment of pain to improve quality of life in cancer patients[J]. Pain Practice, 2009,9:52-53.

[65] Hermann C P, Keeney C E, Looney S W. A Longitudinal Perspective of the Symptom Experience of Patients With Lung Cancer Near the End of Life[J]. JOURNAL OF HOSPICE & PALLIATIVE NURSING, 2016,18(2):100-107.

[66] Chiu H W, Hung S W, Chiu C F, et al. A Mitochondrion-Targeting Protein (B2) Primes ROS/Nrf2-Mediated Stress Signals, Triggering Apoptosis and Necroptosis in Lung Cancer[J]. BIOMEDICINES, 2023,11(1).

[67] Butts C, Murray R N, Smith C, et al. A multicenter open-label study to assess the safety of a new formulation of BLP25 liposome vaccine in patients with unresectable stage III non-small-cell lung cancer[J]. Clinical Lung Cancer, 2010,11(6):391-395.

[68] Raez L E, Santos E S, Webb R T, et al. A multicenter phase II study of docetaxel, oxaliplatin, and bevacizumab in first-line therapy for unresectable locally advanced or metastatic non-squamous cell histology non-small-cell lung cancer (NSCLC)[J]. Cancer Chemotherapy and Pharmacology, 2013,72(5):1103-1110.

[69] Nakagawa R, Tateishi H, Radwan M O, et al. A New 1,2-Naphthoquinone Derivative with Anti-lung Cancer Activity[J]. Chemical and Pharmaceutical Bulletin, 2022,70(7):477-482.

[70] Pérez-Melero C, Maya A B S, Del Rey B, et al. A new family of quinoline and quinoxaline analogues of combretastatins[J]. Bioorganic and Medicinal Chemistry Letters, 2004,14(14):3771-3774.

[71] Cainap C, Cetean S, Havasi A, et al. A new protocol of desensitization for systemic therapy agents in oncology[J]. Journal of B.U.ON., 2021,26(6):2635-2642.

[72] Tang A, Savarimuthu S, Jay M. A new role for multivitamins?[J]. Journal of Clinical Outcomes Management, 2013,20(5):201-202.

[73] Wani T A, Darwish I A. A novel 96-microwell-based high-throughput spectrophotometric assay for pharmaceutical quality control of crizotinib, a novel potent drug for the treatment of non-small cell lung cancer[J]. Brazilian Journal of Pharmaceutical Sciences, 2015,51(2):439-447.

[74] Jefford M, Michael M, Rosenthal M A, et al. A novel combination of cisplatin, irinotecan, and capecitabine in patients with advanced cancer[J]. Investigational New Drugs, 2004,22(2):185-192.

[75] Viscardi G, Sparano F, Di Liello R, et al. A novel ImmunoScore, based on clinical and blood biomarkers, as prognostic model for immunotherapy in NSCLC[J]. 2019,30:i31.

[76] Zhang G H, An Y F, Lu X, et al. A Novel Naphthalimide Compound Restores p53 Function in Non-small Cell Lung Cancer by Reorganizing the Bak center dot Bcl-xl Complex and Triggering Transcriptional Regulation[J]. JOURNAL OF BIOLOGICAL CHEMISTRY, 2016,291(8):4211-4225.

[77] Fujisaka Y, Yamada Y, Yamamoto N, et al. A Phase 1 clinical study of temsirolimus (CCI-779) in Japanese patients with advanced solid tumors[J]. Jpn J Clin Oncol, 2010,40(8):732-738.

[78] Hann C L, Burns T F, Dowlati A, et al. A Phase 1 Study Evaluating Rovalpituzumab Tesirine in Frontline Treatment of Patients With Extensive-Stage SCLC[J]. Journal of Thoracic Oncology, 2021,16(9):1582-1588.

[79] Gordon M S, Mendelson D, Carr R, et al. A phase 1 trial of 2 dose schedules of ABT-510, an antiangiogenic, thrombospondin-1-mimetic peptide, in patients with advanced cancer[J]. Cancer, 2008,113(12):3420-3429.

[80] Becerra C, Hanna W T, Richey S L, et al. A phase 1b/2 study of napabucasin with weekly paclitaxel in advanced, previously treated non-squamous non-small cell lung cancer.[J]. JOURNAL OF CLINICAL ONCOLOGY, 2017,35.

[81] Ahn S H, Lim Y S, Lee K S, et al. A phase 3b study of sofosbuvir plus ribavirin in treatment-naive and treatment-experienced Korean patients chronically infected with genotype 2 hepatitis C virus[J]. Journal of Viral Hepatitis, 2016,23(5):358-365.

[82] Crabb S J, Bradbury J, Nolan L, et al. A phase i clinical trial of irinotecan and carboplatin in patients with extensive stage small cell lung cancer[J]. Chemotherapy, 2012,58(4):257-263.

[83] Cohen R B, Jones S F, Aggarwal C, et al. A phase I dose-escalation study of danusertib (PHA-739358) administered as a 24-hour infusion with and without granulocyte colony-stimulating factor in a 14-day cycle in patients with advanced solid tumors[J]. Clinical Cancer Research, 2009,15(21):6694-6701.

[84] Dugan E, Truax R, Meadows K L, et al. A phase I dose-escalation study of imatinib mesylate (Gleevec/STI571) plus capecitabine (Xeloda) in advanced solid tumors[J]. Anticancer Research, 2010,30(4):1251-1256.

[85] Becerra C R, Conkling P, Vogelzang N, et al. A phase I dose-escalation study of MEDI-575, a PDGFRα monoclonal antibody, in adults with advanced solid tumors[J]. Cancer Chemotherapy and Pharmacology, 2014,74(5):917-925.

[86] Chiorean E G, LoRusso P, Strother R M, et al. A phase I first-in-human study of enoticumab (REGN421), a fully human delta-like ligand 4 (Dll4) monoclonal antibody in patients with advanced solid tumors[J]. Clinical Cancer Research, 2015,21(12):2695-2703.

[87] Heath E I, Forman K, Malburg L, et al. A phase i pharmacokinetic and safety evaluation of oral pazopanib dosing administered as crushed tablet or oral suspension in patients with advanced solid tumors[J]. Investigational New Drugs, 2012,30(4):1566-1574.

[88] Tarhini A A, Belani C P, Luketich J D, et al. A phase i study of concurrent chemotherapy (paclitaxel and carboplatin) and thoracic radiotherapy with swallowed manganese superoxide dismutase plasmid liposome protection in patients with locally advanced stage III non-small-cell lung cancer[J]. Human Gene Therapy, 2011,22(3):336-342.

[89] Burris H A, Gordon M S, Gerber D E, et al. A phase I study of DNIB0600A, an antibody-drug conjugate (ADC) targeting NaPi2b, in patients (pts) with non-small cell lung cancer (NSCLC) or platinum-resistant ovarian cancer (OC)[J]. Journal of Clinical Oncology, 2014,32(15).

[90] Choi H J, Sohn J H, Lee C G, et al. A phase I study of nimotuzumab in combination with radiotherapy in stages IIB-IV non-small cell lung cancer unsuitable for radical therapy: Korean results[J]. Lung Cancer, 2011,71(1):55-59.

[91] Forster M, Hackshaw A, De Pas T, et al. A phase I study of nintedanib combined with cisplatin/gemcitabine as first-line therapy for advanced squamous non-small cell lung cancer (LUME-Lung 3)[J]. Lung Cancer, 2018,120:27-33.

[92] Gordon M S, Gerber D E, Infante J R, et al. A phase I study of the safety and pharmacokinetics of DNIB0600A, an anti-NaPi2b antibody-drug-conjugate (ADC), in patients (pts) with non- small cell lung cancer (NSCLC) and platinum-resistant ovarian cancer (OC)[J]. Journal of Clinical Oncology, 2013,31(15).

[93] Blackhall F H, Obrien M, Schmid P, et al. A phase i study of vandetanib in combination with vinorelbine/cisplatin or gemcitabine/cisplatin as first-line treatment for advanced non-small cell lung cancer[J]. Journal of Thoracic Oncology, 2010,5(8):1285-1288.

[94] Iyer G, Morris M J, Rathkopf D, et al. A phase I trial of docetaxel and pulse-dose 17-allylamino-17- demethoxygeldanamycin in adult patients with solid tumors[J]. Cancer Chemotherapy and Pharmacology, 2012,69(4):1089-1097.

[95] Chiappori A A, Schreeder M T, Moezi M M, et al. A phase i trial of pan-Bcl-2 antagonist obatoclax administered as a 3-h or a 24-h infusion in combination with carboplatin and etoposide in patients with extensive-stage small cell lung cancer[J]. British Journal of Cancer, 2012,106(5):839-845.

[96] Blumenthal G M, Gills J J, Ballas M S, et al. A phase I trial of the HIV protease inhibitor nelfinavir in adults with solid tumors[J]. Oncotarget, 2014,5(18):8161-8172.

[97] Lim W T, Baggstrom M Q, Read W, et al. A Phase I trial of weekly docetaxel and topotecan for solid tumors[J]. Acta Oncologica, 2008,47(2):311-315.

[98] Kjellen E, Pero R W, Brun E, et al. A phase I/II evaluation of metoclopramide as a radiosensitiser in patients with inoperable squamous cell carcinoma of the lung[J]. European Journal of Cancer Part A: General Topics, 1995,31(13-14):2196-2202.

[99] Grilley-Olson J E, Bedard P L, Fasolo A, et al. A phase Ib dose-escalation study of the MEK inhibitor trametinib in combination with the PI3K/mTOR inhibitor GSK2126458 in patients with advanced solid tumors[J]. Investigational New Drugs, 2016,34(6):740-749.

[100] Grilley-Olson J E, Villaruz L C, Stinchcombe T E, et al. A Phase Ib study of bavituximab plus carboplatin and pemetrexed in chemotherapy-naïve incurable stage IIIb/IV non-squamous non-small cell lung cancer[J]. Molecular Cancer Therapeutics, 2013,12(11).

[101] Weiss G J, Waypa J, Blaydorn L, et al. A phase Ib study of pembrolizumab plus chemotherapy in patients with advanced cancer (PembroPlus)[J]. British Journal of Cancer, 2017,117(1):33-40.

[102] Chiappori A, Schreeder M T, Moezi M M, et al. A phase Ib trial of Bcl-2 inhibitor obatoclax in combination with carboplatin and etoposide for previously untreated patients with extensive-stage small cell lung cancer (ES-SCLC)[J]. Journal of Clinical Oncology, 2009,27(15):3576.

[103] Becerra C, Spira A I, Conkling P R, et al. A phase Ib/II study of cancer stemness inhibitor napabucasin (BB608) combined with weekly paclitaxel in advanced non-small cell lung cancer[J]. Journal of Clinical Oncology, 2016,34.

[104] Davis M, Lasheen W, Walsh D, et al. A phase II dose titration study of thalidomide for cancer-associated anorexia[J]. Journal of Pain and Symptom Management, 2012,43(1):78-86.

[105] Chen H, Modiano M R, Neal J W, et al. A phase II multicentre study of ziv-aflibercept in combination with cisplatin and pemetrexed in patients with previously untreated advanced/metastatic non-squamous non-small cell lung cancer[J]. British Journal of Cancer, 2014,110(3):602-608.

[106] Jhaveri K, Chandarlapaty S, Lake D, et al. A phase II open-label study of ganetespib, a novel heat shock protein 90 inhibitor for patients with Metastatic breast cancer[J]. Clinical Breast Cancer, 2014,14(3):154-160.

[107] Candelaria M, Gallardo-Rincón D, Arce C, et al. A phase II study of epigenetic therapy with hydralazine and magnesium valproate to overcome chemotherapy resistance in refractory solid tumors[J]. Annals of Oncology, 2007,18(9):1529-1538.

[108] Minor D R, Monroe D, Damico L A, et al. A phase II study of thalidomide in advanced metastatic renal cell carcinoma[J]. Investigational New Drugs, 2002,20(4):389-393.

[109] Gandhi L, Harding M W, Neubauer M, et al. A phase II study of the safety and efficacy of the multidrug resistance inhibitor VX-710 combined with doxorubicin and vincristine in patients with recurrent small cell lung cancer[J]. Cancer, 2007,109(5):924-932.

[110] Iwata T, Yoshino I, Yoshida S, et al. A phase II trial evaluating the efficacy and safety of perioperative pirfenidone for prevention of acute exacerbation of idiopathic pulmonary fibrosis in lung cancer patients undergoing pulmonary resection: West Japan Oncology Group 6711 L (PEOPLE Study)[J]. Respiratory Research, 2016,17(1).

[111] Gerena-Lewis M, Crawford J, Bonomi P, et al. A phase II trial of denileukin diftitox in patients with previously treated advanced non-small cell lung cancer[J]. American Journal of Clinical Oncology: Cancer Clinical Trials, 2009,32(3):269-273.

[112] Blakely L J, Schwartzberg L, Keaton M, et al. A phase II trial of pemetrexed and gemcitabine as first line therapy for poor performance status and/or elderly patients with stage IIIB/IV non-small cell lung cancer[J]. Lung Cancer, 2009,66(1):97-102.

[113] Neuhaus T, Ko Y, Muller R P, et al. A phase III trial of topotecan and whole brain radiation therapy for patients with CNS-metastases due to lung cancer[J]. British Journal of Cancer, 2009,100(2):291-297.

[114] Davies B H, Weatherstone R M, Graham J D P, et al. A pilot study of orally administered Δ trans tetrahydrocannabinol in the management of patients undergoing radiotherapy for carcinoma of the bronchus[J]. BRIT.J.CLIN.PHARMACOL., 1974,1(4):301-306.

[115] Fouladbakhsh J M, Davis J E, Yarandi H N. A pilot study of the feasibility and outcomes of yoga for lung cancer survivors[J]. Oncol Nurs Forum, 2014,41(2):162-174.

[116] Bedikian A Y, Papadopoulos N E, Kim K B, et al. A pilot study with vincristine sulfate liposome infusion in patients with metastatic melanoma[J]. Melanoma Research, 2008,18(6):400-404.

[117] Parikh A B, Marrone K A, Becker D J, et al. A pooled analysis of two phase II trials evaluating metformin plus platinum-based chemotherapy in advanced non-small cell lung cancer[J]. Cancer Treatment and Research Communications, 2019,20.

[118] Wen J H, Li C Y, Geng Z R, et al. A potent antitumor Zn2+ tetraazamacrocycle complex targeting DNA: The fluorescent recognition, interaction and apoptosis studies[J]. Chemical Communications, 2011,47(40):11330-11332.

[119] Jing Y, Bai L, Chen Z, et al. A prediction model of polycyclic aromatic hydrocarbon quarterly emissions based on GDP from a government policy perspective[J]. Indoor and Built Environment, 2022,31(2):552-567.

[120] Cahill K, Stead L, Lancaster T. A preliminary benefit-risk assessment of varenicline in smoking cessation[J]. Drug Safety, 2009,32(2):119-135.

[121] Tsai M Y, Hung Y C, Chen Y H, et al. A preliminary randomised controlled study of short-term Antrodia cinnamomea treatment combined with chemotherapy for patients with advanced cancer[J]. BMC Complementary and Alternative Medicine, 2016,16(1).

[122] Tabudlo J B. A Proposed Theory of Symptom Cluster Management[J]. BELITUNG NURSING JOURNAL, 2021,7(2):78-87.

[123] Song E K, Shim H, Han H S, et al. A prospective multicentre study to evaluate the efficacy and tolerability of osmotic release oral system (OROS) hydromorphone in opioid-naive cancer patients: Results of the Korean South West Oncology Group study[J]. Pain Research and Management, 2015,20(6):293-299.

[124] Lemos R, Areias-Marques S, Ferreira P, et al. A prospective observational study for a Federated Artificial Intelligence solution for moniToring mental Health status after cancer treatment (FAITH): study protocol[J]. BMC Psychiatry, 2022,22(1).

[125] Katpattil S. A prospective study on quality of life among persons with lung cancer, before and after the chemotherapy treatment - Evidence from South India[J]. European Journal of Cancer, 2017,72:S164.

[126] George A M, Arya M A, Joseph S K, et al. A Prospective Study on the Incidence and Severity of Paclitaxel-induced Peripheral Neuropathy in the Indian Population[J]. Current Cancer Therapy Reviews, 2022,18(4):278-284.

[127] Tang Y, Yu F, Zhang G, et al. A Purified Serine Protease from Nereis virens and Its Impaction of Apoptosis on Human Lung Cancer Cells[J]. Molecules, 2017,22(7).

[128] Ekfors H, Petersson K. A qualitative study of the experiences during radiotherapy of Swedish patients suffering from lung cancer[J]. Oncol Nurs Forum, 2004,31(2):329-334.

[129] Das A, Gayatri Devi R, Jothi Priya A. A questionnaire-based study on effect of chemotherapy treatment in the body[J]. Drug Invention Today, 2018,10(10):2070-2072.

[130] Langer C J, Albert I, Kovacs P, et al. A randomized phase II study of carboplatin (C) and etoposide (E) with or without pan-BCL-2 antagonist obatoclax (Ob) in extensive-stage small cell lung cancer (ES-SCLC)[J]. Journal of Clinical Oncology, 2011,29(15).

[131] Hawkins R E, Gore M, Shparyk Y, et al. A Randomized Phase II/III Study of Naptumomab Estafenatox plus IFN alpha versus IFN alpha in Renal Cell Carcinoma: Final Analysis with Baseline Biomarker Subgroup and Trend Analysis[J]. CLINICAL CANCER RESEARCH, 2016,22(13):3172-3181.

[132] Crawford J, Robert F, Perry M C, et al. A randomized trial comparing immediate versus delayed treatment of anemia with once-weekly epoetin alfa in patients with non-small cell lung cancer Scheduled to Receive First-Line Chemotherapy[J]. Journal of Thoracic Oncology, 2007,2(3):210-220.

[133] He Q, Yi T, Luo B, et al. A randomized trial of NVB plus DDP with versus without thalidomide in the treatment of advanced non small cell lung cancer[J]. Chinese Journal of Lung Cancer, 2008,11(2):264-267.

[134] EUCTR IT. A randomized, non-comparative, phase II study investigating the best epidermal growth factor receptor tyrosine kinase inhibitor (EGFR-TKI) sequence in advanced or metastatic non-small-cell lung cancer (NSCLC) harboring EGFR mutations[J]. 2021.

[135] Liu K J, Wu H Y. A retrospective analysis of cisplatin, pemetrexed, and bevacizumab in previously treated non-small-cell lung cancer[J]. Oncotarget, 2015,6(26):22750-22757.

[136] Datta S, Zosangpuii C, Ningthoujam G, et al. A retrospective study on adverse drug reactions of anticancer drugs in a tertiary care hospital in Northeast India[J]. Journal of Clinical and Diagnostic Research, 2021,15(11):C1-C5.

[137] Saito O, Miyasato A. A retrospective study on naproxen, rather than loxoprofen, for cancer pain[J]. Chiba Medical Journal, 2020,96E:55-58.

[138] Hughes S, Haffey S, McConville J, et al. A reversible cause of fasciculation, muscle wasting and encephalopathy[J]. Journal of Neurology, Neurosurgery and Psychiatry, 2010,81(11):e64.

[139] Tabares J, Santos R, Cassiano J L, et al. A Ru(II)-p-cymene compound bearing naproxen-pyridineamide. Synthesis, spectroscopic studies, computational analysis and in vitro anticancer activity against lung cells compared to Ru(II)-p-cymene-naproxen and the corresponding drug ligands[J]. INORGANICA CHIMICA ACTA, 2019,489:27-38.

[140] Baine M K, Sinard J H, Cai G, et al. A semi-quantitative approach to biopsy diagnosis of large cell neuroendocrine carcinoma of the lung[J]. Journal of Thoracic Oncology, 2017,12(8):S1543.

[141] Baine M K, Sinard J H, Cai G, et al. A Semiquantitative Scoring System May Allow Biopsy Diagnosis of Pulmonary Large Cell Neuroendocrine Carcinoma[J]. American Journal of Clinical Pathology, 2020,153(2):165-174.

[142] Brough D N, Abel S, Priddle L. A service evaluation of a community project combining psychoeducation and mind-body complementary approaches to support those with long covid in the UK[J]. European Journal of Integrative Medicine, 2022,55.

[143] Suzuki K, Watanabe S I, Wakabayashi M, et al. A single-arm study of sublobar resection for ground-glass opacity dominant peripheral lung cancer[J]. Journal of Thoracic and Cardiovascular Surgery, 2022,163(1):289-301.

[144] Kim H R, Lee S J, Park S, et al. A Single-Arm, Prospective, Phase II Study of Cisplatin Plus Weekly Docetaxel as First-Line Therapy in Patients with Metastatic or Recurrent Salivary Gland Cancer[J]. Cancer Research and Treatment, 2022,54(3):719-727.

[145] Wu C N, Wu X H, Yu D N, et al. A single-dose of stellate ganglion block for the prevention of postoperative dysrhythmias in patients undergoing thoracoscopic surgery for cancer A randomised controlled double-blind trial[J]. EUROPEAN JOURNAL OF ANAESTHESIOLOGY, 2020,37(4):323-331.
[truncated: 1,012,329 more chars]
